# Improving a Methane C–H Activation Complex by Metal and Ligand Alterations from Computational Results

**DOI:** 10.1021/acs.inorgchem.2c03342

**Published:** 2023-03-22

**Authors:** Dragan B. Ninković, Salvador Moncho, Predrag Petrović, Michael B. Hall, Snežana D. Zarić, Edward N. Brothers

**Affiliations:** † Department of Chemistry, Texas A&M University at Qatar, P.O. Box 23874 Doha, Qatar; ‡ University of Belgrade – Faculty of Chemistry, Studentski trg 12-16, Belgrade 11000, Serbia; § Department of Chemistry, Texas A&M University College Station, College Station, Texas 77843-3255, United States; ∥ Innovation Center of the Faculty of Chemistry, University of Belgrade, Belgrade 11000, Serbia

## Abstract

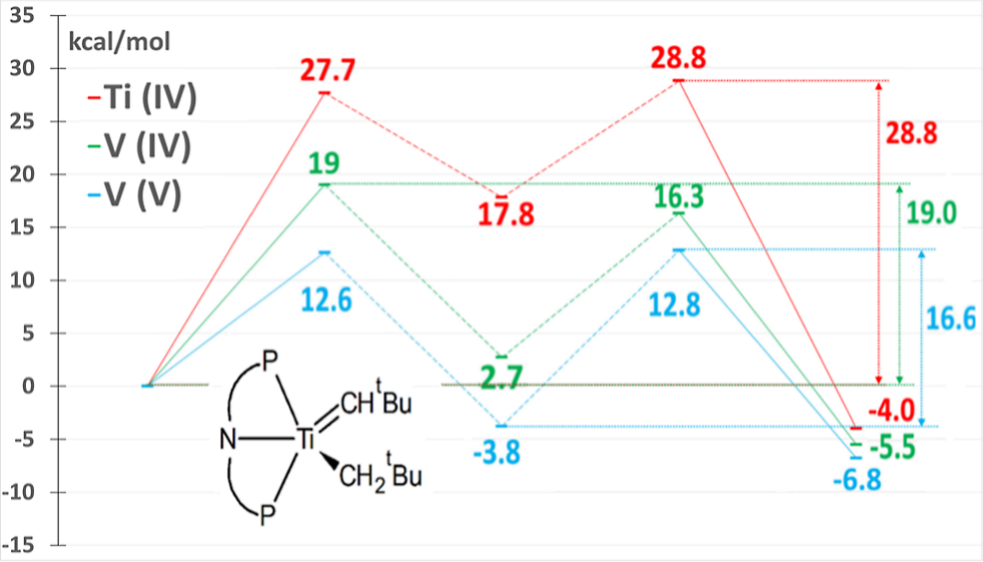

We present results for a series of complexes derived from a titanium complex capable of activating C–H bonds under mild conditions (PNP)Ti=CH^*t*^Bu(CH_2_^*t*^Bu), where PNP = N[2-P^*i*^Pr_2_-4-methylphenyl]^2–^. In addition to the initial activation of methane, a tautomerization reaction to a terminal methylidene is also explored due to methylidene’s potential use as a synthetic starting point. Analogous complexes with other low-cost 3d transition metals were studied, such as scandium, titanium, vanadium, and chromium as both isoelectronic and isocharged complexes. Our results predict that V^(IV)^ and V^(V)^ complexes are promising for methane C–H bond activation. The V^(V)^ complex has a low rate-determining barrier for methane activation, specifically 16.6 kcal/mol, which is approximately 12 kcal/mol less than that for the Ti complex, as well as having a moderate tautomerization barrier of 29.8 kcal/mol, while the V^(IV)^ complex has a methane activation barrier of 19.0 kcal/mol and a tautomerization barrier of 31.1 kcal/mol. Scandium and chromium complexes are much poorer for C–H bond activation; scandium has very high barriers, while chromium strongly overstabilizes the alkylidene intermediate, potentially stopping the further reaction. In addition to the original PNP ligand, some of the most promising ligands from a previous work were tested, although (as shown previously) modification of the ligand does not typically have large effects on the activity of the system. Our best ligand modification improves the performance of the V^(V)^ complex via the substitution of the nitrogen in PNP by phosphorus, which reduces the tautomerization barrier by 5 to 24.4 kcal/mol.

## Abstract 2

A series of complexes derived from a titanium complex capable of activating C−H bonds under mild conditions were studied. Analogous complexes with other low-cost 3d transition metals were studied, such as scandium, titanium, vanadium, and chromium, both isoelectronic and isocharged complexes. Our results predict that V^(IV)^ and V^(V)^ complexes are promising for methane C−H bond activation. These complexes have a low rate-determining barrier for methane activation, 19.0 and 16.6 kcal/mol, respectively.

## 1 Introduction

Due to the inertness of the carbon–hydrogen bond (C–H), converting natural gas to added-value chemicals is a challenging process. Despite 50 years of work in this field, the ideal transition-metal catalyst for C–H bond activation, practical at low temperatures and with good selectivity, is still elusive.^1−9^ Computational studies of the mechanism of transition-metal-driven C–H bond activation describe different mechanistic families, such as oxidative addition, σ-bond metathesis, radical bond homolysis, electrophilic reactions, 1,3-addition, and 1,2-addition reactions.^10−19^ Among the activation of C–H bonds via 1,2-addition reactions, those achieved by complexes that have metal–carbon multiple bonds (both carbenes and carbynes) are of interest here.^9,20−23^ An example is the transient titanium alkylidyne formed from the titanium neopentylidene complex (PNP)Ti=CH^*t*^Bu(CH_2_^*t*^Bu) (PNP = N[2-P^*i*^Pr_2_-4-methylphenyl]^2–^), generated via an abstraction reaction. The transient titanium alkylidyne complex can activate both sp^2^ and sp^3^ C–H bonds under mild conditions.^24,25^ Among other advantages of this complex, titanium is less expensive than other transition metals commonly used in catalysis and does not require photochemical activation.

**Figure 1:**
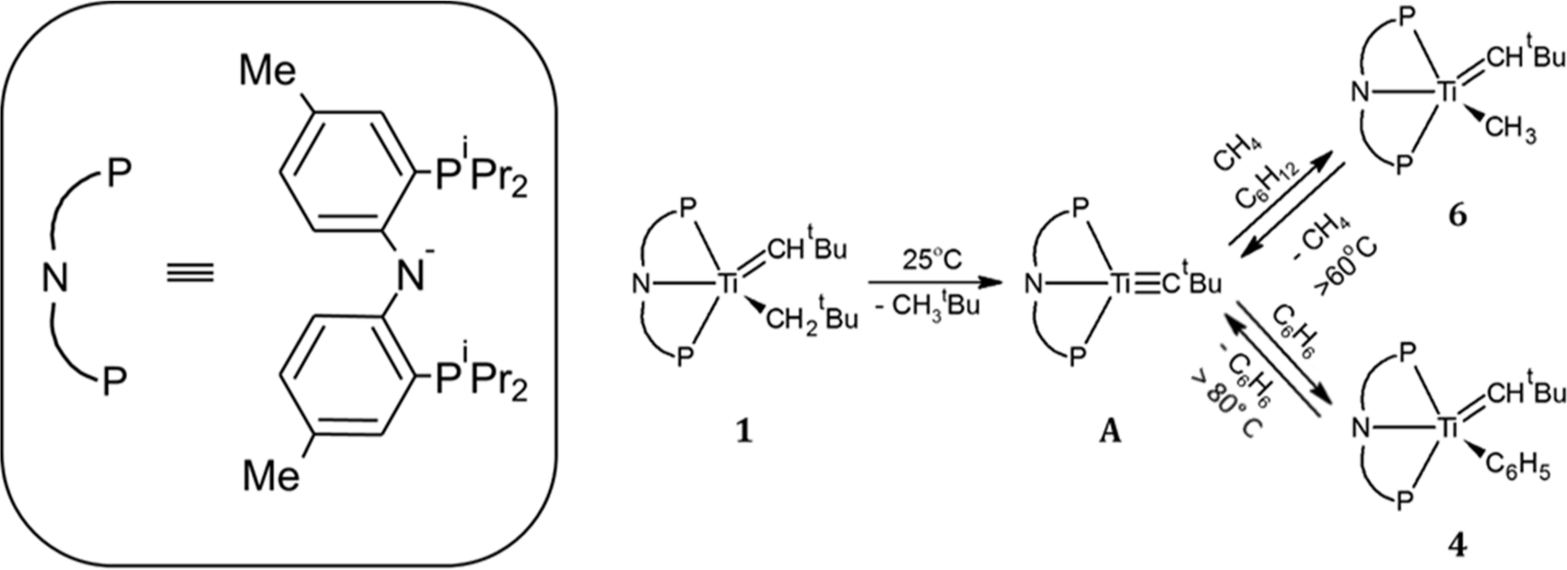
Schematic representation of the PNP ligand (N[2-P^*i*^Pr2-4-methylphenyl]^2–^) (left); C–H bond activation reaction of benzene and methane^24,25^ (right).

As proposed by Mindiola and co-workers,^24,25^ the titanium neopentylidene complex (PNP)Ti=CH^*t*^Bu(CH_2_^*t*^Bu) undergoes reverse hydrogen abstraction and forms the titanium alkylidyne intermediate **A** (Figure 1) and neopentane. Intermediate **A** is capable of activating both benzene^25^ and methane^24^ at room temperatures via 1,2-addition across the alkylidyne–titanium bond in A, forming (PNP)Ti=CH^*t*^Bu(C_6_H_5_) and (PNP)Ti=CH^*t*^Bu(CH_3_) complexes, respectively. Both experiments and calculations suggest the involvement of the titanium-carbon triple bond [(PNP)Ti≡C^*t*^Bu] in C–H bond activation.

In previous work,^26^ we confirmed the proposed mechanism using ωB97XD, a density functional which contains range-separated exact exchange and dispersion corrections.^27^ Our study also included the comparison of several DFT approaches, which showed that dispersion was critical for accurate modeling of this reaction, particularly for the stability of **A**. This was not surprising because modeling noncovalent interactions, which could play a major role in the reaction mechanisms, requires dispersion corrections.^28−33^ In addition to the methodology assessment, we found a new conformer that is both more stable and kinetically more reactive,^26^ improving the accuracy of the model mechanism and its agreement with the experiment.

Mindiola and co-workers also proposed the possible tautomerization and formation of the short-lived terminal methylidene titanium complexes (PNP)Ti=CH_2_(CH_2_^*t*^Bu). Their isotopic labeling studies have shown an exchange between the alkylidene and methyl hydrogens of (PNP)Ti=CH^*t*^Bu(CH_3_). One of the possible exchange mechanisms, the reversible formation of a terminal methylidene, opens a potential secondary reaction to the methane activation process. This is important as terminal methylidene complexes are highly valued as precursors for the synthesis of alkenes.^34−37^ Computational studies show that the tautomerization pathway is higher in free energy than the alternative reaction (H-abstraction/return to methane). The kinetic preference toward H-abstraction is small as the difference between both barriers is 0.7 kcal/mol in our study^26^ and around 3 kcal/mol in Mindiola’s study.^24^

In another previous study,^38^ we explored the effect of modifying the ligands in the (PNP)Ti=CH^*t*^Bu(CH_2_^*t*^Bu) complex both on the activation barriers for the methane activation and in the preference for the tautomerization process. Both ligands (PNP and CH^*t*^Bu) were systematically modified. In general, the modifications which changed electronic properties had small and inconsistent effects, while the use of bulky ligands favored the methane activation process. One of the most significant changes was modifying the PNP ligand by replacing the ^*i*^Pr groups of the phosphine with ^*t*^Bu. Thus, the steric effects that increased crowding around the titanium lowered both C–H activation barriers. Further acceleration of the C–H activation occurred when the ^*t*^Bu phosphine was combined with an extra CH_2_ linker in the PNP ligand, pushing the bulky substituents toward the reaction center. On the other hand, substituting nitrogen in the PNP with phosphorus lowered both activation barriers, reversed the kinetic preference for H-abstraction, and stabilized the terminal methylidene, such that it was only 2 kcal/mol less stable than the product of methane activation. Because most of the ligand modifications did not change the barriers or the mechanism significantly, we emphasized the resilience of the complex toward electronic changes, allowing flexibility in the synthesis of modified complexes to improve properties such as solubility, synthetic cost, stability, or even support on a heterogeneous material.

In the present study, several early first-row transition-metal complexes have been studied in the hope of discovering a more efficient complex for methane C–H activation. Since the initial product of these C–H activations can undergo tautomerization by forming the terminal methylidene complex, which could prevail or be in the mixture with the initial C–H activation product, this tautomerization reaction was also studied. Complexes were grouped in two families: (1) the same total charge and thus the same formal oxidation number (IV) for the metal (isocharged) or (2) the same electronic configuration at the metal (isoelectronic). In a few examples, metal modification has been combined with modifications on the PNP ligand which were shown to be promising in titanium complexes^38^ to further optimize the complex for C–H activation.

## 2 Methodology

All calculations were performed using the ωB97XD^27^ density functional and included solvent effects via the SMD solvation model.^39^ Geometries were optimized using the def2SVP basis set, and the energies were refined with single-point calculations using the def2TZVP basis set.^40,41^ Gibbs free energies were estimated using the vibrational results from the smaller basis set calculations (G_def2TZVP_ = G_def2SVP_ – E_def2SVP_ + E_def2TZVP_) to reduce the computational cost. All the energies listed below are Gdef2TZVP, calculated at 298.15 K and 1 atm. A dense integration grid with 99 radial shells and 590 angular points (ultrafine grid) was used. DFT calculations were performed using the Gaussian 09 (revision D.01)^42^ software package. Bader charges^43^ were calculated with Multiwfn^44^ from Gaussian 09 checkpoint files (wB97XD/def2TZVP level) with a separation of 0.06 Bohr in the integration grid.

It should be noted that due to the high flexibility of the complex, several different conformers were found for most species. These isomers had the same structure but slightly different conformation of the ligands and differed in Gibbs free energy by around 2 kcal/mol. For the sake of simplicity, only the most stable conformers will be reported.

## 3 Results and Discussion

**Figure 2:**
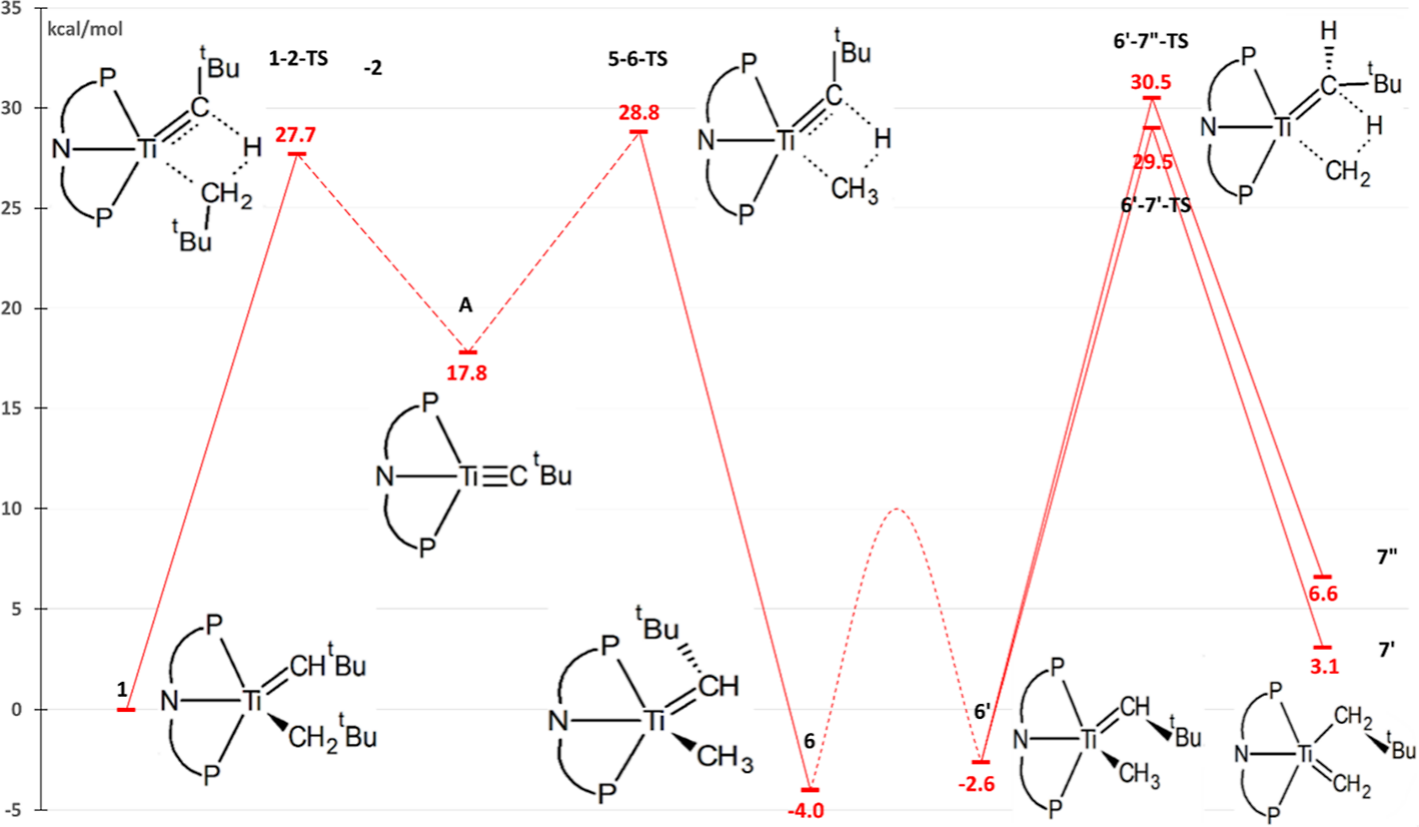
Simplified free-energy profile of methane C–H activation (from **1** to the most stable of **6** or **6′**) and tautomerization reactions (from **6** or **6′** to **7′** or **7″**). “H-abstraction” is the reverse reaction from **6** (or **6′**) to **A**.^24^ All values (kcal/mol) calculated with ωB97XD as per the Methodology section. The original numbering scheme^26^ is used for the structures, and the complete mechanism is given in Figure S1; ’ and ’’ represent alternative closely related isomers.

The energies of species involved in C–H bond activation and tautomerization reactions for complexes of several metals were calculated following the energy profile found in our previous work. As previously reported,^26,38^ some steps and intermediates in the full mechanism are not essential in the energetics because their energy is either too high to be competitive (tautomerization from **6**) or too low to affect the overall rate (isomerization of **6** to **6′** and release of weakly sigma-bonded ligands in **2** and **5**). The complete mechanism is given in Figure S1, with additional species that explain the labels used for the chemical species. Based on these observations, a simplified reaction pathway is studied and reported for the modified complexes in the present study (Figure 2). However, several of the excluded steps were calculated for modified systems, confirming the trends observed in titanium complexes. The profile in Figure 2 contains several related processes, and for our analysis below, we mainly divided it into two processes labeled as “methane activation” (from **1** to the most stable of **6** or **6′**) and “tautomerization” (from **6** or **6′** to **7′** or **7″**). In addition, we used the term “H-abstraction” to describe the process competing with the tautomerization, the reverse reaction from **6** (or **6′**) to **A**.

We calculated energy profiles for scandium, titanium, vanadium, and chromium complexes, both isoelectronic (with the same number of electrons in the metal) and isocharged (with the same charge and oxidation state of the metal) complexes.

### 3.1 Isoelectronic Complexes

Three [(PNP)M=CH^*t*^Bu(CH_2_^*t*^Bu)]^*n*^ complexes were studied, with M = Sc, V, Cr, where “*n*” is the appropriate charge that corresponds to the electron configuration of the original Ti complex. In the original PNP complex, titanium is present as a Ti^4+^ cation and has no valence electrons. Thus, in these isoelectronic complexes, the central metal ion has a noble-gas electronic configuration. Alternatively, using the covalent or neutral model for electron counting, there are four valence electrons in the central M^*n*^ ion. All the electrons on the valence shell around the metal are involved in the ligand–metal bonds, and there are no nonbonding d electrons in the metal. As all their valence electrons are paired, these are closed-shell singlet species.

**Figure 3:**
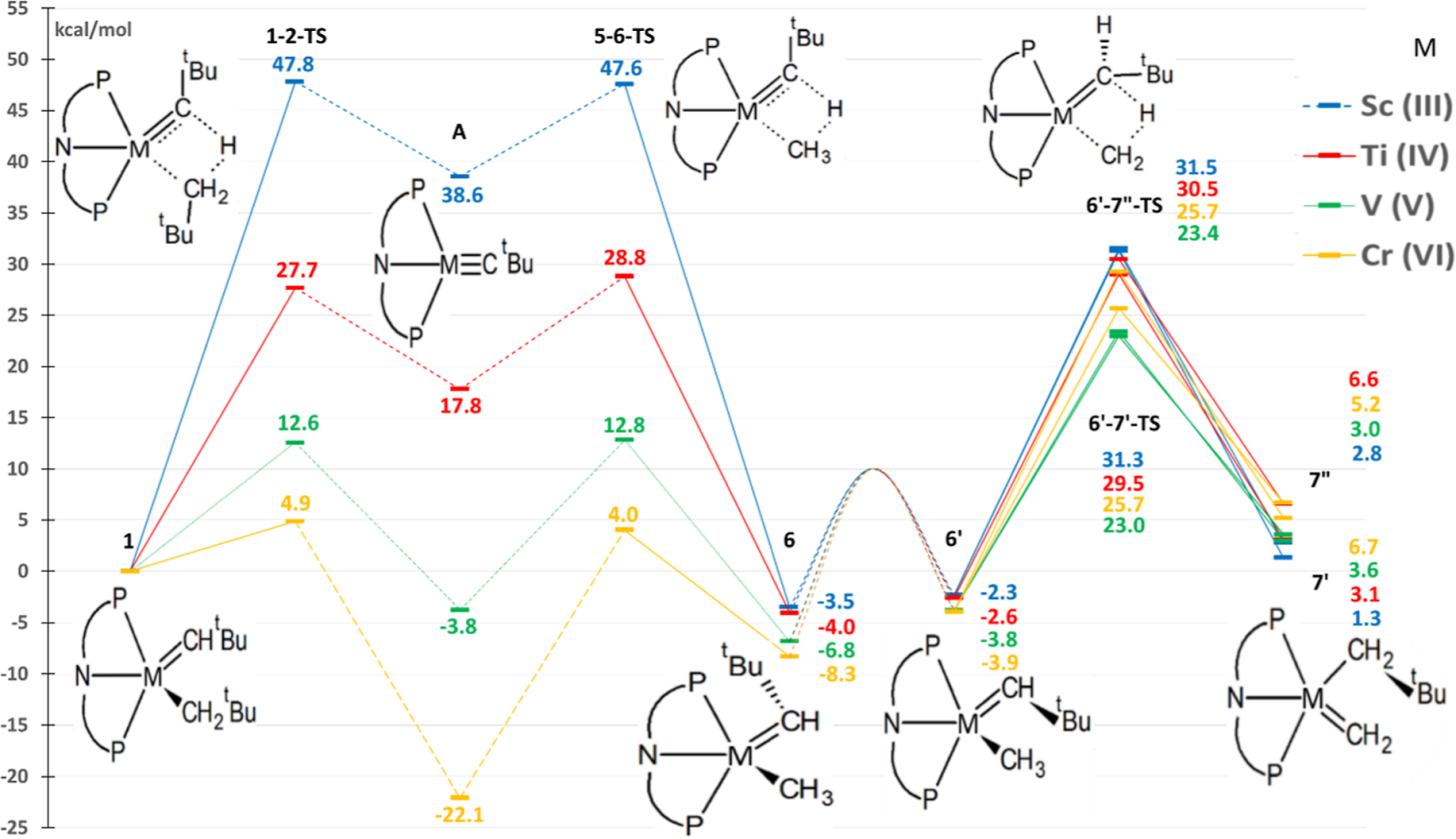
Simplified free-energy profile of the methane C–H activation and tautomerization reactions for different [(PNP)M=CH^*t*^Bu(CH_2_^*t*^Bu)]^*n*^ complexes isoelectronic to (PNP)Ti=CH^*t*^Bu(CH_2_^*t*^Bu). Data for Ti^(IV)^ are from our previous work.^26^ Calculated values for the C–H activation barrier, H-abstraction barrier, and tautomerization barrier are in Table S1.

Results of the methane activation process (from **1** to **6** or **6′**) show that modifying the metal along the Sc–Ti–V–Cr series leads to significant differences, while modifying the metal has a moderate effect in the tautomerization reaction to form **7** (Figure 3). All the involved species in the methane activation (transition states, products **6** and **6′** and intermediate **A**) have increased stability with increased metal atomic number (i.e., from Sc to Cr). The largest difference is found in the stability of intermediate **A** (with a range of 60.7 kcal/mol).

For the Sc^(III)^ complex, the barrier of the C–H activation process, corresponding to **1–2 TS**, is 47.8 kcal/mol, which is the highest among studied complexes (Figure 3). Thus, Sc^(III)^ is not expected to be a good alternative for methane activation since both the products and the barriers are much less stable than those for the Ti^(IV)^ compound, where C–H bond activation was experimentally observed.^24^ On the other hand, V^(V)^ has very promising barriers for C–H activation, with a rate-determining barrier (**5–6 TS**) of 16.6 kcal/mol, a value more than 10 kcal/mol lower than that for Ti^(IV)^. Intermediate **A** is more stable than reactant **1** in the V^(V)^ complex (by 3.8 kcal/mol), but the C–H activation of methane (from **A** to **6**) is still an exoergic process (−3.0 kcal/mol).

For the Cr^(VI)^ complex, the energies of the transition states are the lowest among the complexes considered in Figure 3. However, **A** is also very stable, which increases the rate-determining barrier from **A** to **6**, to 26.1 kcal/mol, a value which now exceeds the barriers for Ti^(IV)^. As a consequence, the activation of methane is no longer thermodynamically favorable; the C–H bond activation product **6** is 14 kcal/mol less stable than intermediate **A**, and the reaction is expected to be trapped by the formation of **A**. This renders the complex unusable for C–H bond activation.

Considering the tautomerization process, as was mentioned above, modifying the metal along the Sc–Ti–V–Cr series does not affect the energies of the tautomerization significantly; the relative barriers (from the most stable **6** or **6′** to **7′** or **7″**, Table S1) only span a small range from 29.8 kcal/mol in V^(V)^ to 34.8 kcal/mol in Sc^(III)^. One can notice that there is an outlier in the trend as the reaction barriers are stabilized from Sc^(III)^ to V^(V)^, but the energy barrier is higher for Cr^(VI)^ than V^(V)^.

Conversely, the kinetic preference for the H-abstraction process (the reverse of methane activation from **6** or **6**′ to **A**, Table S1) is highly affected by the metal. The low barrier of H-abstraction for V^(V)^ (19.6 kcal/mol) and Cr^(VI)^ (12.3 kcal/mol) will cause H-abstraction to be kinetically favored over the tautomerization process (with barriers of 29.8 and 34.0 kcal/mol, respectively, Table S1). Although the Sc^(III)^ complex’s large H-abstraction barrier (51.1 kcal/mol) should prevent the backward reaction from **6**, its large methane activation barrier precludes the formation of **6** with Sc^(III)^.

These isoelectronic complexes have charges that range from −1 to +2 (Figure 3), paralleling the oxidation state of the metal. Bader partial charges show that the charge transfer from the ligands to the metal center for complexes **1**, **A**, and **6** (Table S2) increases with the increasing oxidation state of the metal (from Sc to Cr), as expected. Also as expected, the charge transfer changes are similar for the three species (**1**, **A**, and **6**) (more details about the partial charges are available in the Supporting Information as well as the analysis of the geometries of studied complexes).

In order to understand the origin of the differences among the complexes, we have studied the strength of the key metal–carbon bonds, since the effect of the metal is most notable in the energy of **A** relative to all other intermediates and products. In Table 1, the M–C, M=C, and M≡C bond energies in **1** and **A** are presented as a difference in the electronic energy of the complexes and separated, neutral radical fragments (without geometry reorganization). In the case of complex **1**, with M–C and M=C bonds, the energies of the bonds have been calculated both separately (MR_1_R_2_ compared with MR_1_ + R_2_ and MR_2_ + R_1_) and combined (MR_1_R_2_ compared with M + R_1_ + R_2_).

**Table 1:** Metal–Ligand Bond Energies, Calculated as the Difference in Electronic Energy between the Complex and the Separated Neutral Radical Fragments without Geometry Reorganization

metal	M–C	M=C	C=M–C	M≡C
Sc^(III)^	86.9	129.8	214.2	162.5
Ti^(IV)^	78.0	107.1	181.8	150.6
V^(V)^	46.9	87.8	128.5	116.7
Cr^(VI)^	37.3	70.1	91.1	101.0

Generally, the bond energies decrease with increasing atomic numbers, suggesting that the covalent interactions get weaker from Sc^(III)^ to Cr^(VI)^ (Table 1). However, the bond energy of the M≡C triple bond in **A** decreases less rapidly, which results in the trend toward overstabilization of **A** (one triple bond) relative to **1** and the other species (with one single and one double bond). The difference in the bond energies between **1** (both bonds) and **A** decreases from Sc (51.7 kcal/mol) to Cr (−9.9 kcal/mol) (Table 1), a difference which corresponds closely to the relative stability of **A** (Figure 3). From **1** to **A**, one σ bond is replaced by one π bond, which is less weakened by the metal modification. The data in Figure 3 show that barriers involved in C–H bond activation are stabilized from Sc to Cr, a stabilization which parallels the weakening of the metal–carbon bonds from Sc to Cr since weaker bonds are easier to break or transform.

### 3.2 Isocharged Complexes

**Figure 4:**
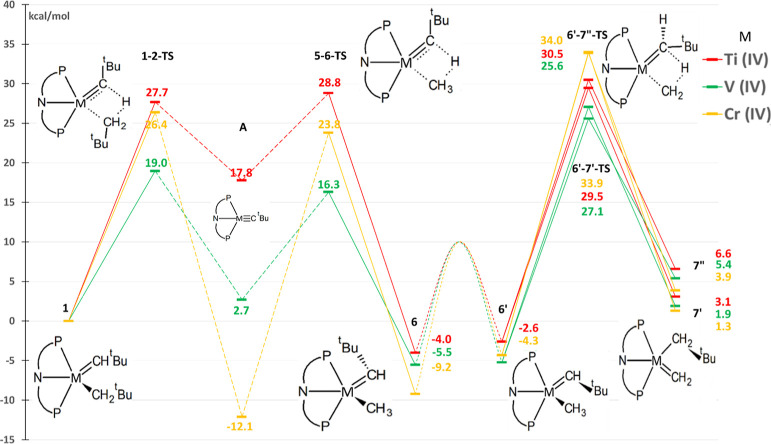
Simplified free-energy profile of the methane C–H activation and tautomerization reactions for different isocharged neutral (PNP)M=CH^*t*^Bu(CH_2_^*t*^Bu) complexes. Data for Ti^(IV)^ are from our previous work.^26^ Calculated values for the C–H activation barrier, H-abstraction barrier, and tautomerization barrier are in Table S4.

Neutral complexes (PNP)M=CH^*t*^Bu(CH_2_^*t*^Bu) were tested with vanadium and chromium and compared with previous results of the Ti^(IV)^ complex^26^ (Figure 4). As these complexes are neutral, all the metals have a formal oxidation state of IV. Scandium was not included because it has only three valence electrons and cannot form Sc^(IV)^ complexes. In V^(IV)^ and Cr^(IV)^ complexes, the additional electrons populate the 3d orbitals of the metal by 1 and 2 electrons, respectively. Thus, the V^(IV)^complex has one unpaired electron and all species were calculated doublets. The two electrons of the Cr^(IV)^ species are also unpaired, and all species were calculated as triplets since the low-spin, singlet species are around 25 kcal/mol less stable.

Despite the difference in the quantitative results compared to the isoelectronic metals (Figure 3), qualitative results are somewhat similar (Figure 4). Again, the vanadium complex, in this case, the V^(IV)^ complex, has the lowest barriers for both processes: methane activation (19.0 kcal/mol) and tautomerization (31.1 kcal/mol, Table S4). The C–H activation barrier is significantly lower than in the original Ti^(IV)^ complex (by almost 10 kcal/mol) but slightly higher than in V^(V)^ (by around 2 kcal/mol). Again, H-abstraction is kinetically preferred to tautomerization (by 9.3 kcal/mol, Table S4). On the other hand, the high stability of **A** with Cr^(IV)^ makes it the most stable species, but in this case, the energy difference between **6** and **A** is around 3 kcal/mol. Thus, this should not prevent the activation of methane because an equilibrium could form between these two species. However, the barrier for C–H activation in Cr^(IV)^ is high (35.9 kcal/mol) due both to the high stability of **A** and to the moderate stabilization of the transition state (which is less stable in Cr^(IV)^ than in V^(IV)^). Also, the tautomerization barrier is very high (43.1 kcal/mol, Table S4), with a destabilization of the transition states compared with Ti^(IV)^. Altogether, Cr^(IV)^ is not a very promising candidate.

Turning to the strength of the metal–carbon bonds in isocharged complexes (Table 2), the results show that the trends for metals are similar to those for isoelectronic complexes (Table 1); however, the magnitude of the decrease is smaller. The bond energies decrease with increasing atomic number, suggesting that the covalent interaction gets weaker as the metal adds electrons. Since the single M–C bond energy does not change significantly between V and Cr, the major differences arise from differences in the π bonding. As in the isoelectronic complexes, the triple bond interaction energy in **A** decreases less than the combination of the single and double bond in **1**, leading to the overstabilization of **A** for Cr^(IV)^. The bond energy of the M≡C triple bond decreases by 14% from Ti to Cr, while the individual bonds in **1** decrease by 18 and 35% (for the single and double bond, respectively). This is consistent with the reduction of the ligand-to-metal charge transfer in these complexes (Table S2).

**Table 2:** Metal–Ligand Bond Energies, Calculated as the Difference in Electronic Energy between the Complex and the Separated Neutral Radical Fragments without Geometry Reorganization

metal	M–C	M=C	C=M–C	M≡C
Ti^(IV)^	78.0	107.1	181.8	150.6
V^(IV)^	63.3	89.7	159.1	139.5
Cr^(IV)^	64.2	69.8	139.2	129.8

Neutral V and Cr complexes show higher barriers (Figure 4 and Table S4) than their cationic equivalent (Figure 3); this can be partially due to the fact that the M–C bonds for the neutral complexes are stronger. Additionally, it must be considered that the presence of d electrons reduces the number of free orbitals in the metal, changing the simultaneous partial interactions with the forming/breaking bonds. This effect could explain why the Cr^(IV)^ barriers are higher than those for V^(IV)^ and Ti^(IV)^, despite having weaker M–C bonds.

General trends in decreasing bond energies from Ti^(IV)^ to Cr^(IV)^ (Table 2), as well as the fact that this decrease is smaller than in the case of isoelectronic complexes (Table 1), is a consequence of hard–soft properties of metal ions. Although hardness properties increase from Ti^(IV)^ to Cr^(IV)^, weakening the bonds with the soft carbon, the increase in hardness is larger from Sc^(III)^ to Cr^(VI)^, causing a larger change in bond energies (Table 1). The difference in bond energies between two oxidation states of the same metal can also be attributed to hard–soft properties, with metal in a higher oxidation state being harder and producing weaker metal–carbon bonds.

### 3.3 Modified Ligands

**Figure 5:**
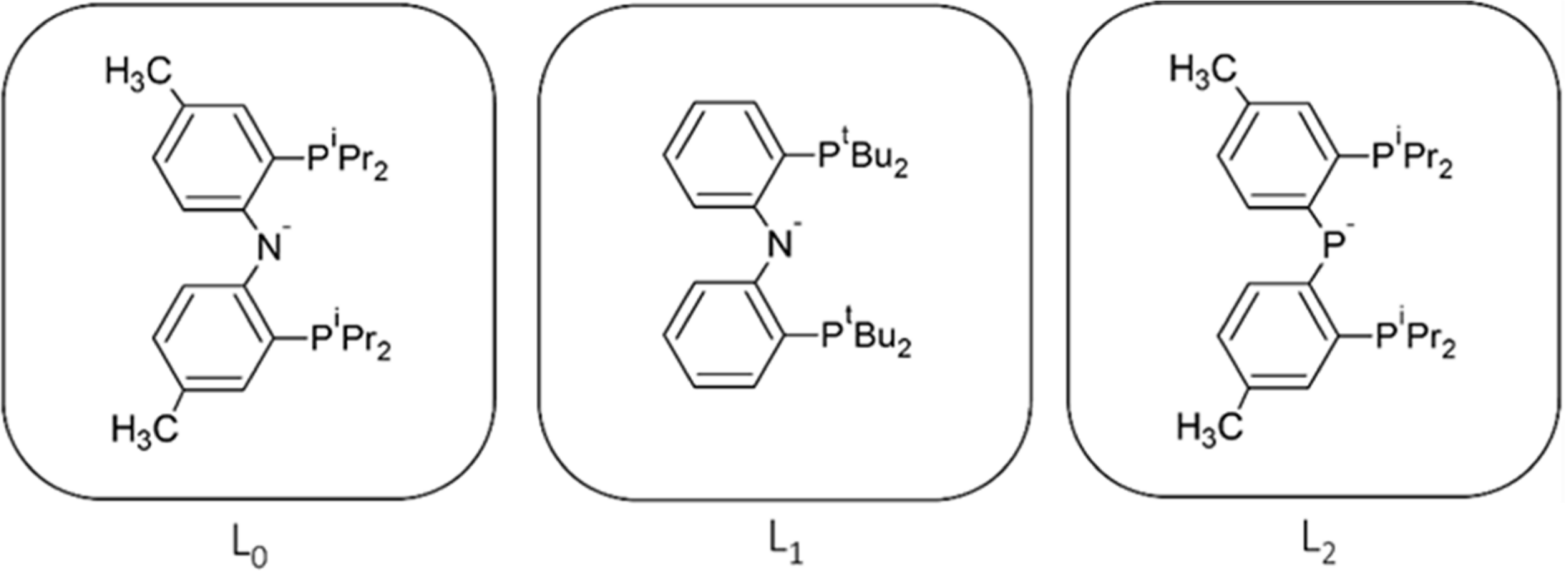
Unmodified PNP ligand (L_0_) and modified versions of the ligand (L_1_ and L_2_).

**Figure 6:**
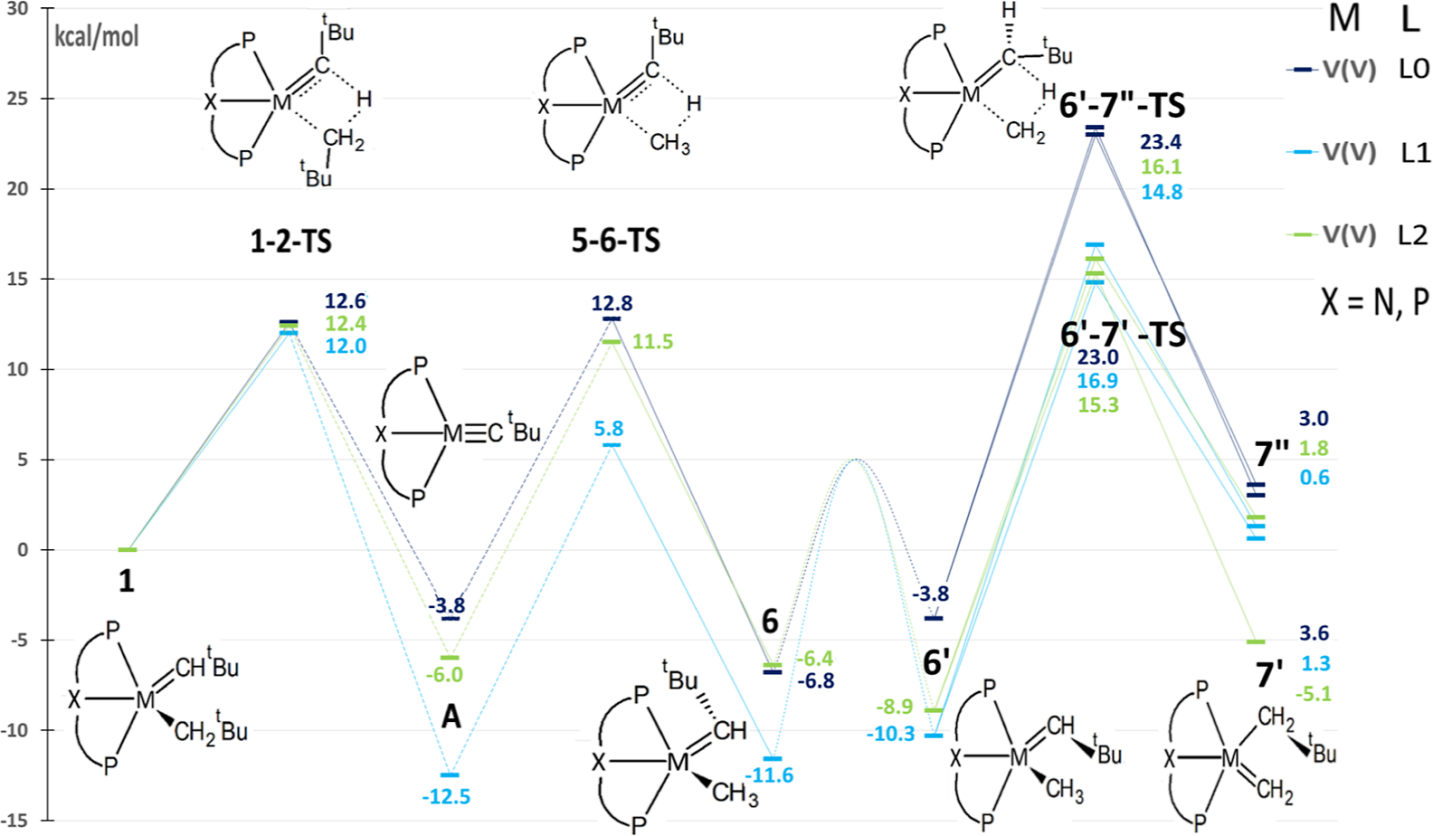
Simplified free-energy profile of the methane C–H activation and tautomerization reactions for [(L)M=CH^*t*^Bu(CH_2_^*t*^Bu)]^*n*^ complexes, where M is V^(V)^, while L refers to modified ligands as depicted in Figure 5. Calculated values for the C–H activation barrier, H-abstraction barrier, and tautomerization barrier are in Table S6.

In our previous work,^38^ we showed the influence of ligand modification for the Ti complex, while data in this work show that the ligand modifications have a similar influence for complexes of all studied metals. Since modifications of the ligand do not have large influences on the reaction energy profile,^26^ ligand modifications could not improve Sc and Cr complexes enough to make them suitable for C–H activation. Thus, we present results only for V complexes here (Figures 6 and S8), while the results for the Sc and Cr are given in the Supporting Information (Table S5). According to our previous study with Ti^(IV)^, the addition of bulky groups to the PNP ligand decreases the barriers and facilitates methane activation.^37^ In accordance with that, one of the most promising modified ligands, L_1_ (Figure 5) has been included here. The modified ligand L_1_ differs from the original ligand in the substitution of the ^*i*^Pr groups of the phosphine by ^*t*^Bu groups and in the removal of the methyl group at the aromatic ring (Figure 5). In our previous work, it was shown that the addition of a ^*t*^Bu group in a Ti^(IV)^ complex decreased the barriers between 2 and 5 kcal/mol, while the removal of the Me has little effect on energetics.^38^ Consequently, a combination of these two modifications has the same effects as only addition of the ^*t*^Bu bulky group (with differences below 1 kcal/mol in the energies).

The L_1_ complexes stabilize all the intermediates and transition states relative to **1**, but the magnitude of the stabilization is not the same (Figures 6 and S8). For example, the alkylidyne complex **A** is significantly stabilized in complexes, V^(V)^, and Ti^(IV)^ (around 9–10 kcal/mol),^29^ while in V^(IV)^, complex **A** is stabilized by only around 3 kcal/mol (Figure S8). This trend is also observed to a lesser extent in the stabilization of the methyl products (**6** and **6′**). For V^(V)^, stabilization of **6** and **6′** is around 5–6 kcal/mol, while for V^(IV)^, it is about 1 kcal/mol. Because complex **1** begins with large neo-pentyl ligands, **1–2 TS** are more weakly stabilized by the bulky ligand than the rest of the species in the reaction pathway, especially compared with intermediate **A**. The larger effect of the ^*t*^Bu substituent in the cationic V^(V)^ complexes can be explained because they are significantly smaller; thus, the steric hindrance is more substantial.

Regarding the kinetics, the initial barrier, **1–2 TS**, is slightly lower in complexes with ligand L_1_. For Ti^(IV)^, it was reduced by 5.1 kcal/mol from 27.7 kcal/mol with L_0_ to 22.6 kcal/mol with L_1_,^38^ while it is reduced by only 0.6 kcal/mol for V^(V)^ and 0.2 kcal/mol for V^(IV)^ (Figures 6 and S8). There is even an increase in the barrier for C–H activation in the cationic complexes due to the large stabilization of **A** (Figure 6 and Table S6). On the other hand, the tautomerization barrier (from the most stable **6** or **6′** to **7′** or **7″**, Table S6) decreases moderately with the cationic V^(V)^ complex (3.4 kcal/mol). For V^(IV)^, the barriers for tautomerization even increase slightly by 1.7 kcal/mol due to the largest size of the complex and the conformational differences directed by the presence of nonbonding electrons.

Our previous study on Ti complexes showed that the exchange of the nitrogen atom of the PNP ligand with a phosphorus (L_2_, Figure 5) led to a partial stabilization of the tautomerization product, a decrease of the tautomerization barrier, and a kinetic preference for tautomerization versus H-abstraction.^38^ Hence, this ligand was the most promising modification to optimize the complex for its reactivity toward the formation of the terminal methylidene. We have applied this change (ligand L_2_) to both complexes with the most promising metal, vanadium (Figure 6). Like the Ti complex, the products of tautomerization (**7′** and **7″**) were stabilized, but only the V^(V)^ complex produced a significant stabilization (**7′** was stabilized by 8.7 kcal/mol). Even in this case, the tautomerization product was still not more stable than the methyl complex **6’** (−8.9 kcal/mol vs −5.1 kcal/mol), but the difference was significantly reduced and an equilibrium could occur. Regarding the kinetics, the presence of L_2_ stabilized the tautomerization transition states and the barrier (from the most stable **6** or **6′** to **7′** or **7″**, Table S6) decreased by 5.6 kcal/mol for V^(V)^ but increased by 1.4 for V^(IV)^ due to the stabilization of **6′**. The barrier for the reverse H-abstraction [from **6** (or **6′**) to **A**] increased slightly for V^(V)^ (from 19.6 to 20.4 kcal/mol), and the kinetic preference toward the H-abstraction (difference between tautomerization and H-abstraction barriers, Table S6) reduced from 10.2 to 3.8 kcal/mol. Hence, the P-substituted complex (L_2_ ligand, Figure 5) with V^(V)^ shows the best potential to form the final methylidene complex **7** since the desired end point can be obtained with a moderate barrier (24.2 kcal/mol, tautomerization barrier Table S6).

## 4 Conclusions

The (PNP)Ti=CH^*t*^Bu(CH_2_^*t*^Bu) complex’s activation of sp^2^ and sp^3^ C–H bonds through a transient titanium neopentylidene, under mild conditions, is a promising starting point in the development of technology for the conversion of natural gas to high-value chemicals. Our previous exploration of the effect of different ligand modifications on the complex shows that its reactions (methane activation and the following tautomerization) are surprisingly resilient toward the modification of the ligands.^38^ Here, the effect of changing the metal on the complex has been explored. In addition to Ti, three inexpensive first-row transition metals were studied (Sc, V, and Cr) using both the isoelectronic and isocharged complexes.

Our calculations predict that the most promising complexes are those formed with vanadium. Both the cationic V^(V)^ and the neutral V^(IV)^ complexes have relatively low barriers for all the processes under study. Methane activation has a rate-determining barrier of 16.6 and 19.0 kcal/mol, for V^(V)^ and V^(IV)^, respectively. This decreases the barrier by around 12 kcal/mol compared with the parent Ti^(IV)^ complex and even 4 kcal/mol lower than the best ligand/Ti^(IV)^ combination obtained in previous work.^38^ The tautomerization barrier is moderate (29.8 and 31.1 kcal/mol), although it is higher than the barrier for the reverse process of H-abstraction.

On the other hand, the results of the other two metals studied indicate that they cannot activate the C–H bond under mild conditions. In the case of chromium, the dramatic stabilization of the alkylidene intermediate **A** renders it as the most stable species, and thus, this is the expected terminal product. For Sc^(III)^, the barrier of the activation reaction is very high (47.8 kcal/mol for methane activation), making it a poor candidate.

Additionally, a few of the ligand modifications previously explored with Ti^(IV)^ were applied to the complexes of other metals. The studies suggest that the ligand effects studied in the parent Ti^(IV)^ complex are partially transferable to other metal complexes. Increasing the steric hindrance by replacing the ^*i*^Pr groups with ^*t*^Bu (L_1_) reduces the C–H activation barriers for most of the tested complexes. However, the use of L_1_ does not decrease the barrier for our best candidate complex (V^(V)^). On the other hand, the substitution of the N atom in the PNP by a P atom (L_2_) enhances the reactivity of V^(V)^ toward the formation of final methylidenes, as found in Ti^(IV)^. The tautomerization barrier is reduced from 29.8 kcal/mol (V^(V)^ with L_0_) to 24.2 kcal/mol. This is the lowest tautomerization barrier in the complexes explored both here and in previous work^38^ (5 kcal/mol lower than in Ti^(IV)^), while this complex (V^(V)^ with L_2_) has also a quite low methane activation barrier (17.5 kcal/mol), calculated to be 3 kcal/mol lower than that of the best Ti^(IV)^ complex.^38^

The V^(V)^ complex with L_0_ is the most suitable for C–H activation, while the V^(V)^ complex with the L_2_ ligand is the most suitable to obtain the methylidene product after tautomerization. These two target complexes would be worthy of experiential investigation.

## Supporting Information Available

Additional tables for the energy of the barriers, Bader charge analysis, metal–ligand-optimized distances, results for modified ligands with Sc and Cr, and coordinates of the ground- and transition-state analysis (PDF)
