# Supplementary material for: Improving a Methane C–H Activation Complex by Metal and Ligand Alterations from Computational Results
Source: Inorg Chem. 2023 Mar 22;62(13):5058–66. doi: 10.1021/acs.inorgchem.2c03342 (PMC10848199; doi:10.1021/acs.inorgchem.2c03342)
Supplement: Supplementary file 1 — ic2c03342_si_001.pdf [file ic2c03342_si_001.pdf]

Improving a Methane C-H Activation Complex by Metal and Ligand Alterations from Computational Results

Dragan B. Ninković†, Salvador Moncho†, Predrag Petrović†, Michael B. Hall§, Snežana D. Zarić*†,‡, and Edward N. Brothers*†*

† Department of Chemistry, Texas A&M University at Qatar, P.O. Box 23874, Doha, Qatar

‡ Department of Chemistry, University of Belgrade, Studentski trg 12-16, Belgrade, Serbia

§ Department of Chemistry, Texas A&M University College Station, TX 77843-3255, USA

*E-mail: hall@science.tamu.edu, szaric@chem.bg.ac.rs, ed.brothers@qatar.tamu.edu

Supporting Information

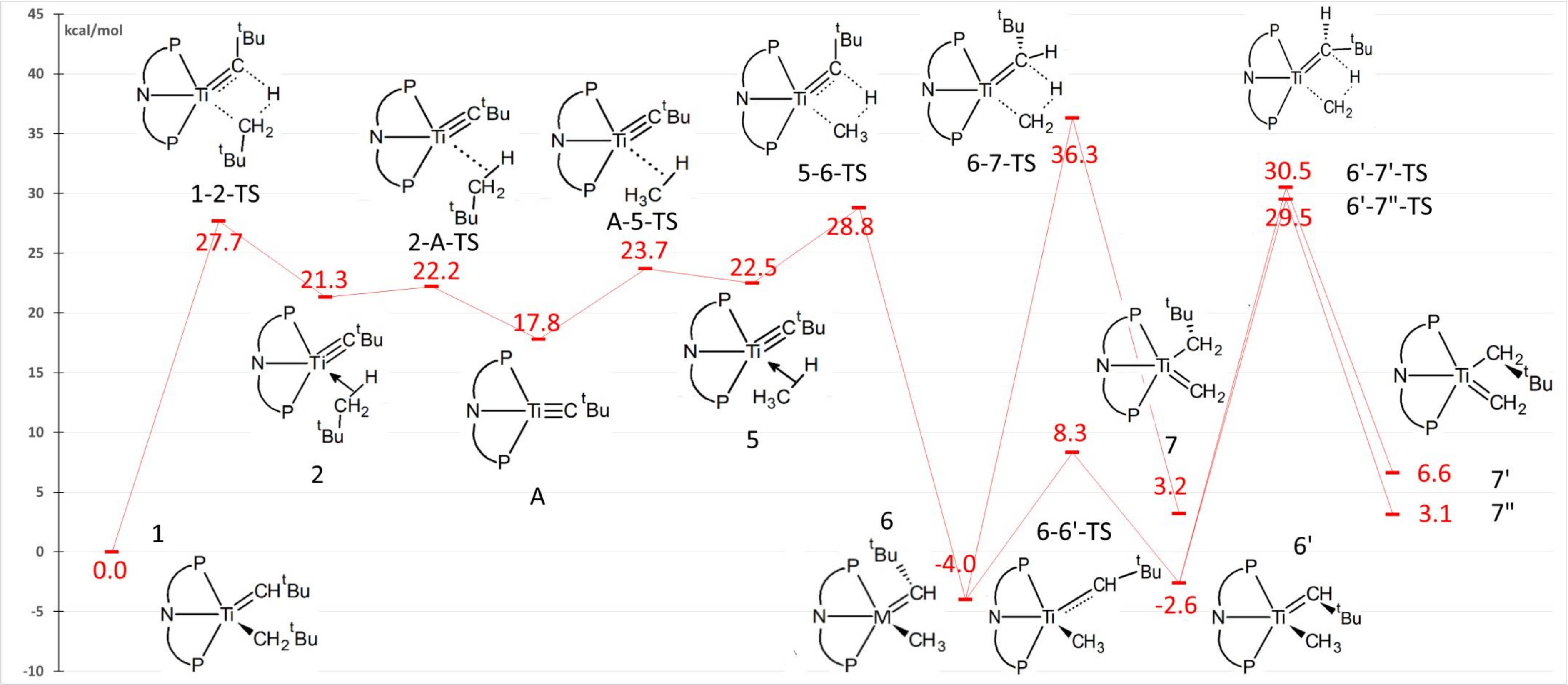

Figure S1. Free-energy profile of the methane C−H activation and plausible tautomerization pathway. All values (kcal/mol) calculated with ωB97XD. Labeling scheme is the same as in the Mindiola's and ours previous papers for consistency reasons. Geometries of the isomers 6

and 6′ are different in orientation of the tBu group to the doble bound. Geometries of the methylidene product isomers (7, 7′, and 7″) differ in conformational details; see geometries below.

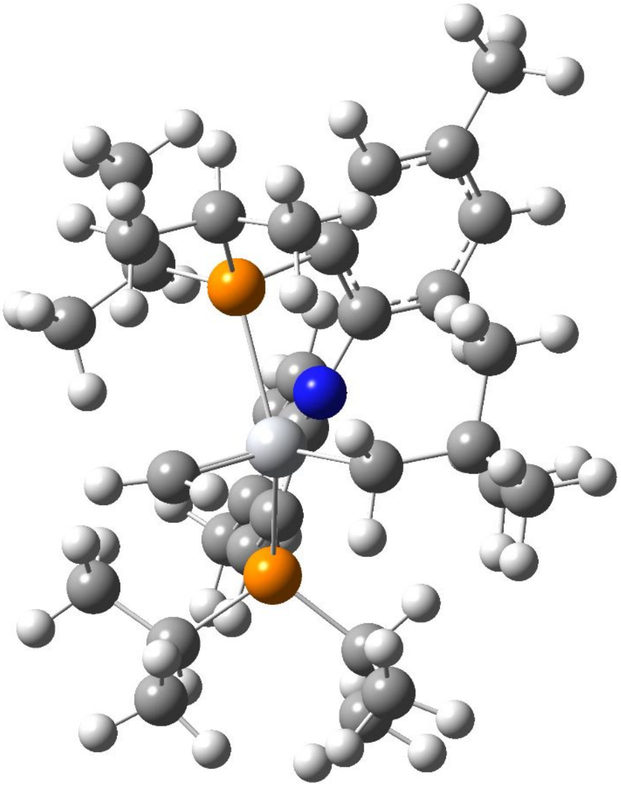

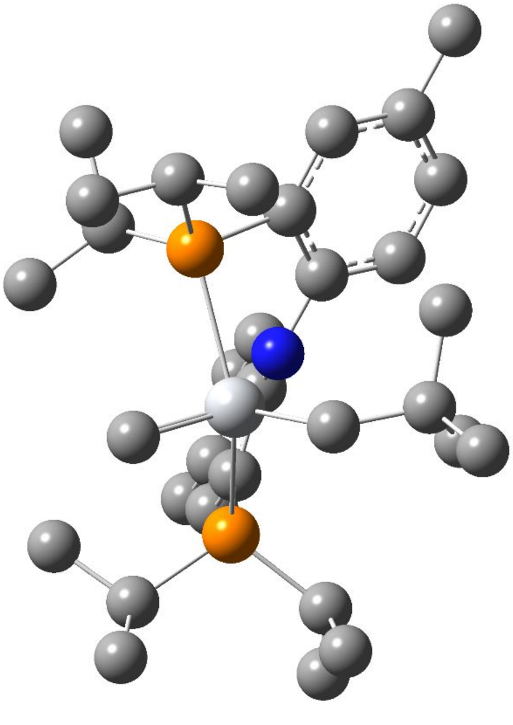

Figure S2. Geometry of the methylidene product isomer 7 with and without H atoms.

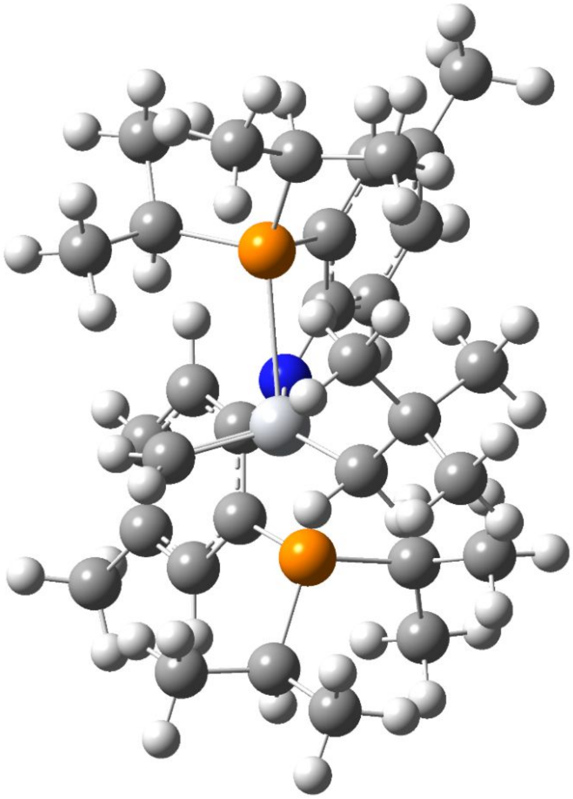

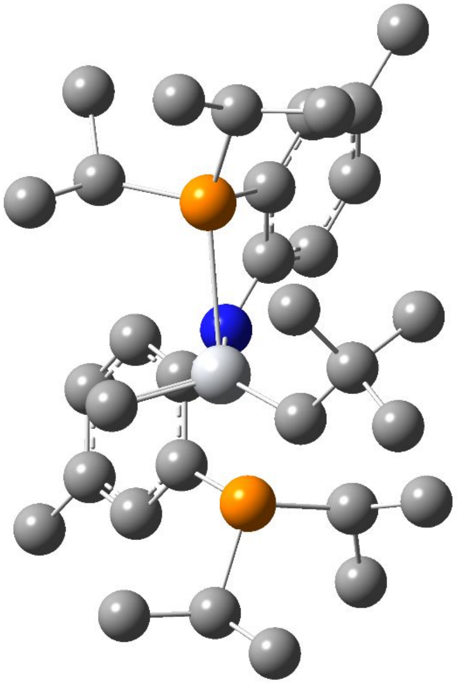

Figure S3. Geometry of the methylidene product isomer 7’ with and without H atoms.

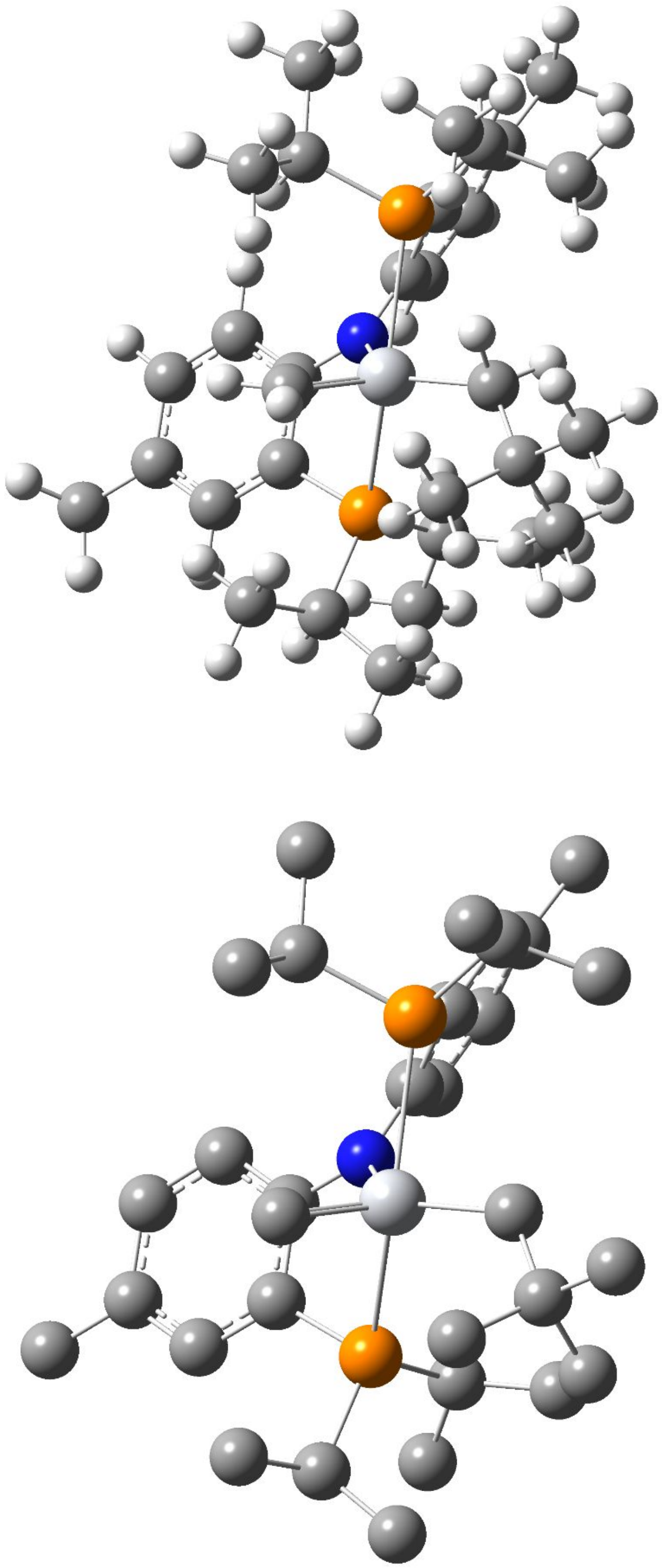

Figure S4. Geometry of the methylidene product isomer 7” with and without H atoms.

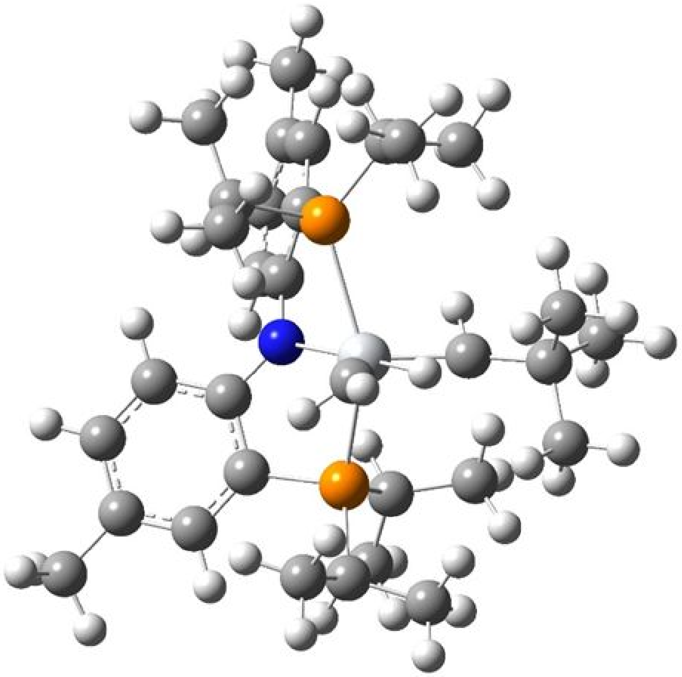

Figure S5. Geometry of the transition state 6’-7’-TS

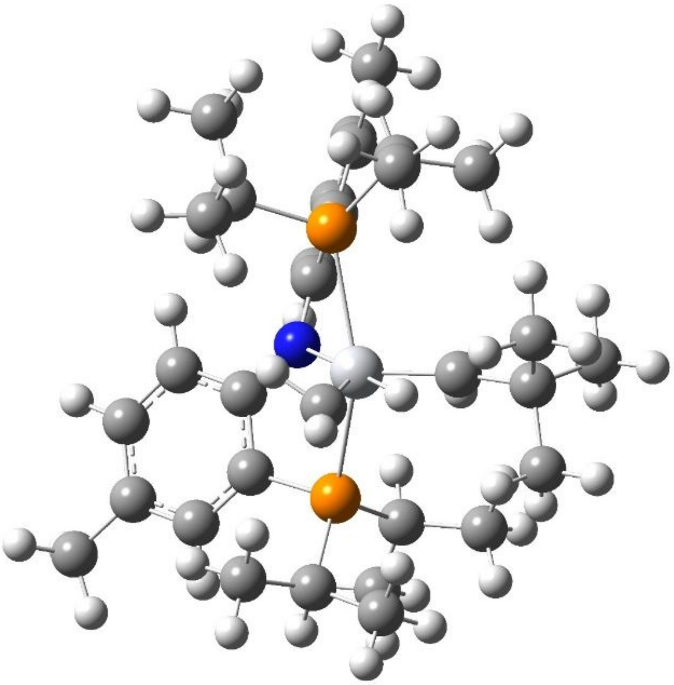

Figure S6. Geometry of the transition state 6’-7”-TS

Table S1. Calculated values for C-H activation barrier that is a higher value between two barriers (1-2-TS – 1 or 5-6-TS – A), H abstraction barrier from more stable product 6 or 6' (5-6-TS – MIN(6, 6')) and tautomerization barrier that is calculated as difference between the minimal transition state and minimal energy of the product (MIN(6'-7'-TS,6'-7"-TS) –MIN(6,6') for different [(PNP)M=CHtBu(CH2tBu)]n complexes isoelectronic to (PNP)Ti=CHtBu(CH2tBu).

**M**	C-H activation barrier	H-abstraction barrier	Tautomerization barrier
$Sc^{(III)}$	47.8	51.1	34.8
$Ti^{(IV)}$	27.7	32.8	33.5
$V^{(V)}$	16.6	19.6	29.8
$Cr^{(VI)}$	26.1	12.3	34.0

Analysis of the Bader Partial Charges and Analysis of the Geometries of Studied Complexes

The charge transfer from the ligands to the metal center was calculated as the difference between the total Bader charge on the ligand (Table S2) and the formal charge of the ligand as an ion. In all the explored complexes (**1**, **A** and **6**), the charge transfer from the ligands increases

with the charge of the metal from Sc to Cr, causing the metal positive charge to decrease when the charge of the full complex increase. For example, there is a large difference in the charge transfer both from the alkylidine of **A** (formal charge of -3) from 1.324e in the Sc complex to 2.955e in the Cr complex and from the PNP (formal charge of -1) from 0.023 in Sc to 1.716e in Cr. Generally, there is an increase in the ligand-to-metal charge transfer of 3.325e in **A** (from Sc to Cr). Complex **1** shows less ligand-to-metal donation (between 0.074 e and 0.209e less global donation), due to the fact that the ligand-to-metal donation of the alkylidine ligand is higher than the added donations of the alkylidene and alkyl ligands. However, the variation of the ligand-to-metal donation with the metal is very similar in **1**, compared to **A**, with a global effect of 3.413e from Sc to Cr (distributed by ligands as 0.703e, 1.182e and 1.529e coming from the neo-pentyl, neo-pentylidene and the PNP ligands, respectively). With a very similar bonding pattern, both the ligand-to-metal donations and their metal dependence are very similar in **6** compared with **1,** with a global metal effect of 2.107e, distributed as 0.438, 0.830e and 0.839e, in the methyl, neo-pentylidene and the PNP, respectively).

Table S2. Bader charges of the fragments (metal and ligands) of selected ground states for different [(PNP)M=CHtBu(CH2tBu)]n complexes, where M= Sc(III), Ti(IV), V(IV), V(V), Cr(IV), Cr(VI).

Complex	Fragment	$Sc^{(III)}$	$Ti^{(IV)}$	$V^{(V)}$	$Cr^{(VI)}$	$V^{(IV)}$	$Cr^{(IV)}$
1	-CH2^{t}Bu	-0.801	-0.626	-0.376	-0.098	-0.585	-0.565
	=CH^{t}Bu	-1.172	-0.690	-0.288	0.009	-0.533	-0.429
	PNP	-0.842	-0.507	0.001	0.687	-0.457	-0.428
	Metal	1.815	1.824	1.663	1.402	1.575	1.422
A	≡C^{t}Bu	-1.676	-1.013	-0.452	-0.045	-0.809	-0.705
	PNP	-0.977	-0.602	-0.069	0.716	-0.572	-0.503
	Metal	1.653	1.615	1.521	1.329	1.381	1.209
6	-CH3	-0.785	-0.630	-0.408	-0.238	-0.576	-0.593
	=CH^{t}Bu	-1.201	-0.716	-0.326	-0.023	-0.433	-0.555
	PNP	-0.831	-0.488	0.053	0.860	-0.410	-0.441
	Metal	1.817	1.834	1.680	1.401	1.420	1.589

The data on the geometries of the studied isoelectronic complexes shows that the geometry around the central metal atom changes from scandium to chromium. For example, in **A** the N-M-

C angle for the complex with Sc is 150˚, while for the other metals, this angle is between 95˚ and 106˚. Increasing the oxidative state of the metal affects the size of the complexes, with a significant reduction of the metal-ligand distances from Sc to Cr (the M-L distances for selected ground states are available in Table S3). Among the M-L bonds, the M-N bond lengths are the most affected. For example, in **1** the M-N distance goes from 2.327 Å in $Sc^{(III)}$to 1.832 Å in $Cr^{(VI)}$ (a difference of 0.495 Å). In the same complexes, the rest of the ligand-metal bonds change around 0.310 Å (two M-P and two M-C bonds). This effect is even larger for intermediate **A**, where the M-N distance differs by 0.700 Å, the M-P distances change around 0.321, and the M-C distance is reduced by 0.274 Å. As the largest effect is for the M-N bond, this suggests that the geometry is not only affected by the metal size but also by the electron deficiency in the metal, which slightly strengthens the M-N bond. This could be caused by an increase increases in the magnitude of the π donation of the lone pair of the N to the metal and slightly strengths the M-N bond. Nevertheless, the M-N bond should mainly be considered a single bond, as the lone pair mainly delocalized among the aromatic rings of the ligand; this can be observed in the fact that the M-N distances are more similar to the M-C distances than to the M=C distances (Table S3).

This is consistent with the fact that the ligand-to-metal donation changes with the metal are larger when using the PNP ligand (Table S2).

Regarding geometries of the isocharged complexes, metal centers are larger in neutral complexes than the corresponding ionic complexes (Table S3). M-L distances are consistently slightly smaller in the $V^{(IV)}$ complex than in the parent $Ti^{(IV)}$ complex, with differences between 0.02 and 0.12 Å (in **1**, **A** and **6**). In the $Cr^{(IV)}$ complex, there is a difference depending on the type of M-L bond; those bond with some ~~sp^2~~π bonding (Cr-carbene, Cr-carbyne and Cr-amido) get longer by less than 0.05 Å and the rest decrease between 0.07 and 0.18 Å. These differences are probably due to the occupation of the d orbitals in $Cr^{(IV)}$ ion, which interfere with the formation of π bonds.

There is also a large difference in the N-M-C angle for the $Cr^{(IV)}$ complex, versus that for the $Ti^{(IV)}$ and $V^{(V)}$ complexes where this angle is around 106°. The different electronic configurations of metal ions also affect the geometry. $Cr^{(IV)}$-**A** has a distorted square-planar geometry with the carbon trans to the N of the PNP (N-Cr-C angle is 165.9°). On the other hand, the $Cr^{(VI)}$ complex has a seesaw geometry, with a N-Cr-C angle of 95.7°, which is similar to the geometry found in the original Ti complex (with a N-Cr-C angle of 105.9°).

Table S3. Metal-ligand optimized distances for different intermediates in the reaction mechanism of the complexes [(PNP)M=CHtBu(CH2tBu)]n, where M=Sc(III), Ti(IV), V(IV), V(V), Cr(IV), Cr(VI).

		Metal					
**Intermediate**	**Atom(Ligand)**	**$Sc^{(III)}$**	**$Ti^{(IV)}$**	**$V^{(IV)}$**	**$V^{(V)}$**	**$Cr^{(IV)}$**	**$Cr^{(VI)}$**
**1**	N(PNP)	2.327	2.121	2.071	1.939	2.124	1.832
	P(PNP)	2.737	2.586	2.504	2.496	2.441	2.432
	P(PNP)	2.814	2.651	2.55	2.555	2.523	2.498
	C(CH2tBu)	2.348	2.184	2.165	2.079	2.113	2.039
	C(CHtBu)	1.994	1.811	1.765	1.725	1.857	1.68

**A**	N(PNP)	2.48	2.102	2.062	1.913	2.135	1.783
	P(PNP)	2.704	2.611	2.491	2.484	2.438	2.388
	P(PNP)	2.727	2.613	2.508	2.512	2.44	2.401
	C(CtBu)	1.887	1.722	1.689	1.644	1.766	1.613
**6**	N(PNP)	2.303	2.097	2.05	1.901	2.106	1.785
	P(PNP)	2.752	2.575	2.492	2.479	2.461	2.367
	P(PNP)	2.777	2.62	2.522	2.527	2.436	2.457
	C(CH3)	2.326	2.157	2.126	2.047	2.083	2.051
	C(CHtBu)	1.991	1.827	1.787	1.747	1.864	1.703

Table S4. Calculated values for C-H activation barrier that is a higher value between two barriers (1-2-TS – 1 or 5-6-TS – A), H abstraction barrier from more stable product 6 or 6' (5-6-TS – MIN(6, 6')) and tautomerization barrier that is calculated as difference between the minimal transition state and minimal energy of the product (MIN(6'-7'-TS,6'-7''-TS) –MIN(6,6') for different isocharged neutral (PNP)M=CH^{t}Bu(CH$_2$^{t}Bu) complexes.

**M**	C-H activation barrier	H-abstraction barrier	Tautomerization barrier
$Ti^{(IV)}$	27.8	32.8	33.5
$V^{(IV)}$	19.0	21.8	31.1
$Cr^{(IV)}$	35.9	33.0	43.1

Modified Ligands for the Sc and Cr

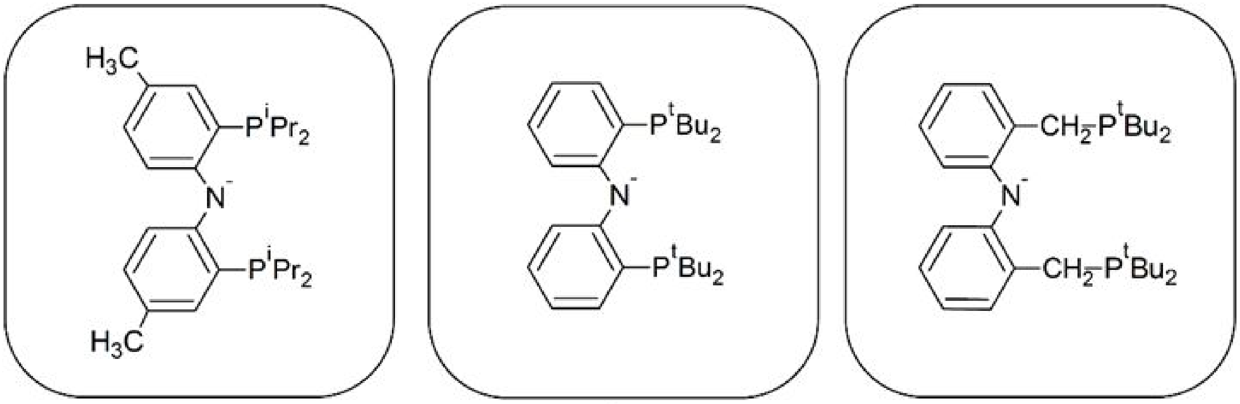

Figure S7. Unmodified PNP ligand (L_0) and modified versions of the ligand (L_1 and L_3)

Table S5. Free Gibbs energies (in kcal/mol) of selected intermediates and transition states in the methane activation process (Figure 2) for $[(L)M{=}CH^tBu(CH_2{}^tBu)]^n$ complexes, where L are modified ligands as depicted in Figure S1.

M	charge	L	1	1-2-TS	A	5-6-TS	6	6'	6'-7'-TS (6'-7"-TS)	7' (7")
$Sc^{(III)}$	-1	L_0	0.0	**47.8**	38.6	**47.6**	-3.5	-2.3	**31.3 (31.5)**	1.3 (2.8)
$Sc^{(III)}$	-1	L_1	0.0	**45.1**	35.6	**43.3**	-6.7	-6.8	**29.3 (26.6)**	-1.3 (0.4)
$Sc^{(III)}$	-1	L_3	0.0	**40.6**	30.5	**38.3**	-8.8	-6.1	**27.3 (25.8)**	-1.6 (0.0)
$Cr^{(VI)}$	+2	L_0	0.0	**4.9**	-22.1	**4.0**	-8.3	-3.9	**25.7 (29.3)**	6.7 (5.2)
$Cr^{(VI)}$	+2	L_1	0.0	**4.5**	-32.4	**-4.9**	-12.4	-11.5	**11.9 (14.0)**	-6.4 (-2.5)

We investigated the influence of the ligands, the conclusions from Ti complex more or less stand for all the metals. Hence the influence of the ligand were small they could not influence the

reactions strongly for the Sc and Cr complexes to make them viable catalyst for this reaction. Sc activation barriers are too large with both modified ligands. The C-H activation barriers are lower but the energy of 40.6 kcal/mol is still too high for this reaction to be viable. For the $Cr^{(VI)}$ the over stabilization of the intermediate A bring the reaction to stop since the barrier for the reaction is now 36.9 kcal/mol and the product is less stable then A for 20.0 kcal/mol.

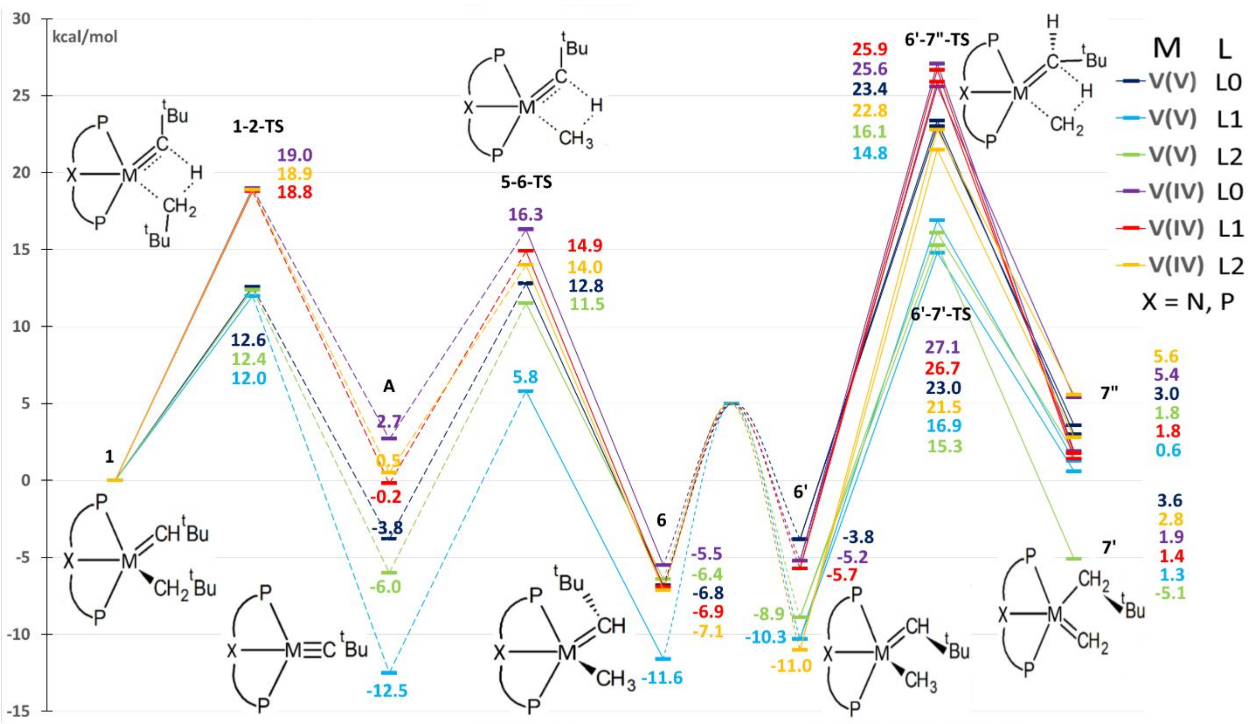

Figure S8. Simplified free energy profile of the methane C-H activation and tautomerization reactions for $[(L)M{=}CH^tBu(CH_2{}^tBu)]^n$ complexes, where M is $V^{(V)}$ and $V^{(IV)}$ while L are modified ligands as depicted in figure 5. Calculated values for C-H activation barrier, H abstraction barrier and tautomerization barrier are in Table S6.

Table S6. Calculated values for C-H activation barrier that is a higher value between two barriers (1-2-TS – 1 or 5-6-TS – A), H abstraction barrier from more stable product 6 or 6' (5-6-TS – MIN(6, 6')) and tautomerization barrier that is calculated as difference between the minimal transition state and minimal energy of the product (MIN(6'-7'-TS,6'-7''-TS) –MIN(6,6').

**M**	**charge**	**L**	C-H activation barrier	H-abstraction barrier	Tautomerization barrier
$V^{(V)}$	+1	L_0	16.6	19.6	29.8
$V^{(IV)}$	0	L_0	19.0	21.8	31.1
$V^{(V)}$	+1	L_1	18.3	17.4	26.4
$V^{(IV)}$	0	L_1	18.8	31.8	32.8
$V^{(V)}$	+1	L_2	17.5	20.4	24.2
$V^{(IV)}$	0	L_2	18.9	25.0	32.5

Computational details

Coordinates of the ground and transition states:

Sc^{III} original ligand (L_0)

1

Symbol	X	Y	Z
C	-1.2524860	-3.3181290	-0.9023490
C	-1.2999840	-1.9940730	-0.3864310
C	-2.5904600	-1.3950800	-0.3122040
C	-2.3960210	-3.9913250	-1.3002150
C	-3.6678350	-3.4040510	-1.2220620
C	-3.7258090	-2.1015690	-0.7246340
N	-0.1802170	-1.2851620	-0.0274290
P	-2.6301590	0.3007320	0.3718710
C	-4.1359500	1.0734380	-0.4093940
C	-4.3630940	2.4788410	0.1517960
C	-3.9752850	1.1132060	-1.9314270
C	-3.0472440	-0.0101970	2.1707560
C	-4.4387900	-0.5796790	2.4276150
C	-2.7352410	1.2062240	3.0432900
C	-4.9031160	-4.1517330	-1.6537690
C	3.3951950	-2.1531680	0.4045330
C	2.2333670	-1.5344760	-0.0604100
C	0.9588560	-1.9659690	0.3883560
C	3.3584920	-3.2123050	1.3161920
C	2.0981040	-3.6459440	1.7456440
C	0.9294860	-3.0438280	1.2966330
C	4.6250470	-3.8290810	1.8515480
P	2.1581310	-0.1583810	-1.2631200
C	3.9083900	0.4249240	-1.4988510
C	1.7044490	-1.1139700	-2.8158080
C	0.9998180	-0.2300390	-3.8457600
C	2.8325700	-1.9517520	-3.4106440
C	3.9828630	1.3869130	-2.6875490
C	4.4083580	1.1103580	-0.2262070

Sc	0.0685080	1.0277550	0.0483900
C	0.9353440	1.5002100	1.7806110
C	1.4565390	1.1933580	3.1781010
C	1.0948460	2.3087190	4.1786170
C	2.9893880	1.0245970	3.1804250
C	0.8459990	-0.1258440	3.6779430
C	-0.3631580	2.8178540	-1.4083900
C	0.0606230	4.2748230	-1.1286170
C	1.5865540	4.3558680	-0.9782940
C	-0.3586840	5.2218800	-2.2704770
C	-0.5995140	4.7651460	0.1688270
H	-0.2831160	-3.8124630	-0.9946830
H	-2.2986550	-5.0073310	-1.6979320
H	-4.7053440	-1.6172440	-0.6571290
H	-5.0164730	0.4545630	-0.1650610
H	-4.6507930	2.4617840	1.2126680
H	-5.1723320	2.9845960	-0.4003140
H	-3.4538620	3.0939470	0.0594080
H	-3.1083490	1.7266630	-2.2219430
H	-4.8709100	1.5583510	-2.3957010
H	-3.8359480	0.1112090	-2.3625360
H	-2.2976820	-0.7842290	2.4136780
H	-4.5464290	-0.8617720	3.4883150
H	-4.6348520	-1.4798250	1.8254750
H	-5.2269320	0.1597490	2.2084630
H	-3.4741290	2.0123310	2.9125760
H	-1.7372940	1.6050860	2.7953820
H	-2.7443660	0.9231780	4.1091150
H	-5.0578140	-5.0646920	-1.0544960
H	-4.8435360	-4.4662490	-2.7090810
H	-5.8053330	-3.5310020	-1.5461420
H	4.3699060	-1.8090070	0.0459770

H	2.0288520	-4.4676000	2.4656770
H	-0.0363680	-3.3917790	1.6695470
H	5.4683080	-3.6857510	1.1587580
H	4.5104190	-4.9108540	2.0221450
H	4.9147700	-3.3766010	2.8151360
H	4.5464040	-0.4498490	-1.7160630
H	0.9438070	-1.8053410	-2.4174410
H	0.6330860	-0.8401300	-4.6881800
H	1.6609390	0.5460550	-4.2618930
H	0.1331370	0.2812640	-3.3998450
H	3.6213890	-1.3274490	-3.8603600
H	2.4457430	-2.6095480	-4.2070800
H	3.2999760	-2.5958520	-2.6493830
H	3.7455340	0.8982770	-3.6434600
H	5.0002100	1.8023490	-2.7749760
H	3.2877460	2.2310320	-2.5552020
H	5.4315440	1.4932270	-0.3786520
H	4.4239220	0.4308740	0.6357690
H	3.7548510	1.9522260	0.0479950
H	1.4527000	2.0894820	5.2034810
H	0.0029070	2.4502760	4.2226420
H	1.5384740	3.2698300	3.8671090
H	3.4846580	1.9295320	2.7903350
H	3.3917580	0.8266860	4.1937940
H	3.2711580	0.1805820	2.5311630
H	1.2290210	-0.4094180	4.6766260
H	1.0787810	-0.9430760	2.9784270
H	-0.2506670	-0.0473300	3.7436740
H	1.1871010	2.5590510	1.4985890
H	0.0541740	2.5262210	-2.4000720
H	-1.4640190	2.8217180	-1.5701850
H	1.9273490	5.3856030	-0.7675040

H	2.0858100	4.0252180	-1.9051700
H	1.9361310	3.7054340	-0.1613540
H	-0.0693060	6.2721390	-2.0777810
H	-1.4520570	5.1976430	-2.4158550
H	0.1056040	4.9162270	-3.2236250
H	-0.3482250	4.1023910	1.0124590
H	-1.6988080	4.7687050	0.0683550
H	-0.2889810	5.7940280	0.4265050

1-2-TS

Symbol	X	Y	Z
C	0.1554040	3.4144060	1.7945510
C	-0.2200400	2.4690850	0.8110320
C	-1.5666430	2.5242900	0.3536000
C	-0.7458290	4.3480340	2.2885270
C	-2.0727860	4.4028970	1.8394470
C	-2.4533390	3.4748020	0.8657180
N	0.6243920	1.4844810	0.3325910
P	-1.9555830	1.2320830	-0.8835090
C	-3.8078660	1.1987210	-1.0217550
C	-4.2221110	0.3579590	-2.2316960
C	-4.3744350	0.5884070	0.2634630
C	-1.2695820	1.9975470	-2.4448400
C	-2.0135370	3.2408790	-2.9224660
C	-1.0563160	0.9588530	-3.5489890
C	-3.0453650	5.4077240	2.4022080
C	4.1980460	1.0432460	-0.5357460
C	2.9054210	0.7529350	-0.0838800
C	1.9159480	1.7816630	-0.0087330
C	4.6009090	2.3223310	-0.9204230

C	3.6344100	3.3360960	-0.8315420
C	2.3443330	3.0843410	-0.3963080
C	5.9936280	2.6085070	-1.4201910
P	2.3793370	-0.9170820	0.4529680
C	3.3963230	-2.0962480	-0.5707000
C	3.0632080	-0.9897120	2.2019650
C	2.4210180	-2.0979630	3.0392630
C	4.5858730	-1.0193930	2.3020580
C	3.0981760	-3.5315060	-0.1312080
C	3.0337860	-1.8958930	-2.0436580
Sc	-0.3277370	-0.7597660	-0.0528730
C	-0.7912870	-2.2611160	-1.1142040
C	-1.1987000	-3.4302780	-2.0014810
C	-2.5769600	-4.0091520	-1.6240180
C	-0.1749730	-4.5819240	-1.9268620
C	-1.2761920	-2.9744890	-3.4729260
C	-1.4672240	-1.6817580	1.8038850
C	-2.1268200	-2.8792750	2.5070240
C	-1.2837200	-4.1326030	2.2368430
C	-2.2132590	-2.6404400	4.0210720
C	-3.5435560	-3.0639760	1.9494330
H	1.1789990	3.3951000	2.1768650
H	-0.4115070	5.0520590	3.0579820
H	-3.4829710	3.5006720	0.4942810
H	-4.1933660	2.2259810	-1.1449680
H	-4.0012180	0.8599730	-3.1847920
H	-5.3062170	0.1589920	-2.2063640
H	-3.6913120	-0.6084790	-2.2227350
H	-4.0286250	-0.4523860	0.3679940
H	-5.4767000	0.5790820	0.2341510
H	-4.0667960	1.1402790	1.1648300
H	-0.2711840	2.3058040	-2.0903290

H	-1.4713000	3.7173500	-3.7565120
H	-2.1133420	3.9897990	-2.1211040
H	-3.0236400	2.9958970	-3.2897640
H	-2.0003630	0.6868380	-4.0456950
H	-0.6245640	0.0286400	-3.1410280
H	-0.3795890	1.3596750	-4.3220310
H	-3.9943260	5.4030750	1.8452750
H	-2.6420430	6.4326630	2.3621990
H	-3.2835450	5.1966050	3.4584440
H	4.9282350	0.2300320	-0.6019390
H	3.8988840	4.3556940	-1.1328870
H	1.6233310	3.9040210	-0.3738890
H	6.6267940	1.7094730	-1.3771210
H	6.4907690	3.3912840	-0.8231870
H	5.9896620	2.9571700	-2.4669020
H	4.4700790	-1.8870870	-0.4230800
H	2.7091190	-0.0246890	2.6050060
H	2.7064040	-1.9901110	4.0990160
H	2.7372190	-3.1002090	2.7141010
H	1.3236990	-2.0670510	2.9833700
H	4.9985020	-1.9647000	1.9130510
H	4.9042280	-0.9347280	3.3548240
H	5.0499080	-0.1891560	1.7498570
H	3.5070610	-3.7577670	0.8651800
H	3.5426780	-4.2500550	-0.8395210
H	2.0081300	-3.6969900	-0.1126930
H	3.5384800	-2.6522750	-2.6677730
H	3.3282560	-0.9014770	-2.4116520
H	1.9419510	-1.9992300	-2.1743340
H	-2.9072680	-4.8033680	-2.3226940
H	-3.3422150	-3.2158470	-1.6238550
H	-2.5520460	-4.4500990	-0.6151540

H	-0.0865790	-4.9567380	-0.8939330
H	-0.4487230	-5.4377640	-2.5763760
H	0.8225200	-4.2296400	-2.2339090
H	-1.5451330	-3.8020120	-4.1603920
H	-0.3075800	-2.5626680	-3.7984200
H	-2.0269720	-2.1772040	-3.5906520
H	-1.3700940	-2.1586610	0.5661290
H	-0.5005160	-1.4658510	2.3087110
H	-2.0930330	-0.7750890	1.9362790
H	-1.7539460	-5.0384170	2.6555120
H	-0.2812230	-4.0328470	2.6856220
H	-1.1359730	-4.2704110	1.1551070
H	-2.7006020	-3.4864750	4.5372020
H	-2.7920790	-1.7292140	4.2457800
H	-1.2097600	-2.5134240	4.4601550
H	-3.5233720	-3.1588710	0.8544970
H	-4.1764440	-2.1955820	2.1978530
H	-4.0289630	-3.9628560	2.3659480

A

Symbol	X	Y	Z
C	-1.6448220	-3.1607970	-0.8598010
C	-1.5002750	-1.8181000	-0.4312320
C	-2.6903860	-1.0348140	-0.3892300
C	-2.8801270	-3.6830190	-1.2193910
C	-4.0501090	-2.9121390	-1.1791050
C	-3.9205440	-1.5861400	-0.7545290
N	-0.2886610	-1.2337360	-0.1196930
P	-2.3955350	0.6825840	0.1762140
C	-3.9431040	1.6288650	-0.2218650

C	-3.8963040	2.9977580	0.4614280
C	-4.0337070	1.7940830	-1.7414920
C	-2.3381990	0.4216280	2.0301440
C	-3.6790260	0.0467470	2.6542530
C	-1.6283210	1.5649630	2.7551240
C	-5.3803050	-3.4915420	-1.5877460
C	3.0240340	-2.2844090	1.1742740
C	2.0533390	-1.6056600	0.4283650
C	0.6723140	-1.9416520	0.5591470
C	2.7136110	-3.3039760	2.0741950
C	1.3552250	-3.6299910	2.2093950
C	0.3701340	-2.9784890	1.4859220
C	3.7732990	-4.0065780	2.8830760
P	2.4483100	-0.2746190	-0.7642240
C	4.0122410	0.5075500	-0.1255960
C	2.9012670	-1.2592680	-2.2968250
C	2.7664390	-0.4230320	-3.5714980
C	4.2381830	-1.9911820	-2.2249650
C	4.4727880	1.5809590	-1.1138660
C	3.7032980	1.1272790	1.2397070
Sc	0.1168530	1.1395970	-0.7133830
C	0.5384310	2.9115220	-0.2207210
C	0.8423110	4.3431880	0.1992710
C	-0.0444570	5.3444800	-0.5720970
C	2.3125680	4.7111900	-0.0920080
C	0.5964910	4.5661150	1.7065130
H	-0.7563990	-3.7942420	-0.9178710
H	-2.9367360	-4.7246490	-1.5532780
H	-4.8204850	-0.9643640	-0.7035280
H	-4.8248820	1.0711670	0.1388350
H	-4.0297520	2.9247660	1.5505480
H	-4.6973900	3.6480190	0.0728210

H	-2.9253000	3.4851090	0.2713380
H	-3.1769500	2.3825520	-2.1103330
H	-4.9554850	2.3322180	-2.0183890
H	-4.0363190	0.8285560	-2.2703260
H	-1.6740260	-0.4576360	2.0840570
H	-3.5432020	-0.2424080	3.7098890
H	-4.1474450	-0.8042740	2.1350710
H	-4.3884570	0.8904350	2.6383700
H	-2.2612990	2.4629080	2.8281480
H	-0.7106210	1.8595240	2.2161730
H	-1.3648470	1.2588820	3.7815130
H	-6.2009230	-2.7818170	-1.4041420
H	-5.6108840	-4.4150900	-1.0318190
H	-5.4016160	-3.7487120	-2.6603150
H	4.0745500	-1.9989820	1.0578500
H	1.0592170	-4.4109230	2.9183660
H	-0.6747500	-3.2546860	1.6429020
H	4.7820700	-3.6763410	2.5926130
H	3.7325660	-5.1005640	2.7509390
H	3.6617510	-3.8088880	3.9626290
H	4.8009680	-0.2578920	-0.0235530
H	2.0953320	-2.0136740	-2.3100870
H	2.8868100	-1.0589070	-4.4646250
H	3.5233240	0.3736520	-3.6284010
H	1.7793190	0.0604850	-3.6363900
H	5.0863340	-1.2875220	-2.2186050
H	4.3670560	-2.6498120	-3.1004490
H	4.3090840	-2.6207410	-1.3252680
H	4.8297480	1.1528690	-2.0633330
H	5.3021070	2.1668220	-0.6845370
H	3.6391460	2.2706730	-1.3268160
H	4.5534860	1.7370500	1.5879900

H	3.4949570	0.3638060	2.0044110
H	2.8087220	1.7717050	1.1492170
H	0.1765910	6.4017310	-0.3175240
H	-1.1094260	5.1586150	-0.3559000
H	0.0946800	5.2109500	-1.6572040
H	2.5284770	4.5939650	-1.1668750
H	2.5533640	5.7554050	0.1943910
H	2.9973520	4.0449060	0.4565050
H	0.8090060	5.6064140	2.0279860
H	1.2305090	3.8892910	2.3023050
H	-0.4519710	4.3415490	1.9606670

5-6-TS

Symbol	X	Y	Z
C	1.5234510	-3.2308540	0.4139610
C	1.4513130	-1.8391600	0.1396650
C	2.6791660	-1.1162780	0.2245340
C	2.7197340	-3.8549270	0.7343840
C	3.9277230	-3.1468750	0.8060160
C	3.8694750	-1.7753560	0.5446840
N	0.2791010	-1.1770870	-0.1382240
P	2.5241650	0.6779970	-0.1180710
C	4.0962530	1.4320900	0.5350000
C	4.2056350	2.8922590	0.0885830
C	4.1094580	1.3364220	2.0629930
C	2.6339620	0.7017830	-1.9871050
C	3.9729070	0.2313230	-2.5465880
C	2.1623820	2.0292970	-2.5819890
C	5.2185960	-3.8352630	1.1695410

C	-3.1239310	-2.1636890	-1.2482190
C	-2.1010850	-1.5370460	-0.5254700
C	-0.7365330	-1.8216080	-0.8137010
C	-2.8748920	-3.0780570	-2.2712660
C	-1.5289790	-3.3479770	-2.5637060
C	-0.4963760	-2.7435240	-1.8661260
C	-3.9897100	-3.7323450	-3.0460060
P	-2.4064490	-0.3765960	0.8568220
C	-4.0795720	0.3760220	0.5245180
C	-2.6418550	-1.5863920	2.2807970
C	-2.3217680	-0.9475260	3.6333820
C	-3.9785500	-2.3219000	2.2941590
C	-4.3880350	1.3703730	1.6465060
C	-4.0610340	1.0845400	-0.8313360
Sc	-0.1203940	1.0994690	0.5723900
C	-0.6765220	2.7025260	-0.2464550
C	-1.0879340	4.0695230	-0.7718710
C	0.0904450	5.0628480	-0.6780360
C	-2.2624470	4.6487430	0.0461270
C	-1.5300730	3.9980630	-2.2484280
H	0.6054580	-3.8217370	0.3795340
H	2.7147470	-4.9296340	0.9462610
H	4.8002640	-1.2010180	0.5901670
H	4.9608350	0.8751230	0.1341850
H	4.4195170	2.9819090	-0.9857010
H	5.0225520	3.3974160	0.6303050
H	3.2695530	3.4386850	0.2898020
H	3.3024220	1.9488610	2.4962830
H	5.0645020	1.7143520	2.4641970
H	3.9790100	0.3046140	2.4221660
H	1.8643810	-0.0512760	-2.2311720
H	3.9139230	0.1252860	-3.6428120

H	4.2725070	-0.7448890	-2.1344270
H	4.7782710	0.9546670	-2.3357890
H	2.9266350	2.8176300	-2.4952420
H	1.2477320	2.3685980	-2.0609570
H	1.9394430	1.9043480	-3.6549390
H	5.2331120	-4.1486270	2.2276210
H	6.0839570	-3.1735770	1.0130030
H	5.3813870	-4.7416160	0.5637940
H	-4.1649060	-1.9288270	-1.0048530
H	-1.2843660	-4.0435320	-3.3737050
H	0.5370740	-2.9685950	-2.1392400
H	-3.9278960	-4.8325030	-3.0035220
H	-3.9643370	-3.4499570	-4.1118980
H	-4.9752180	-3.4407240	-2.6530210
H	-4.8487940	-0.4154900	0.5217910
H	-1.8460420	-2.3214230	2.0694130
H	-2.3680500	-1.7017330	4.4369840
H	-3.0289640	-0.1444250	3.8940770
H	-1.3095860	-0.5158500	3.6426240
H	-4.8140710	-1.6421630	2.5287020
H	-3.9771170	-3.1137600	3.0621130
H	-4.1890870	-2.8016130	1.3262230
H	-4.4844250	0.8821580	2.6288860
H	-5.3359040	1.8968470	1.4465060
H	-3.5854360	2.1238280	1.7090910
H	-4.9635290	1.7074760	-0.9495790
H	-4.0242640	0.3758180	-1.6710280
H	-3.1643750	1.7256160	-0.8987940
H	-0.1735460	6.0802420	-1.0327580
H	0.9417740	4.7042250	-1.2786780
H	0.4383960	5.1442330	0.3651610
H	-1.9871380	4.7288140	1.1111920

H	-2.5745530	5.6544040	-0.3020320
H	-3.1380650	3.9824700	-0.0171390
H	-1.8308020	4.9859720	-2.6524890
H	-2.3834830	3.3103960	-2.3629410
H	-0.7131980	3.6083370	-2.8768930
C	-0.0448300	2.3593990	2.7315280
H	-0.3266260	2.7249000	1.5726640
H	0.9516440	1.9191670	2.9224480
H	-0.8134240	1.7336210	3.2177040
H	-0.0613250	3.3440810	3.2223630

6

Symbol	X	Y	Z
C	1.2033380	-2.7630680	-1.2675530
C	1.2605050	-1.4469830	-0.7309860
C	2.5569930	-0.9637980	-0.3873220
C	2.3399740	-3.5357250	-1.4375830
C	3.6152800	-3.0667040	-1.0876400
C	3.6839760	-1.7754510	-0.5636660
N	0.1435070	-0.6888540	-0.4855390
P	2.6240350	0.7414050	0.2792470
C	4.0536660	0.6963900	1.4754980
C	4.3456040	2.0934020	2.0269950
C	3.7286400	-0.2786240	2.6096630
C	3.1744210	1.7485960	-1.1985220
C	4.5667550	1.4281420	-1.7330140
C	2.9638600	3.2461670	-0.9678070
C	4.8407320	-3.9250940	-1.2688840
C	-3.4511350	-1.0800190	-1.2948900

C	-2.2700120	-0.9220740	-0.5644250
C	-1.0148250	-0.9074000	-1.2230740
C	-3.4511680	-1.2213020	-2.6840250
C	-2.2077370	-1.2034620	-3.3315880
C	-1.0228160	-1.0510060	-2.6263990
C	-4.7332980	-1.3485810	-3.4653240
P	-2.1529850	-0.6842600	1.2453530
C	-3.8892410	-0.3664800	1.8281300
C	-1.7037570	-2.4233010	1.7833000
C	-1.0186400	-2.4405030	3.1502870
C	-2.8303360	-3.4446350	1.6605970
C	-3.9532830	-0.4244100	3.3566430
C	-4.3465690	1.0039310	1.3244930
Sc	-0.0388870	1.0608120	1.0000760
C	-0.8105800	2.6659100	0.1106940
C	-1.4981350	3.4527930	-0.9956990
C	-2.8106730	4.0996880	-0.5087480
C	-1.8321010	2.5007680	-2.1556240
C	-0.5982630	4.5791570	-1.5451400
C	0.3397160	1.3926330	3.2707680
H	0.2304010	-3.1806350	-1.5346180
H	2.2339110	-4.5480070	-1.8424540
H	4.6663010	-1.3862670	-0.2772480
H	4.9530340	0.3388680	0.9451210
H	4.7512650	2.7674730	1.2593530
H	5.0914720	2.0334500	2.8369020
H	3.4352420	2.5564910	2.4398010
H	2.8310950	0.0478280	3.1576460
H	4.5664550	-0.3243110	3.3253180
H	3.5460120	-1.2981660	2.2388920
H	2.4246910	1.4293560	-1.9437180
H	4.7611310	1.9961990	-2.6581420

H	4.6830360	0.3605050	-1.9717740
H	5.3525930	1.7068900	-1.0112420
H	3.7364410	3.6788060	-0.3135670
H	1.9758270	3.4268700	-0.5138950
H	3.0090660	3.7874020	-1.9275070
H	4.7559100	-4.8784430	-0.7212320
H	5.7451050	-3.4153740	-0.9038290
H	5.0111100	-4.1784110	-2.3288090
H	-4.4110280	-1.0913750	-0.7699290
H	-2.1696690	-1.2940430	-4.4220050
H	-0.0712900	-1.0190210	-3.1620810
H	-5.5915790	-1.5365200	-2.8029420
H	-4.6868650	-2.1723310	-4.1956240
H	-4.9507740	-0.4269300	-4.0308120
H	-4.5533850	-1.1482980	1.4201040
H	-0.9322170	-2.6734590	1.0360600
H	-0.6091910	-3.4425310	3.3613060
H	-1.7078770	-2.1856400	3.9702800
H	-0.1878270	-1.7192760	3.1828780
H	-3.6242470	-3.2748520	2.4060100
H	-2.4458680	-4.4657470	1.8224810
H	-3.2905830	-3.4190990	0.6604360
H	-3.7658270	-1.4345760	3.7485870
H	-4.9530720	-0.1177380	3.7053700
H	-3.2171100	0.2580350	3.8110780
H	-5.3672780	1.2215320	1.6814660
H	-4.3476440	1.0657800	0.2269390
H	-3.6677930	1.7927030	1.6837920
H	-3.3197170	4.6758820	-1.3061040
H	-2.6175310	4.7879460	0.3316020
H	-3.5087560	3.3291670	-0.1487320
H	-2.5098600	1.7005760	-1.8191450

H	-2.3105260	3.0253380	-3.0043940
H	-0.9153650	2.0134810	-2.5248340
H	-1.1016090	5.1756570	-2.3316020
H	0.3217980	4.1574230	-1.9784410
H	-0.2997300	5.2698410	-0.7384000
H	-0.6075500	1.8170450	3.6698050
H	0.5672260	0.5160350	3.9131660
H	1.1182110	2.1512290	3.4981920
H	-0.6645830	3.3172500	1.0237450

6’

Symbol	X	Y	Z
C	-1.1905730	-3.2130770	-0.1610100
C	-1.2543690	-1.8076270	0.0123450
C	-2.5305510	-1.2029500	-0.1709080
C	-2.3111200	-3.9567730	-0.5044080
C	-3.5645460	-3.3613590	-0.6988210
C	-3.6394410	-1.9768830	-0.5186090
N	-0.1591820	-1.0226210	0.2972620
P	-2.5468950	0.5996960	0.1494230
C	-4.1209650	1.2133480	-0.6276760
C	-4.3781520	2.6693470	-0.2313270
C	-4.0259460	1.0760910	-2.1494730
C	-2.8163660	0.5991670	2.0049030
C	-4.1780630	0.0722590	2.4471140
C	-2.4553690	1.9360840	2.6506550
C	-4.7660610	-4.1742680	-1.1080900
C	3.3032550	-1.8127500	1.3626750
C	2.2335900	-1.2993650	0.6212100

C	0.8967270	-1.5630870	1.0143100
C	3.1134700	-2.5861750	2.5090410
C	1.7895850	-2.8428280	2.8961240
C	0.7125490	-2.3499850	2.1751700
C	4.2749870	-3.0987380	3.3211830
P	2.3888270	-0.3156350	-0.9155890
C	4.1092670	0.3929010	-0.8734450
C	2.3834870	-1.6824990	-2.2006210
C	1.9652130	-1.1709770	-3.5797790
C	3.6526120	-2.5277940	-2.2459730
C	4.3569830	1.1846690	-2.1602660
C	4.2514680	1.2974540	0.3532960
Sc	0.0684840	1.1319490	-0.6028380
C	0.6963200	2.6627100	0.5534390
C	0.9040540	4.1697810	0.6551060
C	-0.0013630	4.7971460	1.7355320
C	0.5531590	4.8198620	-0.6938600
C	2.3619300	4.5415210	0.9961610
C	-0.3999440	1.6352770	-2.7971280
H	-0.2311090	-3.7191500	-0.0318620
H	-2.2051580	-5.0387940	-0.6372330
H	-4.6108400	-1.4890090	-0.6484250
H	-4.9618070	0.5984340	-0.2622360
H	-4.6209740	2.7745330	0.8357590
H	-5.2287100	3.0747360	-0.8040180
H	-3.4986190	3.2997080	-0.4403460
H	-3.2179850	1.7075850	-2.5486670
H	-4.9718180	1.3922930	-2.6202420
H	-3.8198040	0.0427870	-2.4649420
H	-2.0422700	-0.1255880	2.3125020
H	-4.2034120	-0.0533630	3.5425430
H	-4.4077400	-0.9055640	1.9964660

H	-4.9891060	0.7711040	2.1832770
H	-3.2098290	2.7132060	2.4515240
H	-1.4897640	2.2962880	2.2631910
H	-2.3808730	1.8229110	3.7451990
H	-4.7258620	-4.4544600	-2.1748280
H	-5.7012570	-3.6144570	-0.9546040
H	-4.8404600	-5.1103590	-0.5316810
H	4.3273270	-1.6060620	1.0362280
H	1.6001450	-3.4373280	3.7959510
H	-0.3063600	-2.5633490	2.5073650
H	4.3706960	-2.5536160	4.2755770
H	5.2263480	-2.9819180	2.7808130
H	4.1606950	-4.1662070	3.5691350
H	4.8426880	-0.4303080	-0.8163580
H	1.5585780	-2.3130300	-1.8261380
H	1.7939990	-2.0177440	-4.2653050
H	2.7333990	-0.5278330	-4.0369910
H	1.0362030	-0.5855920	-3.5205390
H	4.5059580	-1.9571570	-2.6480080
H	3.5100830	-3.4039670	-2.9006090
H	3.9302140	-2.9020500	-1.2483370
H	4.3355130	0.5480170	-3.0573210
H	5.3454940	1.6713220	-2.1249950
H	3.5966800	1.9730870	-2.2837120
H	5.1779460	1.8918140	0.2872010
H	4.2818160	0.7256950	1.2917560
H	3.3877310	1.9801050	0.4178890
H	0.1342130	5.8936230	1.8178280
H	0.2112520	4.3594040	2.7257440
H	-1.0617980	4.6022910	1.5092980
H	-0.4976620	4.6132840	-0.9558480
H	0.6972070	5.9168510	-0.6837180

H	1.1771070	4.3952670	-1.4972250
H	2.5032090	5.6347060	1.1061270
H	3.0478010	4.1945540	0.2071430
H	2.6767330	4.0675740	1.9411160
H	-1.0235040	2.5534830	-2.8486200
H	0.5379670	1.8883020	-3.3367040
H	-0.9204770	0.8709530	-3.4106730
H	0.9523700	2.1666150	1.5305950

6’-7’-TS

Symbol	X	Y	Z
C	-1.1637440	-3.3110630	-0.4784610
C	-1.2140410	-1.9257140	-0.1825280
C	-2.4903840	-1.3025810	-0.2762340
C	-2.2980330	-4.0195580	-0.8471960
C	-3.5544280	-3.4048970	-0.9471060
C	-3.6158950	-2.0404960	-0.6522700
N	-0.1029560	-1.1722750	0.1295620
P	-2.4813900	0.4695420	0.1824750
C	-4.0887340	1.1507710	-0.4594330
C	-4.3367650	2.5546580	0.0971340
C	-4.0423180	1.1841880	-1.9890330
C	-2.6439070	0.3313860	2.0452490
C	-3.9791640	-0.2365970	2.5183670
C	-2.2533950	1.6158100	2.7775700
C	-4.7736660	-4.1836380	-1.3701250
C	3.3053690	-1.8417990	1.4090780
C	2.2649900	-1.3449310	0.6212260
C	0.9167660	-1.7266980	0.8741100

C	3.0861120	-2.7292160	2.4668370
C	1.7606970	-3.1180900	2.7045050
C	0.7075860	-2.6383610	1.9377080
C	4.2180920	-3.2195430	3.3329910
P	2.4484940	-0.2264380	-0.8143360
C	4.1671120	0.4714750	-0.6922010
C	2.4490800	-1.4743130	-2.2122570
C	2.1639230	-0.8453440	-3.5765580
C	3.6651540	-2.3962550	-2.2297470
C	4.5176150	1.2257970	-1.9775820
C	4.2465100	1.4088490	0.5156050
Sc	0.0681040	1.1390920	-0.6306210
C	0.4671940	2.8907910	0.2859190
C	0.5465710	4.3786530	0.5855860
C	-0.7832260	5.0333540	0.1762740
C	1.6884640	5.0551780	-0.2030290
C	0.7796290	4.6248450	2.0859320
C	-0.1127650	2.1256990	-2.4003640
H	-0.2018610	-3.8263710	-0.4252290
H	-2.2037370	-5.0860590	-1.0780650
H	-4.5873850	-1.5396620	-0.7129490
H	-4.9128990	0.4925820	-0.1336860
H	-4.5406820	2.5467850	1.1774160
H	-5.2100950	3.0097470	-0.3985870
H	-3.4681400	3.2083240	-0.0806820
H	-3.2439660	1.8626330	-2.3295810
H	-5.0009710	1.5478170	-2.3947730
H	-3.8452020	0.1933220	-2.4252670
H	-1.8553300	-0.4120800	2.2543170
H	-3.9460650	-0.4435310	3.6011360
H	-4.2285380	-1.1801120	2.0087950
H	-4.8062220	0.4727600	2.3500470

H	-3.0180830	2.4016710	2.6765740
H	-1.3081120	2.0192420	2.3814840
H	-2.1305440	1.4126310	3.8547450
H	-4.8803300	-5.1172870	-0.7941830
H	-4.7290310	-4.4655850	-2.4358910
H	-5.6943600	-3.5982920	-1.2256970
H	4.3336870	-1.5353600	1.1916760
H	1.5441560	-3.8101020	3.5252350
H	-0.3120570	-2.9569980	2.1667290
H	4.2895030	-2.6472640	4.2741080
H	5.1874770	-3.1229790	2.8205970
H	4.0909330	-4.2783630	3.6084370
H	4.8858230	-0.3560270	-0.5583320
H	1.5624390	-2.0721250	-1.9385210
H	1.9149210	-1.6324590	-4.3080550
H	3.0342210	-0.2967020	-3.9687810
H	1.3247570	-0.1325050	-3.5211060
H	4.5827170	-1.8541190	-2.5124120
H	3.5252040	-3.2027560	-2.9690360
H	3.8348890	-2.8703570	-1.2505620
H	4.6499550	0.5500070	-2.8347180
H	5.4609440	1.7815010	-1.8472570
H	3.7274220	1.9493750	-2.2361960
H	5.2716220	1.7979990	0.6321420
H	3.9638950	0.9115510	1.4555230
H	3.5677750	2.2647610	0.3773700
H	-0.7636850	6.1304920	0.3108050
H	-1.6106200	4.6249960	0.7782290
H	-1.0085380	4.8167490	-0.8805810
H	1.5528970	4.8990130	-1.2854060
H	1.7462190	6.1448990	-0.0168420
H	2.6619930	4.6158680	0.0719940

H	0.8216310	5.7031060	2.3297590
H	1.7295230	4.1695220	2.4138800
H	-0.0264640	4.1687090	2.6826610
H	0.1978270	2.8384300	-1.1635540
H	-0.0655410	2.9109460	-3.1724060
H	-1.0530960	1.5268750	-2.5656630
H	1.3706630	2.3490380	0.6946090

6’-7”-TS

Symbol	X	Y	Z
C	-1.4876900	-3.2281940	-0.1281710
C	-1.4084520	-1.8183300	0.0428620
C	-2.6301970	-1.1009810	-0.1090050
C	-2.6815720	-3.8672050	-0.4179280
C	-3.8848740	-3.1600310	-0.5664380
C	-3.8197340	-1.7759080	-0.4079440
N	-0.2293410	-1.1514450	0.2700170
P	-2.5255560	0.7076980	0.1342620
C	-3.8680270	1.4213310	-0.9417690
C	-3.8943330	2.9443780	-0.7863590
C	-3.6158350	1.0340880	-2.4013350
C	-3.0807500	0.8904480	1.9166470
C	-4.5166130	0.4607460	2.2030840
C	-2.7759360	2.2767340	2.4900760

C	-5.1744900	-3.8691340	-0.8917670
C	3.2085650	-2.1497720	1.2289770
C	2.1503360	-1.5451380	0.5422980
C	0.8041530	-1.7810760	0.9341230
C	3.0071920	-2.9926950	2.3226910
C	1.6791270	-3.2125340	2.7192910
C	0.6122030	-2.6284990	2.0543790
C	4.1561480	-3.6326190	3.0586830
P	2.3394150	-0.4427110	-0.9024370
C	4.0841310	0.1970500	-0.8281100
C	2.2451050	-1.6682600	-2.3158210
C	1.8263890	-1.0017300	-3.6273970
C	3.4743840	-2.5587240	-2.4678150
C	4.3596360	1.0520830	-2.0680500
C	4.2648730	1.0224120	0.4488130
Sc	0.1279960	1.1067180	-0.4994730
C	0.7770530	2.7253610	0.5252920
C	1.2872700	4.1199700	0.8573480
C	0.1266830	5.1340160	0.9378670
C	2.2557250	4.5771650	-0.2459730
C	2.0291300	4.1264350	2.2046230
C	-0.0850860	2.2609220	-2.1868450
H	-0.5737640	-3.8201750	-0.0465280
H	-2.6793660	-4.9546100	-0.5501890
H	-4.7403390	-1.1957040	-0.5285180
H	-4.8417980	1.0136720	-0.6179200
H	-4.2213910	3.2592870	0.2150370
H	-4.5930480	3.3889540	-1.5141810
H	-2.8949700	3.3708370	-0.9700640
H	-2.6121150	1.3730190	-2.7125920
H	-4.3719750	1.5046530	-3.0521650
H	-3.6646230	-0.0540020	-2.5560980

H	-2.3981290	0.1737730	2.4072140
H	-4.7162400	0.4871960	3.2875440
H	-4.7176210	-0.5631290	1.8551370
H	-5.2434840	1.1369030	1.7232120
H	-3.5180150	3.0243820	2.1708270
H	-1.7856520	2.6363680	2.1742120
H	-2.7969390	2.2473790	3.5919730
H	-5.1144730	-4.4072510	-1.8525560
H	-6.0136070	-3.1610380	-0.9640420
H	-5.4372520	-4.6146990	-0.1227490
H	4.2350350	-1.9583470	0.9005450
H	1.4778740	-3.8513200	3.5858720
H	-0.4059650	-2.8130880	2.4047530
H	4.1816180	-3.3259870	4.1175940
H	5.1228310	-3.3549520	2.6126030
H	4.0888080	-4.7332200	3.0445250
H	4.7868250	-0.6544680	-0.8207870
H	1.3991230	-2.2956940	-1.9862070
H	1.6098560	-1.7672190	-4.3912370
H	2.6137510	-0.3462750	-4.0315480
H	0.9217870	-0.3876010	-3.4930030
H	4.3471880	-1.9885070	-2.8261850
H	3.2846160	-3.3588310	-3.2028020
H	3.7486120	-3.0418980	-1.5169390
H	4.3504980	0.4604030	-2.9955810
H	5.3512170	1.5275390	-1.9907060
H	3.6072510	1.8514660	-2.1666070
H	5.2450470	1.5277670	0.4413390
H	4.2080910	0.4038410	1.3563290
H	3.4674160	1.7804890	0.5255190
H	0.4731680	6.1576330	1.1770850
H	-0.5974750	4.8338620	1.7135040

H	-0.4156400	5.1723360	-0.0206160
H	1.7739040	4.5102080	-1.2347980
H	2.5928690	5.6180690	-0.0911060
H	3.1462570	3.9291410	-0.2756710
H	2.4209900	5.1298110	2.4569530
H	2.8756320	3.4218820	2.1900070
H	1.3566550	3.8143230	3.0216310
H	0.2943880	2.8685290	-0.8213500
H	-0.4304080	3.1525450	-2.7397090
H	0.8296710	1.8706690	-2.7054970
H	0.1358310	2.3314740	1.3679860

7’

Symbol	X	Y	Z
C	-0.7156640	-3.2947330	-0.6974460
C	-0.9363560	-1.9566690	-0.2909100
C	-2.2758840	-1.4893700	-0.3235550
C	-1.7580130	-4.1125140	-1.1057140
C	-3.0834260	-3.6546380	-1.1372340
C	-3.3092070	-2.3365930	-0.7374040
N	0.0889550	-1.1026930	0.0750660
P	-2.5077530	0.2201980	0.2943770
C	-4.1751460	0.7421050	-0.3543810
C	-4.5441100	2.1208990	0.1983790
C	-4.1686630	0.7617950	-1.8848890
C	-2.7377470	-0.1452860	2.1264460
C	-4.0829060	-0.7595370	2.5018090
C	-2.3718640	1.0476980	3.0091410
C	-4.2064450	-4.5491090	-1.5960240
C	3.5238020	-1.5292640	1.3895230
C	2.4548080	-1.0733570	0.6125010

C	1.1475540	-1.5942690	0.8118840
C	3.3701310	-2.4983430	2.3840600
C	2.0806600	-3.0143450	2.5746770
C	1.0022540	-2.5828610	1.8163650
C	4.5301220	-2.9580370	3.2298440
P	2.5677970	0.1686140	-0.7312590
C	4.1810590	1.0462930	-0.4145230
C	2.8388510	-0.9519530	-2.2106780
C	2.6120830	-0.2429590	-3.5461560
C	4.1438600	-1.7421760	-2.1760420
C	4.5263300	1.9756380	-1.5809230
C	4.1004100	1.8325430	0.8972480
Sc	-0.0552470	1.1130540	-0.6273360
C	0.5841970	3.0562600	0.4620200
C	-0.2453930	4.3545030	0.4649870
C	-0.8014680	4.6156590	-0.9431050
C	0.5992660	5.5711270	0.8903340
C	-1.4199660	4.2180840	1.4412410
C	-0.5939330	1.2876850	-2.5432040
H	0.3062590	-3.6811120	-0.6968550
H	-1.5363950	-5.1370240	-1.4227670
H	-4.3372710	-1.9619630	-0.7457180
H	-4.9267880	0.0152240	-0.0005020
H	-4.5547980	2.1502270	1.2983520
H	-5.5488320	2.4125040	-0.1495550
H	-3.8356610	2.8866010	-0.1544180
H	-3.3108990	1.3458370	-2.2583700
H	-5.1074450	1.2016950	-2.2616150
H	-4.0689700	-0.2458260	-2.3130030
H	-1.9554880	-0.9086120	2.2782790
H	-4.0754680	-1.0925820	3.5533550
H	-4.3192630	-1.6368660	1.8800140

H	-4.9059420	-0.0336280	2.3972140
H	-3.0982270	1.8712810	2.9231470
H	-1.3818590	1.4482420	2.7453670
H	-2.3441600	0.7443940	4.0692710
H	-4.0995660	-4.8221780	-2.6591780
H	-5.1835770	-4.0564430	-1.4814450
H	-4.2394440	-5.4906740	-1.0235730
H	4.5243290	-1.1217110	1.2140020
H	1.9150620	-3.7713170	3.3485230
H	0.0115230	-3.0037580	2.0025850
H	4.4411090	-2.6064800	4.2718040
H	5.4863060	-2.5792020	2.8385860
H	4.5987970	-4.0573310	3.2661200
H	4.9797080	0.2893840	-0.3267880
H	1.9970440	-1.6527330	-2.0717830
H	2.5295540	-0.9872090	-4.3559970
H	3.4437690	0.4310370	-3.8050910
H	1.6805020	0.3485610	-3.5143470
H	5.0184590	-1.0879530	-2.3288710
H	4.1578830	-2.4931550	-2.9836660
H	4.2782690	-2.2777380	-1.2236560
H	4.7699290	1.4200820	-2.4975220
H	5.4038590	2.5935510	-1.3280950
H	3.6904780	2.6552800	-1.8123350
H	5.0748540	2.2981730	1.1209460
H	3.8285070	1.1964670	1.7523550
H	3.3492930	2.6339430	0.8307480
H	-1.3722720	5.5612680	-0.9902100
H	-1.4604390	3.7951380	-1.2712370
H	0.0142470	4.6755010	-1.6822940
H	1.4415640	5.7217470	0.1944700
H	0.0112720	6.5080610	0.9127070

H	1.0264700	5.4190160	1.8960760
H	-2.0548120	5.1224990	1.4545870
H	-1.0630080	4.0449420	2.4708140
H	-2.0521250	3.3623290	1.1627980
H	1.4856360	3.2392070	-0.1687260
H	-0.8349290	1.8746860	-3.4511540
H	-0.7646220	0.2045490	-2.7948040
H	0.9892510	2.8809690	1.4836380

7”

Symbol	X	Y	Z
C	1.2878890	-2.8870450	-0.8625950
C	1.2976450	-1.4805220	-0.6283220
C	2.5731360	-0.9105250	-0.3318170
C	2.4421440	-3.6506750	-0.8111890
C	3.6941890	-3.0893840	-0.5179910
C	3.7168320	-1.7154190	-0.2788510
N	0.1618610	-0.7161360	-0.5988710
P	2.5968660	0.8897910	-0.0216480
C	3.8869110	1.1147830	1.3063270
C	4.0211450	2.5998820	1.6482600
C	3.4748200	0.3246940	2.5510180
C	3.3198820	1.5799320	-1.6095300
C	4.7836030	1.2348180	-1.8702470
C	3.0581810	3.0800610	-1.7630950
C	4.9391950	-3.9358050	-0.4491620
C	-3.3737470	-1.7491130	-1.0237910
C	-2.1972890	-1.2651550	-0.4479220
C	-0.9947250	-1.2140450	-1.1968360
C	-3.4211960	-2.1792210	-2.3534940
C	-2.2342020	-2.1120560	-3.0955320

C	-1.0510350	-1.6425440	-2.5376760
C	-4.7030780	-2.6636630	-2.9806970
P	-1.9874600	-0.5478560	1.2193330
C	-3.6888100	-0.3936180	1.9581150
C	-1.1550330	-1.9663000	2.1194390
C	-0.3686300	-1.5053870	3.3475130
C	-2.0706320	-3.1533860	2.4049220
C	-3.5551100	0.0007130	3.4330010
C	-4.5238140	0.6478390	1.2092130
Sc	-0.0331170	1.3308780	0.6168050
C	-0.5710880	2.9848800	-0.9165010
C	-2.0275720	3.4365850	-1.1283550
C	-2.1417070	4.5649380	-2.1707000
C	-2.5992030	3.9477100	0.2028660
C	-2.8584560	2.2434660	-1.6198770
C	0.2145640	2.0488670	2.4638950
H	0.3373360	-3.3812330	-1.0726450
H	2.3668620	-4.7288850	-0.9900180
H	4.6748490	-1.2509940	-0.0238640
H	4.8541950	0.7346790	0.9335520
H	4.4408180	3.1894420	0.8196560
H	4.6858580	2.7333570	2.5179380
H	3.0277900	3.0051410	1.9088810
H	2.5051210	0.7050430	2.9169880
H	4.2277620	0.4533110	3.3467740
H	3.3754990	-0.7523680	2.3489340
H	2.7000430	1.0598330	-2.3606570
H	5.0872390	1.5897320	-2.8695650
H	4.9661920	0.1509280	-1.8376060
H	5.4497610	1.7196490	-1.1377980
H	3.6683170	3.6787910	-1.0699940
H	2.0036220	3.3308780	-1.5839300

H	3.3135350	3.4041610	-2.7858640
H	4.8783760	-4.6937360	0.3502140
H	5.8298250	-3.3208090	-0.2498590
H	5.1184120	-4.4790760	-1.3920010
H	-4.2877820	-1.7955570	-0.4234380
H	-2.2393810	-2.4275910	-4.1438960
H	-0.1413250	-1.5987440	-3.1414270
H	-5.1294220	-1.9075730	-3.6615090
H	-5.4657890	-2.8849460	-2.2191720
H	-4.5452550	-3.5791870	-3.5725130
H	-4.1952170	-1.3726020	1.8889720
H	-0.4161880	-2.2846800	1.3671000
H	0.2784800	-2.3234370	3.7061650
H	-1.0263610	-1.2125490	4.1797390
H	0.2645470	-0.6345860	3.1174140
H	-2.8216710	-2.9203750	3.1771890
H	-1.4809580	-4.0094750	2.7731050
H	-2.6024050	-3.4840140	1.4986040
H	-3.1565810	-0.8176070	4.0494510
H	-4.5403970	0.2732990	3.8456630
H	-2.8797240	0.8659010	3.5437040
H	-5.5327470	0.7075250	1.6502680
H	-4.6393740	0.4157260	0.1413140
H	-4.0670340	1.6458970	1.2843570
H	-3.1872130	4.8916790	-2.3262010
H	-1.7448280	4.2365390	-3.1459330
H	-1.5580960	5.4467270	-1.8571480
H	-2.0474970	4.8397430	0.5441660
H	-3.6661310	4.2241850	0.1195370
H	-2.4887850	3.1943450	1.0005360
H	-3.9231940	2.5039640	-1.7590780
H	-2.8065020	1.4074090	-0.9073890

H	-2.4744020	1.8644740	-2.5816920
H	0.0395570	3.8758460	-0.6534650
H	0.3898020	2.1509120	3.5525140
H	0.1212130	3.0946820	2.0475310
H	-0.1682620	2.6298750	-1.8920020

Sc^{III} (L_1)

1

Symbol	X	Y	Z
C	-0.8205020	-3.7827950	-0.4273540
C	-1.0417790	-2.3938510	-0.1829820
C	-2.3898600	-1.9392030	-0.3272160
C	-1.8554400	-4.6673540	-0.6742650
C	-3.1821770	-4.2265790	-0.7022220
C	-3.4199310	-2.8660790	-0.5391130
N	-0.0218910	-1.5335030	0.1267070
P	-2.6598160	-0.1361880	-0.2128620
C	-3.5812500	0.3071230	-1.8152390
C	-3.9148720	1.8025680	-1.7554980
C	-2.5489440	0.0580070	-2.9284600
C	-3.7158520	0.1362520	1.3389780
C	-5.2007550	-0.2128890	1.2264770
C	-3.5547550	1.6095090	1.7443100
C	3.5362060	-2.2463160	0.9348440
C	2.4144410	-1.6467290	0.3448680
C	1.1118840	-2.0638720	0.7314500
C	3.4189650	-3.2000730	1.9387540
C	2.1406970	-3.5563490	2.3785780
C	1.0178000	-3.0012350	1.7889410

P	2.4833670	-0.2390620	-0.8256160
C	4.0462010	0.7705620	-0.4311150
C	2.6368560	-1.0566820	-2.5355650
C	2.9277860	0.0121340	-3.5968050
C	3.6810520	-2.1756020	-2.6041300
C	3.7968530	2.1572770	-1.0490750
C	4.0785010	0.9623010	1.0933480
Sc	0.0068020	0.7843240	0.0829950
C	0.3157040	1.3701340	1.9479350
C	0.6171840	1.3663780	3.4390960
C	-0.6378770	1.7003830	4.2732810
C	1.7011170	2.3993480	3.8112500
C	1.1114370	-0.0312700	3.8419710
C	-0.0994580	2.6257780	-1.4127880
C	-0.1845760	4.1243180	-1.0276180
C	1.0665590	4.5541620	-0.2486570
C	-0.2929470	5.0191860	-2.2785150
C	-1.4171580	4.3898990	-0.1496910
H	0.2023840	-4.1618940	-0.4016430
H	-1.6229530	-5.7226610	-0.8456470
H	-4.4507550	-2.5122250	-0.5702540
H	-4.7428960	2.0099040	-1.0600020
H	-4.2263850	2.1574850	-2.7527460
H	-3.0444550	2.3958170	-1.4447620
H	-1.6378110	0.6552640	-2.7823930
H	-2.9796560	0.3398090	-3.9047380
H	-2.2654480	-1.0048550	-2.9805220
H	-5.6776610	-0.0990890	2.2156710
H	-5.3660820	-1.2520840	0.9055060
H	-5.7322440	0.4574510	0.5342450
H	-3.9709000	2.3016130	0.9973260
H	-2.4882570	1.8467690	1.8866450

H	-4.0797240	1.7917600	2.6981460
H	4.5332670	-1.9546380	0.6032140
H	2.0206140	-4.2714170	3.1970030
H	0.0243710	-3.2823600	2.1434720
H	2.8432550	-0.4362560	-4.6015060
H	3.9416400	0.4290520	-3.5124430
H	2.2070410	0.8432580	-3.5429840
H	4.7041850	-1.8135490	-2.4381840
H	3.6560780	-2.6438420	-3.6038460
H	3.4735870	-2.9640220	-1.8652400
H	3.7074410	2.1350390	-2.1441610
H	4.6341170	2.8310710	-0.7977160
H	2.8763190	2.5949540	-0.6423150
H	4.7619480	1.7922160	1.3411800
H	4.4265390	0.0695870	1.6296400
H	3.0724120	1.2165510	1.4657740
H	-0.4326290	1.7010160	5.3616520
H	-1.4394480	0.9718230	4.0796440
H	-1.0274900	2.6972930	4.0057580
H	1.3794560	3.4166110	3.5309540
H	1.9214820	2.4034240	4.8963540
H	2.6426110	2.1938560	3.2799090
H	1.3545930	-0.0956140	4.9193850
H	2.0097750	-0.3074710	3.2666590
H	0.3429530	-0.7880920	3.6182490
H	0.0655460	2.4178090	1.6112530
H	0.8245220	2.4935570	-2.0231960
H	-0.9143460	2.4390440	-2.1459150
H	0.9784870	5.5916330	0.1205870
H	1.9610770	4.5123900	-0.8914740
H	1.2395410	3.8918010	0.6144120
H	-0.3288300	6.0956310	-2.0257010

H	-1.2032390	4.7813410	-2.8550260
H	0.5703080	4.8608410	-2.9468200
H	-1.3915200	3.7758720	0.7633080
H	-2.3487650	4.1510050	-0.6878340
H	-1.4804800	5.4509120	0.1521730
C	-3.0546540	-0.7332140	2.4204780
H	-3.2328980	-1.8058140	2.2537020
H	-1.9659940	-0.5565410	2.4479670
H	-3.4683790	-0.4665610	3.4078740
C	-4.8517770	-0.4735580	-2.1760380
H	-5.3089040	-0.0120080	-3.0688050
H	-4.6321120	-1.5177810	-2.4351350
H	-5.6094160	-0.4631160	-1.3815260
C	5.3953650	0.2363920	-0.9246150
H	5.4582390	0.2042330	-2.0220420
H	6.1961180	0.9117610	-0.5766820
H	5.6321970	-0.7663980	-0.5417380
C	1.2575960	-1.6625510	-2.8402330
H	0.9899750	-2.4611660	-2.1353900
H	0.4599870	-0.9054450	-2.8035620
H	1.2659670	-2.0974110	-3.8546880
H	-4.0079400	-4.9226760	-0.8631770
H	4.3108530	-3.6450570	2.3852780

1-2-TS

Symbol	X	Y	Z

C	-0.8940780	-4.0224150	0.0448220
C	-1.0585310	-2.6058690	0.1544110
C	-2.3961370	-2.1174180	-0.0410140
C	-1.9606940	-4.8888200	-0.1079030
C	-3.2718400	-4.4061550	-0.1751790
C	-3.4567480	-3.0283170	-0.1549720
N	-0.0084430	-1.7628610	0.3727050
P	-2.6297280	-0.3042410	-0.1461710
C	-3.5135150	-0.0128720	-1.8074580
C	-3.9380430	1.4605150	-1.8881030
C	-2.4328860	-0.2881760	-2.8684160
C	-3.6907620	0.2000390	1.3397570
C	-5.1512290	-0.2544140	1.3242610
C	-3.6153450	1.7301090	1.4657290
C	3.5839510	-2.3951430	1.0919350
C	2.4357390	-1.8275890	0.5205880
C	1.1476500	-2.2480180	0.9637790
C	3.5163170	-3.3147900	2.1313810
C	2.2581820	-3.6612640	2.6332320
C	1.1080360	-3.1392000	2.0659910
P	2.4882290	-0.4574050	-0.6962920
C	3.9930860	0.6355530	-0.2910320
C	2.7061880	-1.3337100	-2.3665920
C	2.9243330	-0.2879860	-3.4675940
C	3.8234100	-2.3810980	-2.3978670
C	3.6615370	2.0043950	-0.9134230
C	4.0024920	0.8320860	1.2330550
Sc	0.0293140	0.5634140	0.1363380
C	0.3060050	1.7180790	1.6033470
C	0.5453360	2.5550310	2.8540310
C	-0.7761150	3.0842800	3.4505690
C	1.4604930	3.7695140	2.5936240

C	1.2193700	1.6870180	3.9384220
C	-0.1070870	2.5503420	-1.2940870
C	-0.2294460	4.0793280	-1.4242410
C	1.1325690	4.7262270	-1.1454710
C	-0.6871640	4.4637350	-2.8389190
C	-1.2586170	4.5825540	-0.4041830
H	0.1141430	-4.4355190	0.0989440
H	-1.7662990	-5.9635860	-0.1751010
H	-4.4735950	-2.6439610	-0.2319720
H	-4.8345200	1.6580610	-1.2817420
H	-4.1866100	1.7171850	-2.9322460
H	-3.1455500	2.1476510	-1.5565920
H	-1.5810940	0.4035580	-2.7824780
H	-2.8589590	-0.1611290	-3.8786520
H	-2.0514650	-1.3189970	-2.7953000
H	-5.6401420	0.0541050	2.2648380
H	-5.2511680	-1.3475620	1.2529650
H	-5.7199400	0.2049430	0.5011010
H	-4.1925770	2.2479660	0.6865570
H	-2.5650090	2.0640630	1.4187160
H	-4.0304570	2.0395430	2.4404170
H	4.5646680	-2.0994400	0.7159630
H	2.1745750	-4.3442880	3.4832840
H	0.1329780	-3.4133870	2.4728760
H	2.8774880	-0.7746630	-4.4569340
H	3.9050360	0.2041870	-3.3932790
H	2.1476330	0.4932870	-3.4455560
H	4.8194440	-1.9430520	-2.2512930
H	3.8290510	-2.8894210	-3.3782100
H	3.6714840	-3.1519940	-1.6277050
H	3.5543260	1.9661020	-2.0078930
H	4.4673740	2.7226600	-0.6831000

H	2.7263390	2.3909620	-0.4813170
H	4.6379790	1.6987000	1.4829660
H	4.3948990	-0.0400960	1.7730000
H	2.9761680	1.0343410	1.5888660
H	-0.6170050	3.6794920	4.3722180
H	-1.4473880	2.2469040	3.6979510
H	-1.3050840	3.7244700	2.7256140
H	0.9687940	4.4904540	1.9208230
H	1.7175390	4.3124740	3.5249510
H	2.4004060	3.4503060	2.1144900
H	1.3653420	2.2374040	4.8901480
H	2.2048980	1.3326690	3.5984450
H	0.6087190	0.7934380	4.1437750
H	0.1422670	2.4358170	0.0077280
H	0.6701010	2.1823720	-1.9941150
H	-1.0654640	2.1069040	-1.6227900
H	1.0688250	5.8275620	-1.1713830
H	1.8774380	4.4147550	-1.8966080
H	1.5091680	4.4275390	-0.1566760
H	-0.7731110	5.5586020	-2.9567250
H	-1.6717380	4.0227910	-3.0686660
H	0.0251830	4.0980100	-3.5974330
H	-0.9731710	4.2719510	0.6119410
H	-2.2538830	4.1526430	-0.6062950
H	-1.3533660	5.6815290	-0.4297160
C	-2.9573390	-0.4078810	2.5471960
H	-2.9961490	-1.5078320	2.5422560
H	-1.8990110	-0.0889950	2.5592670
H	-3.4306720	-0.0517480	3.4782590
C	-4.7293690	-0.8870950	-2.1402010
H	-5.1860770	-0.5242540	-3.0776930
H	-4.4474850	-1.9358190	-2.3032490

H	-5.5070490	-0.8521750	-1.3644380
C	5.3705030	0.1745510	-0.7771340
H	5.4392370	0.1373560	-1.8744920
H	6.1322750	0.8949330	-0.4320950
H	5.6593570	-0.8109570	-0.3842350
C	1.3677130	-2.0432170	-2.6315100
H	1.1496260	-2.8108170	-1.8750090
H	0.5232480	-1.3376410	-2.6362550
H	1.4012950	-2.5392400	-3.6169680
H	-4.1236800	-5.0835380	-0.2635350
H	4.4286100	-3.7360180	2.5593180

A

Symbol	X	Y	Z
C	-0.7590640	-3.6300240	0.2426390
C	-0.9959100	-2.2262930	0.2051690
C	-2.3334010	-1.8143470	-0.1033390
C	-1.7673300	-4.5572440	0.0344560
C	-3.0773100	-4.1436260	-0.2223740
C	-3.3335140	-2.7764610	-0.2855830
N	0.0061890	-1.3061870	0.3878490
P	-2.5806620	-0.0018210	-0.2579050
C	-3.7534640	0.3103970	-1.7206020
C	-3.6036800	1.7974620	-2.0949680
C	-3.2068050	-0.5329750	-2.8843660
C	-3.3548980	0.4963730	1.3967430
C	-4.5250240	-0.3813180	1.8472030
C	-3.7678080	1.9691850	1.3105030
C	3.4489810	-1.4484510	1.7711550
C	2.3883760	-1.1239200	0.9134450

C	1.0732610	-1.5990330	1.2083070
C	3.2687670	-2.1925430	2.9311580
C	1.9768650	-2.6214540	3.2518800
C	0.9121250	-2.3311020	2.4167290
P	2.5900640	-0.0840150	-0.5873060
C	3.8798950	1.2618770	-0.2137560
C	3.2260040	-1.3180710	-1.8839420
C	3.6419770	-0.5632330	-3.1527850
C	4.3718540	-2.2151850	-1.4059060
C	3.6295120	2.3619090	-1.2615500
C	3.4959430	1.8384090	1.1578300
Sc	-0.0266070	0.8646460	-0.5692120
C	-0.0567190	2.6689810	-0.0156880
C	-0.1159800	4.0838380	0.5455250
C	1.1887960	4.8571270	0.2600290
C	-0.3313150	4.0780130	2.0751440
C	-1.2715970	4.8849090	-0.0921070
H	0.2552950	-3.9827720	0.4395460
H	-1.5253010	-5.6237390	0.0680950
H	-4.3542860	-2.4460080	-0.4850120
H	-4.0950230	2.4566870	-1.3666820
H	-4.0738970	1.9797610	-3.0771190
H	-2.5495990	2.1172340	-2.1365090
H	-2.1312020	-0.3522280	-3.0473180
H	-3.7299540	-0.2611760	-3.8168840
H	-3.3405570	-1.6118280	-2.7212690
H	-4.8848310	-0.0340900	2.8314810
H	-4.2226970	-1.4332460	1.9600040
H	-5.3786500	-0.3419180	1.1557330
H	-4.6748210	2.1146050	0.7031910
H	-2.9420750	2.5592320	0.8791750
H	-3.9841640	2.3557010	2.3209590

H	4.4544040	-1.1064710	1.5223820
H	1.7975960	-3.1840640	4.1726660
H	-0.0907110	-2.6675460	2.6879580
H	3.8276480	-1.2829600	-3.9687070
H	4.5668900	0.0146520	-3.0115740
H	2.8561730	0.1299270	-3.4930340
H	5.2724690	-1.6454010	-1.1400070
H	4.6523180	-2.9185500	-2.2096480
H	4.0766300	-2.8132860	-0.5314140
H	3.8905170	2.0397070	-2.2812660
H	4.2488810	3.2446850	-1.0261030
H	2.5668830	2.6648200	-1.2426630
H	4.0398980	2.7846000	1.3192780
H	3.7415610	1.1601110	1.9873500
H	2.4102980	2.0529550	1.1796470
H	1.1596070	5.8972100	0.6442390
H	1.3775690	4.8996110	-0.8252950
H	2.0500890	4.3519130	0.7239520
H	0.4800080	3.5212480	2.5716770
H	-0.3655030	5.0977680	2.5114870
H	-1.2753860	3.5705390	2.3303540
H	-1.3243240	5.9303290	0.2760710
H	-2.2400980	4.4041080	0.1187110
H	-1.1524440	4.9091150	-1.1876500
C	5.3604100	0.8690130	-0.2358250
H	5.9697230	1.7524310	0.0219520
H	5.6134920	0.0836670	0.4906190
H	5.6903790	0.5332980	-1.2302940
C	2.0175860	-2.2084200	-2.2192750
H	1.6505970	-2.7548730	-1.3383180
H	1.1741100	-1.6237370	-2.6190440
H	2.3039910	-2.9520730	-2.9829960

C	-2.2088420	0.3751240	2.4153950
H	-1.8445610	-0.6601670	2.5044390
H	-1.3608960	1.0223130	2.1286450
H	-2.5686780	0.6917360	3.4097410
C	-5.2441910	0.0118240	-1.5277910
H	-5.7843000	0.2303860	-2.4658430
H	-5.4481620	-1.0380330	-1.2759310
H	-5.6888680	0.6449460	-0.7466230
H	4.1165760	-2.4234020	3.5795950
H	-3.8792130	-4.8688990	-0.3757770

5-6-TS

Symbol	X	Y	Z
C	-0.7268310	-3.7075270	-0.0604080
C	-0.9755670	-2.3101020	0.0540900
C	-2.3171640	-1.8787260	-0.2039750
C	-1.7276300	-4.6148770	-0.3680170
C	-3.0416040	-4.1865750	-0.5766740
C	-3.3099400	-2.8229720	-0.4891670
N	0.0174990	-1.4059460	0.3372620
P	-2.5741240	-0.0630850	-0.1505590
C	-3.8362020	0.3949740	-1.4972090
C	-3.6762760	1.9091730	-1.7346760
C	-3.3981400	-0.3417890	-2.7740920
C	-3.2725270	0.2373630	1.5878280
C	-4.4138460	-0.7036980	1.9824780
C	-3.7037180	1.7033190	1.6985240
C	3.4280520	-1.6418160	1.7851600
C	2.3840180	-1.2526560	0.9349820
C	1.0688450	-1.7707620	1.1465040
C	3.2338650	-2.5035000	2.8586100

C	1.9432540	-2.9868420	3.0958920
C	0.8941460	-2.6310210	2.2659850
P	2.6009660	-0.0867010	-0.4644110
C	3.8777330	1.2292990	0.0341400
C	3.2693630	-1.2073380	-1.8478770
C	3.7399210	-0.3583450	-3.0351440
C	4.3881380	-2.1582200	-1.4108330
C	3.6336460	2.4025260	-0.9327270
C	3.4730920	1.7053320	1.4379580
Sc	-0.0252010	0.8554300	-0.4630450
C	-0.0888410	2.5877450	0.2849450
C	-0.1766500	4.0147700	0.8086360
C	1.1159630	4.8068770	0.5185550
C	-0.4022450	4.0277110	2.3347580
C	-1.3460840	4.7757730	0.1489150
H	0.2911810	-4.0701520	0.0957870
H	-1.4769520	-5.6766890	-0.4509950
H	-4.3347640	-2.4815950	-0.6468320
H	-4.0535010	2.4998730	-0.8891750
H	-4.2499020	2.2051950	-2.6303830
H	-2.6257500	2.2020320	-1.8739440
H	-2.3204110	-0.2215570	-2.9676330
H	-3.9376960	0.0713670	-3.6433880
H	-3.6046820	-1.4200970	-2.7285290
H	-4.7227560	-0.4871090	3.0200750
H	-4.1005220	-1.7578700	1.9461470
H	-5.3030810	-0.5882490	1.3474220
H	-4.6269710	1.9149030	1.1372210
H	-2.8958380	2.3560640	1.3299030
H	-3.8989330	1.9536260	2.7553270
H	4.4327470	-1.2607820	1.5974560
H	1.7516620	-3.6448650	3.9485090

H	-0.1075500	-3.0123820	2.4749210
H	3.9515140	-1.0150920	-3.8965030
H	4.6621890	0.1987540	-2.8156930
H	2.9724310	0.3641770	-3.3534300
H	5.2820580	-1.6245070	-1.0594420
H	4.6946060	-2.7876410	-2.2648780
H	4.0549180	-2.8302730	-0.6066930
H	3.8982920	2.1578860	-1.9723910
H	4.2500660	3.2666150	-0.6299550
H	2.5723070	2.7018740	-0.8937150
H	4.0267850	2.6285740	1.6809580
H	3.6943180	0.9628680	2.2179600
H	2.3909020	1.9334860	1.4593330
H	1.0681080	5.8509530	0.8891770
H	1.3123330	4.8433080	-0.5660300
H	1.9823000	4.3201560	0.9929230
H	0.4148210	3.4917240	2.8442960
H	-0.4548460	5.0541110	2.7517690
H	-1.3392340	3.5089710	2.5917280
H	-1.4354610	5.8207840	0.5095770
H	-2.3021510	4.2660100	0.3486810
H	-1.2167510	4.8071670	-0.9458860
C	0.0728520	1.9601790	-2.7182410
H	-0.0245730	2.4325220	-1.5670050
H	-0.7128200	1.2829680	-3.0986070
H	1.0591740	1.5271330	-2.9562870
H	-0.0193110	2.9032850	-3.2782720
C	5.3609700	0.8470830	0.0068200
H	5.9620860	1.7159780	0.3263620
H	5.6083550	0.0208440	0.6885520
H	5.7063980	0.5721600	-1.0006850
C	2.0606630	-2.0436850	-2.3005290

H	1.6526150	-2.6557710	-1.4834280
H	1.2428010	-1.4089680	-2.6772510
H	2.3628270	-2.7248180	-3.1149930
C	-2.0873440	0.0157980	2.5425450
H	-1.7160860	-1.0193610	2.5056610
H	-1.2526530	0.6959760	2.2985090
H	-2.4134110	0.2192380	3.5775250
C	-5.3174640	0.1053120	-1.2287010
H	-5.9125860	0.4020360	-2.1103470
H	-5.5246890	-0.9578020	-1.0425640
H	-5.6994610	0.6824200	-0.3745420
H	4.0686780	-2.7840140	3.5043500
H	-3.8376950	-4.8977440	-0.8073250

6

Symbol	X	Y	Z
C	-0.7894830	-3.5897500	0.3173850
C	-1.0260890	-2.1888570	0.2540680
C	-2.3607610	-1.7830350	-0.0565550
C	-1.7985990	-4.5196120	0.1240920
C	-3.1076370	-4.1093960	-0.1413560
C	-3.3638490	-2.7440210	-0.2263640
N	-0.0213500	-1.2617940	0.4312530
P	-2.6045080	0.0241710	-0.2027300
C	-3.8490580	0.3552150	-1.5940090
C	-3.6562160	1.8298120	-1.9956900
C	-3.4109010	-0.5221520	-2.7783830
C	-3.2969250	0.5114720	1.4914030
C	-4.4641140	-0.3479210	1.9838770

C	-3.6819870	1.9931370	1.4479520
C	3.3849420	-1.4696350	1.9029980
C	2.3511870	-1.1363760	1.0181700
C	1.0172740	-1.5607940	1.2926940
C	3.1556500	-2.1772840	3.0778750
C	1.8450370	-2.5605480	3.3774730
C	0.8068850	-2.2605160	2.5110610
P	2.5792310	-0.1645390	-0.5168720
C	3.9056970	1.1555650	-0.1965860
C	3.1784030	-1.4622520	-1.7659800
C	3.5494940	-0.7643370	-3.0804420
C	4.3390020	-2.3369130	-1.2814370
C	3.6688260	2.2369550	-1.2676740
C	3.5719020	1.7776540	1.1674880
Sc	-0.0237860	0.8028050	-0.6847810
C	0.0275240	2.6706260	0.0185030
C	0.0586460	3.9983490	0.7662600
C	1.3605360	4.7825420	0.4930050
C	-0.0410410	3.7406820	2.2780710
C	-1.1146400	4.9116100	0.3529880
H	0.2229110	-3.9413600	0.5240270
H	-1.5574400	-5.5853940	0.1763030
H	-4.3839050	-2.4137690	-0.4311860
H	-3.9805510	2.5242010	-1.2085880
H	-4.2543990	2.0448600	-2.8981370
H	-2.6030760	2.0553560	-2.2155700
H	-2.3308110	-0.4265070	-2.9670220
H	-3.9413190	-0.1975490	-3.6898230
H	-3.6363450	-1.5856340	-2.6173340
H	-4.7520760	-0.0260310	2.9999810
H	-4.1865430	-1.4110560	2.0414160
H	-5.3568660	-0.2604920	1.3496200

H	-4.6014670	2.1717730	0.8698720
H	-2.8600260	2.5769650	1.0045890
H	-3.8609170	2.3642900	2.4715570
H	4.4060110	-1.1683590	1.6627270
H	1.6291180	-3.0979260	4.3053720
H	-0.2086450	-2.5707940	2.7652740
H	3.7331650	-1.5224120	-3.8612080
H	4.4665100	-0.1630020	-2.9906520
H	2.7363400	-0.1121340	-3.4335760
H	5.2622600	-1.7674560	-1.1113000
H	4.5627670	-3.1026790	-2.0448490
H	4.0848840	-2.8662490	-0.3510760
H	3.8533670	1.8695580	-2.2878840
H	4.3493430	3.0880790	-1.0910720
H	2.6319750	2.6056410	-1.2110520
H	4.1499540	2.7090180	1.2937350
H	3.8146840	1.1139610	2.0089350
H	2.4985740	2.0291000	1.2105700
H	1.3805660	5.7574550	1.0178690
H	1.4727870	4.9813080	-0.5859650
H	2.2417670	4.2094080	0.8150980
H	0.7640980	3.0667570	2.6131500
H	0.0296060	4.6755910	2.8646860
H	-0.9982580	3.2567350	2.5279180
H	-1.1035040	5.8802260	0.8896730
H	-2.0815360	4.4260260	0.5543480
H	-1.0754130	5.1271540	-0.7278700
C	-0.0259900	0.8082210	-3.0055000
H	0.0247280	2.9010980	-1.0943910
H	-0.0042140	-0.1793950	-3.5116730
H	0.9116360	1.3243650	-3.3050880
H	-0.8371400	1.3831800	-3.4962920

C	5.3742300	0.7221820	-0.2331510
H	6.0117620	1.5908520	0.0064280
H	5.6120180	-0.0628330	0.4989360
H	5.6792970	0.3663500	-1.2281760
C	1.9629960	-2.3693650	-2.0195200
H	1.6685750	-2.9179170	-1.1134610
H	1.0903200	-1.7970280	-2.3654860
H	2.2168460	-3.1142030	-2.7935080
C	-2.1169050	0.3489910	2.4631860
H	-1.7913910	-0.6984560	2.5423920
H	-1.2540900	0.9550930	2.1468050
H	-2.4222390	0.6838380	3.4696020
C	-5.3338360	0.1219290	-1.2954390
H	-5.9221530	0.3226880	-2.2079010
H	-5.5568700	-0.9096130	-0.9883280
H	-5.7095660	0.7997640	-0.5152700
H	3.9810220	-2.4193890	3.7507780
H	-3.9092260	-4.8369420	-0.2850580

6’

Symbol	X	Y	Z
C	-0.7927190	-3.5325120	0.4110830
C	-1.0323530	-2.1299840	0.3342040
C	-2.3584440	-1.7336820	-0.0287600
C	-1.7864570	-4.4687230	0.1793950
C	-3.0859500	-4.0682060	-0.1425800
C	-3.3446820	-2.7047850	-0.2403370
N	-0.0392470	-1.2019610	0.5390170
P	-2.6422420	0.0681660	-0.1874470

C	-3.7869620	0.3533740	-1.6776620
C	-3.6281160	1.8372250	-2.0578680
C	-3.2159700	-0.4953380	-2.8250970
C	-3.5095540	0.5230170	1.4372620
C	-4.6669340	-0.4004730	1.8287310
C	-3.9844010	1.9782640	1.3548320
C	3.4247620	-1.6063610	1.8281520
C	2.3656770	-1.2050490	1.0027390
C	1.0283110	-1.5445400	1.3547700
C	3.2130640	-2.2940570	3.0180440
C	1.8988330	-2.5794570	3.3999880
C	0.8359450	-2.2130390	2.5902120
P	2.5575440	-0.2071610	-0.5213450
C	3.9862120	1.0171790	-0.2513740
C	2.9990500	-1.4879740	-1.8520220
C	3.3679250	-0.7613700	-3.1517650
C	4.1044230	-2.4714280	-1.4563200
C	3.7227210	2.1527430	-1.2578020
C	3.8238820	1.6023030	1.1599680
Sc	0.0072920	0.8675970	-0.5360690
C	0.2351620	2.4869110	0.6286520
C	0.2351390	3.9911500	0.8686380
C	-0.2216960	4.6957110	-0.4193140
C	1.6330880	4.5309240	1.2366590
C	-0.7267300	4.3885470	2.0072390
C	0.0619970	1.2670420	-2.8176460
H	0.2125710	-3.8801950	0.6544280
H	-1.5396830	-5.5326320	0.2438810
H	-4.3570390	-2.3831520	-0.4888840
H	-4.0394430	2.5124050	-1.2951660
H	-4.1665190	2.0350080	-3.0009850
H	-2.5721630	2.1014470	-2.2073480

H	-2.1322170	-0.3369790	-2.9368790
H	-3.6964170	-0.1997120	-3.7734100
H	-3.3920240	-1.5702640	-2.6768820
H	-5.0875840	-0.0680370	2.7938380
H	-4.3282090	-1.4389420	1.9581450
H	-5.4861390	-0.3964650	1.0968300
H	-4.8642810	2.0968720	0.7042310
H	-3.1772480	2.6286030	0.9841080
H	-4.2698970	2.3335980	2.3595760
H	4.4485230	-1.3741550	1.5293060
H	1.7011870	-3.0927320	4.3452910
H	-0.1851610	-2.4457760	2.8998440
H	3.4555620	-1.4955140	-3.9710670
H	4.3320150	-0.2366940	-3.0822810
H	2.5949750	-0.0321450	-3.4383850
H	5.0730870	-1.9804120	-1.2933630
H	4.2439390	-3.2126180	-2.2629060
H	3.8431740	-3.0254260	-0.5421780
H	3.8015170	1.8191700	-2.3028760
H	4.4607520	2.9599770	-1.1088870
H	2.7128900	2.5667040	-1.1020220
H	4.4404810	2.5129630	1.2481280
H	4.1421160	0.9037800	1.9462350
H	2.7747820	1.8831260	1.3401670
H	-0.2214770	5.7977690	-0.3207960
H	-1.2402710	4.3735980	-0.6910000
H	0.4380780	4.4243530	-1.2599290
H	2.3583980	4.3076320	0.4390470
H	1.6308400	5.6265220	1.3982350
H	2.0038230	4.0594690	2.1622130
H	-0.7501330	5.4825980	2.1785640
H	-0.4263210	3.9091080	2.9544190

H	-1.7527280	4.0571510	1.7848000
H	-0.6929130	1.9782440	-3.2105240
H	1.0363520	1.7896770	-2.9369850
H	0.0821850	0.4195520	-3.5347680
H	0.4267890	1.9356390	1.5936750
C	-2.4145910	0.4297870	2.5114810
H	-2.0121720	-0.5912480	2.5981570
H	-1.5817450	1.1103140	2.2761840
H	-2.8360950	0.7108360	3.4921890
C	-5.2815230	0.0566000	-1.5118740
H	-5.7521700	0.7062030	-0.7599880
H	-5.7945950	0.2491500	-2.4703410
H	-5.4902580	-0.9876360	-1.2413450
C	5.4161640	0.4966750	-0.4292670
H	5.6183550	0.1569690	-1.4552620
H	6.1241940	1.3162210	-0.2159580
H	5.6610290	-0.3262120	0.2581080
C	1.7034490	-2.2798270	-2.0944130
H	1.3875570	-2.8357450	-1.2002340
H	0.8733260	-1.6218800	-2.3928710
H	1.8660070	-3.0131220	-2.9031710
H	-3.8766960	-4.8003590	-0.3188630
H	4.0568450	-2.5913560	3.6444650

6’-7’-TS

Symbol	X	Y	Z
C	-0.6770130	-3.7123750	-0.2509560
C	-0.9351450	-2.3289880	-0.0338300
C	-2.2862210	-1.8999410	-0.2210940

C	-1.6802610	-4.6127860	-0.5672060
C	-3.0079150	-4.1906460	-0.6851010
C	-3.2842740	-2.8382870	-0.5125000
N	0.0623390	-1.4364230	0.2862000
P	-2.5622900	-0.0958950	-0.1019360
C	-3.8912730	0.3884530	-1.3736700
C	-3.7364150	1.9054170	-1.5921200
C	-3.5302610	-0.3153060	-2.6924560
C	-3.1875630	0.1570740	1.6719100
C	-4.2901010	-0.8188370	2.0928600
C	-3.6432430	1.6106030	1.8437880
C	3.5005730	-1.8731670	1.6279100
C	2.4425730	-1.3749020	0.8572060
C	1.1202980	-1.8793090	1.0521070
C	3.3095300	-2.8188640	2.6301270
C	2.0097600	-3.2736410	2.8716480
C	0.9487350	-2.8186350	2.1063160
P	2.5957760	-0.0437780	-0.3895950
C	3.9185210	1.1839640	0.2066230
C	3.1631040	-0.9465320	-1.9597300
C	3.5097240	0.0876240	-3.0374150
C	4.3217910	-1.9230320	-1.7377600
C	3.6556610	2.4827760	-0.5776600
C	3.6417890	1.4637300	1.6931940
Sc	-0.0005170	0.8512660	-0.5246000
C	-0.0700760	2.6640830	0.3688160
C	-0.2574260	4.1411670	0.6823440
C	-1.4173610	4.6889980	-0.1639110
C	1.0056260	4.9684710	0.3637410
C	-0.5923230	4.3404150	2.1719170
C	0.0893510	1.7681630	-2.3461280
H	0.3509760	-4.0697910	-0.1659370

H	-1.4216050	-5.6633220	-0.7294870
H	-4.3171700	-2.4994210	-0.6092300
H	-4.0003520	2.4841430	-0.6965030
H	-4.4086050	2.2281580	-2.4060580
H	-2.7037220	2.1686450	-1.8673360
H	-2.4709870	-0.1694360	-2.9544330
H	-4.1330520	0.1152750	-3.5100720
H	-3.7242390	-1.3963180	-2.6630460
H	-4.5592440	-0.6347180	3.1476180
H	-3.9535190	-1.8635910	2.0154510
H	-5.2075600	-0.7105790	1.4986450
H	-4.5907040	1.8215700	1.3256770
H	-2.8674510	2.2988510	1.4714740
H	-3.8025160	1.8220980	2.9151570
H	4.5114950	-1.5084010	1.4371490
H	1.8202360	-3.9932410	3.6734460
H	-0.0571030	-3.1895450	2.3139460
H	3.6341810	-0.4228920	-4.0080730
H	4.4507580	0.6177540	-2.8264190
H	2.6918540	0.8215320	-3.1365420
H	5.2426890	-1.4263520	-1.4032740
H	4.5532190	-2.4374570	-2.6868590
H	4.0620460	-2.6962720	-0.9991190
H	3.8892400	2.3795050	-1.6459540
H	4.2865000	3.2936700	-0.1735460
H	2.6009720	2.7837750	-0.4991430
H	4.2369900	2.3350330	2.0154270
H	3.9073540	0.6162480	2.3400600
H	2.5830300	1.7042800	1.8732630
H	-1.5725550	5.7701300	0.0046370
H	-2.3558640	4.1662310	0.0766020
H	-1.2234390	4.5325270	-1.2373560

H	1.2744850	4.8722530	-0.7005040
H	0.8703000	6.0442850	0.5850210
H	1.8643000	4.6119330	0.9566130
H	-0.7361070	5.4073690	2.4275250
H	0.2192960	3.9495050	2.8086470
H	-1.5106570	3.7970850	2.4423640
H	0.0099280	2.5540110	-1.1012440
H	0.2763960	2.5455430	-3.1049870
H	-0.7656720	1.1313960	-2.7051420
H	0.7725840	2.2378530	0.9917940
C	5.3898960	0.7908190	0.0324590
H	5.6641760	0.6725860	-1.0253810
H	6.0275850	1.5934690	0.4422910
H	5.6579190	-0.1353230	0.5607310
C	1.9362600	-1.7362270	-2.4428340
H	1.5957970	-2.4738560	-1.7019320
H	1.1014370	-1.0544330	-2.6686840
H	2.1951680	-2.2804780	-3.3676340
C	-1.9616230	-0.0626550	2.5730320
H	-1.5504050	-1.0780900	2.4762880
H	-1.1660850	0.6605180	2.3319380
H	-2.2539830	0.0850800	3.6271810
C	-5.3539690	0.0917290	-1.0230850
H	-5.6898290	0.6529010	-0.1394690
H	-5.9967720	0.4030010	-1.8649050
H	-5.5505710	-0.9750140	-0.8457090
H	4.1536730	-3.1849000	3.2184710
H	-3.8069830	-4.8974560	-0.9185150

6’-7”-TS

Symbol	X	Y	Z
C	-0.7335720	-3.6749130	-0.1610910
C	-0.9778200	-2.2808700	-0.0063200
C	-2.3081510	-1.8338450	-0.2808790
C	-1.7277750	-4.5628390	-0.5364890
C	-3.0307130	-4.1173280	-0.7771870
C	-3.2953020	-2.7580450	-0.6439820
N	0.0147120	-1.3924730	0.3289090
P	-2.5960050	-0.0346780	-0.1128820
C	-3.8268780	0.5139080	-1.4499070
C	-3.6404710	2.0370870	-1.5802630
C	-3.3542380	-0.1238550	-2.7661670
C	-3.3516230	0.1180000	1.6251000
C	-4.4883080	-0.8681830	1.9131660
C	-3.8282130	1.5574680	1.8551580
C	3.4076860	-1.7338450	1.7949850
C	2.3771030	-1.3053090	0.9484770
C	1.0506750	-1.8005690	1.1382310
C	3.1872070	-2.6153340	2.8476950
C	1.8864380	-3.0810320	3.0621410
C	0.8503600	-2.6856800	2.2327440
P	2.6001040	-0.1209490	-0.4309870
C	3.9222530	1.1507400	0.0571510
C	3.1990060	-1.2173140	-1.8621320
C	3.6710950	-0.3426240	-3.0300750
C	4.2946260	-2.2136260	-1.4698420
C	3.7429370	2.3277080	-0.9199960
C	3.5438230	1.6474910	1.4618600
Sc	0.0016460	0.8552280	-0.5038200
C	-0.0331020	2.6584060	0.4129440
C	-0.0449170	4.1509200	0.7102180

C	-1.3526910	4.8039570	0.2170300
C	1.1321430	4.8317830	-0.0030780
C	0.0824830	4.4007570	2.2228160
C	-0.0723030	1.7864760	-2.3270180
H	0.2764650	-4.0506470	0.0128650
H	-1.4800120	-5.6222620	-0.6511030
H	-4.3106230	-2.4023450	-0.8270830
H	-3.9400820	2.5808210	-0.6731410
H	-4.2610640	2.4119510	-2.4127340
H	-2.5878440	2.2744510	-1.7958870
H	-2.2758100	0.0561540	-2.9165430
H	-3.8936580	0.3441420	-3.6076580
H	-3.5437360	-1.2061850	-2.8030420
H	-4.8341480	-0.7352490	2.9533480
H	-4.1531330	-1.9105190	1.8077980
H	-5.3573960	-0.7202840	1.2582910
H	-4.7300500	1.7989600	1.2735140
H	-3.0457320	2.2881670	1.5984700
H	-4.0795330	1.6965720	2.9205390
H	4.4214510	-1.3706700	1.6218870
H	1.6761710	-3.7575440	3.8956020
H	-0.1589990	-3.0567770	2.4219370
H	3.8185890	-0.9745990	-3.9224330
H	4.6290800	0.1560220	-2.8226350
H	2.9277670	0.4275580	-3.2885790
H	5.2123620	-1.7202580	-1.1205960
H	4.5643090	-2.8286780	-2.3462270
H	3.9530380	-2.8969200	-0.6784530
H	4.0756360	2.0834580	-1.9383440
H	4.3448630	3.1858330	-0.5746490
H	2.6900950	2.6412900	-0.9745340
H	4.1507140	2.5359030	1.7076460

H	3.7237720	0.8905380	2.2389220
H	2.4795450	1.9387790	1.4959930
H	-1.3872360	5.8910300	0.4221190
H	-2.2254670	4.3422590	0.7080400
H	-1.4714740	4.6566160	-0.8684220
H	1.1042220	4.6206280	-1.0846040
H	1.1132890	5.9283570	0.1332210
H	2.0918580	4.4569650	0.3844800
H	0.0858540	5.4793890	2.4693190
H	1.0117970	3.9554860	2.6121880
H	-0.7567150	3.9349600	2.7669030
H	-0.0565260	2.5659810	-1.0545260
H	-0.3310440	2.5674820	-3.0611830
H	0.8680880	1.2812720	-2.6727630
H	-0.8845040	2.1730620	0.9736570
C	-2.1987660	-0.1756380	2.6005480
H	-1.8082420	-1.1961560	2.4760640
H	-1.3583430	0.5236910	2.4726650
H	-2.5647660	-0.0813980	3.6376030
C	-5.3112830	0.2078840	-1.2225190
H	-5.7138120	0.7257280	-0.3397350
H	-5.8887070	0.5631830	-2.0936520
H	-5.5219930	-0.8656850	-1.1145080
C	5.3898610	0.7090580	0.0334360
H	5.7215350	0.4139980	-0.9725900
H	6.0244520	1.5569180	0.3448900
H	5.6060800	-0.1211450	0.7202670
C	1.9588280	-2.0014360	-2.3221320
H	1.5555310	-2.6407480	-1.5242720
H	1.1513540	-1.3298180	-2.6547140
H	2.2308190	-2.6538790	-3.1699300
H	-3.8207030	-4.8127340	-1.0684330

H	4.0111820	-2.9273260	3.4929040

7’

Symbol	X	Y	Z
C	-0.6018380	-3.6013750	-0.2183310
C	-0.8987050	-2.2245490	-0.0161600
C	-2.2463320	-1.8225850	-0.2583230
C	-1.5671480	-4.5206070	-0.5919970
C	-2.8927330	-4.1225530	-0.7858190
C	-3.2055660	-2.7784620	-0.6172970
N	0.0721860	-1.3124520	0.3456510
P	-2.6197060	-0.0405610	-0.0671610
C	-3.9098350	0.4300520	-1.3884960
C	-3.7561650	1.9510050	-1.5621990
C	-3.4900740	-0.2283040	-2.7124750
C	-3.3748500	0.0382850	1.6776230
C	-4.4457700	-1.0160860	1.9742200
C	-3.9297600	1.4444020	1.9302680
C	3.4979950	-1.8088730	1.7037680
C	2.4555900	-1.3022840	0.9175700
C	1.1214210	-1.7574300	1.1310160
C	3.2764540	-2.7169920	2.7343170
C	1.9647740	-3.1278250	2.9865480
C	0.9185180	-2.6621090	2.2062700
P	2.6445100	-0.0298760	-0.3862830
C	4.0268840	1.1581270	0.1649330
C	3.1718390	-1.0159430	-1.9207540
C	3.5074030	-0.0280260	-3.0443800
C	4.3211590	-1.9996530	-1.6828510

C	3.8186680	2.4525790	-0.6435280
C	3.7990950	1.4766720	1.6528900
Sc	-0.0106000	0.8391290	-0.5525110
C	0.1946800	2.7191790	0.8197690
C	-0.1986900	4.2012000	0.6443240
C	0.1594530	4.6910640	-0.7659410
C	0.5304590	5.0953280	1.6667330
C	-1.7080770	4.3755360	0.8457430
C	-0.1393680	1.1403050	-2.5164830
H	0.4255080	-3.9405980	-0.0761810
H	-1.2794680	-5.5655230	-0.7404070
H	-4.2385930	-2.4599590	-0.7655280
H	-4.0281600	2.5074650	-0.6527770
H	-4.4141340	2.3020050	-2.3759160
H	-2.7129170	2.1882070	-1.8297580
H	-2.4220180	-0.0408870	-2.9116860
H	-4.0746770	0.2201290	-3.5344470
H	-3.6710950	-1.3120730	-2.7236100
H	-4.7804790	-0.9121750	3.0214230
H	-4.0532510	-2.0364630	1.8544520
H	-5.3336840	-0.9136400	1.3365050
H	-4.8437340	1.6485800	1.3533950
H	-3.1884700	2.2168650	1.6832360
H	-4.1865200	1.5556320	2.9978710
H	4.5203150	-1.4845700	1.5020820
H	1.7541240	-3.8205670	3.8063330
H	-0.0979070	-3.0015720	2.4160420
H	3.6122590	-0.5769030	-3.9959590
H	4.4541430	0.5061450	-2.8707510
H	2.6885310	0.7022670	-3.1596590
H	5.2668710	-1.5063740	-1.4221980
H	4.4987530	-2.5800130	-2.6050240

H	4.0772660	-2.7186820	-0.8860340
H	3.9718910	2.3011570	-1.7205990
H	4.5390730	3.2182230	-0.3070800
H	2.8052820	2.8552740	-0.5121890
H	4.4008680	2.3585680	1.9315300
H	4.0966430	0.6469100	2.3082700
H	2.7475280	1.7139630	1.8723560
H	-0.0365570	5.7721140	-0.8852360
H	-0.4256450	4.1522040	-1.5263470
H	1.2245170	4.5171930	-0.9906770
H	1.6232450	5.0365230	1.5274090
H	0.2390270	6.1590810	1.5831310
H	0.3132000	4.7673120	2.6972720
H	-2.0255270	5.4219900	0.6887700
H	-2.0037320	4.0907790	1.8694930
H	-2.2661010	3.7422770	0.1401920
H	1.3016100	2.6962850	0.9036420
H	-0.0812670	2.2552270	-2.3739040
H	-0.2260480	0.9563070	-3.6042890
H	-0.1403480	2.3926710	1.8316050
C	-2.1915290	-0.1926810	2.6321760
H	-1.7393070	-1.1851000	2.4911210
H	-1.4009980	0.5592150	2.4910040
H	-2.5426620	-0.1280880	3.6767020
C	-5.3774440	0.0999370	-1.0946820
H	-5.7585010	0.6196050	-0.2040710
H	-5.9953650	0.4300510	-1.9476850
H	-5.5596900	-0.9767010	-0.9665400
C	5.4778750	0.7016420	-0.0304840
H	5.7327570	0.5772700	-1.0924790
H	6.1549390	1.4734450	0.3754750
H	5.7110100	-0.2383460	0.4891070

C	1.9263060	-1.8100220	-2.3460300
H	1.6236330	-2.5394520	-1.5815570
H	1.0807400	-1.1322850	-2.5465650
H	2.1542360	-2.3698290	-3.2697730
H	4.1090910	-3.0905470	3.3342710
H	-3.6631060	-4.8422900	-1.0705510

7”

Symbol	X	Y	Z
C	-0.7128700	-3.5973740	0.2798040
C	-0.9638490	-2.1981350	0.2323890
C	-2.3012220	-1.8019080	-0.0734410
C	-1.7119880	-4.5364530	0.0861050
C	-3.0265900	-4.1363210	-0.1683370
C	-3.2947990	-2.7741110	-0.2463380
N	0.0377680	-1.2681300	0.4216330
P	-2.6034420	-0.0023170	-0.2056650
C	-3.9074900	0.2951720	-1.5612750
C	-3.7315340	1.7641700	-1.9892710
C	-3.5315710	-0.5812880	-2.7656300
C	-3.3040720	0.4364850	1.5082780
C	-4.4204970	-0.4800460	2.0181090
C	-3.7756240	1.8963110	1.5066250
C	3.4484900	-1.5288160	1.8750930
C	2.4119270	-1.1735700	1.0041840
C	1.0745630	-1.5846240	1.2809840
C	3.2169520	-2.2433220	3.0466900
C	1.9034740	-2.6115200	3.3511890
C	0.8621030	-2.2920030	2.4939160
P	2.5975790	-0.1568050	-0.5054360
C	3.8965300	1.1774640	-0.1580320

C	3.1924660	-1.3895680	-1.8150170
C	3.5334180	-0.6249650	-3.0997750
C	4.3684420	-2.2645300	-1.3702640
C	3.6295140	2.2833420	-1.1969500
C	3.5779140	1.7330300	1.2389310
Sc	0.0096740	0.7379010	-0.7955150
C	-0.2374590	2.9897030	-0.3099440
C	-0.0088560	4.1278110	0.7032430
C	-1.0327780	5.2635120	0.5041130
C	1.3923350	4.7265110	0.5232150
C	-0.1424980	3.6149290	2.1445650
C	-0.0882650	0.6567780	-2.8076880
H	0.3048250	-3.9396410	0.4754040
H	-1.4594420	-5.5999810	0.1282210
H	-4.3180790	-2.4531370	-0.4472330
H	-3.9501090	2.4734380	-1.1773670
H	-4.4216530	1.9890460	-2.8208950
H	-2.7021320	1.9401340	-2.3382820
H	-2.4667840	-0.4441730	-3.0183800
H	-4.1350950	-0.2710790	-3.6361820
H	-3.7220970	-1.6490480	-2.5881380
H	-4.6937750	-0.1878770	3.0474470
H	-4.0967500	-1.5308090	2.0501070
H	-5.3330320	-0.4213500	1.4103820
H	-4.7004320	2.0375600	0.9283850
H	-3.0088220	2.5694980	1.0945020
H	-3.9844690	2.2211000	2.5405340
H	4.4716250	-1.2372060	1.6293660
H	1.6870100	-3.1541530	4.2759440
H	-0.1548400	-2.5958980	2.7506950
H	3.6816070	-1.3419960	-3.9253670
H	4.4619230	-0.0409570	-3.0058810

H	2.7044630	0.0488460	-3.3735160
H	5.2830810	-1.6882010	-1.1759780
H	4.6033290	-2.9907550	-2.1679140
H	4.1252640	-2.8399260	-0.4641840
H	3.8675960	1.9568470	-2.2187150
H	4.2539960	3.1654920	-0.9722120
H	2.5746980	2.5961130	-1.1919550
H	4.1059360	2.6903880	1.3849410
H	3.8901610	1.0510430	2.0415660
H	2.5000010	1.9214050	1.3600590
H	-0.8726600	6.1043070	1.2047400
H	-2.0616510	4.8952130	0.6542320
H	-0.9752890	5.6628540	-0.5218580
H	1.5310310	5.1030230	-0.5039410
H	1.5663450	5.5689220	1.2163880
H	2.1726350	3.9765660	0.7066960
H	0.0384460	4.4147730	2.8854080
H	0.5734000	2.8010580	2.3410620
H	-1.1530570	3.2183840	2.3312230
H	0.0118700	3.3792530	-1.3225470
H	-0.1501310	1.6620220	-3.2902260
H	-0.1193230	-0.0961250	-3.6239640
H	-1.3396690	2.8256090	-0.3799180
C	-2.1049960	0.3096680	2.4624320
H	-1.7403120	-0.7257910	2.5225210
H	-1.2619480	0.9419660	2.1487130
H	-2.4037150	0.6218560	3.4781550
C	-5.3753620	0.0582560	-1.1873950
H	-5.7238300	0.7389480	-0.3973620
H	-6.0043440	0.2478210	-2.0744000
H	-5.5797200	-0.9725390	-0.8642700
C	5.3751200	0.7822870	-0.2229720

H	5.6746480	0.4631820	-1.2315100
H	5.9961320	1.6575690	0.0362960
H	5.6362030	-0.0202710	0.4817490
C	1.9831370	-2.2969950	-2.0949720
H	1.6964150	-2.8813880	-1.2085040
H	1.1125230	-1.7098750	-2.4233100
H	2.2437420	-3.0110440	-2.8953240
H	-3.8226000	-4.8698460	-0.3124500
H	4.0428570	-2.5026270	3.7125790

Sc^{III} (L_3)

1

Symbol	X	Y	Z
C	0.0263510	-3.7692550	-1.0861060
C	-0.6444650	-2.7164690	-0.4147380
C	-0.6460750	-4.7523280	-1.8028390
C	-2.0388190	-4.7508340	-1.8591460
C	-2.7276170	-3.7733770	-1.1430950
N	0.0420500	-1.7136140	0.2580380
P	-2.8337290	-0.0665350	0.1044460
C	-3.9084390	0.0560340	-1.4660040
C	-4.3942080	1.4971990	-1.6731630
C	-2.9835750	-0.3190630	-2.6408690
C	-3.7644100	0.6615690	1.5987290
C	-5.2658930	0.3692290	1.6597310
C	-3.5101140	2.1750620	1.5975580

C	3.2916780	-2.7229330	1.8404900
C	1.0205230	-2.1917420	1.1580150
C	2.8527770	-3.1892660	3.0752690
C	1.4878490	-3.1657700	3.3592280
C	0.5986450	-2.6758650	2.4116860
P	2.5841120	-0.0194380	-0.9299300
C	4.0915180	0.9254530	-0.2725330
C	2.6464050	-0.1512010	-2.8263680
C	1.2850110	-0.7518120	-3.2246800
C	2.7595220	1.2535510	-3.4315570
C	3.7337110	2.4151240	-0.2794980
C	4.2479100	0.4943490	1.1930890
Sc	-0.0021130	0.5748840	0.1186200
C	0.4278560	1.4263790	1.8524370
C	0.7880120	1.7759710	3.2900750
C	-0.3654660	2.5129780	4.0037170
C	2.0223330	2.6982460	3.3653290
C	1.0909430	0.4841260	4.0647500
C	-0.4893620	2.2319300	-1.5368170
C	-0.4672640	3.7789100	-1.5094430
C	0.9499100	4.3076340	-1.2662330
C	-0.9747720	4.3579660	-2.8457720
C	-1.3733790	4.3070840	-0.3906220
H	1.1154210	-3.8096090	-1.0315510
H	-0.0731400	-5.5283700	-2.3185300
H	-3.8209560	-3.8113970	-1.1092150
H	-5.1655070	1.7872340	-0.9450560
H	-4.8417740	1.5906560	-2.6775450
H	-3.5711460	2.2243200	-1.6127030
H	-2.1536060	0.3927310	-2.7502550
H	-3.5622310	-0.3025560	-3.5810340
H	-2.5594900	-1.3276130	-2.5315620

H	-5.6723750	0.7505180	2.6126970
H	-5.4821640	-0.7102340	1.6250780
H	-5.8262670	0.8612300	0.8516960
H	-3.9419960	2.6778920	0.7215270
H	-2.4269240	2.3722080	1.6173310
H	-3.9591570	2.6303600	2.4972330
H	4.3600800	-2.7568900	1.6055390
H	1.1164340	-3.5199300	4.3243530
H	-0.4710400	-2.6416910	2.6295790
H	1.2276850	-0.8460740	-4.3226780
H	0.4487340	-0.1148450	-2.9027120
H	1.1215050	-1.7551520	-2.8018070
H	2.0248830	1.9429230	-2.9918350
H	2.5671080	1.2081740	-4.5172520
H	3.7626110	1.6843830	-3.2955880
H	3.5739560	2.8109020	-1.2917020
H	4.5481710	2.9986260	0.1839770
H	2.8154900	2.5758510	0.3057830
H	4.9109650	1.2058950	1.7137410
H	4.6982810	-0.5027950	1.2871720
H	3.2739040	0.4915370	1.7071000
H	-0.1089670	2.7800900	5.0476010
H	-1.2742720	1.8942600	4.0289310
H	-0.6167580	3.4466080	3.4730340
H	1.8354700	3.6387550	2.8199660
H	2.2836240	2.9625310	4.4083540
H	2.9017460	2.2224750	2.9082760
H	1.3810250	0.6844960	5.1133610
H	1.9047060	-0.0810390	3.5822790
H	0.2076780	-0.1739650	4.0751900
H	0.2926530	2.3849870	1.2647810
H	0.0159380	1.9211170	-2.4776290

H	-1.5467230	1.9600900	-1.7243460
H	0.9836990	5.4120500	-1.2847620
H	1.6475840	3.9425340	-2.0377300
H	1.3216750	3.9697290	-0.2884540
H	-0.9928250	5.4640670	-2.8484930
H	-1.9978620	4.0054070	-3.0608910
H	-0.3337450	4.0320130	-3.6825620
H	-1.0447150	3.9304290	0.5896310
H	-2.4160590	3.9823900	-0.5392460
H	-1.3715930	5.4112480	-0.3529670
C	2.9367000	-1.7806430	-0.4632980
H	4.0148120	-1.9924260	-0.5384090
H	2.4482490	-2.3731770	-1.2494500
C	-2.8498140	-1.8730990	0.4984620
H	-2.3533690	-1.9132880	1.4784560
H	-3.8770250	-2.2480520	0.6271380
C	-2.0693030	-2.7754550	-0.4176280
C	2.4068130	-2.2203570	0.8772150
C	-3.0993570	0.0888220	2.8627120
H	-3.3807430	-0.9584230	3.0490990
H	-2.0006160	0.1684530	2.8026280
H	-3.4317990	0.6731270	3.7372580
C	-5.1159790	-0.8898700	-1.4901820
H	-5.8062620	-0.7287900	-0.6521720
H	-5.6862370	-0.7279520	-2.4215220
H	-4.8088740	-1.9440990	-1.4839180
C	5.4115370	0.6826010	-1.0066740
H	5.4060260	1.0852790	-2.0300430
H	6.2285760	1.1888970	-0.4637850
H	5.6704680	-0.3871030	-1.0559070
C	3.7557700	-1.0442330	-3.3975720
H	4.7622970	-0.6800280	-3.1550440

H	3.6772910	-2.0850000	-3.0498900
H	3.6728600	-1.0676360	-4.4982080
H	-2.5820020	-5.5174950	-2.4163720
H	3.5701090	-3.5684010	3.8070640

1-2-TS

Symbol	X	Y	Z
C	-0.7815770	4.0175730	-0.0573750
C	0.0856340	2.9447360	0.2962760
C	-0.3488030	5.1096310	-0.8017650
C	0.9849090	5.2257410	-1.1900340
C	1.8837360	4.2583150	-0.7331620
N	-0.3183780	1.7971150	0.9162040
P	2.7432090	0.5556570	0.1812740
C	3.7661420	0.7096290	-1.4173760
C	4.4665690	-0.6137410	-1.7523240
C	2.7411060	1.0045010	-2.5280740
C	3.7921350	-0.1862310	1.5819390
C	5.2139680	0.3671050	1.7026100
C	3.8093840	-1.7087440	1.3810540
C	-3.8701930	1.8116330	2.1265970
C	-1.4760570	1.8469010	1.7186450
C	-3.6766150	1.9646660	3.4951200
C	-2.3741770	2.0438620	3.9884590
C	-1.3017700	1.9868380	3.1084310
P	-2.3966340	0.0165980	-1.0276620
C	-3.7293480	-1.3023400	-0.7798530
C	-2.3259950	0.5740490	-2.8484890
C	-1.0160430	1.3754300	-2.9497980

C	-2.2250220	-0.6313570	-3.7918700
C	-3.1008170	-2.6404790	-1.1875390
C	-3.9799390	-1.3607070	0.7344770
Sc	0.0668780	-0.4246030	0.2752200
C	-0.1899870	-1.8486770	1.4842210
C	-0.4289930	-2.8813110	2.5766450
C	0.8913300	-3.3804560	3.1999430
C	-1.2031540	-4.1157070	2.0728250
C	-1.2537460	-2.2377700	3.7105050
C	0.7361680	-2.1746050	-1.3819350
C	1.0773390	-3.6372290	-1.7290460
C	-0.1895610	-4.4995270	-1.7074520
C	1.7023320	-3.7196160	-3.1310130
C	2.0768020	-4.1833420	-0.7024860
H	-1.8224460	3.9821920	0.2682540
H	-1.0702900	5.8857760	-1.0752030
H	2.9527100	4.3974110	-0.9245640
H	5.3170600	-0.8172450	-1.0858650
H	4.8610650	-0.5666600	-2.7820460
H	3.7785150	-1.4707720	-1.7035650
H	2.0650950	0.1510400	-2.6880850
H	3.2675930	1.1901340	-3.4804860
H	2.1307160	1.8927340	-2.3063210
H	5.6983730	-0.0520980	2.6016110
H	5.2257950	1.4636600	1.8089670
H	5.8445540	0.0970780	0.8424520
H	4.3705390	-2.0163130	0.4883980
H	2.7742940	-2.0806480	1.3023350
H	4.2834890	-2.1934350	2.2517190
H	-4.8898520	1.7454720	1.7342440
H	-2.1939860	2.1486070	5.0615140
H	-0.2755750	2.0398670	3.4773640

H	-0.8849710	1.7598050	-3.9760010
H	-0.1445500	0.7448130	-2.7185080
H	-0.9898250	2.2423600	-2.2706120
H	-1.4268760	-1.3281280	-3.4949840
H	-1.9890920	-0.2784410	-4.8104290
H	-3.1668770	-1.1953950	-3.8528000
H	-2.9125290	-2.7148420	-2.2674390
H	-3.7714170	-3.4722100	-0.9107880
H	-2.1476220	-2.7710610	-0.6503180
H	-4.5559390	-2.2711720	0.9726680
H	-4.5570300	-0.4991030	1.0968530
H	-3.0219510	-1.4026710	1.2816660
H	0.7257520	-4.1026420	4.0245160
H	1.4700380	-2.5332140	3.6005790
H	1.5209470	-3.8776540	2.4439550
H	-0.6091070	-4.6674250	1.3265580
H	-1.4483510	-4.8246730	2.8884760
H	-2.1471420	-3.8122110	1.5923770
H	-1.4372870	-2.9359920	4.5527090
H	-2.2291600	-1.8911600	3.3343890
H	-0.7311230	-1.3514250	4.1026000
H	0.3124010	-2.2700040	-0.1077320
H	0.0070480	-1.7952680	-2.1237930
H	1.6559680	-1.5791620	-1.5152850
H	0.0442880	-5.5582330	-1.9133230
H	-0.9109460	-4.1627260	-2.4694330
H	-0.6848660	-4.4426370	-0.7282740
H	1.9562220	-4.7594750	-3.4047780
H	2.6276320	-3.1224820	-3.1900560
H	1.0094450	-3.3299710	-3.8956750
H	1.6751240	-4.0848200	0.3173050
H	3.0239010	-3.6218540	-0.7382330

H	2.3090260	-5.2457310	-0.8897500
C	-3.0809070	1.5565990	-0.2446330
H	-4.1628050	1.6339010	-0.4355670
H	-2.6046510	2.3763810	-0.8017260
C	2.5073470	2.3082130	0.7300450
H	2.1406020	2.1900630	1.7606170
H	3.4703700	2.8394960	0.7794220
C	1.4808610	3.1544340	0.0176960
C	-2.7987420	1.7327580	1.2262950
C	3.0261160	0.0793340	2.8908580
H	3.0857830	1.1299090	3.2123540
H	1.9646830	-0.2147920	2.7963070
H	3.4708390	-0.5318770	3.6941930
C	4.8044690	1.8378390	-1.4015610
H	5.5226280	1.7455190	-0.5756160
H	5.3798770	1.8203320	-2.3434290
H	4.3326920	2.8272590	-1.3339680
C	-5.0497770	-1.0613640	-1.5126180
H	-4.9447070	-1.1278920	-2.6057190
H	-5.7838790	-1.8281920	-1.2101980
H	-5.4879930	-0.0803820	-1.2674290
C	-3.4919640	1.4634340	-3.3012330
H	-4.4661100	0.9650850	-3.2024780
H	-3.5357830	2.4100630	-2.7439430
H	-3.3619600	1.7248160	-4.3657860
H	1.3325530	6.0769370	-1.7796340
H	-4.5342890	2.0142950	4.1701770

A

Symbol	X	Y	Z
C	0.1256660	-2.9205930	2.1678070
C	-0.5730590	-1.7820000	1.6761820
C	-0.4467050	-4.1875120	2.1957370
C	-1.7624620	-4.3858280	1.7798960
C	-2.5093870	-3.2622680	1.4154220
N	-0.0162710	-0.5475900	1.5200110
P	-2.8410010	0.2308560	-0.3095800
C	-3.8183460	-0.7964010	-1.5728320
C	-4.2545510	0.0812510	-2.7528070
C	-2.8179990	-1.8378180	-2.1022070
C	-3.7962590	1.8043900	0.1730530
C	-5.2891520	1.5993070	0.4422270
C	-3.5743940	2.8434500	-0.9370860
C	3.5440750	-0.1722410	2.6440720
C	1.1532630	-0.2048860	2.2263590
C	3.3734800	0.6279770	3.7681200
C	2.0868540	1.0377280	4.1185860
C	1.0043940	0.6226850	3.3553420
P	2.2351740	-0.7007790	-1.0208270
C	3.7059060	0.3474180	-1.5959460
C	2.0862180	-2.2873380	-2.0595480
C	0.7450330	-2.9131630	-1.6405350
C	1.9969940	-1.9164270	-3.5453950
C	3.2185890	1.2058820	-2.7730950
C	4.0095350	1.2943920	-0.4254500
Sc	-0.0914590	0.6727890	-0.3992160
C	0.3492820	2.5005690	-0.4952580
C	0.7753250	3.9375240	-0.2208430
C	-0.4280320	4.9020860	-0.2860330
C	1.8196090	4.4338720	-1.2429800
C	1.3995630	4.0574780	1.1871630

H	1.1455990	-2.7907050	2.5346110
H	0.1490990	-5.0320670	2.5553660
H	-3.5746280	-3.3847640	1.1938290
H	-5.0758850	0.7621150	-2.4869540
H	-4.6165720	-0.5631820	-3.5724500
H	-3.4209780	0.6840600	-3.1457200
H	-1.9839130	-1.3511170	-2.6321740
H	-3.3192190	-2.5090740	-2.8211560
H	-2.4024970	-2.4622110	-1.2972980
H	-5.7280220	2.5401420	0.8172060
H	-5.4748870	0.8275850	1.2065240
H	-5.8456670	1.3266510	-0.4670990
H	-4.0672180	2.5760640	-1.8818070
H	-2.4911880	2.9620140	-1.1187800
H	-3.9840800	3.8167570	-0.6162090
H	4.5535950	-0.4860420	2.3611050
H	1.9278010	1.6833460	4.9861020
H	-0.0101300	0.9387130	3.6058940
H	0.5554200	-3.8274460	-2.2286610
H	-0.0925510	-2.2251700	-1.8205410
H	0.7117800	-3.1961060	-0.5771970
H	1.2484790	-1.1284540	-3.7272030
H	1.6985790	-2.8020380	-4.1323530
H	2.9597870	-1.5677080	-3.9461950
H	2.9905480	0.6106130	-3.6694750
H	4.0031120	1.9318260	-3.0491400
H	2.3120790	1.7638700	-2.4732420
H	4.6532690	2.1176690	-0.7782430
H	4.5394030	0.7876880	0.3926230
H	3.0723510	1.7285810	-0.0340030
H	-0.1440820	5.9541370	-0.0769880
H	-1.1978580	4.6078230	0.4448230

H	-0.8924150	4.8676870	-1.2852880
H	1.4145280	4.3606970	-2.2657250
H	2.1255950	5.4856560	-1.0671680
H	2.7265200	3.8105070	-1.2029110
H	1.7137280	5.0926510	1.4355140
H	2.2817210	3.4025660	1.2756200
H	0.6788510	3.7222080	1.9502450
C	2.7444210	-1.4238770	0.6221700
H	3.8099430	-1.7018560	0.5959090
H	2.1777600	-2.3645810	0.6903170
C	-2.8831920	-0.7797710	1.2525720
H	-2.5485280	-0.0592850	2.0136820
H	-3.9166400	-1.0662910	1.5025310
C	-1.9694180	-1.9769360	1.3822670
C	2.4665180	-0.5837120	1.8464900
C	-3.1170700	2.3594530	1.4383900
H	-3.3456950	1.7713130	2.3397380
H	-2.0211170	2.4166000	1.3022470
H	-3.4835060	3.3840100	1.6181860
C	-5.0354200	-1.5314930	-1.0007300
H	-5.7639740	-0.8521830	-0.5382510
H	-5.5549490	-2.0708440	-1.8117850
H	-4.7451960	-2.2806890	-0.2513520
C	4.9715430	-0.4263020	-1.9690420
H	4.8455650	-1.0262240	-2.8828360
H	5.7920700	0.2864980	-2.1615400
H	5.3072310	-1.0933200	-1.1584800
C	3.1961520	-3.3251500	-1.8515940
H	4.1885830	-2.9479030	-2.1297880
H	3.2441640	-3.6764620	-0.8104160
H	2.9937540	-4.2108020	-2.4789540
H	-2.2183550	-5.3781980	1.7932660

H	4.2389270	0.9375940	4.3587760

5-6-TS

Symbol	X	Y	Z
C	0.0578070	-3.1971590	1.9127010
C	-0.6195800	-2.0031340	1.5345910
C	-0.5554360	-4.4443740	1.8726740
C	-1.8926080	-4.5699010	1.4988600
C	-2.6122510	-3.4005480	1.2397870
N	-0.0278810	-0.7772490	1.4615940
P	-2.8363970	0.2279300	-0.2124790
C	-3.8152770	-0.6961230	-1.5564210
C	-4.2928970	0.2611140	-2.6556820
C	-2.8014980	-1.6686650	-2.1836750
C	-3.7916900	1.7609380	0.3747220
C	-5.2914020	1.5489090	0.5981180
C	-3.5390620	2.8747300	-0.6543010
C	3.4848830	-0.5199040	2.7562580
C	1.1088030	-0.5171330	2.2533530
C	3.2681840	0.1328350	3.9646680
C	1.9642130	0.4768390	4.3218330
C	0.9126570	0.1530740	3.4755270
P	2.2985690	-0.6428760	-0.9797580
C	3.7902690	0.4427660	-1.4054250
C	2.1773990	-2.1176820	-2.1809200
C	0.7913410	-2.7259470	-1.9057390
C	2.2056990	-1.6316830	-3.6356710
C	3.3366770	1.4037860	-2.5144810
C	4.0647140	1.2857640	-0.1507720

Sc	-0.0529280	0.7082510	-0.3086350
C	0.4479120	2.4961470	-0.0184950
C	0.8315060	3.9431960	0.2584770
C	-0.3938980	4.8788940	0.1895660
C	1.8712950	4.4708270	-0.7521860
C	1.4361600	4.0660470	1.6726850
C	-0.3416770	1.4302930	-2.7650200
H	1.0943280	-3.1264320	2.2474260
H	0.0242730	-5.3315890	2.1455130
H	-3.6890280	-3.4745240	1.0565440
H	-5.1128740	0.9115550	-2.3196050
H	-4.6720940	-0.3278020	-3.5085960
H	-3.4795630	0.8981240	-3.0333970
H	-1.9924090	-1.1195390	-2.6892380
H	-3.3014310	-2.2972930	-2.9411200
H	-2.3529730	-2.3403010	-1.4362550
H	-5.7341840	2.4647400	1.0268140
H	-5.4929150	0.7289480	1.3058370
H	-5.8322210	1.3377900	-0.3363080
H	-4.0038720	2.6735850	-1.6290690
H	-2.4537460	3.0101270	-0.7968850
H	-3.9566200	3.8254120	-0.2803290
H	4.5061740	-0.7908200	2.4704350
H	1.7674110	1.0041110	5.2589680
H	-0.1123400	0.4286070	3.7316630
H	0.6229610	-3.5986540	-2.5599720
H	-0.0094980	-2.0006660	-2.1092560
H	0.6689100	-3.0685340	-0.8662010
H	1.4924690	-0.8127700	-3.8150780
H	1.9280190	-2.4632120	-4.3060420
H	3.2039000	-1.2870940	-3.9418920
H	3.1164460	0.8944780	-3.4635290

H	4.1322690	2.1426440	-2.7124700
H	2.4366000	1.9476500	-2.1841250
H	4.7544600	2.1075740	-0.4088350
H	4.5344320	0.6990730	0.6506550
H	3.1240150	1.7243520	0.2297330
H	-0.1404660	5.9327420	0.4251220
H	-1.1704380	4.5494880	0.8979460
H	-0.8386560	4.8578000	-0.8193730
H	1.4807150	4.4006590	-1.7814110
H	2.1472980	5.5286740	-0.5671810
H	2.7944930	3.8727970	-0.7077810
H	1.7427610	5.1031730	1.9205640
H	2.3162880	3.4106850	1.7738300
H	0.7055790	3.7322970	2.4267930
H	-0.0006290	2.0492790	-1.6980650
H	-0.0412780	0.3870150	-2.9714190
H	-1.4223320	1.5213260	-2.9574130
C	2.7535020	-1.5428570	0.5844590
H	3.8162760	-1.8311500	0.5617000
H	2.1751110	-2.4774550	0.5332150
C	-2.8989970	-0.8993910	1.2603690
H	-2.5199000	-0.2551250	2.0681090
H	-3.9386820	-1.1658670	1.5068810
C	-2.0283080	-2.1343900	1.2720780
C	2.4370120	-0.8407080	1.8823190
C	-3.1408040	2.2139640	1.6943840
H	-3.3913380	1.5534080	2.5381120
H	-2.0413720	2.2805760	1.5969060
H	-3.5147780	3.2202680	1.9480940
C	-5.0133440	-1.4960400	-1.0323640
H	-5.7423260	-0.8691050	-0.5008300
H	-5.5394270	-1.9688930	-1.8799330

H	-4.7021670	-2.3037690	-0.3570640
C	5.0644910	-0.3007120	-1.8096360
H	4.9615260	-0.8294410	-2.7688220
H	5.8899470	0.4226820	-1.9271290
H	5.3805820	-1.0286730	-1.0450540
C	3.2424090	-3.2064620	-1.9931150
H	4.2639310	-2.8289310	-2.1343550
H	3.1871290	-3.6760340	-1.0004790
H	3.0829110	-4.0083350	-2.7350060
H	-2.3828500	-5.5453550	1.4646680
H	4.1098830	0.3756630	4.6177110
H	0.1861380	2.0578370	-3.5011550

6

Symbol	X	Y	Z
C	-0.2191550	-2.9546470	2.1321430
C	-0.8114310	-1.7712770	1.6217570
C	-0.9599110	-4.0878450	2.4462870
C	-2.3457460	-4.0866160	2.2938030
C	-2.9616840	-2.9101570	1.8686280
N	-0.0558550	-0.6751900	1.2480000
P	-2.7947500	0.2897570	-0.3823450
C	-3.7675260	-0.8752370	-1.5257580
C	-4.0715370	-0.1734260	-2.8559880
C	-2.8168380	-2.0491240	-1.8207610
C	-3.7018650	1.9491710	-0.1955020
C	-5.2269040	1.8676040	-0.0908070
C	-3.2845010	2.8310940	-1.3832770
C	3.2829010	-0.1938210	2.8929200
C	0.9753530	-0.3161790	2.1435540

C	2.9163610	0.5263640	4.0252830
C	1.5695680	0.8238390	4.2305560
C	0.6264060	0.4065030	3.3006850
P	2.4155440	-0.7523260	-0.9005350
C	3.9278800	0.2856910	-1.3638380
C	2.3330910	-2.3360490	-1.9467790
C	2.3775450	-1.9740800	-3.4369020
C	3.4077780	-3.3856480	-1.6371320
C	3.5026330	1.1935800	-2.5282030
C	4.2085810	1.1797190	-0.1474060
Sc	-0.0075600	0.5742010	-0.6530570
C	0.5001910	2.4749430	-0.3313550
C	0.9337350	3.8016520	0.2778830
C	-0.1838320	4.8621120	0.1852640
C	2.1698020	4.3875840	-0.4363490
C	1.2785510	3.5911430	1.7595090
C	-0.3200700	0.4402540	-2.9771530
H	0.8627610	-2.9703580	2.2793620
H	-0.4463770	-4.9801070	2.8162750
H	-4.0546260	-2.8703700	1.8222130
H	-4.8299570	0.6164190	-2.7537440
H	-4.4666730	-0.9124570	-3.5741760
H	-3.1649970	0.2652810	-3.2983140
H	-1.9005350	-1.6922580	-2.3101640
H	-3.3119120	-2.7634950	-2.5015560
H	-2.5336810	-2.5978830	-0.9110660
H	-5.6332130	2.8721730	0.1201800
H	-5.5525350	1.2072320	0.7285490
H	-5.6964330	1.5198130	-1.0223450
H	-3.6167620	2.4315480	-2.3514980
H	-2.1876540	2.9348300	-1.4043120
H	-3.7236660	3.8374320	-1.2692030

H	4.3367320	-0.4449190	2.7376970
H	1.2553970	1.3942730	5.1084670
H	-0.4295670	0.6498500	3.4381120
H	2.1306580	-2.8652830	-4.0392870
H	3.3737460	-1.6335670	-3.7553840
H	1.6450030	-1.1915250	-3.6816690
H	4.4244810	-3.0249640	-1.8414860
H	3.2421820	-4.2750850	-2.2698470
H	3.3708930	-3.7270650	-0.5920090
H	3.2666180	0.6295140	-3.4420140
H	4.3190130	1.8966280	-2.7688520
H	2.6127670	1.7782290	-2.2399750
H	4.9003950	1.9880970	-0.4389230
H	4.6796180	0.6236060	0.6752280
H	3.2774910	1.6387750	0.2216080
H	0.1194450	5.8323400	0.6255540
H	-1.0908590	4.5233090	0.7070640
H	-0.4588780	5.0442510	-0.8673900
H	1.9556730	4.5537550	-1.5056970
H	2.4843380	5.3567280	-0.0025430
H	3.0253860	3.6996800	-0.3761900
H	1.6170540	4.5248080	2.2476050
H	2.0702500	2.8340370	1.8772180
H	0.3988010	3.2192730	2.3084990
H	0.3273110	2.6277450	-1.4394250
H	0.5979560	0.8181980	-3.4741960
H	-0.6121280	-0.4781430	-3.5263730
C	2.7991990	-1.4568080	0.7734250
H	3.8701020	-1.6971960	0.8656210
H	2.2610100	-2.4161100	0.7956160
C	-2.9693520	-0.4632720	1.2992560
H	-2.5091550	0.2920650	1.9540590

H	-4.0272030	-0.5585190	1.5910300
C	-2.2383780	-1.7576590	1.5484940
C	2.3436270	-0.6220130	1.9451650
H	-1.0985530	1.1977460	-3.2110210
C	-3.1470520	2.6087030	1.0782400
H	-3.5443960	2.1507080	1.9965730
H	-2.0439680	2.5597930	1.0875710
H	-3.4396920	3.6720430	1.0903570
C	-5.0631720	-1.4324310	-0.9251980
H	-5.5539970	-2.0906240	-1.6633680
H	-4.8683500	-2.0427080	-0.0327800
H	-5.7835340	-0.6491290	-0.6549750
C	5.1984590	-0.4925200	-1.7115650
H	5.1004220	-1.0696770	-2.6423640
H	6.0332050	0.2153600	-1.8561310
H	5.4951990	-1.1819990	-0.9049190
C	0.9478380	-2.9343200	-1.6419840
H	0.1485110	-2.2359910	-1.9279680
H	0.8065240	-3.8630750	-2.2210780
H	0.8102610	-3.1869310	-0.5789890
H	3.6760590	0.8493650	4.7410800
H	-2.9413740	-4.9697690	2.5357190

6’

Symbol	X	Y	Z
C	0.1697850	-3.0851330	2.0789660
C	-0.5527300	-1.9536600	1.6111640
C	-0.4167970	-4.3346660	2.2421130
C	-1.7742680	-4.5189200	1.9869490

C	-2.5287040	-3.4023530	1.6244910
N	0.0368890	-0.7366390	1.3627520
P	-2.7617270	0.0365280	-0.2694480
C	-3.4430310	-1.1226050	-1.6121300
C	-3.8141070	-0.3199060	-2.8659230
C	-2.2665820	-2.0469270	-1.9741210
C	-3.9609200	1.4770630	0.0433760
C	-5.4456290	1.1040120	0.0516650
C	-3.6726110	2.5469570	-1.0213470
C	3.5020210	-0.2230500	2.7188080
C	1.1348390	-0.3844180	2.1813290
C	3.2226930	0.4751920	3.8896040
C	1.8939970	0.7422630	4.2181070
C	0.8759240	0.3153910	3.3738030
P	2.4449390	-0.5279560	-0.9636180
C	3.9657330	0.5722530	-1.2626690
C	2.3812040	-1.9682630	-2.2050510
C	1.8612700	-1.4451540	-3.5520430
C	3.7046910	-2.7101400	-2.4094890
C	3.9886330	1.0282190	-2.7270150
C	3.7392330	1.8108370	-0.3795480
Sc	-0.1058510	0.7461680	-0.4242920
C	0.0011010	2.5612980	0.3994850
C	0.1573890	4.0744090	0.3368260
C	1.4022210	4.5413520	1.1194430
C	-1.0646020	4.8088630	0.9277150
C	0.3144280	4.5122620	-1.1279900
C	-0.3888250	1.1740470	-2.6897720
H	1.2267340	-2.9665270	2.3228820
H	0.1994580	-5.1730130	2.5805750
H	-3.6129350	-3.5086130	1.5153950
H	-4.7272040	0.2770080	-2.7269130

H	-4.0044440	-1.0168260	-3.7004000
H	-2.9987660	0.3513120	-3.1731610
H	-1.4143930	-1.4642660	-2.3548740
H	-2.5758060	-2.7514520	-2.7660540
H	-1.9298610	-2.6433400	-1.1134080
H	-6.0434820	1.9847400	0.3440670
H	-5.6690980	0.3052720	0.7767260
H	-5.8063130	0.7842090	-0.9368000
H	-3.9172410	2.2160540	-2.0397420
H	-2.6058000	2.8232610	-0.9899590
H	-4.2738720	3.4488730	-0.8123980
H	4.5419540	-0.4447750	2.4598230
H	1.6504580	1.2921960	5.1308110
H	-0.1678270	0.5347070	3.6089170
H	1.7034590	-2.2947800	-4.2384210
H	2.5600620	-0.7549890	-4.0415050
H	0.9052700	-0.9180800	-3.4297760
H	4.4464660	-2.0915700	-2.9359670
H	3.5342690	-3.6092500	-3.0272890
H	4.1462210	-3.0465680	-1.4582590
H	4.3016660	0.2244160	-3.4102060
H	4.7118390	1.8535130	-2.8405640
H	3.0061000	1.4034380	-3.0527360
H	4.5530470	2.5382030	-0.5458550
H	3.7224530	1.5607800	0.6913510
H	2.7743860	2.2916520	-0.6001760
H	1.5370680	5.6405010	1.0855940
H	2.3132510	4.0741470	0.7149120
H	1.3276600	4.2426130	2.1786420
H	-1.2210210	4.5176740	1.9802780
H	-0.9507520	5.9103820	0.8975810
H	-1.9798820	4.5490510	0.3737150

H	0.4326730	5.6082680	-1.2262260
H	-0.5620790	4.2088460	-1.7221890
H	1.1952300	4.0305080	-1.5824490
H	-1.1676570	1.9647100	-2.6980720
H	-0.6681410	0.4631020	-3.4934340
C	2.8338440	-1.4271690	0.6121510
H	3.8807570	-1.7626070	0.6409610
H	2.2124310	-2.3299420	0.5755050
C	-2.8735050	-0.9409200	1.2973840
H	-2.5638570	-0.2089560	2.0595890
H	-3.9152550	-1.2265420	1.5139830
C	-1.9662720	-2.1359080	1.4516700
C	2.4860730	-0.6558550	1.8568980
H	0.5390360	1.6773860	-3.0381800
H	0.0312060	2.2220710	1.4694780
C	1.3294300	-2.9536100	-1.6562380
H	0.4506420	-2.4387800	-1.2396460
H	0.9750030	-3.6070960	-2.4700730
H	1.7303620	-3.6084210	-0.8692780
C	5.3152700	-0.0546520	-0.8964260
H	5.5296150	-0.9771880	-1.4532360
H	6.1229220	0.6623420	-1.1244040
H	5.3854880	-0.2788700	0.1779210
C	-3.5852950	2.0799830	1.4079340
H	-3.9154710	1.4532150	2.2502390
H	-2.4945450	2.2397640	1.4676640
H	-4.0780830	3.0607200	1.5168610
C	-4.6319230	-1.9862410	-1.1775390
H	-4.9532330	-2.6197050	-2.0229320
H	-4.3643150	-2.6625880	-0.3543150
H	-5.5009160	-1.3920030	-0.8649450
H	4.0358130	0.8061900	4.5401070

H	-2.2481550	-5.4951010	2.1116640

6’-7’-TS

Symbol	X	Y	Z
C	0.2104310	-3.0420440	2.2278790
C	-0.5247910	-1.9443410	1.6991810
C	-0.3448280	-4.3070250	2.3851850
C	-1.6808110	-4.5421800	2.0667020
C	-2.4555350	-3.4547110	1.6577430
N	0.0250290	-0.7177530	1.4374620
P	-2.7437130	-0.0177120	-0.2523490
C	-3.3645460	-1.1747620	-1.6225110
C	-3.6425730	-0.3625130	-2.8936570
C	-2.1817260	-2.1096230	-1.9241170
C	-3.9683310	1.4040750	0.0319570
C	-5.4492790	1.0191240	-0.0099100
C	-3.6597740	2.4801860	-1.0205750
C	3.5427850	-0.1927190	2.6360260
C	1.1553400	-0.3281750	2.1833540
C	3.3191190	0.6149770	3.7474500
C	2.0110870	0.9684580	4.0770620
C	0.9544220	0.4994580	3.3044740
P	2.3517150	-0.6196140	-0.9621670
C	3.8622670	0.4797930	-1.3136120
C	2.2586890	-2.0669770	-2.1917840
C	1.6572260	-1.5616930	-3.5125440
C	3.5911450	-2.7723420	-2.4613620
C	3.8704160	0.8887510	-2.7919830
C	3.6379870	1.7524400	-0.4794790

Sc	-0.1298670	0.7643170	-0.4120780
C	0.0330810	2.6965870	0.1871070
C	0.1273510	4.2170980	0.2513730
C	-0.6920630	4.7452020	1.4437150
C	-0.4256440	4.8460090	-1.0369000
C	1.5854510	4.6897200	0.4265260
C	-0.1057460	1.4744880	-2.3951110
H	1.2497860	-2.8852150	2.5219820
H	0.2807690	-5.1187490	2.7689230
H	-3.5300420	-3.5974350	1.5028020
H	-4.5521910	0.2509150	-2.8121520
H	-3.7906440	-1.0535160	-3.7416090
H	-2.7837710	0.2882090	-3.1312820
H	-1.3097320	-1.5248050	-2.2542190
H	-2.4542570	-2.8023570	-2.7394860
H	-1.8975790	-2.7165830	-1.0523310
H	-6.0636420	1.8927370	0.2697790
H	-5.6905300	0.2110470	0.6988920
H	-5.7724200	0.7071240	-1.0136090
H	-3.8991910	2.1590170	-2.0425960
H	-2.5911320	2.7421860	-0.9872180
H	-4.2503340	3.3883840	-0.8076390
H	4.5676020	-0.4686400	2.3698250
H	1.8128030	1.6116880	4.9382900
H	-0.0749060	0.7745800	3.5437470
H	1.4752810	-2.4199030	-4.1821160
H	2.3237340	-0.8686340	-4.0422970
H	0.7035490	-1.0358820	-3.3509470
H	4.2953050	-2.1342820	-3.0151020
H	3.4139930	-3.6713870	-3.0772890
H	4.0812270	-3.1050940	-1.5327220
H	4.1559760	0.0606460	-3.4573920

H	4.6080110	1.6957240	-2.9404800
H	2.8885680	1.2766300	-3.1040290
H	4.4692930	2.4581240	-0.6519910
H	3.5933870	1.5470270	0.5999740
H	2.6972280	2.2480230	-0.7569170
H	-0.6243680	5.8455070	1.5427050
H	-0.3372490	4.2967080	2.3867950
H	-1.7538920	4.4763410	1.3295920
H	-1.4837010	4.5804610	-1.1838950
H	-0.3523550	5.9482080	-1.0115260
H	0.1260890	4.4858960	-1.9199770
H	1.6576850	5.7884090	0.5377470
H	2.1964060	4.4029670	-0.4436960
H	2.0405820	4.2290990	1.3192340
H	0.3543370	2.3433620	-1.1813270
H	-0.7461920	2.2620660	-2.8444840
C	2.7765090	-1.4959180	0.6200000
H	3.8146750	-1.8591440	0.6257020
H	2.1266820	-2.3793970	0.6349360
C	-2.8691660	-1.0075090	1.3072690
H	-2.6075250	-0.2684790	2.0810760
H	-3.9067050	-1.3286970	1.4931980
C	-1.9283910	-2.1731220	1.4940560
C	2.4904180	-0.6591120	1.8379020
H	0.6723630	1.2236660	-3.1451410
H	0.5593170	2.2643430	1.0822080
C	1.2746200	-3.0845830	-1.5807940
H	0.4119520	-2.5999580	-1.1010550
H	0.8846720	-3.7438220	-2.3732220
H	1.7516370	-3.7299600	-0.8289640
C	5.2151000	-0.1324420	-0.9348240
H	5.4173640	-1.0830500	-1.4472990

H	6.0216570	0.5686560	-1.2109070
H	5.2999340	-0.3023020	0.1482140
C	-3.6458300	2.0021760	1.4123590
H	-3.9950530	1.3641790	2.2383590
H	-2.5629570	2.1794380	1.5209450
H	-4.1588590	2.9737180	1.5116870
C	-4.5811380	-2.0267000	-1.2463770
H	-4.8465500	-2.6803320	-2.0956660
H	-4.3685770	-2.6842030	-0.3912360
H	-5.4691890	-1.4276770	-1.0057410
H	4.1606450	0.9701510	4.3470890
H	-2.1266210	-5.5325390	2.1823080

6'-7''-TS

Symbol	X	Y	Z
C	-0.1260440	-3.3386850	1.7529380
C	-0.7615530	-2.1086240	1.4290630
C	-0.8118180	-4.5467950	1.8002490
C	-2.1843030	-4.5929860	1.5650450
C	-2.8484570	-3.3879600	1.3329840
N	-0.0826210	-0.9183660	1.3086960
P	-2.7760120	0.2160540	-0.2372970
C	-3.5049570	-0.7728950	-1.6861450
C	-3.8251820	0.1548920	-2.8643940
C	-2.3743190	-1.7147000	-2.1347740
C	-3.8927180	1.6941480	0.1877910
C	-5.3944370	1.3937330	0.2063340
C	-3.5894230	2.8241220	-0.8078680
C	3.4375900	-0.9907620	2.6161080

C	1.0675220	-0.7716790	2.1170260
C	3.2623350	-0.4061790	3.8669280
C	1.9884810	0.0158070	4.2452820
C	0.9165980	-0.1712040	3.3800830
P	2.3784030	-0.5556000	-1.0214640
C	3.9740930	0.4769100	-1.0451390
C	2.2766840	-1.7009150	-2.5364150
C	1.8001850	-0.8770270	-3.7429200
C	3.5706550	-2.4434880	-2.8802470
C	4.0491180	1.2386570	-2.3751550
C	3.8189430	1.5106300	0.0841930
Sc	-0.0479770	0.7410810	-0.3386940
C	0.3045860	2.5774690	0.4458570
C	0.6223530	4.0586080	0.6000910
C	1.5856180	4.2727260	1.7817400
C	-0.6454850	4.8990860	0.8601240
C	1.2879080	4.5802360	-0.6835250
C	-0.4048790	1.6373470	-2.2018600
H	0.9417400	-3.3338620	1.9777340
H	-0.2607870	-5.4633910	2.0314410
H	-3.9389210	-3.3954080	1.2358090
H	-4.7048540	0.7882490	-2.6773050
H	-4.0501140	-0.4590290	-3.7536470
H	-2.9631770	0.7931940	-3.1084670
H	-1.5059730	-1.1263050	-2.4728840
H	-2.7224970	-2.3370120	-2.9778760
H	-2.0573100	-2.3946200	-1.3297810
H	-5.9423640	2.2784410	0.5750390
H	-5.6446890	0.5550080	0.8749740
H	-5.7867570	1.1649850	-0.7950540
H	-3.8891300	2.5798070	-1.8349420
H	-2.5151230	3.0564920	-0.8185510

H	-4.1378640	3.7341300	-0.5084830
H	4.4364890	-1.3176040	2.3113990
H	1.8285120	0.4898400	5.2171300
H	-0.0880630	0.1499530	3.6638710
H	1.5857140	-1.5558960	-4.5865830
H	2.5553710	-0.1583930	-4.0881140
H	0.8850640	-0.3101510	-3.4988100
H	4.3472950	-1.7654480	-3.2632560
H	3.3704970	-3.1853430	-3.6729420
H	3.9824090	-2.9901810	-2.0169720
H	4.3040000	0.5828300	-3.2207040
H	4.8369110	2.0083670	-2.3106950
H	3.1020210	1.7524660	-2.6026420
H	4.6359070	2.2507320	0.0200920
H	3.8635940	1.0476410	1.0800130
H	2.8484560	2.0301540	0.0232600
H	1.8485480	5.3391240	1.9164700
H	2.5171420	3.7043290	1.6352710
H	1.1325750	3.9152290	2.7217200
H	-1.1640570	4.5513840	1.7691410
H	-0.4179390	5.9740080	0.9936270
H	-1.3522980	4.8054080	0.0204220
H	1.5054810	5.6618640	-0.6192860
H	0.6390230	4.4099860	-1.5575450
H	2.2357100	4.0528410	-0.8760510
H	-0.2929420	2.4662090	-0.8573160
H	-1.1978620	2.1364910	-2.7920560
C	2.6148310	-1.7687330	0.3668770
H	3.6011920	-2.2547240	0.3241070
H	1.8622030	-2.5460630	0.1847410
C	-2.9937210	-0.8867930	1.2362180
H	-2.6384320	-0.2579880	2.0681390

H	-4.0570240	-1.1092450	1.4186270
C	-2.1861480	-2.1598860	1.2758320
C	2.3711280	-1.1655460	1.7249970
H	0.5677650	1.9256550	-2.6680520
H	-0.1027060	2.1684060	1.4123920
C	1.1790720	-2.7333160	-2.2152470
H	0.3009860	-2.2697920	-1.7418120
H	0.8364700	-3.2074260	-3.1494730
H	1.5345270	-3.5374660	-1.5545280
C	5.2699730	-0.3098690	-0.8214610
H	5.4582770	-1.0653750	-1.5960040
H	6.1241660	0.3891280	-0.8299210
H	5.2819360	-0.8125420	0.1566680
C	-3.4648350	2.1849690	1.5818670
H	-3.8007480	1.5141570	2.3866240
H	-2.3714080	2.3009190	1.6446820
H	-3.9146670	3.1739510	1.7716520
C	-4.7398550	-1.6073250	-1.3286610
H	-5.0981500	-2.1316480	-2.2317660
H	-4.5078240	-2.3788130	-0.5826090
H	-5.5741580	-1.0014660	-0.9498250
H	4.1155180	-0.2754340	4.5369260
H	-2.7348820	-5.5355510	1.6022240

7’

Symbol	X	Y	Z
C	0.2294160	-2.7934480	2.4584110
C	-0.5015140	-1.7345150	1.8450140
C	-0.3324190	-4.0356110	2.7268960

C	-1.6747620	-4.2865880	2.4479820
C	-2.4451020	-3.2288540	1.9618560
N	0.0632310	-0.5438120	1.4742640
P	-2.7148280	0.0110990	-0.2410850
C	-3.3625260	-1.2603940	-1.4893020
C	-3.7021670	-0.5632770	-2.8125450
C	-2.1780110	-2.2036160	-1.7431900
C	-3.9471610	1.4485060	-0.0776080
C	-5.4158870	1.0417980	0.0763200
C	-3.7892800	2.3659550	-1.3027450
C	3.6375080	-0.0582560	2.5283790
C	1.2333360	-0.1526140	2.1603010
C	3.4742160	0.8149710	3.5997550
C	2.1861500	1.2062210	3.9633670
C	1.0938090	0.7234580	3.2526880
P	2.2463030	-0.7584140	-0.9732090
C	3.7364640	0.2810970	-1.5242390
C	2.0729530	-2.3089070	-2.0599310
C	1.3959890	-1.9213060	-3.3840140
C	3.3856790	-3.0450780	-2.3467200
C	3.6453490	0.5545840	-3.0309040
C	3.5630770	1.6235460	-0.7982810
Sc	-0.0780390	0.7953830	-0.5357780
C	0.2961460	2.7812330	0.5899370
C	0.3004280	4.2601230	0.1474770
C	0.6264590	5.1882070	1.3341560
C	-1.0685880	4.6637120	-0.4145220
C	1.3526670	4.4911460	-0.9458010
C	-0.2359710	1.0881440	-2.5054450
H	1.2725330	-2.6220490	2.7289360
H	0.2932050	-4.8167770	3.1697300
H	-3.5236670	-3.3721960	1.8372990

H	-4.6504700	-0.0082580	-2.7595300
H	-3.8139130	-1.3231500	-3.6051560
H	-2.8930880	0.1252440	-3.1074080
H	-1.3209350	-1.6395450	-2.1397550
H	-2.4579040	-2.9649980	-2.4919780
H	-1.8726350	-2.7321080	-0.8289100
H	-6.0251150	1.9390300	0.2832960
H	-5.5727540	0.3408600	0.9112170
H	-5.8177210	0.5848740	-0.8394100
H	-4.3104750	1.9698150	-2.1837290
H	-2.7338420	2.4912710	-1.5834600
H	-4.2181250	3.3588170	-1.0856220
H	4.6449870	-0.3758250	2.2422180
H	2.0316700	1.8926030	4.7998510
H	0.0801580	1.0294430	3.5194990
H	1.1235470	-2.8359420	-3.9385830
H	2.0537130	-1.3269520	-4.0310190
H	0.4907670	-1.3206440	-3.2159980
H	4.0571800	-2.4633360	-2.9948520
H	3.1685880	-3.9920420	-2.8716870
H	3.9301810	-3.3017220	-1.4242080
H	3.9013650	-0.3294510	-3.6339340
H	4.3611950	1.3505390	-3.2993400
H	2.6335750	0.8974340	-3.3025540
H	4.3850450	2.3070880	-1.0745740
H	3.5692360	1.5192290	0.2967490
H	2.6178040	2.0979490	-1.0944070
H	0.6629120	6.2538110	1.0392040
H	1.6022600	4.9264070	1.7761280
H	-0.1311990	5.0865680	2.1297230
H	-1.8696050	4.4961960	0.3234530
H	-1.0949450	5.7321080	-0.6955860

H	-1.3090200	4.0736260	-1.3113150
H	1.3306290	5.5319680	-1.3159140
H	1.1795590	3.8148900	-1.7986040
H	2.3682330	4.2994140	-0.5625660
H	1.3019500	2.5587790	1.0116430
H	-0.3178710	1.0057970	-3.6062780
C	2.7763720	-1.4921720	0.6486830
H	3.8150050	-1.8538950	0.6381380
H	2.1328670	-2.3706420	0.7825260
C	-2.8454900	-0.8202300	1.4071850
H	-2.5677540	-0.0150310	2.1065250
H	-3.8821560	-1.1151550	1.6352560
C	-1.9103100	-1.9702980	1.6828120
C	2.5469410	-0.5474430	1.7974470
H	-0.3639560	2.1798310	-2.2463160
H	-0.3637070	2.6936940	1.4832520
C	1.1345340	-3.2639730	-1.2949910
H	0.3148950	-2.7344240	-0.7898250
H	0.6826300	-3.9836580	-1.9967920
H	1.6667830	-3.8489540	-0.5308660
C	5.1086840	-0.3028710	-1.1728150
H	5.2765390	-1.2998590	-1.6027540
H	5.8976870	0.3619030	-1.5651800
H	5.2610830	-0.3687540	-0.0854300
C	-3.5232040	2.2332140	1.1768990
H	-3.7821340	1.7041830	2.1059410
H	-2.4428250	2.4387210	1.1877110
H	-4.0488880	3.2024100	1.1976550
C	-4.5532410	-2.0912960	-0.9995940
H	-4.8536350	-2.7979020	-1.7928910
H	-4.2940670	-2.6919870	-0.1165060
H	-5.4332760	-1.4824370	-0.7536160

H	4.3452890	1.1850970	4.1458800
H	-2.1265770	-5.2595520	2.6531120

7”

Symbol	X	Y	Z
C	0.0954870	-2.4291270	2.7655830
C	-0.5889800	-1.4310780	2.0133400
C	-0.5249640	-3.5880770	3.2157720
C	-1.8823030	-3.8050630	2.9853980
C	-2.6032170	-2.7911340	2.3521680
N	0.0288760	-0.3342240	1.4779170
P	-2.7471030	0.1300400	-0.2904570
C	-3.3733700	-1.3004370	-1.3684920
C	-3.6634720	-0.7876230	-2.7843590
C	-2.1799760	-2.2631340	-1.4546910
C	-3.9984740	1.5662260	-0.3349370
C	-5.4706790	1.1419110	-0.2860740
C	-3.7434250	2.3879870	-1.6102660
C	3.6302010	0.1417340	2.4496720
C	1.2215150	0.1021220	2.0931370
C	3.5111530	1.1674680	3.3826370
C	2.2441180	1.6665730	3.6801390
C	1.1282490	1.1396910	3.0405590
P	2.1757370	-0.9814920	-0.9092820
C	3.6814490	-0.0470650	-1.5960760
C	1.9697970	-2.6529890	-1.7903090
C	1.2852320	-2.4252730	-3.1470390
C	3.2707750	-3.4352330	-1.9989440
C	3.5625510	0.0764730	-3.1200370

C	3.5579810	1.3631090	-1.0007060
Sc	-0.0615540	0.7024180	-0.6837930
C	-0.1534420	2.8663410	0.0757520
C	0.6520370	4.1714950	-0.0421320
C	1.9106240	4.0947720	0.8310400
C	-0.1779070	5.3816170	0.4322450
C	1.0496860	4.4145070	-1.5049860
C	-0.1356820	0.7143500	-2.6845400
H	1.1507450	-2.2770970	2.9974460
H	0.0666060	-4.3291230	3.7621700
H	-3.6890420	-2.8949760	2.2541530
H	-4.5917780	-0.1994660	-2.8380710
H	-3.7831500	-1.6478800	-3.4654580
H	-2.8212900	-0.1744350	-3.1468280
H	-1.3077120	-1.7432350	-1.8792700
H	-2.4238800	-3.1093630	-2.1202030
H	-1.9173060	-2.6768470	-0.4709360
H	-6.1065360	2.0407110	-0.2041340
H	-5.6937630	0.5058030	0.5848180
H	-5.7837570	0.6080310	-1.1941920
H	-4.0527680	1.8538990	-2.5180240
H	-2.6794110	2.6349250	-1.7332300
H	-4.3182720	3.3291370	-1.5652090
H	4.6214260	-0.2584960	2.2147490
H	2.1245390	2.4739940	4.4071220
H	0.1303350	1.5255340	3.2590020
H	1.0053580	-3.3997060	-3.5837800
H	1.9420370	-1.9173340	-3.8647820
H	0.3847320	-1.8043150	-3.0516720
H	3.9327490	-2.9522360	-2.7323420
H	3.0348210	-4.4413720	-2.3879370
H	3.8315800	-3.5707200	-1.0606720

H	3.7815560	-0.8709680	-3.6352470
H	4.2930610	0.8207170	-3.4817970
H	2.5515440	0.4171160	-3.3995570
H	4.3560850	2.0140550	-1.3980080
H	3.6430690	1.3596700	0.0950380
H	2.5929140	1.8171160	-1.2724630
H	2.5210490	5.0123690	0.7502500
H	2.5402210	3.2398640	0.5504770
H	1.6415990	3.9624430	1.8913940
H	-0.4919240	5.2522700	1.4816910
H	0.3850910	6.3312870	0.3642260
H	-1.0929040	5.4899200	-0.1744720
H	1.6248140	5.3507050	-1.6249430
H	0.1537900	4.4865090	-2.1441210
H	1.6537380	3.5837160	-1.8983280
H	-1.1306850	3.0519650	-0.4211530
H	-0.1486350	0.4424670	-3.7576000
C	2.7038470	-1.5085110	0.7933600
H	3.7305590	-1.9023600	0.8277180
H	2.0337690	-2.3383350	1.0516570
C	-2.8946760	-0.4747960	1.4533640
H	-2.5833970	0.4061930	2.0382450
H	-3.9422570	-0.6941670	1.7151380
C	-2.0093890	-1.6160920	1.8886500
C	2.5158380	-0.4019880	1.7968850
H	-0.2551090	1.8352120	-2.6274640
H	-0.4098220	2.7094910	1.1465340
C	1.0323320	-3.4962170	-0.9024730
H	0.2214660	-2.9020360	-0.4592940
H	0.5692410	-4.2946910	-1.5048080
H	1.5697830	-3.9845880	-0.0765680
C	5.0489240	-0.6227970	-1.2141290

H	5.1992660	-1.6507000	-1.5705720
H	5.8422890	-0.0002880	-1.6630200
H	5.2131530	-0.6103320	-0.1264670
C	-3.7269880	2.4634640	0.8866190
H	-4.0625430	1.9977510	1.8251430
H	-2.6649320	2.7272240	0.9882040
H	-4.2897660	3.4051150	0.7717670
C	-4.5779750	-2.0646430	-0.8101000
H	-4.8239340	-2.8981680	-1.4910190
H	-4.3595350	-2.5077760	0.1715930
H	-5.4790720	-1.4453460	-0.7151590
H	4.4004230	1.5729670	3.8710890
H	-2.3804930	-4.7125880	3.3333040

V^{IV} original ligand (L_0)

1

Symbol	X	Y	Z
C	-0.8942500	-3.4815050	-0.7612040
C	-1.0277060	-2.1411090	-0.3252500
C	-2.3434420	-1.6276420	-0.2359880
C	-2.0005540	-4.2596000	-1.0616950
C	-3.3075730	-3.7604300	-0.9615650
C	-3.4432800	-2.4347670	-0.5548310
N	0.0585900	-1.3158080	-0.0658410
P	-2.4689050	0.1413390	0.2052550

C	-3.7821970	0.7309450	-0.9858000
C	-4.2073120	2.1806560	-0.7621990
C	-3.3387580	0.5052070	-2.4332200
C	-3.2324210	0.1477520	1.9099380
C	-4.6834690	-0.3200470	1.9850610
C	-3.0240650	1.4818140	2.6260330
C	-4.4974740	-4.6148940	-1.3104890
C	3.7268550	-1.7775590	0.1935870
C	2.4749720	-1.2850390	-0.1724930
C	1.2876090	-1.8891060	0.2951350
C	3.8577160	-2.8733920	1.0515810
C	2.6782110	-3.4562840	1.5323340
C	1.4224150	-2.9844600	1.1668360
C	5.2075780	-3.3864370	1.4800520
P	2.1167850	0.1966810	-1.1621950
C	3.7049450	1.1368250	-1.3228480
C	1.7486140	-0.5728260	-2.8295990
C	0.8788080	0.3103760	-3.7241150
C	2.9835350	-1.1172620	-3.5432490
C	3.5885060	2.2436300	-2.3731370
C	4.1134020	1.7076870	0.0352060
V	0.0022890	0.7510470	0.0588650
C	0.5274300	1.0711190	1.7136510
C	0.9554840	0.7710230	3.1285320
C	0.7092170	1.9896270	4.0322700
C	2.4537770	0.4215450	3.1489630
C	0.1547390	-0.4297070	3.6548340
C	-0.3153810	2.5656120	-1.0786330
C	-0.3064120	4.0140940	-0.5218470
C	1.1095090	4.4304490	-0.1001860
C	-0.7783370	4.9782230	-1.6265850
C	-1.2417790	4.1831060	0.6844930

H	0.1018620	-3.9099040	-0.8800530
H	-1.8445390	-5.2877800	-1.4027010
H	-4.4504300	-2.0121270	-0.5055120
H	-4.6593310	0.0911860	-0.7940690
H	-4.5443750	2.3711740	0.2664110
H	-5.0452330	2.4277870	-1.4335330
H	-3.3885930	2.8782690	-0.9889110
H	-2.4748110	1.1364060	-2.6917570
H	-4.1574720	0.7731570	-3.1199090
H	-3.0679900	-0.5423170	-2.6290960
H	-2.5992620	-0.6023430	2.4133260
H	-5.0064480	-0.3594980	3.0378100
H	-4.8196740	-1.3265670	1.5656360
H	-5.3653150	0.3700650	1.4632040
H	-3.6692450	2.2765100	2.2215920
H	-1.9809920	1.8147050	2.5408210
H	-3.2662050	1.3772350	3.6958460
H	-4.5380440	-5.5259940	-0.6922300
H	-4.4647670	-4.9411620	-2.3627410
H	-5.4413970	-4.0705050	-1.1616060
H	4.6316370	-1.2914310	-0.1830970
H	2.7436230	-4.3006570	2.2250120
H	0.5290830	-3.4600410	1.5760760
H	6.0203080	-2.8889410	0.9312460
H	5.3022230	-4.4701950	1.3088870
H	5.3783500	-3.2116670	2.5548170
H	4.4739680	0.4193190	-1.6568410
H	1.1275660	-1.4308220	-2.5241770
H	0.6135720	-0.2347080	-4.6442620
H	1.3840960	1.2405010	-4.0246760
H	-0.0596570	0.5898930	-3.2230510
H	3.6358620	-0.3135180	-3.9190000

H	2.6814240	-1.7231960	-4.4126780
H	3.5818370	-1.7647830	-2.8838080
H	3.4364610	1.8486440	-3.3872480
H	4.5136030	2.8413440	-2.3896010
H	2.7555360	2.9250680	-2.1452300
H	5.0745570	2.2389670	-0.0522670
H	4.2275620	0.9247540	0.7973660
H	3.3611990	2.4235920	0.3993590
H	1.0094330	1.7759170	5.0715620
H	-0.3553480	2.2713430	4.0421790
H	1.2870750	2.8625430	3.6882500
H	3.0586260	1.2677160	2.7897590
H	2.7821250	0.1819760	4.1744450
H	2.6645300	-0.4479670	2.5086020
H	0.5184900	-0.7399530	4.6484490
H	0.2453280	-1.2863410	2.9713890
H	-0.9120670	-0.1784260	3.7511290
H	0.4698110	2.1511100	1.4069680
H	0.3992210	2.5550150	-1.9243350
H	-1.3027110	2.4127220	-1.5560330
H	1.1216960	5.4584640	0.2992450
H	1.8056780	4.4010680	-0.9533970
H	1.5128860	3.7668150	0.6804900
H	-0.7432180	6.0316350	-1.2985640
H	-1.8161550	4.7565350	-1.9257390
H	-0.1489440	4.8844230	-2.5266230
H	-0.8897580	3.6183160	1.5598750
H	-2.2599440	3.8349000	0.4580300
H	-1.3117430	5.2422970	0.9846190

1-2-TS

Symbol	X	Y	Z
C	-1.5178800	-3.4917720	-0.4068410
C	-1.4074190	-2.1053400	-0.1292570
C	-2.6159340	-1.3608000	-0.1336670
C	-2.7440060	-4.0890170	-0.6482490
C	-3.9420820	-3.3574510	-0.6445340
C	-3.8400520	-1.9909990	-0.3934680
N	-0.2008890	-1.4591670	0.0526510
P	-2.4378160	0.4351780	0.1430150
C	-3.5973680	1.1592430	-1.1262770
C	-3.8404670	2.6557870	-0.9342620
C	-3.0552390	0.8549690	-2.5254500
C	-3.1639310	0.7507650	1.8297590
C	-4.6451520	0.4168180	1.9818920
C	-2.8331650	2.1567680	2.3331540
C	-5.2659670	-4.0242660	-0.9125720
C	3.3559440	-2.3802070	0.4893640
C	2.1919910	-1.7648490	0.0300030
C	0.9187470	-2.1524760	0.5139840
C	3.3170260	-3.3928620	1.4527070
C	2.0577840	-3.7646150	1.9408730
C	0.8862340	-3.1669140	1.4895740
C	4.5759190	-4.0317060	1.9786450
P	2.0773100	-0.3837700	-1.1484260
C	3.7942600	0.2598070	-1.4223780
C	1.5674580	-1.2721250	-2.7155610
C	0.8347550	-0.3494370	-3.6910890
C	2.6803170	-2.0854780	-3.3696930
C	3.8295930	1.2387620	-2.5988840

C	4.3173240	0.9276950	-0.1517180
V	0.0661090	0.6275220	-0.0443680
C	0.6028820	1.1978770	1.4557770
C	1.0813160	1.4084350	2.8677460
C	0.8920450	2.8502910	3.3633230
C	2.5705760	1.0363590	2.9654620
C	0.2755310	0.4507760	3.7657140
C	0.1760000	2.6566090	-1.0453310
C	0.5694280	4.1213720	-0.7411300
C	2.0212130	4.1872600	-0.2497060
C	0.4403700	4.9597570	-2.0215160
C	-0.3651390	4.7007700	0.3279650
H	-0.6134330	-4.1004250	-0.4530240
H	-2.7733790	-5.1612550	-0.8664210
H	-4.7551220	-1.3918660	-0.4119430
H	-4.5633860	0.6404220	-1.0122240
H	-4.4173180	2.8686760	-0.0238240
H	-4.4154150	3.0543230	-1.7854270
H	-2.8985580	3.2221340	-0.8764010
H	-2.1200570	1.4044410	-2.7205210
H	-3.7827150	1.1671190	-3.2915370
H	-2.8607690	-0.2186770	-2.6691130
H	-2.5781380	0.0359380	2.4331040
H	-4.9611040	0.5848610	3.0241740
H	-4.8597090	-0.6337040	1.7412710
H	-5.2774590	1.0556910	1.3442650
H	-3.4699680	2.9248350	1.8704140
H	-1.7854260	2.4106100	2.1218900
H	-2.9904450	2.2164830	3.4220280
H	-5.5109150	-4.7671750	-0.1359560
H	-5.2645430	-4.5565090	-1.8774190
H	-6.0859980	-3.2917260	-0.9383220

H	4.3268370	-2.0654000	0.0951340
H	1.9912730	-4.5404830	2.7097540
H	-0.0719530	-3.4748110	1.9121900
H	5.4501370	-3.7613610	1.3684770
H	4.5009410	-5.1302600	1.9872630
H	4.7844290	-3.7135340	3.0134930
H	4.4348380	-0.6046950	-1.6671240
H	0.8196900	-1.9770220	-2.3171790
H	0.4434570	-0.9293550	-4.5424340
H	1.4825940	0.4402960	-4.1007370
H	-0.0253040	0.1414270	-3.2088130
H	3.4543440	-1.4464910	-3.8228080
H	2.2677610	-2.7164350	-4.1734830
H	3.1686750	-2.7545690	-2.6445030
H	3.6022840	0.7561750	-3.5594560
H	4.8342420	1.6820500	-2.6860920
H	3.1158320	2.0653060	-2.4549510
H	5.3487170	1.2824500	-0.3077520
H	4.3204640	0.2462490	0.7094440
H	3.6942510	1.7948910	0.1116350
H	1.2445790	2.9479050	4.4036150
H	-0.1662640	3.1499290	3.3430490
H	1.4614650	3.5659590	2.7514040
H	3.1895130	1.7281500	2.3754760
H	2.9160670	1.0834610	4.0122200
H	2.7419390	0.0156670	2.5905100
H	0.6339720	0.4958650	4.8081350
H	0.3714380	-0.5864880	3.4099910
H	-0.7928190	0.7134070	3.7629020
H	0.4670010	2.2142800	0.3310450
H	0.8077540	2.3070700	-1.8813330
H	-0.8646590	2.6639300	-1.4109220

H	2.3137510	5.2205980	-0.0023910
H	2.7159900	3.8193050	-1.0216540
H	2.1699360	3.5702670	0.6499420
H	0.7042550	6.0154630	-1.8412400
H	-0.5908930	4.9329810	-2.4097350
H	1.1049410	4.5786630	-2.8140760
H	-0.3281560	4.1117270	1.2552210
H	-1.4103010	4.7054270	-0.0211970
H	-0.0933280	5.7390080	0.5788650

A

Symbol	X	Y	Z
C	1.1152590	-2.8743850	-1.0831210
C	1.1522640	-1.5319050	-0.6283440
C	2.4357160	-1.0090780	-0.3075630
C	2.2689360	-3.6276200	-1.2256240
C	3.5375450	-3.1129380	-0.9182680
C	3.5824840	-1.8014420	-0.4508880
N	0.0182580	-0.7750490	-0.3999690
P	2.4710240	0.6823810	0.3835740
C	3.5558910	0.5115730	1.8893790
C	3.8643410	1.8688660	2.5218290
C	2.8600950	-0.4148580	2.8884060
C	3.3805550	1.7354220	-0.8554550
C	4.8609590	1.4106180	-1.0392070
C	3.1456730	3.2267480	-0.6044190
C	4.7792660	-3.9508540	-1.0745990
C	-3.6324580	-0.9901270	-0.9716540
C	-2.3975220	-0.7937720	-0.3519120

C	-1.1884580	-1.0443610	-1.0471060
C	-3.7310450	-1.4210160	-2.2972160
C	-2.5331570	-1.6415310	-2.9892860
C	-1.2935840	-1.4596120	-2.3881040
C	-5.0650120	-1.6406210	-2.9616380
P	-2.1472740	-0.1345310	1.3318950
C	-3.7594960	0.6005950	1.8787690
C	-1.8944890	-1.7080800	2.3129600
C	-1.1345640	-1.4721600	3.6185630
C	-3.1614160	-2.5370770	2.5030300
C	-3.7019170	0.9771840	3.3611650
C	-4.1032810	1.8183030	1.0219390
V	0.0175150	0.9665600	0.7047610
C	-0.3997600	2.2171690	-0.3508250
C	-0.7573240	2.9047430	-1.6448080
C	-0.5878110	4.4280420	-1.5129180
C	-2.2165650	2.5935490	-2.0195520
C	0.1556550	2.3798560	-2.7672060
H	0.1524740	-3.3371510	-1.3045340
H	2.1811720	-4.6624170	-1.5713970
H	4.5540880	-1.3867970	-0.1676520
H	4.5045200	0.0437080	1.5777740
H	4.5175290	2.4852200	1.8887070
H	4.3794610	1.7299950	3.4858820
H	2.9439100	2.4447020	2.7153120
H	1.9326200	0.0462060	3.2695030
H	3.5096480	-0.6038060	3.7578060
H	2.6027670	-1.3881650	2.4441280
H	2.8463300	1.4645690	-1.7814640
H	5.2905620	2.0629510	-1.8164500
H	5.0236330	0.3727790	-1.3608710
H	5.4380410	1.5828540	-0.1165380

H	3.7551620	3.6048800	0.2300500
H	2.0889340	3.4248290	-0.3737510
H	3.4219600	3.8082850	-1.4985530
H	4.6939230	-4.9064580	-0.5329410
H	5.6678390	-3.4285120	-0.6905270
H	4.9718860	-4.1960890	-2.1319560
H	-4.5536710	-0.7946210	-0.4152320
H	-2.5708940	-1.9521820	-4.0378690
H	-0.3850120	-1.6175640	-2.9720330
H	-5.8936740	-1.3067750	-2.3203510
H	-5.2312650	-2.7059490	-3.1907040
H	-5.1370380	-1.0902280	-3.9129120
H	-4.5395070	-0.1680240	1.7450810
H	-1.2181940	-2.2614800	1.6400740
H	-0.8664270	-2.4346050	4.0830430
H	-1.7228590	-0.9048140	4.3553140
H	-0.1964780	-0.9225340	3.4441050
H	-3.8693630	-2.0552840	3.1960040
H	-2.9121800	-3.5237600	2.9255030
H	-3.6794930	-2.7092290	1.5471210
H	-3.5962960	0.1008860	4.0159990
H	-4.6308300	1.4921820	3.6527970
H	-2.8649950	1.6640470	3.5688790
H	-5.0536400	2.2609600	1.3607700
H	-4.2094320	1.5603860	-0.0402790
H	-3.3160860	2.5844200	1.0987840
H	-0.8615210	4.9414970	-2.4512090
H	0.4538470	4.6943980	-1.2751430
H	-1.2269900	4.8254650	-0.7086450
H	-2.9104430	3.0098800	-1.2752080
H	-2.4706230	3.0349220	-2.9988860
H	-2.3859550	1.5074290	-2.0784010

H	-0.1309550	2.8051330	-3.7446930
H	0.0906000	1.2828580	-2.8332330
H	1.2053490	2.6503170	-2.5809280

5-6-TS

Symbol	X	Y	Z
C	1.1860010	-3.1494330	-0.6215660
C	1.1860920	-1.7462200	-0.4119190
C	2.4535510	-1.1381620	-0.2080660
C	2.3610500	-3.8811930	-0.6478270
C	3.6162370	-3.2821880	-0.4543440
C	3.6245200	-1.9085340	-0.2257070
N	0.0345010	-0.9927730	-0.3133350
P	2.4317520	0.6494030	0.1634780
C	3.5979060	0.7730120	1.6144760
C	3.9352550	2.2138020	1.9966220
C	2.9871940	0.0198540	2.7986660
C	3.2228320	1.4945600	-1.2948720
C	4.6654140	1.0958540	-1.5929610
C	3.0477480	3.0133970	-1.2238910
C	4.8827080	-4.0973360	-0.4796040
C	-3.5975160	-1.4069780	-0.8832980
C	-2.3760990	-1.0677410	-0.3018130
C	-1.1491040	-1.3983300	-0.9289230
C	-3.6645790	-2.0674370	-2.1134700
C	-2.4506660	-2.3684570	-2.7451200
C	-1.2235320	-2.0481010	-2.1759940
C	-4.9814270	-2.4182360	-2.7554550
P	-2.1310670	-0.1218450	1.2341500

C	-3.7611740	0.6218800	1.7059480
C	-1.7677800	-1.4842610	2.4645430
C	-0.9511590	-0.9858240	3.6576660
C	-2.9898560	-2.3040100	2.8683750
C	-3.6959190	1.2239810	3.1120020
C	-4.1669400	1.6835900	0.6851660
V	-0.0418140	0.9700190	0.4267340
C	-0.5183580	2.0192060	-0.8112280
C	-0.9213040	2.8658150	-1.9867320
C	-0.7414000	4.3589880	-1.6686050
C	-2.3918880	2.5955020	-2.3484650
C	-0.0446520	2.4753450	-3.1902120
H	0.2344530	-3.6692710	-0.7441470
H	2.3035440	-4.9628540	-0.8060180
H	4.5861480	-1.4204320	-0.0423030
H	4.5319390	0.2642060	1.3243910
H	4.5489860	2.7150810	1.2360320
H	4.5089360	2.2261350	2.9371920
H	3.0274750	2.8161610	2.1551080
H	2.0865120	0.5359980	3.1686430
H	3.7043850	-0.0252820	3.6334410
H	2.7122280	-1.0134030	2.5385990
H	2.5778290	1.1242530	-2.1103680
H	5.0226480	1.6287930	-2.4890410
H	4.7638800	0.0197500	-1.7921750
H	5.3442260	1.3598430	-0.7657450
H	3.7689340	3.4813770	-0.5385420
H	2.0341350	3.2787030	-0.8910400
H	3.2102790	3.4601590	-2.2177150
H	5.7680330	-3.4693730	-0.3017460
H	5.0221390	-4.5992820	-1.4507900
H	4.8739990	-4.8850520	0.2913050

H	-4.5299290	-1.1446230	-0.3741410
H	-2.4659390	-2.8621360	-3.7217580
H	-0.3031860	-2.2835770	-2.7137460
H	-5.0463470	-3.4937400	-2.9848400
H	-5.1226680	-1.8757720	-3.7045150
H	-5.8287070	-2.1661330	-2.1010930
H	-4.5106850	-0.1878620	1.7035620
H	-1.1049340	-2.1320440	1.8679730
H	-0.6792270	-1.8290550	4.3127700
H	-1.4955710	-0.2523190	4.2712440
H	-0.0119070	-0.5153770	3.3280480
H	-3.6842280	-1.7313320	3.5029180
H	-2.6799100	-3.1914900	3.4433600
H	-3.5447380	-2.6606290	1.9868690
H	-3.5469460	0.4649080	3.8925050
H	-4.6396410	1.7445430	3.3396050
H	-2.8819660	1.9621270	3.1940710
H	-5.1529160	2.1014320	0.9443400
H	-4.2257490	1.2813010	-0.3352890
H	-3.4368540	2.5074260	0.6751110
H	-1.0396480	4.9846650	-2.5267820
H	0.3073870	4.5952170	-1.4302810
H	-1.3589960	4.6555970	-0.8057030
H	-3.0652040	2.9431660	-1.5527450
H	-2.6672900	3.1242050	-3.2768840
H	-2.5671700	1.5190990	-2.4996760
H	-0.3431500	3.0366960	-4.0919360
H	-0.1422220	1.4000260	-3.4048040
H	1.0158220	2.6887490	-2.9957680
C	0.0259050	2.6489330	1.9587310
H	-0.2881690	2.6299630	0.5931650
H	0.8434850	3.3867310	1.9595570

H	0.2664770	1.8897710	2.7242850
H	-0.8844220	3.1629050	2.3067900

6

Symbol	X	Y	Z
C	-1.0399280	2.9605190	-0.9178690
C	-1.1257460	1.5919890	-0.5722720
C	-2.4193420	1.0762640	-0.3137530
C	-2.1729700	3.7536430	-1.0166120
C	-3.4571620	3.2472570	-0.7697730
C	-3.5447950	1.9032340	-0.4098660
N	-0.0116590	0.7777420	-0.4029030
P	-2.4716050	-0.6696320	0.2221220
C	-3.7372080	-0.6537570	1.5875640
C	-4.1063010	-2.0616280	2.0562150
C	-3.2360230	0.2126580	2.7446460
C	-3.2210580	-1.5876860	-1.2172280
C	-4.6699440	-1.2313790	-1.5386440
C	-3.0120410	-3.0988510	-1.1019420
C	-4.6747750	4.1288440	-0.8651200
C	3.6390330	1.0834090	-0.9668980
C	2.4067600	0.8499920	-0.3588900
C	1.1963950	1.0903450	-1.0469150
C	3.7277520	1.5470550	-2.2830640
C	2.5257880	1.7697300	-2.9672940
C	1.2880070	1.5492940	-2.3724290
C	5.0569200	1.7971050	-2.9463440
P	2.1008530	0.1788100	1.3038760

C	3.6876660	-0.5443690	1.9274690
C	1.8020240	1.7695670	2.2496350
C	0.9331290	1.5885370	3.4934330
C	3.0769800	2.5644090	2.5214280
C	3.5608040	-0.9088010	3.4090350
C	4.0755380	-1.7632920	1.0916140
V	-0.0055120	-0.9815080	0.6498520
C	0.5121710	-2.2672250	-0.4773370
C	0.9271050	-2.7661770	-1.8457030
C	0.7015950	-4.2822540	-1.9612440
C	2.4183480	-2.4660000	-2.0782850
C	0.1092640	-2.0446430	-2.9282080
H	-0.0607870	3.4088530	-1.0933900
H	-2.0552700	4.8092880	-1.2799910
H	-4.5319470	1.4906570	-0.1824860
H	-4.6442170	-0.1844650	1.1708690
H	-4.6447030	-2.6277460	1.2833880
H	-4.7676840	-2.0003240	2.9351070
H	-3.2179410	-2.6415490	2.3486710
H	-2.3460390	-0.2321250	3.2130510
H	-4.0160870	0.2960450	3.5181290
H	-2.9815000	1.2319060	2.4171110
H	-2.5835510	-1.2228050	-2.0397540
H	-4.9930890	-1.7675490	-2.4455440
H	-4.8011070	-0.1573250	-1.7314840
H	-5.3550440	-1.5248520	-0.7272990
H	-3.7087940	-3.5586490	-0.3855650
H	-1.9880930	-3.3343670	-0.7787960
H	-3.1835810	-3.5799520	-2.0782640
H	-5.5995120	3.5557210	-0.7041130
H	-4.7490140	4.6099580	-1.8533440
H	-4.6491420	4.9350520	-0.1138830

H	4.5611870	0.8905140	-0.4105030
H	2.5580770	2.1143610	-4.0052510
H	0.3756220	1.7187530	-2.9475960
H	5.1980400	2.8655460	-3.1766760
H	5.1422280	1.2470080	-3.8967120
H	5.8923800	1.4835600	-2.3037990
H	4.4699810	0.2264130	1.8205940
H	1.2022200	2.3349190	1.5176470
H	0.7502280	2.5666140	3.9669690
H	1.3991980	0.9371870	4.2480960
H	-0.0442740	1.1566990	3.2349720
H	3.7123380	2.0828610	3.2812160
H	2.8221190	3.5676810	2.8989080
H	3.6754690	2.6997180	1.6072460
H	3.4035900	-0.0271270	4.0461360
H	4.4833660	-1.4024140	3.7533210
H	2.7243780	-1.6037910	3.5817230
H	5.0121520	-2.2001910	1.4733880
H	4.2286320	-1.5073610	0.0343960
H	3.2928010	-2.5363590	1.1371060
H	0.9915500	-4.6488040	-2.9600740
H	-0.3560610	-4.5436540	-1.8017640
H	1.3010270	-4.8292270	-1.2157320
H	3.0453600	-2.9832830	-1.3371550
H	2.7293600	-2.8076330	-3.0801640
H	2.6189220	-1.3867270	-2.0071260
H	0.4733270	-2.3068360	-3.9355160
H	0.1814720	-0.9537760	-2.8096880
H	-0.9519930	-2.3274620	-2.8735930
C	-0.2232740	-1.7922800	2.6028840
H	0.4592470	-3.0263970	0.3465000
H	-1.1049090	-2.4516340	2.7051740

H	-0.2886160	-1.0396760	3.4087440
H	0.6600500	-2.4154790	2.8411380

6’

Symbol	X	Y	Z
C	-1.1023240	-3.2480080	-0.2734090
C	-1.1818180	-1.8590280	-0.0430900
C	-2.4471660	-1.2451400	-0.2048600
C	-2.2249180	-3.9772620	-0.6444150
C	-3.4786760	-3.3751780	-0.8131190
C	-3.5596110	-1.9992010	-0.5825770
N	-0.0886570	-1.0603580	0.2759460
P	-2.4258140	0.5407620	0.1884630
C	-3.9863840	1.2128590	-0.5646230
C	-4.2666610	2.6456710	-0.1100860
C	-3.9419670	1.1208100	-2.0914810
C	-2.7090930	0.4612910	2.0415550
C	-4.0911540	-0.0630880	2.4242620
C	-2.3548540	1.7437200	2.7916830
C	-4.6781740	-4.1768470	-1.2466010
C	3.4333600	-1.6796400	1.2246290
C	2.3068330	-1.1926630	0.5567160
C	1.0115280	-1.6033230	0.9402580
C	3.3267370	-2.5745520	2.2915610
C	2.0367850	-2.9773350	2.6665820
C	0.9041010	-2.5067630	2.0156010
C	4.5403170	-3.0634650	3.0377000
P	2.2987600	-0.0681230	-0.8834840
C	3.9091900	0.8522800	-0.7974310

C	2.4526340	-1.3029380	-2.2862680
C	1.9891610	-0.7553970	-3.6359030
C	3.8234720	-1.9684070	-2.3775440
C	4.0517650	1.7790350	-2.0068350
C	4.0269280	1.6244740	0.5168160
V	0.0081940	0.8777660	-0.4410870
C	0.4894900	2.2646590	0.6380280
C	0.5736200	3.7815520	0.6915890
C	-0.8381890	4.3746110	0.5245660
C	1.4419100	4.3153710	-0.4620290
C	1.1628870	4.2479400	2.0318390
C	-0.3883590	1.5789470	-2.3541330
H	-0.1425830	-3.7572250	-0.1666900
H	-2.1197050	-5.0521990	-0.8204580
H	-4.5294970	-1.5071720	-0.7009410
H	-4.8064760	0.5727450	-0.1980220
H	-4.4465180	2.7143400	0.9719790
H	-5.1672510	3.0278650	-0.6165150
H	-3.4330130	3.3191540	-0.3595500
H	-3.1947070	1.8102430	-2.5095560
H	-4.9233590	1.3962180	-2.5096130
H	-3.6986920	0.1087270	-2.4461180
H	-1.9577210	-0.2967400	2.3221910
H	-4.1319520	-0.2640040	3.5068290
H	-4.3361970	-1.0022120	1.9062480
H	-4.8810700	0.6719030	2.2009880
H	-3.0651220	2.5591690	2.5879160
H	-1.3482910	2.0941690	2.5276290
H	-2.3755060	1.5566760	3.8772980
H	-4.6193970	-4.4461210	-2.3143570
H	-5.6123580	-3.6145950	-1.1011750
H	-4.7617280	-5.1172850	-0.6800810

H	4.4295840	-1.3557060	0.9083120
H	1.9164790	-3.6732620	3.5023540
H	-0.0851760	-2.8361030	2.3406480
H	4.6436830	-2.5531350	4.0098700
H	5.4644680	-2.8800020	2.4703370
H	4.4810630	-4.1431670	3.2436770
H	4.7158890	0.1010650	-0.8354420
H	1.7185370	-2.0647290	-1.9723400
H	2.0089420	-1.5594390	-4.3892150
H	2.6369890	0.0539800	-4.0055220
H	0.9632950	-0.3685570	-3.5858170
H	4.5934150	-1.2653160	-2.7336500
H	3.7903010	-2.8028090	-3.0963810
H	4.1511570	-2.3801420	-1.4111930
H	4.1127850	1.2219530	-2.9529730
H	4.9722080	2.3777900	-1.9187050
H	3.2016450	2.4750180	-2.0777420
H	4.9621320	2.2068370	0.5281550
H	4.0319450	0.9623870	1.3939880
H	3.1871360	2.3220370	0.6391250
H	-0.8075650	5.4767900	0.5610840
H	-1.5182830	4.0273080	1.3152800
H	-1.2642110	4.0769590	-0.4453190
H	1.0624130	3.9606050	-1.4320230
H	1.4365010	5.4183390	-0.4738900
H	2.4878950	3.9898200	-0.3676060
H	1.2321420	5.3475360	2.0773240
H	2.1753090	3.8409060	2.1841870
H	0.5374800	3.9154150	2.8760850
H	-1.0328770	2.4757320	-2.2887320
H	0.5043090	1.8508860	-2.9443380
H	-0.9546050	0.8204380	-2.9295320

H	0.7458860	1.7213360	1.5767770

6’-7’-TS

Symbol	X	Y	Z
C	-1.3803550	-3.1400010	0.3385600
C	-1.3317200	-1.7251530	0.3323120
C	-2.5464340	-1.0486220	0.0590730
C	-2.5634070	-3.8214210	0.0932090
C	-3.7679780	-3.1543010	-0.1729130
C	-3.7255580	-1.7598720	-0.1820380
N	-0.1769550	-0.9874940	0.5097900
P	-2.4134060	0.7669470	0.0887970
C	-3.7360880	1.3776790	-1.0656930
C	-3.7870330	2.9076520	-1.0833930
C	-3.5541600	0.8025010	-2.4728130
C	-2.9691680	1.1644680	1.8322190
C	-4.4099450	0.7718930	2.1472780
C	-2.6701450	2.6034920	2.2510440
C	-5.0375920	-3.9159880	-0.4506180
C	3.3622680	-1.9129090	1.0385940
C	2.2005350	-1.3699170	0.4864640
C	0.9456480	-1.5775070	1.0970220
C	3.3279230	-2.6673620	2.2142480
C	2.0797150	-2.8564080	2.8252860
C	0.9132100	-2.3257050	2.2882860
C	4.5783950	-3.2624220	2.8077620
P	2.0465950	-0.3628270	-1.0278900
C	3.7744650	0.1672700	-1.4476730
C	1.5695350	-1.6757010	-2.2768700

C	0.9097440	-1.1152440	-3.5356360
C	2.6962940	-2.6573950	-2.5896020
C	3.8340120	0.8353980	-2.8225530
C	4.3383510	1.0898510	-0.3690200
V	0.0910070	0.9736250	-0.2741480
C	0.8362640	2.3858810	0.6537140
C	1.4602900	3.7388410	0.9489190
C	0.4049710	4.6926320	1.5393280
C	2.0118730	4.3590280	-0.3414500
C	2.5895130	3.5598150	1.9753520
C	-0.1346530	1.8520370	-1.9351720
H	-0.4676610	-3.7090370	0.5220000
H	-2.5486920	-4.9159820	0.0954230
H	-4.6481670	-1.2107040	-0.3934800
H	-4.6950630	1.0073720	-0.6652320
H	-4.1052260	3.3245500	-0.1180370
H	-4.5095370	3.2508800	-1.8407890
H	-2.8077190	3.3446950	-1.3334030
H	-2.6055800	1.1277350	-2.9222440
H	-4.3759700	1.1451380	-3.1217670
H	-3.5594620	-0.2964670	-2.4758820
H	-2.2942780	0.5038340	2.4051840
H	-4.6131980	0.9199030	3.2201360
H	-4.6142850	-0.2832840	1.9160000
H	-5.1301290	1.3954630	1.5931520
H	-3.3983870	3.3154200	1.8348980
H	-1.6700350	2.9200460	1.9290350
H	-2.7183330	2.6958840	3.3477560
H	-5.8842610	-3.2357590	-0.6242540
H	-5.3081620	-4.5755240	0.3896940
H	-4.9384900	-4.5552910	-1.3430190
H	4.3247950	-1.7518340	0.5440890

H	2.0220980	-3.4296320	3.7556260
H	-0.0416700	-2.4848180	2.7943250
H	5.4713670	-2.9778650	2.2325800
H	4.5310660	-4.3633430	2.8253350
H	4.7308760	-2.9292040	3.8466420
H	4.3829040	-0.7524120	-1.4811630
H	0.7873630	-2.2143570	-1.7161020
H	0.4984970	-1.9412690	-4.1379800
H	1.6193500	-0.5652510	-4.1727010
H	0.0887560	-0.4309310	-3.2774680
H	3.4959670	-2.1877850	-3.1847070
H	2.3068300	-3.5027350	-3.1792900
H	3.1462520	-3.0739910	-1.6752080
H	3.5637540	0.1471320	-3.6355510
H	4.8566860	1.1934060	-3.0217910
H	3.1603120	1.7052840	-2.8761720
H	5.3941860	1.3209850	-0.5829280
H	4.2841640	0.6502120	0.6370250
H	3.7813240	2.0355880	-0.3460860
H	0.8408500	5.6865950	1.7339380
H	0.0109850	4.3105490	2.4940210
H	-0.4431300	4.8251980	0.8487790
H	1.2123220	4.5167780	-1.0831840
H	2.4761580	5.3368300	-0.1367200
H	2.7698050	3.7141060	-0.8091670
H	3.0527760	4.5294890	2.2213610
H	3.3747870	2.8912470	1.5962790
H	2.2045400	3.1234870	2.9109650
H	0.1562270	2.6278820	-0.4992750
H	0.7348000	2.0962510	-2.5735610
H	-1.0232540	2.3819700	-2.3186980
H	0.4559790	1.8657730	1.5755410

6’-7”-TS

Symbol	X	Y	Z
C	0.8665770	-3.3288180	-0.2868490
C	1.0072240	-1.9221560	-0.2823190
C	2.2930700	-1.4042060	0.0070990
C	1.9480070	-4.1574800	-0.0201970
C	3.2230830	-3.6498880	0.2661580
C	3.3639650	-2.2605990	0.2730280
N	-0.0338140	-1.0339140	-0.4756840
P	2.3826210	0.4174570	-0.0782940
C	3.7666770	0.8900710	1.0696510
C	4.0053770	2.4012490	1.0406310
C	3.5249290	0.3848990	2.4935630
C	3.0326640	0.6493070	-1.8235080
C	4.4579940	0.1505700	-2.0452520
C	2.8345700	2.0631880	-2.3611310
C	4.3818790	-4.5692410	0.5534640
C	-3.6440040	-1.3616850	-1.1986820
C	-2.4400780	-1.0398800	-0.5696080
C	-1.2016470	-1.4333000	-1.1256120
C	-3.6703810	-2.0794980	-2.3974080
C	-2.4388650	-2.4731420	-2.9415120
C	-1.2307520	-2.1607000	-2.3302200
C	-4.9648960	-2.4102940	-3.0932450
P	-2.2072460	-0.1499690	1.0057500
C	-3.8180260	0.6864720	1.3768930
C	-2.0084890	-1.5751690	2.1983090
C	-1.2613680	-1.1947750	3.4756700

C	-3.3107300	-2.3249450	2.4669080
C	-3.8028510	1.2879100	2.7839280
C	-4.0958310	1.7652680	0.3295240
V	-0.0209060	0.9317670	0.3156560
C	-0.3414470	2.5175560	-0.5902380
C	-0.3387150	4.0169500	-0.8509850
C	0.9023710	4.6400220	-0.1960280
C	-1.5991660	4.6687110	-0.2527190
C	-0.3255180	4.2871270	-2.3628390
C	0.3043730	1.6821200	2.0244810
H	-0.1108700	-3.7718160	-0.4872160
H	1.7935620	-5.2410730	-0.0229540
H	4.3465300	-1.8361930	0.5012400
H	4.6705040	0.3922340	0.6794250
H	4.3266030	2.7538720	0.0502060
H	4.7966860	2.6722000	1.7577190
H	3.0966870	2.9576010	1.3168720
H	2.6280930	0.8410930	2.9350290
H	4.3901460	0.6381470	3.1274030
H	3.3891550	-0.7052850	2.5286080
H	2.3424260	-0.0107180	-2.3776480
H	4.7011650	0.1749790	-3.1197890
H	4.5972880	-0.8847120	-1.7021470
H	5.1959080	0.7888140	-1.5329630
H	3.5386390	2.7822290	-1.9147220
H	1.8140350	2.4109510	-2.1583390
H	2.9977080	2.0813180	-3.4507770
H	4.1785500	-5.2216790	1.4180200
H	5.2982840	-4.0027010	0.7749810
H	4.5984720	-5.2278530	-0.3033000
H	-4.5914370	-1.0510940	-0.7475810
H	-2.4269440	-3.0321690	-3.8821220

H	-0.2898210	-2.4718210	-2.7893730
H	-5.8343080	-2.1482140	-2.4729010
H	-5.0333500	-3.4838860	-3.3287020
H	-5.0598900	-1.8631360	-4.0455110
H	-4.6107350	-0.0794820	1.3265160
H	-1.3426080	-2.2371560	1.6202820
H	-1.0713790	-2.0985370	4.0766080
H	-1.8316830	-0.4945390	4.1054690
H	-0.2936160	-0.7290990	3.2405760
H	-4.0114590	-1.7293350	3.0732900
H	-3.1031170	-3.2510080	3.0265910
H	-3.8195860	-2.6120990	1.5337000
H	-3.7210560	0.5218810	3.5680660
H	-4.7350410	1.8459620	2.9655830
H	-2.9637230	1.9918770	2.9062930
H	-5.0821430	2.2229700	0.5066730
H	-4.0878270	1.3715900	-0.6974400
H	-3.3400390	2.5613090	0.3878460
H	0.9284050	5.7297220	-0.3590180
H	1.8251490	4.2069760	-0.6087880
H	0.9049940	4.4623020	0.8910460
H	-1.6737200	4.4741960	0.8295280
H	-1.5814580	5.7616980	-0.3969750
H	-2.5113230	4.2841230	-0.7345750
H	-0.3376710	5.3709050	-2.5634430
H	-1.2095100	3.8456410	-2.8505360
H	0.5686740	3.8683060	-2.8465440
H	-0.4062980	2.4925130	0.7770820
H	1.1589450	2.3437120	2.2518690
H	-0.4165320	1.7133510	2.8613490
H	-1.0225160	1.9417140	-1.2723870

7’

Symbol	X	Y	Z
C	-0.6217910	-3.2920820	-0.7253030
C	-0.8672500	-1.9787950	-0.2800610
C	-2.1964310	-1.5038920	-0.3371730
C	-1.6577940	-4.0893900	-1.1951860
C	-2.9787150	-3.6260690	-1.2564200
C	-3.2203060	-2.3209480	-0.8186300
N	0.1310240	-1.1021300	0.1438810
P	-2.3702940	0.1872950	0.3445960
C	-4.0411440	0.7494680	-0.2510450
C	-4.5339650	2.0164230	0.4493510
C	-4.0461810	0.9297290	-1.7696830
C	-2.5548640	-0.2742600	2.1585950
C	-3.8891390	-0.9359400	2.4946270
C	-2.1982440	0.8368040	3.1426530
C	-4.0860570	-4.5048750	-1.7776920
C	3.6542510	-1.3828880	1.2411010
C	2.5099180	-0.9631620	0.5569200
C	1.2655580	-1.5863530	0.7947880
C	3.6188950	-2.4271120	2.1674710
C	2.3812500	-3.0486960	2.3876710
C	1.2305930	-2.6439320	1.7247510
C	4.8479540	-2.8524340	2.9273590
P	2.4277890	0.3510330	-0.7107120
C	3.8985510	1.4367470	-0.3615960
C	2.8111780	-0.6079820	-2.2715450
C	2.5524400	0.1781610	-3.5575490
C	4.1874820	-1.2678620	-2.2781080

C	4.0740200	2.5129420	-1.4354860
C	3.8116330	2.0688780	1.0304080
V	-0.0677550	0.8326310	-0.4800510
C	0.3434670	2.7350150	0.3212780
C	-0.5634440	3.9783160	0.4233610
C	-1.3397000	4.1725980	-0.8854170
C	0.3019960	5.2266950	0.6719530
C	-1.5524120	3.8415530	1.5807970
C	-0.5758420	0.9271520	-2.2318710
H	0.3971540	-3.6839760	-0.7081010
H	-1.4303470	-5.1036650	-1.5371620
H	-4.2448390	-1.9398230	-0.8560150
H	-4.7347190	-0.0676470	0.0085320
H	-4.5142500	1.9323560	1.5455180
H	-5.5754830	2.2200280	0.1536840
H	-3.9349230	2.8932320	0.1650220
H	-3.3779160	1.7495930	-2.0727850
H	-5.0637200	1.1764440	-2.1130390
H	-3.7183540	0.0255530	-2.3019610
H	-1.7634140	-1.0378810	2.2426510
H	-3.8579000	-1.3469690	3.5165650
H	-4.1208090	-1.7686730	1.8136500
H	-4.7233920	-0.2175660	2.4594860
H	-2.9372160	1.6521200	3.1460470
H	-1.2137360	1.2722140	2.9210070
H	-2.1606560	0.4254960	4.1642750
H	-3.9235640	-4.7759890	-2.8334230
H	-5.0626370	-4.0037620	-1.7109670
H	-4.1549030	-5.4462640	-1.2094540
H	4.6097290	-0.8861560	1.0483850
H	2.3148270	-3.8664450	3.1115640
H	0.2813550	-3.1427180	1.9318550

H	4.7977750	-2.5313160	3.9809690
H	5.7608000	-2.4183630	2.4940540
H	4.9649060	-3.9472200	2.9284700
H	4.7824040	0.7768910	-0.3894300
H	2.0406370	-1.3960330	-2.2073440
H	2.4907640	-0.5163030	-4.4103790
H	3.3626570	0.8887940	-3.7776490
H	1.6062750	0.7357400	-3.5070840
H	4.9947340	-0.5209690	-2.3505040
H	4.2825630	-1.9311070	-3.1528960
H	4.3612500	-1.8790440	-1.3803990
H	4.3372860	2.0906030	-2.4143910
H	4.8860360	3.1987510	-1.1459020
H	3.1599670	3.1152120	-1.5598010
H	4.7677550	2.5590040	1.2741990
H	3.6029810	1.3326780	1.8201440
H	3.0249890	2.8354820	1.0673150
H	-1.9677650	5.0793330	-0.8559640
H	-1.9911650	3.3081050	-1.0839310
H	-0.6522510	4.2712360	-1.7419490
H	1.0145930	5.3872950	-0.1540140
H	-0.3123600	6.1391680	0.7654290
H	0.8873160	5.1210850	1.6002650
H	-2.2246040	4.7138280	1.6467330
H	-1.0275080	3.7493300	2.5456060
H	-2.1731740	2.9473550	1.4532510
H	1.1902370	3.0075190	-0.3405590
H	-0.6637040	1.8965960	-2.7506170
H	-0.8218130	0.0582430	-2.8643630
H	0.8114470	2.5352340	1.3092480

7''

Symbol	X	Y	Z
C	-1.4267380	-3.0578180	-0.4255960
C	-1.4097750	-1.6816050	-0.1309120
C	-2.6330490	-0.9807470	-0.2205710
C	-2.6108420	-3.6940480	-0.7805370
C	-3.8278930	-3.0046090	-0.8665430
C	-3.8105430	-1.6366500	-0.5784350
N	-0.2491540	-0.9658890	0.1817190
P	-2.4314600	0.7698180	0.2346290
C	-3.8959090	1.6806720	-0.4456510
C	-3.9128480	3.1268500	0.0557440
C	-3.9212550	1.6288690	-1.9731450
C	-2.6788490	0.6444870	2.0913500
C	-4.1309310	0.4107020	2.5016640
C	-2.0196570	1.7686040	2.8895470
C	-5.0943120	-3.7113120	-1.2743390
C	3.1413790	-1.9012300	1.3385590
C	2.1126720	-1.3047660	0.6025840
C	0.7656640	-1.5917990	0.9099940
C	2.8858870	-2.7846570	2.3897860
C	1.5443950	-3.0592840	2.6883750
C	0.5068190	-2.4797690	1.9714790
C	4.0014890	-3.4138370	3.1827040
P	2.3158340	-0.1622230	-0.8179790
C	4.1338980	0.2710120	-0.7751070
C	2.1314630	-1.3647510	-2.2490010
C	2.0547800	-0.6922970	-3.6216300
C	3.1399640	-2.5101820	-2.2389330
C	4.6007560	0.9695150	-2.0549210

C	4.5201680	1.0905760	0.4586670
V	-0.0962830	0.9278500	-0.5631020
C	-0.0262590	2.9351270	0.0992270
C	1.2349840	3.7077020	0.5197500
C	0.8767700	5.1547960	0.8985720
C	2.2321220	3.7465880	-0.6429030
C	1.8495430	3.0222080	1.7477060
C	-0.6118880	0.9217120	-2.3268730
H	-0.4987350	-3.6312180	-0.3778840
H	-2.5864750	-4.7641200	-1.0080650
H	-4.7464550	-1.0723120	-0.6366700
H	-4.7930200	1.1667810	-0.0606680
H	-3.9987190	3.1918230	1.1499810
H	-4.7742590	3.6629010	-0.3733870
H	-3.0000300	3.6646900	-0.2446250
H	-3.0671820	2.1751520	-2.3994730
H	-4.8472830	2.0952060	-2.3463420
H	-3.8806070	0.6001140	-2.3593810
H	-2.1115600	-0.2764110	2.3075850
H	-4.1843520	0.1643780	3.5743270
H	-4.5843740	-0.4267840	1.9498690
H	-4.7523900	1.3059880	2.3418810
H	-2.4628580	2.7546500	2.6823020
H	-0.9434070	1.8320020	2.6780020
H	-2.1377760	1.5752240	3.9680080
H	-5.0833810	-3.9709210	-2.3458250
H	-5.9803970	-3.0847250	-1.0962520
H	-5.2299160	-4.6499830	-0.7150740
H	4.1831540	-1.6858190	1.0877070
H	1.3055680	-3.7390510	3.5118060
H	-0.5282170	-2.7115830	2.2317400
H	3.9384630	-4.5135360	3.1661460

H	3.9669310	-3.1040970	4.2397580
H	4.9870580	-3.1300060	2.7862260
H	4.6544170	-0.6997630	-0.7168520
H	1.1319370	-1.7750480	-2.0267920
H	1.5852090	-1.3783370	-4.3444760
H	3.0501740	-0.4423040	-4.0158600
H	1.4521900	0.2268070	-3.5949490
H	4.1581420	-2.1598160	-2.4742080
H	2.8700310	-3.2530290	-3.0068250
H	3.1701580	-3.0304390	-1.2701690
H	4.5938920	0.2979970	-2.9230760
H	5.6364310	1.3220070	-1.9252780
H	3.9829690	1.8474230	-2.2992290
H	5.6180900	1.1256660	0.5449320
H	4.1267000	0.6715120	1.3955070
H	4.1627490	2.1250360	0.3777570
H	1.7679020	5.7329610	1.1997130
H	0.1633850	5.1782800	1.7388660
H	0.4076210	5.6796790	0.0502960
H	1.8099940	4.3014850	-1.4971100
H	3.1795970	4.2372310	-0.3620410
H	2.4581100	2.7310190	-0.9959630
H	2.7868420	3.5059860	2.0718970
H	2.0655860	1.9616970	1.5452970
H	1.1492720	3.0567490	2.5986300
H	-0.4344700	3.3818130	-0.8352130
H	-0.6041500	1.8291700	-2.9535350
H	-0.9635800	0.0245150	-2.8630790
H	-0.8080470	3.1197530	0.8559590

V^{IV} (L_1)

1

Symbol	X	Y	Z
C	0.6960320	3.4472390	-1.4478190
C	0.9709390	2.2531660	-0.7453630
C	2.3022440	1.7707500	-0.7626520
C	1.6960920	4.1434720	-2.1111470
C	3.0124660	3.6795730	-2.0982490
C	3.2992000	2.4994630	-1.4201540
N	-0.0255600	1.5363230	-0.0805600
P	2.5221260	0.1998950	0.1463980
C	3.9730330	-0.7441450	-0.6425110
C	3.8833140	-2.1901210	-0.1262430
C	3.7518850	-0.7525470	-2.1635700
C	3.0196780	0.7931990	1.8908590
C	4.0192410	1.9558530	1.8662570
C	3.5915120	-0.3680080	2.7113260
C	-3.2874210	2.4098860	1.4543310
C	-2.3101720	1.7106890	0.7376080
C	-1.0073140	2.2504980	0.6019990
C	-3.0134620	3.6297410	2.0636480
C	-1.7301330	4.1649630	1.9396310

C	-0.7488020	3.4943920	1.2243140
P	-2.5534570	0.1324450	-0.1475970
C	-3.7889930	-0.9089090	0.8492000
C	-3.3393050	0.7301840	-1.7839300
C	-3.7498280	-0.4753780	-2.6377650
C	-4.5481920	1.6559250	-1.5938960
C	-3.6201590	-2.3532610	0.3555430
C	-3.3308310	-0.8270390	2.3132960
V	0.0209680	-0.5421380	-0.0309040
C	0.0442200	-1.8318320	1.2200930
C	0.1351480	-2.7864370	2.3917490
C	-1.0705020	-3.7453430	2.4117700
C	0.1850960	-2.0225650	3.7221840
C	1.4029070	-3.6493240	2.2460770
C	0.0252770	-1.3653970	-1.9776780
H	-0.3247370	3.8314810	-1.4632990
H	1.4431190	5.0606610	-2.6489820
H	4.3293100	2.1443780	-1.4003430
H	4.1839330	-2.2744110	0.9263200
H	4.5593290	-2.8344540	-0.7122900
H	2.8670930	-2.5897770	-0.2201530
H	2.7438840	-1.0898720	-2.4317110
H	4.4705450	-1.4481120	-2.6273710
H	3.9040240	0.2355540	-2.6179480
H	4.2458020	2.2542570	2.9037770
H	3.6064290	2.8358970	1.3528690
H	4.9714840	1.6963560	1.3860510
H	4.5898240	-0.6788180	2.3752920
H	2.9305030	-1.2436860	2.6896310
H	3.6882070	-0.0540850	3.7636140
H	-4.2933810	1.9963970	1.5341370
H	-1.4863260	5.1192540	2.4135560

H	0.2449740	3.9371850	1.1416900
H	-4.0912630	-0.1246900	-3.6258180
H	-4.5807350	-1.0397000	-2.1900620
H	-2.9139080	-1.1686130	-2.8072760
H	-5.4093150	1.1568300	-1.1355580
H	-4.8733110	2.0180890	-2.5837920
H	-4.2973300	2.5406610	-0.9917690
H	-3.9681260	-2.4821570	-0.6790530
H	-4.2101090	-3.0361820	0.9894430
H	-2.5700450	-2.6625800	0.4003910
H	-3.8235930	-1.6215230	2.8979180
H	-3.5859320	0.1352350	2.7771340
H	-2.2433900	-0.9648260	2.3963060
H	-0.9483330	-4.4936660	3.2123470
H	-1.1646420	-4.2894280	1.4584730
H	-2.0122220	-3.2129860	2.5942720
H	-0.6920500	-1.3692780	3.8434240
H	0.2017070	-2.7249770	4.5714250
H	1.0821460	-1.3919690	3.7941100
H	1.4980840	-4.3469440	3.0946540
H	2.3122720	-3.0357140	2.2096200
H	1.3689250	-4.2481340	1.3218830
H	-0.8794050	-0.8769470	-2.3755410
H	0.8649810	-0.8220550	-2.4537250
H	0.0902510	-2.3985540	0.2315380
C	1.7376750	1.2837890	2.5795730
H	1.3306100	2.1791550	2.0950670
H	0.9503820	0.5169490	2.5889880
H	1.9686810	1.5497160	3.6246770
C	-5.2759790	-0.5460600	0.7652890
H	-5.6891850	-0.7108770	-0.2395630
H	-5.8355170	-1.2023500	1.4529780

H	-5.4924720	0.4891150	1.0613920
C	-2.2659210	1.5430460	-2.5270260
H	-2.0093950	2.4576780	-1.9753800
H	-1.3388530	0.9834810	-2.7051190
H	-2.6661910	1.8514890	-3.5070460
C	5.3881650	-0.2197360	-0.3620480
H	6.1084450	-0.8581810	-0.9006020
H	5.5499730	0.8084770	-0.7113280
H	5.6555370	-0.2643820	0.7015530
H	-3.7891420	4.1556220	2.6232750
H	3.8048580	4.2279580	-2.6110190
C	0.0020260	-2.8152540	-2.5177250
C	-1.2277650	-3.5732710	-2.0041890
H	-2.1608880	-3.0413810	-2.2414300
H	-1.2917890	-4.5765500	-2.4570890
H	-1.1903070	-3.7060060	-0.9126240
C	-0.0682020	-2.7646260	-4.0553290
H	0.8041860	-2.2369760	-4.4738920
H	-0.0942200	-3.7754270	-4.4972160
H	-0.9708210	-2.2283240	-4.3912780
C	1.2458790	-3.6154520	-2.1181580
H	1.3224510	-3.7127400	-1.0243300
H	1.2060890	-4.6353790	-2.5356150
H	2.1712070	-3.1460960	-2.4826490

1-2-TS

Symbol	X	Y	Z
C	-0.7583300	-3.5272810	-1.5010860
C	-1.0030620	-2.3347610	-0.7780300

C	-2.3164400	-1.7981200	-0.8308100
C	-1.7648870	-4.1655300	-2.2106960
C	-3.0616760	-3.6482350	-2.2307960
C	-3.3197080	-2.4707650	-1.5359820
N	-0.0099870	-1.6642860	-0.0785420
P	-2.5053320	-0.2427780	0.1108120
C	-3.8831650	0.7826060	-0.7072120
C	-3.7489550	2.2064840	-0.1439070
C	-3.5967660	0.8274380	-2.2171560
C	-3.0887780	-0.8584650	1.8194230
C	-4.1481420	-1.9629830	1.7269130
C	-3.6227770	0.3115790	2.6527480
C	3.3256020	-2.6203580	1.2202170
C	2.3114410	-1.8798880	0.6023630
C	0.9975200	-2.4056010	0.5243270
C	3.0767620	-3.8624240	1.7939470
C	1.7789750	-4.3760460	1.7428200
C	0.7623190	-3.6672810	1.1207520
P	2.5033190	-0.2356110	-0.1695490
C	3.7982070	0.7269110	0.8252000
C	3.1751520	-0.6562000	-1.9035420
C	3.6885810	0.6063490	-2.6090080
C	4.2813640	-1.7181190	-1.8850590
C	3.6325110	2.1997280	0.4199640
C	3.4029660	0.5666680	2.3017210
V	-0.0138870	0.4723970	0.0587490
C	-0.0043490	1.6479200	1.3106020
C	-0.0742800	2.6401590	2.4504400
C	1.1797580	3.5272490	2.5401360
C	-0.2024220	1.8619340	3.7730670
C	-1.2922250	3.5653700	2.2880990
C	0.0785420	1.9156890	-1.6086560

H	0.2459760	-3.9543460	-1.4981450
H	-1.5319120	-5.0808460	-2.7611220
H	-4.3339890	-2.0714810	-1.5418630
H	-4.0941430	2.2744040	0.8960020
H	-4.3640020	2.9019360	-0.7387650
H	-2.7095150	2.5530090	-0.1742980
H	-2.5702730	1.1490070	-2.4365520
H	-4.2759740	1.5547010	-2.6917980
H	-3.7523630	-0.1432990	-2.7065070
H	-4.4278210	-2.2767780	2.7468510
H	-3.7645750	-2.8501820	1.2032760
H	-5.0674330	-1.6397040	1.2219500
H	-4.5977230	0.6752770	2.3003150
H	-2.9196490	1.1545910	2.6612540
H	-3.7584250	-0.0175430	3.6961370
H	4.3400190	-2.2228530	1.2515050
H	1.5542580	-5.3434870	2.1994320
H	-0.2433460	-4.0901790	1.0892440
H	3.8981630	0.3705270	-3.6655330
H	4.6236000	0.9816100	-2.1701320
H	2.9534800	1.4250240	-2.5983490
H	5.1643520	-1.4075130	-1.3116680
H	4.6132090	-1.9111940	-2.9193480
H	3.9222390	-2.6711210	-1.4713740
H	3.9931800	2.3908500	-0.6001920
H	4.2161590	2.8431910	1.0993220
H	2.5816830	2.5062070	0.4759310
H	3.9453030	1.3092430	2.9102530
H	3.6511790	-0.4297290	2.6919280
H	2.3238330	0.7253600	2.4442080
H	1.0706070	4.2530510	3.3631230
H	1.3393070	4.1001590	1.6151490

H	2.0833670	2.9361950	2.7346840
H	0.6458640	1.1744600	3.9113270
H	-0.2218590	2.5549930	4.6308680
H	-1.1236490	1.2638870	3.8042400
H	-1.3808360	4.2469400	3.1505790
H	-2.2273590	2.9956710	2.2091190
H	-1.1993590	4.1852150	1.3835940
H	1.0193660	1.4672440	-1.9609670
H	-0.7363120	1.3842270	-2.1318480
H	-0.0007580	2.1480260	-0.1558680
C	-1.8575400	-1.4389600	2.5314190
H	-1.4636380	-2.3217900	2.0118970
H	-1.0428330	-0.7055410	2.6109510
H	-2.1449700	-1.7508000	3.5495540
C	5.2716530	0.3415500	0.6483520
H	5.6296400	0.5128640	-0.3760520
H	5.8830610	0.9757900	1.3122650
H	5.4868240	-0.7015030	0.9154750
C	1.9898030	-1.2214070	-2.7027570
H	1.5739870	-2.1248400	-2.2366290
H	1.1721690	-0.4947740	-2.8172050
H	2.3322680	-1.4962670	-3.7143650
C	-5.3308400	0.3169100	-0.5006610
H	-6.0008860	1.0043410	-1.0439190
H	-5.5248380	-0.6919520	-0.8879740
H	-5.6354600	0.3415090	0.5536320
H	3.8819120	-4.4199710	2.2760540
H	-3.8587670	-4.1524440	-2.7802570
C	0.0854980	3.3800530	-2.1029220
C	1.2598990	4.1464630	-1.4877150
H	2.2213490	3.6683070	-1.7295150
H	1.2949930	5.1817750	-1.8630980

H	1.1758060	4.1926550	-0.3921140
C	0.2529630	3.3641340	-3.6314110
H	-0.5785070	2.8286650	-4.1176030
H	0.2784320	4.3875440	-4.0408640
H	1.1890120	2.8618850	-3.9249730
C	-1.2193160	4.1039260	-1.7634680
H	-1.3767090	4.1585680	-0.6766140
H	-1.2037780	5.1365750	-2.1478590
H	-2.0885890	3.5951690	-2.2069350

A

Symbol	X	Y	Z
C	-0.8271590	-3.1852240	1.3484710
C	-1.0160400	-1.8603780	0.8731020
C	-2.2852590	-1.5689890	0.2839860
C	-1.8272410	-4.1418700	1.2652920
C	-3.0730620	-3.8389050	0.7120090
C	-3.2809350	-2.5509950	0.2305440
N	-0.0223640	-0.9034950	0.8757090
P	-2.4758440	0.1311130	-0.3552700
C	-3.2181400	0.0558970	-2.0965390
C	-2.9674720	1.4276290	-2.7491100
C	-2.3937350	-0.9937340	-2.8576960
C	-3.6231990	0.9627560	0.8918040
C	-4.8838760	0.1609590	1.2334490
C	-4.0156110	2.3479640	0.3668830
C	3.5883470	-1.1298920	1.6951980
C	2.4075940	-0.8813980	0.9864590
C	1.1508630	-1.1019630	1.6070930

C	3.5600840	-1.5464730	3.0225510
C	2.3258400	-1.7043220	3.6562920
C	1.1428380	-1.4844910	2.9630320
P	2.3163360	-0.2001120	-0.7111270
C	3.7408950	1.0255170	-0.9600510
C	2.5073540	-1.7127900	-1.8409140
C	2.5665710	-1.2489160	-3.3020890
C	3.7158350	-2.5937110	-1.5062920
C	3.2841350	1.9706030	-2.0860490
C	3.8615700	1.8403900	0.3349150
V	-0.0419680	0.7063470	-0.4302690
C	0.1097490	2.3000830	0.1503120
C	0.2871500	3.5928550	0.9114460
C	1.2770160	4.5079080	0.1650080
C	0.8055700	3.3094430	2.3335010
C	-1.0498250	4.3480940	1.0187620
H	0.1353150	-3.4682840	1.7749230
H	-1.6254330	-5.1503800	1.6364950
H	-4.2496180	-2.3036730	-0.2064180
H	-3.5887380	2.2191890	-2.3096480
H	-3.2097610	1.3751730	-3.8235930
H	-1.9180340	1.7494580	-2.6518130
H	-1.3157260	-0.7605320	-2.8289680
H	-2.6929230	-1.0015240	-3.9185860
H	-2.5281000	-2.0082430	-2.4565150
H	-5.4865200	0.7384650	1.9546090
H	-4.6419350	-0.8000580	1.7083160
H	-5.5206500	-0.0341450	0.3599750
H	-4.7663280	2.2827400	-0.4352410
H	-3.1449440	2.9008440	-0.0107700
H	-4.4632110	2.9395910	1.1824350
H	4.5525510	-0.9919330	1.2026360

H	2.2850220	-2.0000620	4.7076430
H	0.1821830	-1.6082570	3.4670970
H	2.5137840	-2.1250200	-3.9693540
H	3.4985420	-0.7163450	-3.5393880
H	1.7217550	-0.5891450	-3.5588240
H	4.6755700	-2.0934040	-1.6826930
H	3.6952530	-3.4949660	-2.1419420
H	3.6888940	-2.9316340	-0.4597910
H	3.1689010	1.4513270	-3.0488480
H	4.0354890	2.7648870	-2.2291210
H	2.3247480	2.4467440	-1.8333640
H	4.4382380	2.7586220	0.1366130
H	4.3754220	1.2889010	1.1330230
H	2.8705270	2.1302040	0.7073780
H	1.3997370	5.4680880	0.6952800
H	0.9127780	4.7259350	-0.8514480
H	2.2698680	4.0480990	0.0731680
H	1.7730850	2.7863510	2.3152540
H	0.9358650	4.2450880	2.9048010
H	0.1006280	2.6681010	2.8848250
H	-0.9066220	5.3215360	1.5187090
H	-1.7878450	3.7811770	1.6030540
H	-1.4754600	4.5401010	0.0213380
C	-2.7730840	1.0987500	2.1657970
H	-2.5125760	0.1122220	2.5796370
H	-1.8338210	1.6338930	1.9699510
H	-3.3441990	1.6458140	2.9344260
C	5.1108040	0.4407070	-1.3180950
H	5.1147510	-0.0566970	-2.2980660
H	5.8430890	1.2636100	-1.3710110
H	5.4810990	-0.2729130	-0.5684020
C	1.2405290	-2.5602820	-1.6454400

H	1.1543020	-2.9443940	-0.6195960
H	0.3177570	-2.0053910	-1.8706780
H	1.2766370	-3.4290100	-2.3234280
C	-4.7075530	-0.2803650	-2.2091370
H	-4.9941250	-0.3098540	-3.2739610
H	-4.9554890	-1.2612310	-1.7812940
H	-5.3382790	0.4791490	-1.7261920
H	4.4910370	-1.7326490	3.5615140
H	-3.8613320	-4.5912400	0.6510730

5-6-TS

Symbol	X	Y	Z
C	1.0331580	-3.1844030	-1.4321160
C	1.1375860	-1.8550480	-0.9373530
C	2.4386580	-1.4255540	-0.5439350
C	2.1481550	-3.9729870	-1.6585460
C	3.4326240	-3.4905910	-1.3931020
C	3.5531870	-2.2304810	-0.8212400
N	0.0435470	-1.0302270	-0.7801860
P	2.5204300	0.1571320	0.3582670
C	3.3536630	-0.2752280	2.0167480
C	3.6743290	1.0192730	2.7758100
C	2.3091280	-1.0836500	2.8004110
C	3.5899370	1.3305080	-0.6683440
C	5.0848690	1.0076350	-0.7355290
C	3.4109600	2.7446350	-0.0964590
C	-3.5441850	-1.5105410	-1.5961280
C	-2.3853980	-1.1366060	-0.9057060
C	-1.1142450	-1.3026980	-1.5107990

C	-3.4907420	-1.9699330	-2.9073990
C	-2.2498900	-2.0298910	-3.5465880
C	-1.0868900	-1.7035630	-2.8644820
P	-2.3461180	-0.2858460	0.7086890
C	-3.8764850	0.8359670	0.8062530
C	-2.4482030	-1.6729590	1.9988220
C	-2.5823130	-1.0625050	3.3999760
C	-3.5688340	-2.6858850	1.7411760
C	-3.5364770	1.9388310	1.8221680
C	-4.0519730	1.4941640	-0.5696090
V	-0.0146960	0.7196000	0.3671100
C	-0.2950870	2.1331340	-0.5410800
C	-0.5876590	3.3733560	-1.3526890
C	-1.6150430	4.2658870	-0.6329570
C	-1.1278220	2.9389830	-2.7269510
C	0.6794220	4.2165820	-1.5786620
H	0.0463870	-3.5912880	-1.6544400
H	2.0124930	-4.9842400	-2.0515990
H	4.5496410	-1.8646960	-0.5767540
H	4.5561600	1.5264750	2.3582550
H	3.9060870	0.7785760	3.8265960
H	2.8351810	1.7284250	2.7762520
H	1.4156240	-0.4887670	3.0348430
H	2.7405580	-1.4148210	3.7595970
H	2.0009720	-1.9855750	2.2493820
H	5.5722100	1.7236290	-1.4186360
H	5.2849350	0.0014210	-1.1295000
H	5.5777640	1.1108820	0.2420090
H	3.9724610	2.8868020	0.8365340
H	2.3534270	2.9613780	0.0999120
H	3.7853430	3.4885500	-0.8189510
H	-4.5136850	-1.4295950	-1.1020100

H	-2.1877850	-2.3373270	-4.5936500
H	-0.1252930	-1.7612900	-3.3771830
H	-2.4732040	-1.8556260	4.1581380
H	-3.5615510	-0.5908120	3.5636610
H	-1.8034150	-0.3084640	3.5960100
H	-4.5718910	-2.2539690	1.8394410
H	-3.4933290	-3.5033480	2.4780550
H	-3.4818140	-3.1361020	0.7412150
H	-3.3205580	1.5418770	2.8246280
H	-4.3921410	2.6280330	1.9158400
H	-2.6667770	2.5198600	1.4890920
H	-4.6819530	2.3928540	-0.4666000
H	-4.5353780	0.8297980	-1.2967470
H	-3.0841800	1.7985140	-0.9871950
H	-1.8041570	5.1846510	-1.2131980
H	-1.2444400	4.5696040	0.3596030
H	-2.5769410	3.7570410	-0.4932270
H	-2.0463280	2.3418240	-2.6353020
H	-1.3524370	3.8172060	-3.3557510
H	-0.3872660	2.3189210	-3.2549400
H	0.4452300	5.0928330	-2.2064140
H	1.4632880	3.6391490	-2.0868640
H	1.0890360	4.5895100	-0.6276340
C	4.6274110	-1.1286830	1.9467210
H	5.0553310	-1.2004000	2.9609830
H	4.4163350	-2.1535170	1.6146610
H	5.4030860	-0.7033130	1.2973140
C	2.9926190	1.2615310	-2.0836100
H	1.9000040	1.3976730	-2.0676680
H	3.4344350	2.0557920	-2.7079620
H	3.2059020	0.2953220	-2.5641850
C	-5.1982220	0.1755390	1.2140710

H	-5.1827050	-0.2020630	2.2457900
H	-5.9983970	0.9325940	1.1606670
H	-5.4898050	-0.6491530	0.5490480
C	-1.1081800	-2.4194310	1.9193470
H	-0.9437730	-2.8775570	0.9337190
H	-0.2566750	-1.7578980	2.1278550
H	-1.0934550	-3.2238230	2.6734590
H	4.3177250	-4.0945650	-1.6004310
H	-4.4053050	-2.2517210	-3.4325790
C	0.0254390	1.9202910	2.3102220
H	-0.1412280	2.2892220	0.9852740
H	0.0594940	0.9628690	2.8558500
H	0.9315500	2.4849210	2.5756390
H	-0.8372720	2.4711630	2.7128940

6

Symbol	X	Y	Z
C	-0.7929540	-3.2772800	1.0386280
C	-1.0242650	-1.9384180	0.6420400
C	-2.3083850	-1.6254850	0.1225900
C	-1.7775020	-4.2482070	0.9261480
C	-3.0396280	-3.9318000	0.4223500
C	-3.2875600	-2.6201490	0.0315830
N	-0.0380470	-0.9597730	0.6857510
P	-2.5399440	0.1340250	-0.3157820
C	-3.6844800	0.2398060	-1.8280720
C	-3.5437250	1.6640750	-2.3972170
C	-3.1555940	-0.7705700	-2.8588950
C	-3.4170270	0.8167850	1.2256460

C	-4.5523210	-0.0726550	1.7460650
C	-3.9534410	2.2222940	0.9291370
C	3.4598830	-1.1267230	1.9168490
C	2.3659220	-0.8972570	1.0759750
C	1.0533620	-1.1377660	1.5465290
C	3.2837460	-1.5413550	3.2336260
C	1.9870110	-1.7246070	3.7181790
C	0.8897870	-1.5269590	2.8903330
P	2.4137780	-0.2345860	-0.6262340
C	3.8631230	0.9856520	-0.7521370
C	2.7482930	-1.7680460	-1.7038300
C	3.0218920	-1.3456480	-3.1537570
C	3.8987650	-2.6386970	-1.1827170
C	3.5322660	1.9144550	-1.9334160
C	3.8612830	1.8248350	0.5307300
V	-0.0067070	0.6531680	-0.5598830
C	0.1719810	2.3231090	0.0644090
C	0.3585380	3.5730860	0.8979460
C	1.3742160	4.5112330	0.2172400
C	0.8330460	3.2212020	2.3164640
C	-0.9746990	4.3360400	1.0100960
C	0.0394470	0.7067140	-2.6752820
H	0.1856230	-3.5591800	1.4288430
H	-1.5507790	-5.2725990	1.2328420
H	-4.2767720	-2.3652050	-0.3487080
H	-4.0272550	2.4142310	-1.7573020
H	-4.0327270	1.7126150	-3.3842150
H	-2.4938860	1.9575270	-2.5244110
H	-2.0804270	-0.6503320	-3.0410130
H	-3.6754930	-0.6145830	-3.8185410
H	-3.3330490	-1.8094120	-2.5475140
H	-4.9999620	0.4071310	2.6328450

H	-4.1867340	-1.0609830	2.0583090
H	-5.3577460	-0.2196490	1.0152400
H	-4.8605980	2.1965300	0.3088550
H	-3.2074330	2.8485830	0.4222190
H	-4.2221260	2.7211090	1.8750080
H	4.4725770	-0.9789510	1.5372740
H	1.8285780	-2.0257510	4.7567070
H	-0.1194270	-1.6837510	3.2760330
H	3.0836270	-2.2469310	-3.7859630
H	3.9742400	-0.8085490	-3.2654090
H	2.2190500	-0.7132630	-3.5576680
H	4.8739510	-2.1393990	-1.2273790
H	3.9694090	-3.5469270	-1.8048450
H	3.7241810	-2.9656960	-0.1472100
H	3.4832910	1.3771980	-2.8914280
H	4.3150260	2.6857540	-2.0250110
H	2.5688780	2.4207960	-1.7780260
H	4.4604960	2.7366570	0.3738530
H	4.2874700	1.2886310	1.3885910
H	2.8415300	2.1270010	0.7936610
H	1.5159070	5.4270590	0.8148360
H	1.0196700	4.8144120	-0.7811120
H	2.3562610	4.0370250	0.0933740
H	1.7977420	2.6956020	2.3139810
H	0.9486890	4.1344120	2.9229540
H	0.1099750	2.5678940	2.8265850
H	-0.8225650	5.3035060	1.5171780
H	-1.7102570	3.7678610	1.5945850
H	-1.4053800	4.5404370	0.0168830
H	0.0272360	-0.3273630	-3.0665690
H	-0.7928180	1.2379960	-3.1666350
H	-0.0369380	2.5556940	-1.0263210

C	-2.3391090	0.8988920	2.3158840
H	-1.9534050	-0.0967540	2.5805990
H	-1.4858220	1.5078340	1.9925630
H	-2.7697920	1.3467260	3.2271720
C	5.2637340	0.3953330	-0.9494000
H	5.3752330	-0.1302870	-1.9072200
H	5.9957770	1.2203860	-0.9470290
H	5.5518960	-0.2925320	-0.1420590
C	1.4722800	-2.6230780	-1.6697480
H	1.2631670	-3.0031280	-0.6610510
H	0.5846610	-2.0773230	-2.0168490
H	1.6088090	-3.4972810	-2.3277160
C	-5.1780940	-0.0283830	-1.5993470
H	-5.6993730	0.0549840	-2.5677570
H	-5.3863280	-1.0338460	-1.2105920
H	-5.6401230	0.7038530	-0.9239240
H	4.1484730	-1.7109140	3.8780100
H	-3.8172860	-4.6926540	0.3354220
H	0.9674520	1.1751790	-3.0453910

6’

Symbol	X	Y	Z
C	-0.6326440	-3.5179070	0.3605310
C	-0.9097780	-2.1314800	0.2782860
C	-2.2090270	-1.7508300	-0.1503270
C	-1.5905090	-4.4700590	0.0463770
C	-2.8698520	-4.0888190	-0.3616270
C	-3.1605480	-2.7319980	-0.4500490
N	0.0451810	-1.1592460	0.5405860

P	-2.4975820	0.0542990	-0.2035620
C	-3.6605550	0.4235840	-1.6626000
C	-3.5760550	1.9357540	-1.9361150
C	-3.0976380	-0.3356370	-2.8753350
C	-3.3905800	0.3694420	1.4475050
C	-4.4958930	-0.6499310	1.7506060
C	-3.9717700	1.7884000	1.4555990
C	3.5222240	-1.4002900	1.8113210
C	2.4318280	-1.0557380	1.0045610
C	1.1286340	-1.4761910	1.3644810
C	3.3505970	-2.1158890	2.9919200
C	2.0606540	-2.4899380	3.3754080
C	0.9683110	-2.1747830	2.5790730
P	2.4810420	-0.0466650	-0.5190840
C	3.8454350	1.2554130	-0.3209030
C	2.9335200	-1.2941100	-1.8801100
C	3.2183770	-0.5554050	-3.1946570
C	4.1167060	-2.1978260	-1.5136730
C	3.5187250	2.3654080	-1.3338720
C	3.7202640	1.8327050	1.0971080
V	0.0170400	0.6823090	-0.3786870
C	0.1171320	2.1445070	0.7110450
C	-0.0899790	3.6346490	0.9068260
C	-0.9182430	4.1938410	-0.2584100
C	1.2504930	4.3895800	0.9648500
C	-0.8246560	3.8933270	2.2347860
C	0.0432390	1.2358420	-2.3993530
H	0.3603340	-3.8467650	0.6694680
H	-1.3297380	-5.5294140	0.1147610
H	-4.1623960	-2.4303460	-0.7549650
H	-4.0483490	2.5284240	-1.1411240
H	-4.1071190	2.1665560	-2.8744320

H	-2.5396570	2.2776670	-2.0419840
H	-2.0218830	-0.1618390	-3.0079140
H	-3.6067180	0.0129140	-3.7889690
H	-3.2584060	-1.4196420	-2.7951640
H	-4.9776440	-0.3780640	2.7049080
H	-4.0914900	-1.6654870	1.8652740
H	-5.2820350	-0.6783010	0.9860580
H	-4.8769240	1.8715540	0.8373730
H	-3.2428830	2.5297940	1.0992020
H	-4.2543790	2.0655380	2.4846090
H	4.5292880	-1.1040850	1.5134120
H	1.9034820	-3.0328530	4.3107760
H	-0.0345790	-2.4788740	2.8853840
H	3.3125970	-1.2920740	-4.0095800
H	4.1595690	0.0114530	-3.1633790
H	2.4061750	0.1348160	-3.4647820
H	5.0570100	-1.6475510	-1.3860440
H	4.2738620	-2.9283260	-2.3251660
H	3.9221490	-2.7681400	-0.5935080
H	3.6114350	2.0246030	-2.3746120
H	4.2171190	3.2076800	-1.1972450
H	2.4957670	2.7378420	-1.1911170
H	4.2815690	2.7795810	1.1547690
H	4.1274360	1.1559550	1.8600670
H	2.6745900	2.0448940	1.3544740
H	-1.0687960	5.2813000	-0.1547470
H	-1.9082210	3.7163050	-0.3014300
H	-0.4169900	4.0072300	-1.2208020
H	1.8332970	4.2543870	0.0433710
H	1.0705760	5.4699760	1.0943920
H	1.8670160	4.0536390	1.8121360
H	-0.9969590	4.9724470	2.3836070

H	-0.2334850	3.5269920	3.0894410
H	-1.8012320	3.3915430	2.2661770
H	-0.8263140	1.8080170	-2.7613060
H	0.9370400	1.8567920	-2.5880990
H	0.1349710	0.3505740	-3.0572530
H	0.3912110	1.5889870	1.6404870
C	-2.3305300	0.2529140	2.5527860
H	-1.8455050	-0.7343320	2.5526690
H	-1.5474060	1.0139380	2.4496580
H	-2.8152830	0.3881340	3.5339060
C	-5.1429540	0.0622080	-1.4984090
H	-5.6305910	0.6355020	-0.6984190
H	-5.6681100	0.3106560	-2.4359980
H	-5.3160520	-1.0054630	-1.3105500
C	5.2902970	0.7934400	-0.5413360
H	5.4791550	0.4755210	-1.5756630
H	5.9654050	1.6412090	-0.3364640
H	5.5877470	-0.0246480	0.1292260
C	1.7021180	-2.1919370	-2.0754540
H	1.4736650	-2.7743780	-1.1728660
H	0.8031050	-1.6219450	-2.3481910
H	1.9048410	-2.9090850	-2.8881100
H	-3.6278570	-4.8350190	-0.6063620
H	4.2124670	-2.3721720	3.6109750

6’-7’-TS

Symbol	X	Y	Z
C	-0.5585070	-3.6112160	0.2451300
C	-0.8522430	-2.2260150	0.2087090

C	-2.1550560	-1.8467240	-0.2096110
C	-1.5055580	-4.5626450	-0.1033140
C	-2.7894390	-4.1823860	-0.4977800
C	-3.0961550	-2.8261470	-0.5413620
N	0.0794810	-1.2462750	0.5015010
P	-2.4423360	-0.0410020	-0.2000930
C	-3.6270630	0.3803440	-1.6256620
C	-3.5814980	1.9047270	-1.8315260
C	-3.0663340	-0.3152000	-2.8780850
C	-3.3015260	0.2215540	1.4798960
C	-4.3868440	-0.8195630	1.7803410
C	-3.8952980	1.6326660	1.5471020
C	3.5536990	-1.4156550	1.7858510
C	2.4561250	-1.0777710	0.9854200
C	1.1640820	-1.5593570	1.3163120
C	3.4032150	-2.1857940	2.9342440
C	2.1253280	-2.6240350	3.2907640
C	1.0269970	-2.3166180	2.5008730
P	2.4806820	-0.0142840	-0.5011510
C	3.8111510	1.3170470	-0.2571300
C	2.9498810	-1.1937300	-1.9153130
C	3.2739020	-0.4055510	-3.1910340
C	4.1205550	-2.1165400	-1.5563280
C	3.4883920	2.4415210	-1.2562400
C	3.6485550	1.8732130	1.1662390
V	-0.0089940	0.6898210	-0.3192990
C	-0.0885600	2.3226390	0.5581340
C	-0.3080110	3.8011870	0.8312260
C	-1.3274970	4.3680250	-0.1647390
C	1.0083240	4.5868170	0.6874170
C	-0.8272000	3.9737500	2.2668180
C	-0.0096130	1.3363780	-2.0990180

H	0.4392700	-3.9386980	0.5403700
H	-1.2327090	-5.6208500	-0.0730710
H	-4.1011580	-2.5275180	-0.8384230
H	-4.0873080	2.4463470	-1.0208840
H	-4.1024740	2.1605570	-2.7689890
H	-2.5564680	2.2857210	-1.8991020
H	-1.9911930	-0.1231360	-3.0036590
H	-3.5913590	0.0657030	-3.7697520
H	-3.2129170	-1.4037740	-2.8427820
H	-4.8492300	-0.5837940	2.7537600
H	-3.9681460	-1.8333030	1.8516410
H	-5.1902190	-0.8343150	1.0330550
H	-4.8064830	1.7327800	0.9402750
H	-3.1715290	2.3898260	1.2147970
H	-4.1712420	1.8671960	2.5886260
H	4.5513620	-1.0726940	1.5078920
H	1.9825150	-3.2110060	4.2016470
H	0.0349670	-2.6701260	2.7888770
H	3.4061940	-1.1152670	-4.0241360
H	4.2050500	0.1723990	-3.1099640
H	2.4585440	0.2769080	-3.4708230
H	5.0561310	-1.5732480	-1.3703200
H	4.3038420	-2.8057190	-2.3977480
H	3.8968540	-2.7310250	-0.6721760
H	3.6237370	2.1280590	-2.3004480
H	4.1612190	3.2965850	-1.0774230
H	2.4550790	2.7942270	-1.1434010
H	4.2339760	2.8025320	1.2602700
H	4.0061220	1.1738320	1.9336250
H	2.6014130	2.1153210	1.3924500
H	-1.4663270	5.4501170	-0.0100630
H	-2.3059070	3.8796260	-0.0554710

H	-0.9937190	4.2175500	-1.2040770
H	1.3995180	4.5261110	-0.3397680
H	0.8509560	5.6526810	0.9212740
H	1.7836770	4.2049450	1.3683940
H	-1.0131670	5.0369440	2.4905780
H	-0.0942360	3.5998620	2.9999020
H	-1.7672480	3.4260270	2.4251320
H	0.2510900	2.2835060	-0.7672180
H	0.8974260	1.4487750	-2.7181580
H	-0.8451080	1.8538430	-2.5982490
H	0.4451670	1.8014630	1.3969760
C	5.2687290	0.8846520	-0.4571800
H	5.4781830	0.5774160	-1.4906420
H	5.9259410	1.7423700	-0.2361560
H	5.5711410	0.0664330	0.2104180
C	1.7125940	-2.0638210	-2.1846850
H	1.4601480	-2.6951160	-1.3221970
H	0.8297200	-1.4565260	-2.4330750
H	1.9245630	-2.7343950	-3.0341180
C	-2.2120960	0.0924830	2.5560070
H	-1.7163610	-0.8884020	2.5239700
H	-1.4405350	0.8670360	2.4489810
H	-2.6722950	0.2042680	3.5519120
C	-5.1000330	-0.0152940	-1.4520750
H	-5.5823250	0.5114140	-0.6172740
H	-5.6465480	0.2673060	-2.3675950
H	-5.2523050	-1.0929900	-1.3112020
H	4.2705700	-2.4356170	3.5481920
H	-3.5385540	-4.9284670	-0.7690390

6'-7''-TS

Symbol	X	Y	Z
C	-0.8536960	-3.5421070	0.2955170
C	-1.0432940	-2.1388910	0.2304740
C	-2.3105000	-1.6794050	-0.2205660
C	-1.8556500	-4.4286390	-0.0693920
C	-3.0978500	-3.9667930	-0.5090880
C	-3.3088290	-2.5935960	-0.5719080
N	-0.0484850	-1.2254420	0.5250230
P	-2.5027020	0.1333840	-0.1870030
C	-3.6408530	0.6683200	-1.6072760
C	-3.4450460	2.1851430	-1.7904430
C	-3.1479960	-0.0700130	-2.8640440
C	-3.3575170	0.4068360	1.4959370
C	-4.5423450	-0.5374860	1.7366210
C	-3.8139400	1.8642870	1.6152970
C	3.4064800	-1.5976060	1.8064550
C	2.3318830	-1.2128470	0.9974990
C	1.0141470	-1.6051690	1.3419760
C	3.2093750	-2.3326330	2.9715290
C	1.9089330	-2.6889900	3.3351810
C	0.8304700	-2.3319570	2.5377120
P	2.4046650	-0.1897560	-0.5183210
C	3.8679360	1.0096390	-0.3649350
C	2.7350810	-1.4499760	-1.9081270
C	3.0737360	-0.7218760	-3.2149240
C	3.8415000	-2.4512170	-1.5550090
C	3.6561660	2.0976520	-1.4311730
C	3.7584210	1.6623550	1.0210790
V	0.0333710	0.7071590	-0.3231740

C	0.2252430	2.3312490	0.5555410
C	0.3577290	3.8191220	0.8530380
C	-1.0286870	4.4857070	0.7761410
C	1.2827940	4.5019900	-0.1614700
C	0.9156630	4.0161480	2.2708440
C	0.0503440	1.3504800	-2.1008040
H	0.1093370	-3.9361940	0.6222810
H	-1.6583680	-5.5026690	-0.0176800
H	-4.2827870	-2.2264910	-0.8971020
H	-3.8492620	2.7582960	-0.9448070
H	-3.9820930	2.5145420	-2.6952740
H	-2.3887730	2.4571410	-1.9076680
H	-2.0549950	-0.0309320	-2.9697010
H	-3.5944460	0.3962260	-3.7579350
H	-3.4426160	-1.1285400	-2.8561290
H	-4.9878210	-0.3048260	2.7184780
H	-4.2237710	-1.5890340	1.7616710
H	-5.3370150	-0.4375750	0.9875360
H	-4.6822780	2.0854080	0.9786170
H	-3.0078200	2.5627730	1.3523110
H	-4.1120710	2.0734890	2.6559190
H	4.4229610	-1.3217890	1.5241740
H	1.7323400	-3.2498330	4.2565460
H	-0.1810850	-2.6196740	2.8311680
H	3.0959530	-1.4558070	-4.0372030
H	4.0598920	-0.2378910	-3.1890170
H	2.3130260	0.0321970	-3.4671370
H	4.8229180	-1.9811760	-1.4157440
H	3.9415760	-3.1789910	-2.3779370
H	3.5995600	-3.0181610	-0.6440410
H	3.7943530	1.7198910	-2.4525010
H	4.3900150	2.9068910	-1.2798150

H	2.6518370	2.5314430	-1.3629400
H	4.4214740	2.5422580	1.0657130
H	4.0532770	0.9813700	1.8311560
H	2.7295650	1.9954070	1.2157020
H	-0.9512820	5.5724420	0.9454970
H	-1.7058630	4.0813540	1.5430050
H	-1.4941460	4.3286720	-0.2097360
H	0.9650330	4.2971860	-1.1968140
H	1.2743340	5.5942440	-0.0183310
H	2.3196830	4.1583000	-0.0489260
H	0.9907630	5.0887540	2.5135500
H	1.9182790	3.5766130	2.3775640
H	0.2613770	3.5476530	3.0237050
H	-0.1333330	2.3124460	-0.7533020
H	0.9857840	1.7086960	-2.5657690
H	-0.7965530	1.6269520	-2.7492200
H	-0.2329170	1.7763760	1.4149760
C	5.2760240	0.4283150	-0.5423820
H	5.4495280	0.0550990	-1.5608800
H	6.0116260	1.2308400	-0.3649430
H	5.5069390	-0.3813140	0.1628810
C	1.4342710	-2.2349470	-2.1263710
H	1.1658440	-2.8362500	-1.2485110
H	0.5911490	-1.5695250	-2.3607060
H	1.5740470	-2.9303260	-2.9707810
C	-2.3152980	0.1223170	2.5891800
H	-1.9104490	-0.8967750	2.5192700
H	-1.4717600	0.8257900	2.5620300
H	-2.7954110	0.2247190	3.5763070
C	-5.1442020	0.4091830	-1.4426410
H	-5.5829170	0.9861380	-0.6170940
H	-5.6563450	0.7285330	-2.3657960

H	-5.3897700	-0.6502480	-1.2911960
H	4.0604740	-2.6195380	3.5920980
H	-3.8875210	-4.6631350	-0.7966320

7’

Symbol	X	Y	Z
C	-0.6769300	-3.5713130	-0.2883340
C	-0.9476170	-2.2003990	-0.0759240
C	-2.2621840	-1.7440580	-0.3439430
C	-1.6647160	-4.4456950	-0.7186530
C	-2.9644900	-3.9946930	-0.9536920
C	-3.2462140	-2.6461880	-0.7609300
N	0.0325220	-1.2943790	0.3289390
P	-2.5034300	0.0426820	-0.0591210
C	-3.7548000	0.6837330	-1.3410790
C	-3.5444690	2.2038440	-1.4228060
C	-3.3935720	0.0736510	-2.7040410
C	-3.2423210	0.0672150	1.6973370
C	-4.4024660	-0.9141900	1.9036810
C	-3.6873950	1.4884220	2.0553490
C	3.4596260	-1.7790870	1.6759630
C	2.3998300	-1.2790610	0.9118090
C	1.0854230	-1.7622240	1.1211240
C	3.2485830	-2.7181540	2.6807870
C	1.9474360	-3.1623800	2.9237100
C	0.8861420	-2.6966350	2.1596370
P	2.5091170	0.0031100	-0.3817480
C	3.8516570	1.2484950	0.1357810
C	3.0515160	-0.9660270	-1.9273750

C	3.3013130	0.0175270	-3.0771960
C	4.2792640	-1.8581160	-1.7046290
C	3.5319600	2.5428110	-0.6288370
C	3.7243340	1.5201730	1.6451200
V	-0.0136330	0.6108830	-0.4187320
C	0.0689520	2.3276520	0.8280390
C	-0.1037870	3.8621940	0.6147560
C	0.1020060	4.3139820	-0.8392540
C	0.9130770	4.6053520	1.5013700
C	-1.5081250	4.3109930	1.0369640
C	-0.0915550	0.9560260	-2.1711260
H	0.3302020	-3.9513120	-0.1121720
H	-1.4122660	-5.4972320	-0.8775190
H	-4.2619910	-2.2892940	-0.9366910
H	-3.8295560	2.7107800	-0.4900610
H	-4.1714070	2.6215250	-2.2279120
H	-2.4952580	2.4481250	-1.6424330
H	-2.3227790	0.1837650	-2.9215820
H	-3.9563750	0.6007450	-3.4920630
H	-3.6473870	-0.9928010	-2.7688670
H	-4.7188120	-0.8723840	2.9596820
H	-4.1032610	-1.9511260	1.6943440
H	-5.2827010	-0.6788580	1.2932850
H	-4.5554980	1.8196520	1.4675140
H	-2.8782060	2.2153750	1.9122870
H	-3.9816950	1.5233850	3.1173990
H	4.4753360	-1.4295200	1.4829590
H	1.7555860	-3.8836770	3.7220530
H	-0.1223590	-3.0639400	2.3577060
H	3.4476090	-0.5440940	-4.0146580
H	4.2035350	0.6264440	-2.9204250
H	2.4442150	0.6932950	-3.2212890

H	5.2005520	-1.2961930	-1.5147980
H	4.4489730	-2.4602790	-2.6128300
H	4.1234960	-2.5618810	-0.8736340
H	3.5956620	2.4142480	-1.7187210
H	4.2474970	3.3319000	-0.3437680
H	2.5224420	2.8985470	-0.3939800
H	4.3153440	2.4164720	1.8959600
H	4.1090130	0.6892710	2.2506600
H	2.6914190	1.7101760	1.9655030
H	0.0787100	5.4141070	-0.9132100
H	-0.6954740	3.9276060	-1.4925700
H	1.0656180	3.9790240	-1.2506810
H	1.9483210	4.3832430	1.1969100
H	0.7776900	5.6993610	1.4488980
H	0.8061960	4.3027570	2.5557200
H	-1.6471270	5.3922570	0.8701280
H	-1.6856440	4.1151970	2.1067160
H	-2.2807580	3.7861010	0.4589850
H	1.0961400	2.1641570	1.1950390
H	0.0104930	2.0466040	-1.9495980
H	-0.1384780	0.7308220	-3.2421980
H	-0.5687380	2.0361140	1.6848140
C	-2.1068640	-0.3514770	2.6464930
H	-1.7705180	-1.3789070	2.4497980
H	-1.2317830	0.3090470	2.5786990
H	-2.4760400	-0.3158450	3.6848660
C	-5.2366010	0.4121040	-1.0539190
H	-5.5875270	0.9107670	-0.1401450
H	-5.8354890	0.8131750	-1.8886360
H	-5.4722320	-0.6580730	-0.9748660
C	5.3061860	0.8576020	-0.1520600
H	5.5145780	0.7701310	-1.2268150

H	5.9650290	1.6503150	0.2403790
H	5.6037820	-0.0802910	0.3367310
C	1.8817150	-1.8892860	-2.3039310
H	1.6805940	-2.6291890	-1.5166820
H	0.9548060	-1.3342390	-2.4952120
H	2.1463260	-2.4445170	-3.2189950
H	4.0864660	-3.0926180	3.2718300
H	-3.7451620	-4.6815210	-1.2857000

7”

Symbol	X	Y	Z
C	-0.6769300	-3.5713130	-0.2883340
C	-0.9476170	-2.2003990	-0.0759240
C	-2.2621840	-1.7440580	-0.3439430
C	-1.6647160	-4.4456950	-0.7186530
C	-2.9644900	-3.9946930	-0.9536920
C	-3.2462140	-2.6461880	-0.7609300
N	0.0325220	-1.2943790	0.3289390
P	-2.5034300	0.0426820	-0.0591210
C	-3.7548000	0.6837330	-1.3410790
C	-3.5444690	2.2038440	-1.4228060
C	-3.3935720	0.0736510	-2.7040410
C	-3.2423210	0.0672150	1.6973370
C	-4.4024660	-0.9141900	1.9036810
C	-3.6873950	1.4884220	2.0553490
C	3.4596260	-1.7790870	1.6759630
C	2.3998300	-1.2790610	0.9118090
C	1.0854230	-1.7622240	1.1211240
C	3.2485830	-2.7181540	2.6807870

C	1.9474360	-3.1623800	2.9237100
C	0.8861420	-2.6966350	2.1596370
P	2.5091170	0.0031100	-0.3817480
C	3.8516570	1.2484950	0.1357810
C	3.0515160	-0.9660270	-1.9273750
C	3.3013130	0.0175270	-3.0771960
C	4.2792640	-1.8581160	-1.7046290
C	3.5319600	2.5428110	-0.6288370
C	3.7243340	1.5201730	1.6451200
V	-0.0136330	0.6108830	-0.4187320
C	0.0689520	2.3276520	0.8280390
C	-0.1037870	3.8621940	0.6147560
C	0.1020060	4.3139820	-0.8392540
C	0.9130770	4.6053520	1.5013700
C	-1.5081250	4.3109930	1.0369640
C	-0.0915550	0.9560260	-2.1711260
H	0.3302020	-3.9513120	-0.1121720
H	-1.4122660	-5.4972320	-0.8775190
H	-4.2619910	-2.2892940	-0.9366910
H	-3.8295560	2.7107800	-0.4900610
H	-4.1714070	2.6215250	-2.2279120
H	-2.4952580	2.4481250	-1.6424330
H	-2.3227790	0.1837650	-2.9215820
H	-3.9563750	0.6007450	-3.4920630
H	-3.6473870	-0.9928010	-2.7688670
H	-4.7188120	-0.8723840	2.9596820
H	-4.1032610	-1.9511260	1.6943440
H	-5.2827010	-0.6788580	1.2932850
H	-4.5554980	1.8196520	1.4675140
H	-2.8782060	2.2153750	1.9122870
H	-3.9816950	1.5233850	3.1173990
H	4.4753360	-1.4295200	1.4829590

H	1.7555860	-3.8836770	3.7220530
H	-0.1223590	-3.0639400	2.3577060
H	3.4476090	-0.5440940	-4.0146580
H	4.2035350	0.6264440	-2.9204250
H	2.4442150	0.6932950	-3.2212890
H	5.2005520	-1.2961930	-1.5147980
H	4.4489730	-2.4602790	-2.6128300
H	4.1234960	-2.5618810	-0.8736340
H	3.5956620	2.4142480	-1.7187210
H	4.2474970	3.3319000	-0.3437680
H	2.5224420	2.8985470	-0.3939800
H	4.3153440	2.4164720	1.8959600
H	4.1090130	0.6892710	2.2506600
H	2.6914190	1.7101760	1.9655030
H	0.0787100	5.4141070	-0.9132100
H	-0.6954740	3.9276060	-1.4925700
H	1.0656180	3.9790240	-1.2506810
H	1.9483210	4.3832430	1.1969100
H	0.7776900	5.6993610	1.4488980
H	0.8061960	4.3027570	2.5557200
H	-1.6471270	5.3922570	0.8701280
H	-1.6856440	4.1151970	2.1067160
H	-2.2807580	3.7861010	0.4589850
H	1.0961400	2.1641570	1.1950390
H	0.0104930	2.0466040	-1.9495980
H	-0.1384780	0.7308220	-3.2421980
H	-0.5687380	2.0361140	1.6848140
C	-2.1068640	-0.3514770	2.6464930
H	-1.7705180	-1.3789070	2.4497980
H	-1.2317830	0.3090470	2.5786990
H	-2.4760400	-0.3158450	3.6848660
C	-5.2366010	0.4121040	-1.0539190

H	-5.5875270	0.9107670	-0.1401450
H	-5.8354890	0.8131750	-1.8886360
H	-5.4722320	-0.6580730	-0.9748660
C	5.3061860	0.8576020	-0.1520600
H	5.5145780	0.7701310	-1.2268150
H	5.9650290	1.6503150	0.2403790
H	5.6037820	-0.0802910	0.3367310
C	1.8817150	-1.8892860	-2.3039310
H	1.6805940	-2.6291890	-1.5166820
H	0.9548060	-1.3342390	-2.4952120
H	2.1463260	-2.4445170	-3.2189950
H	4.0864660	-3.0926180	3.2718300
H	-3.7451620	-4.6815210	-1.2857000

V^{IV} (L_2)

1

Symbol	X	Y	Z
C	0.0061050	-3.7033620	0.5520410
C	-0.5141200	-2.4058030	0.6925990
C	-1.8558460	-2.1908920	0.3028610
C	-0.7711840	-4.7403510	0.0506390
C	-2.1124700	-4.5418670	-0.3060900
C	-2.6369480	-3.2556080	-0.1625450
P	0.3694990	-0.9921400	1.4616590
C	-4.0299420	-0.3902280	-0.5161910
C	-4.7930340	0.9145160	-0.2763110
C	-3.8198750	-0.6252840	-2.0143710
C	-2.9100360	-0.3228430	2.2449960
C	-4.1543290	-1.1168200	2.6304990
C	-2.9417450	1.1290450	2.7257980

C	-2.9473540	-5.6798010	-0.8314630
C	4.0878740	-0.9960960	-0.3669430
C	2.7259900	-0.8044780	-0.1134450
C	2.1375350	-1.3351090	1.0482530
C	4.9010100	-1.7100090	0.5192370
C	4.3096330	-2.2391750	1.6721710
C	2.9547720	-2.0514260	1.9326170
C	6.3706960	-1.8999710	0.2525030
C	2.6688920	1.1276890	-2.3140680
C	0.9938650	-1.3440170	-2.3167970
C	-0.3384390	-1.0375320	-2.9972520
C	2.0456770	-1.8872190	-3.2795780
C	1.9097920	1.7312090	-3.4955940
C	3.3456560	2.2207950	-1.4874410
V	-0.2673500	1.0317720	0.1100390
C	0.7169920	1.9632380	1.2377870
C	1.4535330	2.5048440	2.4340570
C	1.1187800	3.9953260	2.6157310
C	2.9694830	2.3438130	2.2197270
C	1.0385420	1.7374780	3.6964570
C	-1.3048280	2.2374080	-1.3593540
C	-1.5331350	3.7619000	-1.2236310
C	-0.2309500	4.5471920	-1.4245980
C	-2.5361230	4.2294940	-2.2943590
C	-2.1104590	4.0925540	0.1599860
H	1.0433900	-3.9002240	0.8359730
H	-0.3295630	-5.7354350	-0.0600000
H	-3.6800480	-3.0876730	-0.4435320
H	-4.6446940	-1.2153860	-0.1191120
H	-5.0980630	1.0394280	0.7711180
H	-5.7107820	0.9222600	-0.8856270
H	-4.1985440	1.7940930	-0.5643820

H	-3.2711260	0.2074620	-2.4792030
H	-4.7978510	-0.6973470	-2.5166590
H	-3.2705080	-1.5531070	-2.2254030
H	-2.0402860	-0.7982630	2.7296170
H	-4.2637100	-1.1320710	3.7267670
H	-4.0900860	-2.1628750	2.2929250
H	-5.0767620	-0.6779730	2.2189470
H	-3.7874300	1.6960910	2.3095490
H	-2.0143100	1.6587100	2.4555700
H	-3.0333530	1.1589840	3.8233360
H	-3.0097530	-6.5012670	-0.1001560
H	-2.5174160	-6.1010150	-1.7544000
H	-3.9735570	-5.3568670	-1.0588080
H	4.5444570	-0.5791970	-1.2685250
H	4.9243630	-2.7968320	2.3846950
H	2.5183610	-2.4542900	2.8506830
H	6.6662830	-1.4673110	-0.7138390
H	6.6383760	-2.9680800	0.2403530
H	6.9807930	-1.4217090	1.0349760
H	3.4464860	0.4573750	-2.7163830
H	0.7952670	-2.1202700	-1.5610450
H	-0.7423260	-1.9492200	-3.4656410
H	-0.2517000	-0.2686660	-3.7798160
H	-1.0795090	-0.6841800	-2.2682020
H	2.2717650	-1.1796370	-4.0925400
H	1.6781720	-2.8142060	-3.7481320
H	2.9865230	-2.1333960	-2.7642530
H	1.4915490	0.9681260	-4.1666860
H	2.5914170	2.3556640	-4.0948880
H	1.0846090	2.3728110	-3.1548980
H	4.0665980	2.7727140	-2.1113990
H	3.8890280	1.8151390	-0.6224860

H	2.6065180	2.9458840	-1.1137290
H	1.6757980	4.4179360	3.4684140
H	0.0446670	4.1404470	2.8096890
H	1.3819840	4.5798940	1.7195040
H	3.3146200	2.9405260	1.3618030
H	3.5248090	2.6833600	3.1100060
H	3.2306420	1.2906710	2.0327400
H	1.5487620	2.1513170	4.5817540
H	1.2915700	0.6700890	3.6186840
H	-0.0473910	1.8058080	3.8640660
H	0.8049620	2.5967340	0.2930890
H	-0.8215000	2.0465450	-2.3390130
H	-2.3030500	1.7823930	-1.4735260
H	-0.4009590	5.6334390	-1.3379620
H	0.1983070	4.3591830	-2.4224290
H	0.5294500	4.2770760	-0.6761540
H	-2.7151350	5.3178870	-2.2473360
H	-3.5091880	3.7263320	-2.1688180
H	-2.1708720	3.9925080	-3.3072170
H	-1.4000280	3.8195390	0.9550880
H	-3.0439260	3.5350620	0.3422470
H	-2.3403420	5.1670580	0.2609870
P	-2.4283100	-0.4542780	0.4411970
P	1.5740930	0.0714560	-1.2394450

1-2-TS

Symbol	X	Y	Z
C	-0.7649790	3.7481260	0.2745150
C	-0.0040570	2.5941790	0.5276630

C	1.3546920	2.6041620	0.1250710
C	-0.2065180	4.8641970	-0.3370090
C	1.1457000	4.8959970	-0.7032030
C	1.9082280	3.7521830	-0.4549960
P	-0.5951690	1.1131320	1.4389410
C	3.8027250	1.1893960	-0.6795620
C	4.7891120	0.0576950	-0.3857350
C	3.4372850	1.2240310	-2.1660720
C	2.8625470	1.1944920	2.1593050
C	3.9204780	2.2717550	2.3783220
C	3.2371470	-0.1559200	2.7700660
C	1.7406650	6.1149360	-1.3584730
C	-4.3772880	0.1813590	0.0183840
C	-2.9889310	0.2657750	0.1573600
C	-2.4213530	1.0938490	1.1458290
C	-5.2404050	0.9127270	0.8421790
C	-4.6725180	1.7408660	1.8156700
C	-3.2903390	1.8199060	1.9705240
C	-6.7344040	0.7892810	0.7005910
C	-2.7534440	-1.8624390	-1.8560340
C	-1.5625440	0.8627610	-2.2102910
C	-0.2078460	0.7777190	-2.9120350
C	-2.7288260	1.0820540	-3.1680290
C	-1.9904730	-2.4233590	-3.0563690
C	-3.1092600	-2.9795310	-0.8755680
V	0.4063200	-0.7686640	0.1416900
C	0.2144330	-1.9923350	1.2955620
C	-0.0115170	-2.9237440	2.4548750
C	1.2204840	-3.7971610	2.7413810
C	-1.2297140	-3.8270000	2.2005690
C	-0.3003280	-2.0401800	3.6826100
C	1.4646250	-2.1187930	-1.3609690

C	2.0475280	-3.5481260	-1.4535490
C	0.9645610	-4.5967160	-1.1681280
C	2.5953240	-3.7807330	-2.8697090
C	3.1964770	-3.6959980	-0.4474280
H	-1.8194660	3.7685520	0.5625280
H	-0.8342060	5.7400720	-0.5279320
H	2.9619760	3.7616130	-0.7464690
H	4.2888040	2.1435780	-0.4166540
H	5.2302190	0.1378420	0.6170590
H	5.6186510	0.0831580	-1.1101670
H	4.3077220	-0.9290420	-0.4632710
H	3.0184710	0.2627630	-2.5012550
H	4.3400400	1.4155030	-2.7678120
H	2.7066610	2.0099400	-2.4040830
H	1.9307050	1.5198480	2.6525330
H	4.0810670	2.4269500	3.4572620
H	3.6158250	3.2387640	1.9492990
H	4.8930080	1.9915210	1.9432520
H	4.1888670	-0.5476100	2.3804520
H	2.4545820	-0.9012310	2.5644870
H	3.3424730	-0.0589600	3.8625020
H	2.8179100	5.9906570	-1.5409680
H	1.6087890	7.0123230	-0.7334390
H	1.2601500	6.3237420	-2.3280190
H	-4.8155380	-0.4608410	-0.7503510
H	-5.3240110	2.3232480	2.4734410
H	-2.8698040	2.4508390	2.7583840
H	-7.0219040	0.5063570	-0.3225780
H	-7.2409960	1.7336540	0.9480460
H	-7.1319930	0.0168380	1.3794140
H	-3.6805660	-1.3960950	-2.2287960
H	-1.5142490	1.7345220	-1.5387770

H	-0.0254560	1.6889620	-3.5036760
H	-0.1289930	-0.0860990	-3.5902260
H	0.6109970	0.7009000	-2.1784270
H	-2.8259010	0.2706260	-3.9057980
H	-2.5813050	2.0179510	-3.7306440
H	-3.6843360	1.1728250	-2.6289890
H	-1.7438550	-1.6567520	-3.8042280
H	-2.6012010	-3.1890490	-3.5608370
H	-1.0538730	-2.9047980	-2.7411880
H	-3.7260780	-3.7434450	-1.3755780
H	-3.6706640	-2.6115780	-0.0041070
H	-2.1967560	-3.4680760	-0.5029260
H	1.0553340	-4.4117690	3.6419880
H	2.1160380	-3.1807110	2.9143750
H	1.4341820	-4.4828030	1.9077350
H	-1.0744820	-4.4778210	1.3261560
H	-1.4215990	-4.4764480	3.0713980
H	-2.1298130	-3.2214010	2.0193240
H	-0.5058060	-2.6635780	4.5691690
H	-1.1727060	-1.3931780	3.5047700
H	0.5535820	-1.3849150	3.9107530
H	0.8821570	-2.3589140	-0.0032820
H	0.7579820	-1.9774200	-2.1956230
H	2.2972310	-1.4191900	-1.5372900
H	1.3675690	-5.6193840	-1.2475030
H	0.1313130	-4.5119280	-1.8838190
H	0.5438220	-4.4814090	-0.1584700
H	3.0567260	-4.7780870	-2.9664710
H	3.3609540	-3.0298800	-3.1247320
H	1.7937360	-3.7074390	-3.6228420
H	2.8885010	-3.3811250	0.5602070
H	4.0554530	-3.0718440	-0.7415490

H	3.5522160	-4.7374830	-0.3868150
P	2.2834630	1.0358550	0.3893100
P	-1.7894360	-0.5367490	-0.9777000

A

Symbol	X	Y	Z
C	1.7467960	-2.7108920	1.6056400
C	1.5162350	-1.4001300	1.1548220
C	2.6036360	-0.7298860	0.5438550
C	2.9523900	-3.3655760	1.3791530
C	4.0116660	-2.7238590	0.7290350
C	3.8191480	-1.3949460	0.3448160
P	-0.0414790	-0.4688180	1.4712580
C	3.1572750	1.9613260	1.5123260
C	2.9403060	3.4634970	1.3187230
C	2.6216760	1.4999960	2.8663990
C	3.5465150	1.3598800	-1.3566420
C	4.9947620	1.7086000	-1.0084170
C	2.9471110	2.4050270	-2.3011450
C	5.3053030	-3.4424700	0.4524460
C	-3.7968910	-1.4444160	0.1428790
C	-2.6167390	-0.7711230	0.4736780
C	-1.4780090	-1.4851060	0.9241330
C	-3.8922770	-2.8367690	0.1926210
C	-2.7558410	-3.5409670	0.6034270
C	-1.5892750	-2.8829860	0.9781480
C	-5.1595980	-3.5569820	-0.1854430
C	-3.8051460	1.6199530	-0.8166670
C	-2.8801520	1.6389600	2.0210240

C	-2.2740390	3.0079380	2.3337900
C	-4.3650740	1.5504610	2.3605520
C	-3.8587880	3.1493670	-0.8456740
C	-3.6086570	1.0676630	-2.2270850
V	-0.0340450	1.2698680	-0.2759530
C	-0.0031300	0.3934990	-1.7186800
C	0.0490340	-0.6097840	-2.8422600
C	-0.4899830	0.0068780	-4.1449850
C	-0.7874330	-1.8456930	-2.4716040
C	1.5019300	-1.0582980	-3.0790820
H	0.9736250	-3.2293570	2.1740950
H	3.0781380	-4.3935840	1.7315930
H	4.6492270	-0.8757450	-0.1389650
H	4.2381390	1.7489130	1.4882210
H	3.3526180	3.8347270	0.3685830
H	3.4197500	4.0333620	2.1307200
H	1.8652850	3.7124900	1.3338440
H	1.5365680	1.6628880	2.9592460
H	3.1157860	2.0638790	3.6742550
H	2.8089230	0.4308370	3.0423150
H	3.5397560	0.3941510	-1.8850380
H	5.6003660	1.7241220	-1.9286950
H	5.4642320	0.9914020	-0.3205710
H	5.0782240	2.7071970	-0.5527030
H	2.9341500	3.4072150	-1.8416020
H	1.9146340	2.1418830	-2.5756440
H	3.5459960	2.4761500	-3.2236290
H	5.6318760	-4.0332640	1.3217760
H	6.1126550	-2.7407490	0.1975190
H	5.1952310	-4.1419310	-0.3925060
H	-4.6700990	-0.8774930	-0.1911600
H	-2.7854760	-4.6340460	0.6427820

H	-0.7423870	-3.4864960	1.3016400
H	-5.9405510	-2.8551620	-0.5121230
H	-5.5619960	-4.1331220	0.6631920
H	-4.9857090	-4.2696740	-1.0071490
H	-4.7578190	1.2469540	-0.4059990
H	-2.3436760	0.9035430	2.6447900
H	-2.4230720	3.2591560	3.3962470
H	-2.7230840	3.8177780	1.7389220
H	-1.1870160	3.0158820	2.1502150
H	-4.9642680	2.2881690	1.8036840
H	-4.5236850	1.7456340	3.4334150
H	-4.7689680	0.5490880	2.1464890
H	-4.1420570	3.5834530	0.1233300
H	-4.6035680	3.4852680	-1.5844520
H	-2.8866500	3.5772940	-1.1426850
H	-4.4454230	1.3735710	-2.8753490
H	-3.5500330	-0.0297760	-2.2453030
H	-2.6741780	1.4546450	-2.6589950
H	-0.3854080	-0.6997610	-4.9864750
H	0.0586020	0.9264290	-4.4043270
H	-1.5556700	0.2630340	-4.0560550
H	-1.8291100	-1.5719020	-2.2469890
H	-0.7942420	-2.5786330	-3.2970990
H	-0.3771640	-2.3371590	-1.5776240
H	1.5422270	-1.8483170	-3.8484260
H	1.9460960	-1.4601170	-2.1555570
H	2.1223360	-0.2200000	-3.4320910
P	-2.4332070	1.0450330	0.2995620
P	2.3789110	1.0365330	0.0777530

5-6-TS

Symbol	X	Y	Z
C	1.8641360	-2.8825740	1.5527630
C	1.5962720	-1.5648560	1.1421620
C	2.6445690	-0.8542490	0.5158950
C	3.0743290	-3.5058440	1.2746920
C	4.0974090	-2.8237670	0.6055940
C	3.8657820	-1.4918380	0.2580840
P	0.0120620	-0.7191490	1.5564810
C	3.1240180	1.8206420	1.5822880
C	2.8932440	3.3255520	1.4288450
C	2.5947080	1.3210910	2.9250410
C	3.5154770	1.3060750	-1.3037960
C	4.9656840	1.6365430	-0.9408710
C	2.9422560	2.3886720	-2.2197850
C	5.4003200	-3.5056960	0.2827410
C	-3.6478910	-1.7079640	-0.0290260
C	-2.5283740	-1.0202550	0.4465590
C	-1.3360170	-1.7140720	0.7759350
C	-3.6259490	-3.0867920	-0.2523950
C	-2.4324480	-3.7611850	0.0257140
C	-1.3234940	-3.0973640	0.5416450
C	-4.8291680	-3.8208500	-0.7825130
C	-3.9069550	1.4956890	-0.3387680
C	-2.7984570	1.0930950	2.3941170
C	-2.2073350	2.4125320	2.8923030
C	-4.2542770	0.8831710	2.8008080
C	-4.0521270	2.9961590	-0.0692540
C	-3.7810060	1.2373430	-1.8385150
V	-0.0822680	1.1449330	-0.1327890

C	-0.1359480	0.4900210	-1.6915880
C	-0.1622340	-0.1849490	-3.0333600
C	-0.5973190	0.7934660	-4.1353940
C	-1.1427200	-1.3687990	-2.9590130
C	1.2387120	-0.7327610	-3.3602320
H	1.1103920	-3.4275380	2.1249790
H	3.2341190	-4.5390210	1.5967810
H	4.6695460	-0.9479590	-0.2411090
H	4.2062800	1.6171350	1.5508210
H	3.2790600	3.7218020	0.4778560
H	3.3927150	3.8759980	2.2421670
H	1.8190840	3.5683110	1.4754160
H	1.5099790	1.4820580	3.0263700
H	3.0901960	1.8657620	3.7450280
H	2.7857270	0.2485770	3.0745800
H	3.5031420	0.3587040	-1.8668440
H	5.5756740	1.6694330	-1.8577550
H	5.4300190	0.9053760	-0.2649460
H	5.0493570	2.6252600	-0.4641920
H	2.9517220	3.3779670	-1.7358360
H	1.9078800	2.1645540	-2.5102410
H	3.5487860	2.4668130	-3.1365420
H	5.8321200	-3.9882080	1.1733410
H	6.1426240	-2.7960130	-0.1101460
H	5.2583820	-4.2929670	-0.4751900
H	-4.5637430	-1.1585010	-0.2640810
H	-2.3688990	-4.8381020	-0.1576840
H	-0.4226420	-3.6765230	0.7454310
H	-4.5865000	-4.3794500	-1.7003000
H	-5.6513330	-3.1298100	-1.0190070
H	-5.2086360	-4.5513070	-0.0497730
H	-4.8089750	0.9877470	0.0418470

H	-2.2035330	0.2887250	2.8585020
H	-2.3275150	2.4952650	3.9845220
H	-2.6841300	3.2952980	2.4402360
H	-1.1269690	2.4666770	2.6818550
H	-4.9186730	1.6729050	2.4171470
H	-4.3449380	0.8882010	3.8989960
H	-4.6345160	-0.0872040	2.4456070
H	-4.2967060	3.2193570	0.9781260
H	-4.8661820	3.4072430	-0.6870560
H	-3.1324990	3.5430470	-0.3286100
H	-4.6515970	1.6549120	-2.3692720
H	-3.7250200	0.1666080	-2.0761470
H	-2.8735040	1.7145870	-2.2369170
H	-0.6002110	0.2982420	-5.1207920
H	0.0862180	1.6558950	-4.1954950
H	-1.6117770	1.1777240	-3.9507890
H	-2.1614360	-1.0282630	-2.7295940
H	-1.1700030	-1.9141550	-3.9176400
H	-0.8451320	-2.0716880	-2.1667310
H	1.2151490	-1.3307420	-4.2868240
H	1.6046210	-1.3760370	-2.5451400
H	1.9616060	0.0827450	-3.5103870
C	-0.2639400	3.2683830	-0.8264900
H	-0.2225000	2.0400350	-1.5434060
H	0.6672780	3.7896620	-1.0933660
H	-0.5101940	3.5330890	0.2193310
H	-1.0703770	3.6976660	-1.4425380
P	-2.4423310	0.8038430	0.5783970
P	2.3570100	0.9225680	0.1267510

6

Symbol	X	Y	Z
C	1.7080480	2.8994200	-1.4866760
C	1.4917820	1.5671410	-1.0947970
C	2.5898690	0.8831780	-0.5206560
C	2.9056190	3.5564370	-1.2273050
C	3.9701510	2.9013600	-0.5998390
C	3.7958770	1.5523940	-0.2828860
P	-0.0555990	0.6302250	-1.4288110
C	3.0573270	-1.7465360	-1.7177230
C	2.8430200	-3.2577200	-1.6115500
C	2.4718350	-1.1984360	-3.0168510
C	3.6369280	-1.3337410	1.1523530
C	5.0624080	-1.6076480	0.6660640
C	3.1576300	-2.4717310	2.0548440
C	5.2504180	3.6241630	-0.2770890
C	-3.8176360	1.5670130	-0.1092070
C	-2.6328170	0.9116100	-0.4568140
C	-1.4951340	1.6387240	-0.8817400
C	-3.9179210	2.9596870	-0.1199800
C	-2.7841250	3.6781360	-0.5138430
C	-1.6133000	3.0369600	-0.9033170
C	-5.1825190	3.6663210	0.2906530
C	-3.7889170	-1.5265770	0.7472960
C	-2.8531820	-1.4378490	-2.0991510
C	-2.2531070	-2.7964080	-2.4609630
C	-4.3375380	-1.3261190	-2.4372100
C	-3.8863690	-3.0527700	0.7073940
C	-3.5794980	-1.0299110	2.1771810
V	-0.0430850	-1.3348650	0.2735740

C	0.0246050	-0.6723020	2.0291990
C	0.1173880	0.5900540	2.8824250
C	-0.6853720	0.3961950	4.1815910
C	-0.3939990	1.8432260	2.1691130
C	1.5957350	0.8163410	3.2548530
H	0.9323840	3.4349950	-2.0342010
H	3.0193980	4.6004120	-1.5334070
H	4.6310660	1.0225210	0.1790160
H	4.1385580	-1.5362520	-1.7258570
H	3.2817160	-3.6880290	-0.6993840
H	3.3043250	-3.7676520	-2.4722280
H	1.7703040	-3.5081180	-1.6105410
H	1.3867380	-1.3666070	-3.0858950
H	2.9458150	-1.7068720	-3.8719110
H	2.6487310	-0.1192030	-3.1307610
H	3.6455620	-0.4167240	1.7614500
H	5.7376340	-1.6672700	1.5343620
H	5.4582040	-0.8320380	-0.0043180
H	5.1327700	-2.5703070	0.1371590
H	3.1455010	-3.4371230	1.5253850
H	2.1483460	-2.2864400	2.4436510
H	3.8414740	-2.5767720	2.9121790
H	5.1384180	4.2363250	0.6329250
H	5.5455150	4.3032480	-1.0910710
H	6.0794920	2.9231570	-0.1026040
H	-4.6875640	0.9864250	0.2089560
H	-2.8182470	4.7716950	-0.5246120
H	-0.7703230	3.6547430	-1.2068970
H	-5.5495070	4.3293110	-0.5087040
H	-5.0179140	4.2935400	1.1813560
H	-5.9847920	2.9532530	0.5289960
H	-4.7315180	-1.1105160	0.3541290

H	-2.3113890	-0.6798100	-2.6902270
H	-2.4239650	-3.0096840	-3.5282940
H	-2.6920310	-3.6228070	-1.8826250
H	-1.1657740	-2.8113060	-2.2919420
H	-4.9413640	-2.0836160	-1.9137190
H	-4.4899940	-1.4777300	-3.5176950
H	-4.7381000	-0.3320620	-2.1860610
H	-4.1811470	-3.4313370	-0.2811980
H	-4.6477440	-3.3926370	1.4271240
H	-2.9317490	-3.5242490	0.9838490
H	-4.4369980	-1.3185460	2.8056610
H	-3.4744710	0.0636650	2.2297880
H	-2.6700950	-1.4729420	2.6092880
H	-0.6105210	1.2861890	4.8290460
H	-0.3166400	-0.4695040	4.7557610
H	-1.7519780	0.2288380	3.9696090
H	-1.4560860	1.7463150	1.8994720
H	-0.2880190	2.7307850	2.8154450
H	0.1712230	2.0293470	1.2460730
H	1.6935240	1.6536180	3.9668920
H	2.1830880	1.0728380	2.3601600
H	2.0395540	-0.0767730	3.7241760
C	-0.1401890	-3.4014110	0.6822790
H	0.0797630	-1.5520590	2.7090470
H	0.8820920	-3.7633200	0.9057290
H	-0.5210810	-4.0227160	-0.1512060
H	-0.7438880	-3.6334330	1.5788950
P	-2.4319190	-0.9031700	-0.3568940
P	2.3848860	-0.9090590	-0.1828490

6’

Symbol	X	Y	Z
C	0.0304860	-3.3560620	-0.7217830
C	0.5796380	-2.0641930	-0.7615810
C	1.8758260	-1.8773390	-0.2212350
C	0.7363120	-4.4207130	-0.1747270
C	2.0345130	-4.2547880	0.3268830
C	2.5867570	-2.9724590	0.2846800
P	-0.1995250	-0.6155860	-1.5668810
C	3.9329690	-0.1265570	0.9617140
C	4.6324390	1.2345660	0.9242120
C	3.5121950	-0.4877750	2.3887470
C	3.2339440	0.0085630	-1.9353390
C	4.5173700	-0.7939850	-2.1292910
C	3.3597430	1.4579440	-2.4005110
C	2.7917950	-5.4212560	0.9049750
C	-4.2278260	-0.4840860	-0.6039630
C	-2.8356630	-0.3841520	-0.5171030
C	-2.0217270	-0.9251610	-1.5310720
C	-4.8497500	-1.1282940	-1.6785500
C	-4.0351490	-1.6863080	-2.6702830
C	-2.6489950	-1.5753340	-2.6027070
C	-6.3501290	-1.1990100	-1.7802300
C	-3.1678320	1.3316590	1.8480480
C	-1.7719810	-1.3334920	1.8929280
C	-0.6073470	-1.3750010	2.8788640
C	-3.0811880	-1.8047650	2.5222230
C	-2.6607680	1.7133060	3.2392220
C	-3.4842270	2.5724700	1.0125100
V	0.3379620	1.1125010	0.2106310

C	0.3510280	2.7051560	-0.5320240
C	0.6054330	4.1820850	-0.6827950
C	1.9451630	4.5244170	-0.0117410
C	-0.5207970	4.9767490	0.0009080
C	0.6651370	4.5662550	-2.1698070
C	0.8529740	1.7502820	2.1543850
H	-0.9710690	-3.5259420	-1.1266250
H	0.2735370	-5.4118250	-0.1465990
H	3.5957940	-2.8308860	0.6807690
H	4.6440980	-0.8891180	0.6027260
H	5.1204190	1.4255420	-0.0418620
H	5.4132240	1.2766030	1.6999810
H	3.9239630	2.0560010	1.1145630
H	2.9013140	0.3067930	2.8357600
H	4.4095140	-0.6141110	3.0154470
H	2.9379770	-1.4237620	2.4413410
H	2.4423530	-0.4567160	-2.5469200
H	4.7880140	-0.8136300	-3.1970310
H	4.4045690	-1.8387310	-1.8006800
H	5.3675520	-0.3503870	-1.5872870
H	4.1492250	2.0054650	-1.8634990
H	2.4122670	1.9943680	-2.2520700
H	3.6086240	1.4870470	-3.4733330
H	2.8987380	-6.2343390	0.1696110
H	2.2697690	-5.8422500	1.7792580
H	3.8007490	-5.1276460	1.2285070
H	-4.8593170	-0.0560110	0.1791250
H	-4.4955140	-2.1980800	-3.5202270
H	-2.0338590	-1.9852190	-3.4083840
H	-6.8322610	-0.9841700	-0.8158270
H	-6.6839910	-2.1928300	-2.1140740
H	-6.7295590	-0.4662360	-2.5110610

H	-4.0857040	0.7329720	1.9703220
H	-1.5217110	-2.0321690	1.0794870
H	-0.5412020	-2.3807410	3.3240500
H	-0.7207590	-0.6522320	3.7010250
H	0.3525760	-1.1749580	2.3817850
H	-3.3788410	-1.1802390	3.3787890
H	-2.9632690	-2.8345770	2.8955450
H	-3.9082490	-1.8147970	1.7968330
H	-2.4619250	0.8355330	3.8707640
H	-3.4164140	2.3262950	3.7557570
H	-1.7338330	2.3011810	3.1817010
H	-4.2304760	3.1995120	1.5258900
H	-3.8846880	2.3169380	0.0198760
H	-2.5767530	3.1755720	0.8597960
H	2.1592610	5.6035590	-0.0890060
H	2.7715590	3.9753700	-0.4888230
H	1.9294140	4.2539300	1.0543700
H	-0.5969800	4.7087190	1.0656640
H	-0.3325170	6.0616770	-0.0663390
H	-1.4925270	4.7727950	-0.4750220
H	0.8549150	5.6459030	-2.2888240
H	-0.2832440	4.3327990	-2.6796670
H	1.4702350	4.0243360	-2.6897450
H	-0.1274280	2.1501100	-1.3969400
H	0.1949670	2.5952440	2.4260090
H	0.7615930	1.0059460	2.9639580
H	1.8867180	2.1382750	2.1843120
P	-1.9338740	0.2837390	0.9390020
P	2.4976640	-0.1415360	-0.2215230

6’-7’-TS

Symbol	X	Y	Z
C	-0.3994420	-3.4114500	-0.6863580
C	0.2810080	-2.1851920	-0.7332830
C	1.5767400	-2.1228740	-0.1707870
C	0.1880520	-4.5366500	-0.1177810
C	1.4898440	-4.4977030	0.3980210
C	2.1686570	-3.2765920	0.3534150
P	-0.3257710	-0.6729830	-1.5731110
C	3.7461560	-0.5528620	1.0509630
C	4.5658660	0.7391060	1.0346020
C	3.2675510	-0.8887540	2.4654750
C	3.2067670	-0.4957880	-1.8786320
C	4.4271420	-1.4096660	-1.9498040
C	3.4798780	0.8977900	-2.4385390
C	2.1235310	-5.7293440	0.9900370
C	-4.2940460	-0.0742430	-0.5690870
C	-2.8957540	-0.1172580	-0.5089880
C	-2.1672810	-0.7865390	-1.5085440
C	-5.0024790	-0.7031330	-1.5966410
C	-4.2712520	-1.3880100	-2.5753580
C	-2.8811370	-1.4176800	-2.5378770
C	-6.5062410	-0.6538340	-1.6549780
C	-2.9690070	1.8610600	1.6379150
C	-1.9838670	-0.9661080	2.0157840
C	-0.8741170	-1.0640080	3.0591900
C	-3.3640280	-1.1761840	2.6362840
C	-2.4210570	2.3675690	2.9720710
C	-3.1286620	3.0109480	0.6413360
V	0.4508340	1.0828230	0.0847670

C	0.8885930	2.7356490	-0.5821040
C	1.4478270	4.1416590	-0.6717450
C	0.4167620	5.1587730	-0.1501910
C	1.7760460	4.4612480	-2.1376960
C	2.7276320	4.2306580	0.1700110
C	0.9259850	1.4526470	1.8968880
H	-1.4077680	-3.4843030	-1.1031640
H	-0.3735130	-5.4752690	-0.0852920
H	3.1814390	-3.2316110	0.7629210
H	4.3970400	-1.3747120	0.7097630
H	5.0564050	0.9097090	0.0656240
H	5.3561610	0.6946300	1.8008090
H	3.9355760	1.6145940	1.2513760
H	2.6798420	-0.0678480	2.8958590
H	4.1414410	-1.0631230	3.1140350
H	2.6430710	-1.7922660	2.4984380
H	2.4117370	-0.9401180	-2.5016810
H	4.7453990	-1.5288780	-2.9978350
H	4.2144790	-2.4160650	-1.5574420
H	5.2852170	-0.9956920	-1.3964690
H	4.2521070	1.4366300	-1.8675760
H	2.5648630	1.5054930	-2.4242310
H	3.8308160	0.8221810	-3.4804750
H	2.1657020	-6.5497790	0.2562420
H	1.5513840	-6.0987090	1.8561280
H	3.1506120	-5.5314530	1.3291070
H	-4.8610870	0.4594240	0.1983610
H	-4.8008310	-1.8869160	-3.3921530
H	-2.3289330	-1.9231970	-3.3348000
H	-6.9401480	-1.6616540	-1.5568300
H	-6.8542120	-0.2442160	-2.6159990
H	-6.9228450	-0.0295030	-0.8518920

H	-3.9573600	1.4104100	1.8248110
H	-1.8080660	-1.7750750	1.2871170
H	-0.9422050	-2.0392540	3.5676490
H	-0.9536410	-0.2830240	3.8315260
H	0.1190410	-0.9807570	2.5996610
H	-3.5900700	-0.4136360	3.3983970
H	-3.3990260	-2.1563320	3.1379730
H	-4.1681650	-1.1663380	1.8862120
H	-2.2778450	1.5600660	3.7051470
H	-3.1237690	3.0932080	3.4117380
H	-1.4564140	2.8793400	2.8372730
H	-3.7832420	3.7913230	1.0612870
H	-3.5678070	2.6815990	-0.3121870
H	-2.1540750	3.4744040	0.4197600
H	0.8090690	6.1880610	-0.2045560
H	0.1562490	4.9542700	0.9010420
H	-0.5113900	5.1208000	-0.7426640
H	0.8789140	4.3841390	-2.7727190
H	2.1704470	5.4860140	-2.2358140
H	2.5314710	3.7683190	-2.5369120
H	3.1568120	5.2449540	0.1293380
H	3.4842250	3.5209960	-0.1978390
H	2.5276500	3.9930520	1.2269690
H	0.6498790	2.5946030	0.7825150
H	1.9331790	1.8021940	2.1860760
H	0.2575250	1.5450130	2.7669070
H	0.0757540	2.5217170	-1.3373200
P	-1.8932410	0.5391390	0.8879220
P	2.3673930	-0.4576060	-0.1991750

6’-7”-TS

Symbol	X	Y	Z
C	-1.0394450	-3.4315490	0.7585610
C	-1.1875260	-2.0359010	0.7150820
C	-2.3767090	-1.5201840	0.1557770
C	-2.0307440	-4.2786770	0.2790750
C	-3.2305610	-3.7768860	-0.2414510
C	-3.3816010	-2.3897950	-0.2864830
P	0.0016870	-0.8324320	1.4171620
C	-3.7561680	0.6127740	-1.2931030
C	-4.1047030	2.1000510	-1.3968480
C	-3.3124010	0.0284940	-2.6377020
C	-3.3658410	0.7143040	1.6664310
C	-4.8062300	0.2153150	1.7477200
C	-3.2455610	2.1841690	2.0667640
C	-4.3031260	-4.7050900	-0.7475070
C	3.8899100	-1.7064100	0.3447210
C	2.6001410	-1.1645090	0.3652560
C	1.6455150	-1.6504250	1.2790640
C	4.2649020	-2.7472490	1.2008720
C	3.3099580	-3.2265260	2.1033540
C	2.0318880	-2.6760510	2.1531260
C	5.6529600	-3.3280480	1.1361610
C	3.5623600	1.1878990	-1.0627820
C	1.8030000	-0.7712000	-2.4036260
C	3.0547150	-1.4375950	-2.9677610
C	0.6503850	-1.7664010	-2.2747830
C	3.3411840	2.1902120	-2.1945230
C	3.9557850	1.8760000	0.2429580
V	-0.0800710	1.1816200	-0.0871960

C	0.3102990	2.8185180	0.6629090
C	0.5582240	4.3046040	0.8511950
C	-0.7863240	5.0396550	1.0079570
C	1.3031950	4.8753840	-0.3616230
C	1.3975440	4.5185340	2.1197940
C	-0.3623870	1.7891200	-1.8742490
H	-0.1252280	-3.8661000	1.1683090
H	-1.8721800	-5.3606350	0.3160660
H	-4.3065330	-1.9853090	-0.7058740
H	-4.6685400	0.0795150	-0.9789580
H	-4.6460820	2.4574830	-0.5094220
H	-4.7565780	2.2736490	-2.2676140
H	-3.2086770	2.7275580	-1.5192140
H	-2.4109910	0.5234580	-3.0224490
H	-4.1176860	0.1585620	-3.3786780
H	-3.0921020	-1.0461880	-2.5720820
H	-2.7570570	0.1304990	2.3789800
H	-5.1861180	0.3329400	2.7751510
H	-4.8921560	-0.8500300	1.4875270
H	-5.4754420	0.7899680	1.0874660
H	-3.8290330	2.8482140	1.4114670
H	-2.2022870	2.5238260	2.0391600
H	-3.6184340	2.3245350	3.0938690
H	-3.9403710	-5.3138840	-1.5911820
H	-5.1875350	-4.1504390	-1.0928350
H	-4.6317750	-5.4038850	0.0380280
H	4.6350340	-1.3167850	-0.3542690
H	3.5782490	-4.0284330	2.7968050
H	1.3227170	-3.0352920	2.9035280
H	5.7878560	-3.9298400	0.2227260
H	5.8590530	-3.9804330	1.9965020
H	6.4182950	-2.5372050	1.1197290

H	4.3766450	0.5075740	-1.3607320
H	1.4753880	0.0307260	-3.0864350
H	2.8261700	-1.8823800	-3.9497180
H	3.4057310	-2.2527450	-2.3159300
H	3.8863920	-0.7327340	-3.1172400
H	0.8821490	-2.5665520	-1.5537840
H	0.4532960	-2.2408800	-3.2494750
H	-0.2727830	-1.2689320	-1.9461960
H	3.1430580	1.6967550	-3.1580170
H	4.2372180	2.8180440	-2.3234110
H	2.4947920	2.8553790	-1.9724340
H	4.8316530	2.5254250	0.0850580
H	4.2109890	1.1495250	1.0291890
H	3.1258370	2.4936160	0.6173900
H	-0.6324960	6.1244010	1.1335150
H	-1.3390330	4.6754340	1.8879570
H	-1.4256110	4.8878170	0.1234150
H	0.7589930	4.6718850	-1.2982530
H	1.4181380	5.9673570	-0.2707170
H	2.3076430	4.4365760	-0.4510520
H	1.5925290	5.5909420	2.2844190
H	2.3676650	4.0041100	2.0487500
H	0.8776580	4.1303840	3.0102450
H	-0.3022630	2.7733460	-0.5557440
H	-1.3233970	2.0675280	-2.3360800
H	0.4534500	2.1657430	-2.5183730
H	-0.0806260	2.3253100	1.5976910
P	-2.4999220	0.3125350	0.0509290
P	2.0422500	0.1497710	-0.7932450

7’

Symbol	X	Y	Z
C	0.5831630	-3.0814270	0.0749440
C	-0.2359780	-2.0054190	0.4595150
C	-1.5630390	-1.9745580	-0.0407700
C	0.1057450	-4.0903320	-0.7510950
C	-1.2151090	-4.0842570	-1.2224990
C	-2.0321870	-3.0150620	-0.8509190
P	0.2862490	-0.6516490	1.5948330
C	-4.1079310	-0.5259990	-0.5807940
C	-5.0838520	0.5477520	-0.0892900
C	-3.8931260	-0.3851870	-2.0864150
C	-3.0516850	-0.9532120	2.1575260
C	-4.1513450	-2.0117130	2.2104770
C	-3.3215070	0.2344580	3.0784980
C	-1.7279620	-5.2022940	-2.0911400
C	4.3013050	-0.0351240	0.7376090
C	2.9042020	-0.0463950	0.6268640
C	2.1325010	-0.7428080	1.5690470
C	4.9625630	-0.7103980	1.7642540
C	4.1848520	-1.4192130	2.6905660
C	2.7982830	-1.4273990	2.5983470
C	6.4627800	-0.6806540	1.8848260
C	3.0282340	2.2004220	-1.2435590
C	2.2289430	-0.5974210	-2.1074590
C	2.0245980	-0.0718190	-3.5284900
C	3.5256950	-1.4005350	-2.0275010
C	2.4001420	3.0218130	-2.3749670
C	3.2516120	3.0906220	-0.0156720
V	-0.4583240	0.8666090	-0.2458160

C	-0.5191130	2.8804480	0.2270480
C	-1.7666000	3.7816940	0.2955640
C	-2.5156830	3.7388030	-1.0430790
C	-1.3425410	5.2327410	0.5794370
C	-2.7062590	3.3167290	1.4101240
C	-0.8486250	0.6209200	-2.0220200
H	1.6151800	-3.1241820	0.4326470
H	0.7717100	-4.9114100	-1.0329340
H	-3.0619150	-2.9959040	-1.2169750
H	-4.5557470	-1.5141590	-0.3836350
H	-5.3333210	0.4460450	0.9757190
H	-6.0264860	0.4789480	-0.6551330
H	-4.6780110	1.5589240	-0.2463230
H	-3.5174850	0.6157160	-2.3428790
H	-4.8541450	-0.5304520	-2.6063490
H	-3.1778420	-1.1150940	-2.4879530
H	-2.1257640	-1.4212260	2.5285340
H	-4.2505080	-2.3930380	3.2395500
H	-3.9230980	-2.8738180	1.5645510
H	-5.1350970	-1.6124550	1.9178240
H	-4.2049440	0.8201690	2.7818360
H	-2.4571730	0.9127670	3.1097600
H	-3.4967700	-0.1271280	4.1047650
H	-1.0730220	-5.3673260	-2.9609760
H	-2.7392660	-4.9907220	-2.4669020
H	-1.7711680	-6.1520510	-1.5337250
H	4.9015120	0.5075320	0.0021830
H	4.6767670	-1.9591690	3.5048000
H	2.2088000	-1.9640350	3.3467690
H	6.7753750	-0.1699590	2.8095460
H	6.9275380	-0.1549410	1.0387020
H	6.8796540	-1.6991170	1.9208810

H	4.0040210	1.8178710	-1.5916210
H	1.3939040	-1.2793400	-1.8771170
H	1.8711480	-0.9207390	-4.2134340
H	2.9126960	0.4744060	-3.8835690
H	1.1483820	0.5850670	-3.6144830
H	4.4160700	-0.7623060	-2.1496740
H	3.5420710	-2.1414640	-2.8432080
H	3.6280070	-1.9483230	-1.0816300
H	2.3648000	2.4843000	-3.3289290
H	2.9927400	3.9365940	-2.5352330
H	1.3738500	3.3299470	-2.1276370
H	3.8820230	3.9511450	-0.2906960
H	3.7479510	2.5651440	0.8113040
H	2.2972700	3.4861710	0.3637740
H	-3.3882130	4.4137900	-1.0415720
H	-2.8729480	2.7196350	-1.2578680
H	-1.8601300	4.0430000	-1.8762190
H	-0.6843430	5.6162870	-0.2175750
H	-2.2135680	5.9073540	0.6477430
H	-0.7879330	5.3024620	1.5294030
H	-3.6133030	3.9421240	1.4661010
H	-2.2091990	3.3558850	2.3930800
H	-3.0208640	2.2788070	1.2391300
H	0.2157360	3.3575260	-0.4467070
H	-1.1582550	1.5794750	-2.4808070
H	-0.8594530	-0.2150970	-2.7357020
H	-0.0263540	2.8403610	1.2249570
P	1.9897530	0.7173150	-0.7751040
P	-2.5294600	-0.4754700	0.4131910

7”

Symbol	X	Y	Z
C	-0.9670250	-3.0925920	0.9032460
C	-1.1779660	-1.7065410	0.8218300
C	-2.4023750	-1.2562690	0.2781590
C	-1.9342200	-3.9940650	0.4754720
C	-3.1679730	-3.5582250	-0.0263510
C	-3.3809630	-2.1806060	-0.1072770
P	-0.0264890	-0.4261180	1.4586180
C	-3.8735720	0.8044950	-1.2202900
C	-4.1214370	2.2949660	-1.4620870
C	-3.4622560	0.1208830	-2.5258580
C	-3.4050540	1.0130370	1.7415120
C	-4.7150320	0.2770830	2.0116220
C	-3.5539150	2.5184120	1.9745020
C	-4.2115220	-4.5451960	-0.4787700
C	3.7943380	-1.5823410	0.3672170
C	2.5274550	-0.9870970	0.3651840
C	1.6279900	-1.2436720	1.4114670
C	4.2056700	-2.4234500	1.4041100
C	3.3059460	-2.6653240	2.4504050
C	2.0441300	-2.0804570	2.4569230
C	5.5782010	-3.0422850	1.4123560
C	3.3474970	0.6375150	-1.9458900
C	1.0497580	-1.3524960	-2.0555640
C	0.9108540	-1.0349940	-3.5433560
C	1.6884070	-2.7297560	-1.8756690
C	2.9728340	1.5445340	-3.1217980
C	4.3077630	1.3957560	-1.0198990
V	-0.2082180	1.2824480	-0.3623390

C	-0.0917270	2.9872210	0.8336920
C	1.2368830	3.6944380	1.1493540
C	0.9937530	4.8569820	2.1259220
C	1.8425450	4.2539560	-0.1434060
C	2.2108570	2.7045970	1.8018220
C	-0.4400310	1.7638550	-2.0838770
H	-0.0259310	-3.4727410	1.3080980
H	-1.7303570	-5.0671530	0.5410400
H	-4.3365380	-1.8251000	-0.5021680
H	-4.8072410	0.3447250	-0.8543000
H	-4.5571720	2.8013920	-0.5909830
H	-4.8202550	2.4278700	-2.3031650
H	-3.1808370	2.8069750	-1.7211130
H	-2.5440830	0.5754430	-2.9238340
H	-4.2586020	0.2473160	-3.2767440
H	-3.2794480	-0.9562150	-2.4098240
H	-2.6435540	0.6395620	2.4490010
H	-5.0646320	0.5020320	3.0321320
H	-4.6050490	-0.8140900	1.9347820
H	-5.5116690	0.5957320	1.3198660
H	-4.4304600	2.9272460	1.4500420
H	-2.6690110	3.0849250	1.6560390
H	-3.7040320	2.7123070	3.0483040
H	-3.8453790	-5.1568600	-1.3189420
H	-5.1289940	-4.0383500	-0.8106550
H	-4.4859000	-5.2393310	0.3311910
H	4.4847140	-1.4072420	-0.4621290
H	3.6048630	-3.3144930	3.2783770
H	1.3647730	-2.2676680	3.2930490
H	5.5305780	-4.1204790	1.6287580
H	6.2125080	-2.5838060	2.1881590
H	6.0875030	-2.9119270	0.4467920

H	3.8597760	-0.2587630	-2.3385740
H	0.0321220	-1.4008200	-1.6308070
H	0.2546510	-1.7865750	-4.0100530
H	1.8816220	-1.0881920	-4.0609800
H	0.4661600	-0.0501260	-3.7268310
H	2.7471090	-2.7340210	-2.1828210
H	1.1576320	-3.4593380	-2.5081270
H	1.6344780	-3.0894040	-0.8405120
H	2.4030480	1.0307800	-3.9035980
H	3.8914360	1.9377540	-3.5859640
H	2.3725900	2.4033590	-2.7850530
H	5.2394300	1.6193800	-1.5633910
H	4.5763690	0.8422680	-0.1108230
H	3.8732630	2.3554370	-0.7068160
H	1.9310410	5.3853900	2.3725660
H	0.5556320	4.4929980	3.0694560
H	0.2953680	5.5947180	1.6977020
H	1.1663590	4.9910710	-0.6078460
H	2.8089270	4.7544910	0.0371760
H	2.0020440	3.4506850	-0.8778630
H	3.1780830	3.1798970	2.0370680
H	2.4119250	1.8457490	1.1444390
H	1.7946980	2.3018680	2.7396680
H	-0.8204080	3.7264550	0.4473110
H	-0.3572590	2.8215730	-1.7164880
H	-0.5543320	1.7295370	-3.1737530
H	-0.5220600	2.5936900	1.7755110
P	-2.5924220	0.5691840	0.1141570
P	1.8465540	0.0215730	-1.0152270

V^V original ligand (L_0)

1

Symbol	X	Y	Z
C	-0.7072230	-3.4461090	-0.7264660
C	-0.9497850	-2.1385240	-0.2762970
C	-2.2777620	-1.7022080	-0.1584240
C	-1.7678710	-4.2919580	-1.0227880
C	-3.1011550	-3.8784350	-0.8927400
C	-3.3307750	-2.5695180	-0.4650260
N	0.0746550	-1.2247880	0.0330750
P	-2.4326830	0.0600450	0.2866340
C	-3.8398870	0.5909540	-0.8228780
C	-4.3498340	2.0112370	-0.5871210
C	-3.5330450	0.3432870	-2.3029230
C	-3.0641510	0.0701340	2.0378910
C	-4.4593040	-0.5246100	2.2141370
C	-2.9388310	1.4441750	2.6959630
C	-4.2336200	-4.8109240	-1.2265060
C	3.7560900	-1.6275360	0.3369780
C	2.4990910	-1.1801540	-0.0712790
C	1.3265410	-1.7451550	0.4599160
C	3.8838300	-2.6450440	1.2859530
C	2.7073280	-3.1957320	1.8162210
C	1.4494440	-2.7622000	1.4154930
C	5.2320740	-3.1322010	1.7415150
P	2.1318980	0.1746520	-1.2247710

C	3.6721990	1.1587930	-1.4901810
C	1.7250210	-0.7479230	-2.7960290
C	0.7971890	0.0276840	-3.7317130
C	2.9614390	-1.3103480	-3.4940360
C	3.5031290	2.1510150	-2.6426650
C	4.0976750	1.8644840	-0.2043050
V	0.0194220	0.7129030	-0.0090000
C	0.5725960	1.2045370	1.5492830
C	1.0495350	1.1330230	2.9747580
C	0.7604690	2.4632600	3.6885170
C	2.5636780	0.8627410	2.9899780
C	0.3257900	-0.0176360	3.6908780
C	-0.6764500	2.3388040	-1.1009760
C	-0.6177290	3.8388200	-0.7172020
C	0.8295100	4.3216000	-0.5584800
C	-1.2627230	4.6340100	-1.8670380
C	-1.3897420	4.1382720	0.5755090
H	0.3172620	-3.8010290	-0.8479180
H	-1.5544540	-5.3045550	-1.3757820
H	-4.3639600	-2.2211770	-0.3908120
H	-4.6514010	-0.0959520	-0.5326160
H	-4.6327810	2.1938470	0.4580590
H	-5.2483710	2.1768520	-1.2012200
H	-3.6127030	2.7707680	-0.8813880
H	-2.7848660	1.0483040	-2.6945350
H	-4.4509100	0.4910030	-2.8921810
H	-3.1761940	-0.6781480	-2.4972340
H	-2.3341800	-0.6033540	2.5185050
H	-4.7025530	-0.5746430	3.2868340
H	-4.5325970	-1.5462580	1.8158380
H	-5.2329040	0.0957960	1.7353540
H	-3.7095870	2.1446270	2.3433110

H	-1.9576980	1.8985450	2.5049230
H	-3.0631060	1.3487490	3.7857450
H	-4.2028540	-5.7157790	-0.6000130
H	-4.1797960	-5.1419090	-2.2754000
H	-5.2107040	-4.3310510	-1.0757700
H	4.6594850	-1.1765710	-0.0819190
H	2.7807180	-3.9838030	2.5703640
H	0.5534690	-3.2091770	1.8503070
H	6.0477530	-2.6155410	1.2169860
H	5.3459590	-4.2123590	1.5622860
H	5.3673490	-2.9658840	2.8215900
H	4.4473290	0.4251980	-1.7701980
H	1.1467740	-1.5977640	-2.3976380
H	0.5505710	-0.5944530	-4.6057300
H	1.2477680	0.9583150	-4.1072160
H	-0.1520190	0.2852280	-3.2380530
H	3.5774840	-0.5224310	-3.9529000
H	2.6523180	-1.9931600	-4.3004220
H	3.5944270	-1.8858920	-2.8014100
H	3.3142870	1.6555630	-3.6045370
H	4.4243290	2.7425460	-2.7539000
H	2.6803000	2.8555690	-2.4509450
H	5.0367870	2.4120090	-0.3764520
H	4.2669150	1.1609640	0.6215580
H	3.3391380	2.5953440	0.1157820
H	1.1028980	2.4143610	4.7336490
H	-0.3157160	2.6922340	3.7008060
H	1.2856550	3.3008090	3.2032620
H	3.1184560	1.6796000	2.5074820
H	2.9144670	0.7881690	4.0311320
H	2.8076480	-0.0793970	2.4782420
H	0.7290190	-0.1392050	4.7080620

H	0.4631600	-0.9663380	3.1532620
H	-0.7506760	0.1822030	3.7793430
H	0.3601010	2.2251210	1.1073660
H	0.0493690	2.1583710	-1.9235830
H	-1.6601950	2.1208850	-1.5320900
H	0.8568340	5.3862870	-0.2795560
H	1.3893460	4.2149360	-1.5003780
H	1.3771680	3.7683050	0.2214270
H	-1.1996730	5.7187700	-1.6850840
H	-2.3277040	4.3771910	-1.9827030
H	-0.7616290	4.4238320	-2.8253070
H	-0.8874970	3.7265110	1.4642010
H	-2.4081580	3.7264300	0.5478560
H	-1.4736200	5.2251950	0.7313950

1-2-TS

Symbol	X	Y	Z
C	-1.1050920	-3.4864870	-0.4344870
C	-1.1995190	-2.1239590	-0.1020180
C	-2.4763760	-1.5324390	-0.0788380
C	-2.2483090	-4.2242640	-0.7138740
C	-3.5269960	-3.6505470	-0.6838030
C	-3.6129990	-2.2933400	-0.3678490
N	-0.0880060	-1.3139840	0.1717970
P	-2.4606210	0.2560420	0.2711150
C	-3.7636100	0.9125820	-0.8841070
C	-4.1748340	2.3573620	-0.6028810
C	-3.3244080	0.7067900	-2.3365200
C	-3.0596220	0.4413930	2.0171880

C	-4.4930250	-0.0316700	2.2423850
C	-2.8151900	1.8544640	2.5500060
C	-4.7523080	-4.4717340	-0.9823000
C	3.5342210	-2.0368610	0.4689860
C	2.3145200	-1.5140000	0.0380190
C	1.1041450	-1.9228440	0.6292170
C	3.5865510	-2.9792810	1.5001430
C	2.3744680	-3.3802530	2.0821520
C	1.1522760	-2.8693360	1.6603650
C	4.8946660	-3.5407620	1.9870990
P	2.0413390	-0.2417690	-1.2348080
C	3.6557280	0.5471450	-1.6633310
C	1.4653670	-1.2399240	-2.7026450
C	0.5615320	-0.4314890	-3.6355290
C	2.5931970	-1.9726030	-3.4243750
C	3.5063950	1.4651340	-2.8794970
C	4.2391630	1.3010870	-0.4710680
V	0.0384390	0.6344560	-0.0721870
C	0.7188540	1.2201420	1.3286310
C	1.2469640	1.4562380	2.7114340
C	0.9561210	2.8753690	3.2203720
C	2.7635370	1.2059490	2.7252030
C	0.5543130	0.4243340	3.6231600
C	-0.3258900	2.5739740	-0.9719680
C	0.0507110	4.0608530	-0.7748120
C	1.5431210	4.2163230	-0.4639030
C	-0.2688190	4.8072750	-2.0786960
C	-0.7864830	4.6546450	0.3638000
H	-0.1278490	-3.9695480	-0.4804970
H	-2.1429350	-5.2810920	-0.9746640
H	-4.5995230	-1.8221480	-0.3577870
H	-4.6463710	0.2778350	-0.7036120

H	-4.7000150	2.4578880	0.3566280
H	-4.8664980	2.6994780	-1.3880260
H	-3.3197880	3.0492590	-0.5945820
H	-2.4804810	1.3602750	-2.6077250
H	-4.1551290	0.9552620	-3.0144300
H	-3.0348410	-0.3352680	-2.5391680
H	-2.3737290	-0.2442030	2.5449740
H	-4.7403990	0.0296180	3.3135300
H	-4.6370910	-1.0763950	1.9322270
H	-5.2189770	0.5962460	1.7027020
H	-3.5548040	2.5738270	2.1713590
H	-1.8177190	2.2221260	2.2719920
H	-2.8866620	1.8612090	3.6485520
H	-4.9093470	-5.2459260	-0.2146550
H	-4.6618250	-4.9896380	-1.9494290
H	-5.6572250	-3.8488330	-1.0172160
H	4.4649210	-1.7076500	-0.0010950
H	2.3909900	-4.1095520	2.8966620
H	0.2264290	-3.1951600	2.1387490
H	5.7377730	-3.1938940	1.3736370
H	4.8899200	-4.6411820	1.9646880
H	5.0901260	-3.2389740	3.0282280
H	4.3323530	-0.2827280	-1.9298560
H	0.8312660	-1.9970470	-2.2140680
H	0.1792740	-1.0793810	-4.4392910
H	1.0823520	0.4118310	-4.1129490
H	-0.3174800	-0.0313940	-3.1047970
H	3.2513740	-1.2879660	-3.9802250
H	2.1703800	-2.6814770	-4.1529710
H	3.2117620	-2.5537230	-2.7230940
H	3.2382720	0.9184570	-3.7933160
H	4.4620870	1.9755810	-3.0726710

H	2.7465060	2.2442290	-2.7109200
H	5.2175180	1.7245610	-0.7448580
H	4.3859780	0.6545460	0.4036490
H	3.5840790	2.1332350	-0.1742860
H	1.3677270	2.9900660	4.2348750
H	-0.1230180	3.0765320	3.2792140
H	1.4213860	3.6429730	2.5851680
H	3.2978310	1.9647240	2.1365030
H	3.1374260	1.2571540	3.7595320
H	3.0050720	0.2113200	2.3223140
H	0.9492570	0.5075630	4.6476690
H	0.7341530	-0.6006890	3.2672010
H	-0.5309020	0.5944080	3.6627690
H	0.1986160	2.2222300	0.4066910
H	0.2686100	2.1639090	-1.8192260
H	-1.3798940	2.5213920	-1.2693600
H	1.8012990	5.2748560	-0.3082580
H	2.1636120	3.8428890	-1.2930900
H	1.8369870	3.6682270	0.4446140
H	-0.0258820	5.8778330	-1.9895100
H	-1.3376610	4.7271020	-2.3330380
H	0.3103220	4.4017310	-2.9236640
H	-0.6117770	4.1268850	1.3129570
H	-1.8630600	4.5974370	0.1400450
H	-0.5378300	5.7148760	0.5243940

A

Symbol	X	Y	Z
C	0.8727490	-2.5735640	-1.4386030

C	1.0566200	-1.2981700	-0.8744010
C	2.3651530	-0.9283300	-0.4802110
C	1.9459530	-3.4373030	-1.6063400
C	3.2464520	-3.0882300	-1.2149990
C	3.4256660	-1.8269040	-0.6467040
N	-0.0036660	-0.4053450	-0.6494490
P	2.5558900	0.7222920	0.2873590
C	3.8042190	0.4918320	1.6378810
C	4.2022500	1.8246050	2.2738950
C	3.2377570	-0.4777270	2.6771740
C	3.2269110	1.8333270	-1.0354470
C	4.6245940	1.4452660	-1.5108420
C	3.1228600	3.3118220	-0.6544810
C	4.3931050	-4.0445700	-1.3999950
C	-3.6601890	-0.7874630	-1.0136830
C	-2.3869010	-0.6509070	-0.4624730
C	-1.2505460	-0.5880300	-1.2921140
C	-3.8363630	-0.8665750	-2.3997250
C	-2.6948970	-0.8088580	-3.2122690
C	-1.4175530	-0.6741550	-2.6762650
C	-5.2068180	-0.9861460	-3.0098450
P	-1.9584150	-0.5040920	1.3075930
C	-3.4744230	-0.0380460	2.2559880
C	-1.4990270	-2.2566020	1.7548810
C	-0.5152060	-2.3167690	2.9239680
C	-2.6987640	-3.1840360	1.9284880
C	-3.1884530	-0.0635930	3.7594010
C	-4.0021040	1.3259600	1.8182640
V	0.1053360	0.7948510	0.8358050
C	-0.6237950	2.1079200	0.1670320
C	-1.1380780	3.1507980	-0.7765490
C	-0.9135660	4.5365370	-0.1475210

C	-2.6332030	2.9503960	-1.0681760
C	-0.3496570	3.0294820	-2.0940210
H	-0.1262400	-2.8923300	-1.7398390
H	1.7668670	-4.4217520	-2.0474210
H	4.4304150	-1.5378710	-0.3269660
H	4.7008730	0.0408190	1.1827770
H	4.7812280	2.4572990	1.5881210
H	4.8339550	1.6372560	3.1554780
H	3.3243780	2.3993900	2.6114500
H	2.3558090	-0.0483660	3.1832370
H	3.9880800	-0.6751280	3.4577500
H	2.9549210	-1.4461930	2.2389250
H	2.5071990	1.6419830	-1.8504010
H	4.9226660	2.0908230	-2.3513130
H	4.6678360	0.4054480	-1.8653490
H	5.3760160	1.5758640	-0.7162270
H	3.9090580	3.6131230	0.0518700
H	2.1491710	3.5511910	-0.2022340
H	3.2397480	3.9345880	-1.5543530
H	4.2136270	-4.9892460	-0.8633940
H	5.3368580	-3.6214760	-1.0284130
H	4.5342860	-4.2973830	-2.4622900
H	-4.5367600	-0.8296260	-0.3613000
H	-2.8116860	-0.8639160	-4.2977300
H	-0.5456640	-0.6258910	-3.3319670
H	-5.9694120	-1.2148500	-2.2524860
H	-5.2374350	-1.7775980	-3.7733450
H	-5.4988850	-0.0461880	-3.5048060
H	-4.2318160	-0.8064080	2.0255680
H	-0.9508270	-2.5753430	0.8525090
H	-0.1603630	-3.3493670	3.0643460
H	-0.9633080	-1.9909620	3.8743510

H	0.3761270	-1.6950400	2.7395370
H	-3.2784620	-2.9479750	2.8340780
H	-2.3553090	-4.2258330	2.0226300
H	-3.3774390	-3.1391790	1.0630480
H	-2.9224170	-1.0653820	4.1232590
H	-4.0844180	0.2570710	4.3119920
H	-2.3727740	0.6282680	4.0257340
H	-4.9051730	1.5764590	2.3956000
H	-4.2692070	1.3460290	0.7535340
H	-3.2553260	2.1151230	1.9980530
H	-1.2698010	5.3241330	-0.8303240
H	0.1529620	4.7196280	0.0538840
H	-1.4627180	4.6360530	0.8012810
H	-3.2415090	3.1080580	-0.1680240
H	-2.9610240	3.6810970	-1.8241940
H	-2.8354670	1.9422610	-1.4587940
H	-0.7112730	3.7758230	-2.8188630
H	-0.4781740	2.0315140	-2.5382540
H	0.7231470	3.2050270	-1.9362340

5-6-TS

Symbol	X	Y	Z
C	1.0861050	-2.9871410	-0.8266960
C	1.1756940	-1.6106640	-0.5617170
C	2.4506330	-1.0576970	-0.3241700
C	2.2319070	-3.7714250	-0.8731970
C	3.5073550	-3.2338720	-0.6533640
C	3.5881220	-1.8686460	-0.3695020
N	0.0622840	-0.7628420	-0.4873920

P	2.4458660	0.7205060	0.0901320
C	3.7279450	0.8515950	1.4304960
C	4.1814110	2.2798650	1.7301320
C	3.2199880	0.1352680	2.6836240
C	3.0446840	1.6032030	-1.4285210
C	4.4646480	1.2330100	-1.8480890
C	2.8377240	3.1164360	-1.3243800
C	4.7366450	-4.0982370	-0.7336750
C	-3.5814410	-1.2799290	-0.9099270
C	-2.3368530	-0.9902830	-0.3497340
C	-1.1547330	-1.1357100	-1.1040520
C	-3.6876770	-1.7243620	-2.2308690
C	-2.5043030	-1.8654110	-2.9721570
C	-1.2571790	-1.5815470	-2.4273570
C	-5.0221320	-2.0460440	-2.8473340
P	-2.0064220	-0.3331940	1.3182350
C	-3.5684630	0.2969390	2.0721950
C	-1.4366330	-1.8327800	2.2667250
C	-0.4525770	-1.4666580	3.3784860
C	-2.5768260	-2.7342400	2.7322820
C	-3.3192950	0.7302260	3.5198200
C	-4.1550010	1.4456260	1.2560400
V	-0.0498350	0.9068340	0.5157750
C	-0.7799980	1.9997520	-0.5002620
C	-1.3117690	2.8687790	-1.5940960
C	-1.1108420	4.3469990	-1.2268670
C	-2.8026540	2.5787020	-1.8292560
C	-0.5280040	2.5148560	-2.8721020
H	0.1102560	-3.4475380	-0.9897110
H	2.1308080	-4.8405450	-1.0790330
H	4.5727280	-1.4330260	-0.1794360
H	4.6034640	0.3009650	1.0496210

H	4.7109130	2.7328930	0.8812970
H	4.8828670	2.2659320	2.5785460
H	3.3480960	2.9424240	2.0074830
H	2.3643860	0.6660010	3.1292230
H	4.0149660	0.1001960	3.4441150
H	2.9195130	-0.9027110	2.4749240
H	2.3396700	1.2181180	-2.1861030
H	4.7215900	1.7494160	-2.7860110
H	4.5742220	0.1540580	-2.0276460
H	5.2060800	1.5385530	-1.0933260
H	3.5913540	3.5945090	-0.6836320
H	1.8446990	3.3659680	-0.9226740
H	2.9238690	3.5742010	-2.3218450
H	5.6204940	-3.5830370	-0.3319600
H	4.9582250	-4.3738880	-1.7772920
H	4.6050900	-5.0350680	-0.1717320
H	-4.4895550	-1.1579950	-0.3132110
H	-2.5646930	-2.1992800	-4.0114710
H	-0.3545770	-1.6896390	-3.0322270
H	-5.1063450	-3.1209820	-3.0720530
H	-5.1617350	-1.5045160	-3.7953010
H	-5.8523400	-1.7778330	-2.1791450
H	-4.2766230	-0.5490290	2.0678320
H	-0.8685740	-2.3760110	1.4938460
H	-0.0601190	-2.3814670	3.8484730
H	-0.9135190	-0.8613990	4.1732040
H	0.4145370	-0.9113120	2.9865900
H	-3.1730760	-2.2737940	3.5345640
H	-2.1673510	-3.6759380	3.1292210
H	-3.2534700	-2.9945760	1.9039220
H	-2.9964830	-0.0980560	4.1650270
H	-4.2497560	1.1329510	3.9476540

H	-2.5595820	1.5263820	3.5733730
H	-5.1024620	1.7742070	1.7096830
H	-4.3604290	1.1626630	0.2154380
H	-3.4705670	2.3081200	1.2467700
H	-1.4793370	4.9920350	-2.0394340
H	-0.0479240	4.5825850	-1.0660410
H	-1.6650080	4.6114470	-0.3128080
H	-3.4146430	2.9083380	-0.9798080
H	-3.1471480	3.1238080	-2.7216300
H	-2.9786200	1.5049920	-1.9952670
H	-0.9103290	3.1033850	-3.7206230
H	-0.6410200	1.4477170	-3.1164930
H	0.5428720	2.7339510	-2.7625510
C	0.2756440	2.3884480	2.1177700
H	-0.3527330	2.5290980	0.8029970
H	1.3074470	2.7577920	2.1413360
H	0.1580220	1.6078470	2.8896570
H	-0.3823320	3.2197720	2.4155960

6

Symbol	X	Y	Z
C	1.0738040	-2.6792730	-1.4291000
C	1.1870950	-1.4133330	-0.8395960
C	2.4536410	-0.9599860	-0.4339210
C	2.2066550	-3.4670390	-1.6008760
C	3.4768200	-3.0375240	-1.1923250
C	3.5760840	-1.7719100	-0.6065100
N	0.0940630	-0.5660580	-0.5865750
P	2.4279060	0.7182220	0.2847390

C	3.8380110	0.7255440	1.4959100
C	4.0836380	2.1040280	2.1125090
C	3.6902690	-0.3508760	2.5756350
C	2.9039710	1.8060810	-1.1494300
C	4.2586430	1.4727570	-1.7688940
C	2.7742010	3.2956000	-0.8271260
C	4.6828710	-3.9188780	-1.3744210
C	-3.5514670	-0.7922300	-1.2313540
C	-2.3225920	-0.7315580	-0.5704470
C	-1.1208830	-0.7062070	-1.3043650
C	-3.6169870	-0.8480000	-2.6256950
C	-2.4093270	-0.8368590	-3.3427910
C	-1.1794510	-0.7634520	-2.7026210
C	-4.9341000	-0.9177950	-3.3486640
P	-2.0416330	-0.6699610	1.2322480
C	-3.6265270	-0.2524270	2.0844790
C	-1.6169970	-2.4511660	1.5976910
C	-0.7601410	-2.6371780	2.8493430
C	-2.8444650	-3.3601130	1.5650880
C	-3.4786200	-0.4127710	3.6003720
C	-4.0916080	1.1542960	1.7182020
V	-0.0470090	0.7348090	0.7930540
C	-0.6478840	2.2711470	0.2182720
C	-1.1915180	3.2223130	-0.8206120
C	-0.8652180	4.6713000	-0.4252540
C	-2.7192530	3.0579380	-0.9017230
C	-0.5875290	2.8987640	-2.1946180
C	0.4936530	0.8849100	2.7615850
H	0.0979130	-3.0515590	-1.7461060
H	2.0992360	-4.4545050	-2.0578000
H	4.5577200	-1.4200650	-0.2775990
H	4.7167860	0.4704670	0.8800830

H	4.4427780	2.8342550	1.3765340
H	4.8558440	2.0230530	2.8927980
H	3.1788260	2.5141820	2.5863700
H	2.9749170	-0.0465730	3.3509500
H	4.6616940	-0.5028000	3.0704400
H	3.3706380	-1.3227120	2.1720070
H	2.1105900	1.5425240	-1.8703750
H	4.4152480	2.0930970	-2.6650400
H	4.3282340	0.4210460	-2.0807890
H	5.0883990	1.6886210	-1.0774310
H	3.6507500	3.6747150	-0.2839090
H	1.8803590	3.5102310	-0.2246800
H	2.7002140	3.8752030	-1.7600300
H	4.8324890	-4.1755860	-2.4345140
H	4.5688520	-4.8659800	-0.8245200
H	5.5989080	-3.4297380	-1.0151750
H	-4.4792430	-0.8039380	-0.6526800
H	-2.4385930	-0.8759680	-4.4348470
H	-0.2548780	-0.7394110	-3.2829580
H	-5.7803660	-0.9253200	-2.6481780
H	-4.9995360	-1.8255360	-3.9679930
H	-5.0599280	-0.0560090	-4.0222560
H	-4.3714500	-0.9807500	1.7218770
H	-0.9858150	-2.7069660	0.7309960
H	-0.5357680	-3.7064580	2.9837000
H	-1.2564120	-2.2873430	3.7659890
H	0.2011780	-2.1117790	2.7601040
H	-3.5084350	-3.1928860	2.4265500
H	-2.5252210	-4.4130890	1.5982560
H	-3.4309730	-3.2241090	0.6436160
H	-3.2776170	-1.4506090	3.8985990
H	-4.4140310	-0.1072130	4.0930540

H	-2.6722270	0.2220920	4.0003470
H	-5.0435120	1.3729360	2.2256160
H	-4.2523810	1.2723350	0.6390850
H	-3.3588360	1.9111050	2.0385290
H	-1.2612330	5.3691380	-1.1793570
H	0.2190480	4.8409200	-0.3520230
H	-1.3189630	4.9325630	0.5435190
H	-3.2026040	3.3109670	0.0523650
H	-3.1218170	3.7348770	-1.6715070
H	-2.9941560	2.0283680	-1.1743880
H	-1.0336130	3.5473880	-2.9642880
H	-0.7842020	1.8548910	-2.4768320
H	0.4976020	3.0660930	-2.2069700
H	-0.4994180	2.6714460	1.2601160
H	-0.4412860	1.1607860	3.2811650
H	0.8172790	-0.1005360	3.1274670
H	1.2569490	1.6354190	3.0106040

6’

Symbol	X	Y	Z
C	-0.9096960	-3.1532580	-0.1083590
C	-1.1053690	-1.7781700	0.0610000
C	-2.3851390	-1.2353580	-0.1595230
C	-1.9756160	-3.9588980	-0.4988450
C	-3.2529370	-3.4347910	-0.7351890
C	-3.4380720	-2.0585220	-0.5534580
N	-0.0897940	-0.8744490	0.4280340
P	-2.4271720	0.5530470	0.2151080
C	-3.9927540	1.1970260	-0.5431040

C	-4.2833860	2.6359930	-0.1155550
C	-4.0236440	1.0558650	-2.0663910
C	-2.6892350	0.4895610	2.0681390
C	-4.0882960	0.0101650	2.4500870
C	-2.2901560	1.7610900	2.8115510
C	-4.3812650	-4.3237420	-1.1841860
C	3.4532690	-1.4523780	1.3180370
C	2.3070570	-1.0437450	0.6307270
C	1.0341290	-1.3253160	1.1531950
C	3.3599780	-2.1412770	2.5299380
C	2.0787660	-2.4183950	3.0333400
C	0.9289890	-2.0207400	2.3636280
C	4.5876490	-2.5660280	3.2890320
P	2.2308520	-0.1710630	-0.9726920
C	3.8406160	0.7182740	-1.1724700
C	2.1758830	-1.5782840	-2.1956080
C	1.5743670	-1.1843070	-3.5447040
C	3.5130330	-2.3021690	-2.3349920
C	3.8782150	1.4575180	-2.5118760
C	4.1163020	1.6590960	-0.0005450
V	-0.0064110	0.8256380	-0.4452600
C	0.5538190	2.1903950	0.5398010
C	0.6531060	3.7057640	0.5022750
C	-0.7645120	4.2997270	0.6059500
C	1.2711460	4.1824450	-0.8233270
C	1.4994050	4.2045950	1.6835310
C	-0.6297060	1.4420780	-2.2489130
H	0.0736500	-3.5931350	0.0695000
H	-1.8074220	-5.0313940	-0.6279520
H	-4.4319250	-1.6325950	-0.7162050
H	-4.7793910	0.5487810	-0.1216180
H	-4.3888540	2.7408940	0.9729760

H	-5.2290790	2.9691460	-0.5696730
H	-3.4955390	3.3269460	-0.4517300
H	-3.3733120	1.7950480	-2.5531430
H	-5.0486780	1.2369890	-2.4244120
H	-3.7245810	0.0558890	-2.4118610
H	-1.9642810	-0.2928620	2.3472850
H	-4.1257720	-0.1961870	3.5308360
H	-4.3680370	-0.9183860	1.9305530
H	-4.8535990	0.7726270	2.2383670
H	-2.9473470	2.6112740	2.5768490
H	-1.2554900	2.0516350	2.5855940
H	-2.3569570	1.5863060	3.8964670
H	-4.2787740	-4.5828930	-2.2504350
H	-5.3576070	-3.8358190	-1.0547640
H	-4.3962760	-5.2682190	-0.6205440
H	4.4424300	-1.2358680	0.9059090
H	1.9830300	-2.9553140	3.9807800
H	-0.0575980	-2.2469240	2.7734800
H	4.6669750	-2.0207580	4.2428910
H	5.5052620	-2.3740440	2.7162020
H	4.5559830	-3.6391880	3.5305860
H	4.6148920	-0.0671360	-1.1813850
H	1.4608790	-2.2603420	-1.7040080
H	1.4824010	-2.0788570	-4.1795900
H	2.1968870	-0.4596320	-4.0899680
H	0.5693530	-0.7548090	-3.4336420
H	4.2614270	-1.6833580	-2.8542290
H	3.3787950	-3.2187970	-2.9295280
H	3.9255000	-2.6013540	-1.3595570
H	3.7960640	0.7746360	-3.3690220
H	4.8315510	1.9978970	-2.6135190
H	3.0669100	2.1991850	-2.5848840

H	5.0874860	2.1558960	-0.1474740
H	4.1509680	1.1375560	0.9657960
H	3.3499460	2.4416460	0.0695650
H	-0.7102350	5.3995540	0.5888740
H	-1.2680750	4.0018940	1.5350500
H	-1.3860450	3.9778080	-0.2422990
H	0.6707760	3.8588240	-1.6859300
H	1.3154560	5.2823780	-0.8411300
H	2.2953380	3.8078410	-0.9585280
H	1.5724130	5.3031480	1.6690960
H	2.5225770	3.8005480	1.6498920
H	1.0501370	3.9104150	2.6448700
H	-1.2353730	2.3599550	-2.1897600
H	0.3259720	1.6790220	-2.7531290
H	-1.1677500	0.6752180	-2.8269720
H	0.8800790	1.6853470	1.4748660

6’-7’-TS

Symbol	X	Y	Z
C	-1.2228210	-3.2353970	-0.3848360
C	-1.2526250	-1.8581490	-0.0961880
C	-2.4947500	-1.1997590	-0.1997000
C	-2.3838660	-3.9059090	-0.7493330
C	-3.6229150	-3.2558880	-0.8520030
C	-3.6524610	-1.8896510	-0.5664420
N	-0.1260470	-1.0978710	0.2331030
P	-2.3673250	0.5520040	0.2489730
C	-3.7878800	1.4091340	-0.5750630
C	-3.9557870	2.8497320	-0.0942090

C	-3.6628830	1.3396620	-2.0973440
C	-2.6688910	0.5258310	2.0898540
C	-4.1101520	0.1506570	2.4323950
C	-2.2053450	1.7778560	2.8338310
C	-4.8604810	-4.0077830	-1.2630910
C	3.3614970	-1.8527580	1.1992820
C	2.2453310	-1.3107310	0.5576620
C	0.9419910	-1.7023810	0.9084070
C	3.2186670	-2.7929860	2.2214250
C	1.9135940	-3.1750610	2.5705830
C	0.7947670	-2.6481200	1.9383110
C	4.4090220	-3.3860140	2.9268340
P	2.2222640	-0.1438340	-0.8301780
C	3.8233320	0.7768320	-0.8004360
C	2.2176920	-1.2891840	-2.3074130
C	2.0206250	-0.6113490	-3.6631630
C	3.4251940	-2.2261160	-2.3301140
C	3.9239580	1.7512680	-1.9760370
C	4.0196560	1.4837430	0.5391510
V	0.0216280	0.8547890	-0.4524730
C	0.4909990	2.3978080	0.3120600
C	0.7703930	3.8433180	0.6433780
C	-0.5711820	4.6034890	0.6189410
C	1.7090320	4.4563310	-0.4058750
C	1.3791410	3.9497010	2.0498510
C	-0.1261120	1.5758240	-2.0782610
H	-0.2802350	-3.7827360	-0.3282070
H	-2.3243080	-4.9745060	-0.9753580
H	-4.6043890	-1.3557200	-0.6390770
H	-4.6821090	0.8386760	-0.2725330
H	-4.2025250	2.9087280	0.9746590
H	-4.7785560	3.3298600	-0.6456170

H	-3.0483360	3.4462970	-0.2714940
H	-2.8351170	1.9697570	-2.4574920
H	-4.5875000	1.7154330	-2.5614340
H	-3.5023460	0.3143320	-2.4623530
H	-2.0107910	-0.3056860	2.3947050
H	-4.1996740	-0.0354350	3.5136200
H	-4.4326730	-0.7645590	1.9144570
H	-4.8120200	0.9606640	2.1800520
H	-2.7966110	2.6676200	2.5733410
H	-1.1467130	2.0049010	2.6470410
H	-2.3158790	1.6200070	3.9174900
H	-5.0736520	-4.8366260	-0.5701310
H	-4.7474980	-4.4463610	-2.2671260
H	-5.7429140	-3.3525250	-1.2823250
H	4.3663500	-1.5431840	0.8969670
H	1.7715060	-3.9055410	3.3721430
H	-0.2034000	-2.9704150	2.2415600
H	4.3684760	-3.1962020	4.0108780
H	5.3528530	-2.9679710	2.5494260
H	4.4488300	-4.4781640	2.7908590
H	4.6060010	0.0064240	-0.9076760
H	1.3157580	-1.8886000	-2.0916260
H	1.7239060	-1.3662690	-4.4070570
H	2.9489360	-0.1490210	-4.0276290
H	1.2415870	0.1627400	-3.6462500
H	4.3608070	-1.6781760	-2.5248820
H	3.3061530	-2.9602960	-3.1419930
H	3.5386910	-2.7873970	-1.3921810
H	4.0311670	1.2343740	-2.9383200
H	4.8073070	2.3960350	-1.8518620
H	3.0380380	2.4018290	-2.0374390
H	4.9905400	2.0021810	0.5486220

H	3.9989280	0.7932900	1.3934720
H	3.2379480	2.2371610	0.6985330
H	-0.4110230	5.6544590	0.9035550
H	-1.2943740	4.1670530	1.3231010
H	-1.0176270	4.5962240	-0.3882500
H	1.3053330	4.3388430	-1.4235300
H	1.8275090	5.5338310	-0.2164980
H	2.7065790	3.9979990	-0.3796610
H	1.5928540	5.0024140	2.2881130
H	2.3204890	3.3886100	2.1369810
H	0.6821250	3.5690190	2.8128440
H	-0.3170220	2.4041040	-0.8750920
H	-0.0266180	2.3136310	-2.8783560
H	-0.7407870	0.6784450	-2.3482070
H	0.2863820	1.7291550	1.2057340

6’-7”-TS

Symbol	X	Y	Z
C	-1.0554460	-3.3187330	-0.3504800
C	-1.1430750	-1.9369950	-0.0950870
C	-2.4075760	-1.3295800	-0.2172690
C	-2.1867130	-4.0438660	-0.7019130
C	-3.4498280	-3.4456660	-0.8233830
C	-3.5345730	-2.0752420	-0.5713920
N	-0.0545830	-1.1190330	0.2176680
P	-2.3528370	0.4363600	0.2095720
C	-3.8475720	1.1724130	-0.6028130
C	-4.1129260	2.6159180	-0.1840930
C	-3.7928990	1.0370960	-2.1244290

C	-2.6575760	0.3921940	2.0506700
C	-4.0803100	-0.0425000	2.3984340
C	-2.2431990	1.6606970	2.7898450
C	-4.6551890	-4.2539650	-1.2227540
C	3.4807880	-1.7394570	1.1386060
C	2.3385330	-1.2360400	0.5077240
C	1.0519910	-1.6691230	0.8755310
C	3.3848970	-2.6783020	2.1656610
C	2.0967940	-3.0967880	2.5375600
C	0.9542390	-2.6090010	1.9185990
C	4.6036830	-3.2310100	2.8545830
P	2.2940590	-0.0365860	-0.8476780
C	3.8070490	1.0165910	-0.6745770
C	2.4473130	-1.0847470	-2.3795260
C	2.2522300	-0.3193070	-3.6902490
C	3.7224700	-1.9257330	-2.4019220
C	3.8964570	2.0601740	-1.7898500
C	3.8678790	1.6670080	0.7087200
V	-0.0236190	0.8389310	-0.4700640
C	0.2754950	2.3973350	0.3515590
C	0.3612760	3.8656740	0.7004080
C	-1.0218760	4.5016590	0.5034870
C	1.3643760	4.5610270	-0.2395150
C	0.8207130	4.0367120	2.1560920
C	-0.4716820	1.6157830	-2.0070260
H	-0.0918530	-3.8261570	-0.2792150
H	-2.0833070	-5.1143470	-0.9017590
H	-4.5074650	-1.5842510	-0.6614130
H	-4.6804310	0.5521280	-0.2310180
H	-4.2580860	2.7213870	0.9000240
H	-5.0300590	2.9777510	-0.6736330
H	-3.2907840	3.2776050	-0.4901190

H	-3.0560220	1.7289170	-2.5577330
H	-4.7744790	1.2946790	-2.5509330
H	-3.5427390	0.0182460	-2.4535810
H	-1.9626770	-0.4099200	2.3525870
H	-4.1551940	-0.2355100	3.4796060
H	-4.3704940	-0.9682260	1.8800610
H	-4.8151480	0.7405020	2.1549640
H	-2.8893310	2.5182430	2.5506890
H	-1.2069520	1.9379500	2.5583750
H	-2.3088720	1.4945880	3.8760370
H	-4.5240710	-4.7024840	-2.2202600
H	-5.5637900	-3.6358530	-1.2517010
H	-4.8353380	-5.0807420	-0.5180390
H	4.4701490	-1.3910450	0.8272820
H	1.9882950	-3.8231740	3.3480330
H	-0.0285510	-2.9568300	2.2425280
H	4.6861820	-4.3188880	2.7040120
H	4.5641460	-3.0566230	3.9411420
H	5.5265770	-2.7702640	2.4750700
H	4.6609860	0.3243750	-0.7697730
H	1.5856370	-1.7620430	-2.2438090
H	1.9969460	-1.0250820	-4.4950780
H	3.1722100	0.1978660	-3.9978290
H	1.4506870	0.4314840	-3.6358020
H	4.6206070	-1.2956930	-2.5022620
H	3.7030680	-2.6056320	-3.2676910
H	3.8287400	-2.5434900	-1.4989410
H	4.0731580	1.6090190	-2.7745530
H	4.7337490	2.7462020	-1.5903380
H	2.9796180	2.6666420	-1.8547640
H	4.8101740	2.2264310	0.8118750
H	3.8234620	0.9333630	1.5253930

H	3.0467450	2.3831560	0.8525190
H	-0.9792010	5.5768050	0.7351910
H	-1.7704120	4.0390370	1.1606020
H	-1.3606400	4.3941040	-0.5380610
H	1.0721500	4.4512080	-1.2967150
H	1.3940140	5.6383890	-0.0175910
H	2.3830100	4.1662380	-0.1190100
H	0.8773640	5.1061050	2.4087570
H	1.8189590	3.6013350	2.3171190
H	0.1215040	3.5650100	2.8616530
H	0.4307620	2.2958450	-1.0747410
H	-0.7457530	2.3878380	-2.7287680
H	-0.4132150	0.5767620	-2.4244600
H	0.8385020	1.6829000	1.0282390

7’

Symbol	X	Y	Z
C	-1.0560900	-3.1092840	-0.7018820
C	-1.1718140	-1.7823340	-0.2650030
C	-2.4353420	-1.1671340	-0.2644080
C	-2.1819310	-3.8033710	-1.1220300
C	-3.4514580	-3.2054720	-1.1371230
C	-3.5534870	-1.8807260	-0.7075400
N	-0.0539590	-1.0160350	0.1271220
P	-2.4284850	0.5586840	0.3521680
C	-3.9363960	1.3248480	-0.4194510
C	-4.3242210	2.6762860	0.1807650
C	-3.7957370	1.4155120	-1.9384510
C	-2.7517490	0.3403320	2.1757960

C	-4.1452580	-0.2077270	2.4777370
C	-2.4257650	1.5826780	3.0073080
C	-4.6582260	-3.9788590	-1.5935640
C	3.3537410	-1.7074740	1.3601910
C	2.2893280	-1.1899140	0.6219580
C	0.9766480	-1.6046740	0.8941830
C	3.1403180	-2.6431150	2.3784570
C	1.8225310	-3.0520560	2.6276940
C	0.7488290	-2.5468680	1.9024830
C	4.2801890	-3.1924400	3.1926310
P	2.3460420	-0.0008650	-0.7617870
C	4.0170280	0.8088080	-0.6493380
C	2.3974000	-1.1410240	-2.2424240
C	2.1840670	-0.4380030	-3.5840770
C	3.6236050	-2.0512510	-2.2672810
C	4.3136890	1.6819860	-1.8708030
C	4.2551290	1.5824160	0.6509090
V	-0.0156980	0.8002490	-0.3810610
C	0.8008390	2.2571850	0.7465500
C	0.5923910	3.7414370	0.4106530
C	-0.8828100	4.0240400	0.1054680
C	1.4604300	4.1397680	-0.7896210
C	1.0141620	4.5760810	1.6321990
C	-0.4831060	1.0619970	-2.0735480
H	-0.0772470	-3.5928540	-0.7103920
H	-2.0737100	-4.8375890	-1.4595340
H	-4.5362110	-1.4028920	-0.7187040
H	-4.7476990	0.6159110	-0.1842840
H	-4.5419090	2.6149190	1.2554570
H	-5.2391390	3.0366430	-0.3138800
H	-3.5474870	3.4376450	0.0253100
H	-3.0252000	2.1473080	-2.2246940

H	-4.7495000	1.7498780	-2.3748230
H	-3.5392350	0.4508680	-2.3993450
H	-2.0092450	-0.4361200	2.4306450
H	-4.2224360	-0.4585450	3.5469760
H	-4.3633960	-1.1236700	1.9090880
H	-4.9306310	0.5320530	2.2590810
H	-3.1090090	2.4188250	2.8009690
H	-1.3984660	1.9383920	2.8462280
H	-2.5205970	1.3422340	4.0772590
H	-4.8559250	-4.8317810	-0.9255050
H	-4.5100570	-4.3870890	-2.6050480
H	-5.5594890	-3.3505230	-1.6112420
H	4.3759020	-1.3864990	1.1433720
H	1.6313380	-3.7829510	3.4179030
H	-0.2677290	-2.8815300	2.1188390
H	4.2137300	-2.8574430	4.2398860
H	5.2532180	-2.8674690	2.7990360
H	4.2687660	-4.2928470	3.2027060
H	4.7151540	-0.0454190	-0.6629650
H	1.5048220	-1.7595910	-2.0457750
H	1.9042260	-1.1819310	-4.3455420
H	3.0962980	0.0600890	-3.9404080
H	1.3831160	0.3135460	-3.5410970
H	4.5466530	-1.4873170	-2.4744180
H	3.5154460	-2.7960390	-3.0711380
H	3.7566380	-2.6012450	-1.3240750
H	4.4268850	1.0927080	-2.7896980
H	5.2590720	2.2231970	-1.7131790
H	3.5266080	2.4325500	-2.0389130
H	5.3333520	1.7744010	0.7616800
H	3.9227570	1.0402360	1.5477980
H	3.7559180	2.5614050	0.6343630

H	-1.0474390	5.0939010	-0.0980680
H	-1.5185460	3.7477910	0.9615610
H	-1.2349940	3.4631960	-0.7745250
H	1.2032260	3.5590860	-1.6896260
H	1.3299460	5.2063470	-1.0303080
H	2.5286840	3.9771770	-0.5798190
H	0.8811920	5.6522780	1.4369150
H	2.0731430	4.4083950	1.8840280
H	0.4131920	4.3188340	2.5187740
H	0.1353420	1.9439320	1.5844530
H	-0.3351210	2.0701450	-2.4981890
H	-0.9449250	0.3350660	-2.7577180
H	1.8245140	2.0717300	1.0887830

7”

Symbol	X	Y	Z
C	-1.0493730	-3.1090870	-0.7035900
C	-1.1683210	-1.7838960	-0.2635650
C	-2.4331740	-1.1705950	-0.2642420
C	-2.1729410	-3.8033320	-1.1308380
C	-3.4427880	-3.2071870	-1.1488990
C	-3.5482890	-1.8840530	-0.7137330
N	-0.0524820	-1.0155550	0.1302070
P	-2.4297750	0.5545610	0.3547030
C	-3.9394490	1.3191800	-0.4148110
C	-4.3298390	2.6681470	0.1893170
C	-3.7991000	1.4145630	-1.9335690
C	-2.7515130	0.3326290	2.1780930
C	-4.1434050	-0.2196400	2.4798060

C	-2.4282460	1.5745320	3.0113530
C	-4.6507090	-3.9806170	-1.6024570
C	3.3562840	-1.6984610	1.3645760
C	2.2912580	-1.1851730	0.6243690
C	0.9794230	-1.6028480	0.8964750
C	3.1446560	-2.6340290	2.3833870
C	1.8277120	-3.0452380	2.6327990
C	0.7531920	-2.5439570	1.9060110
C	4.2885290	-3.2010690	3.1796040
P	2.3455530	0.0035360	-0.7600160
C	4.0156570	0.8150860	-0.6493140
C	2.3975030	-1.1383280	-2.2393810
C	2.1819230	-0.4372540	-3.5816980
C	3.6250770	-2.0467070	-2.2641220
C	4.3116830	1.6858290	-1.8726860
C	4.2524390	1.5918290	0.6492870
V	-0.0178430	0.8001910	-0.3791390
C	0.7963650	2.2605250	0.7461310
C	0.5862340	3.7437740	0.4066830
C	-0.8895100	4.0243380	0.1022070
C	1.4527690	4.1398970	-0.7954210
C	1.0084420	4.5819610	1.6256350
C	-0.4874380	1.0588460	-2.0715680
H	-0.0695890	-3.5907770	-0.7116410
H	-2.0614520	-4.8355100	-1.4731830
H	-4.5316320	-1.4074340	-0.7277280
H	-4.7492650	0.6079170	-0.1815370
H	-4.5487920	2.6029220	1.2635060
H	-5.2445660	3.0289900	-0.3053200
H	-3.5538410	3.4309690	0.0374100
H	-3.0304530	2.1491550	-2.2177160
H	-4.7537310	1.7478220	-2.3688880

H	-3.5401530	0.4519960	-2.3973810
H	-2.0068000	-0.4422330	2.4313460
H	-4.2191550	-0.4731400	3.5485150
H	-4.3596560	-1.1347760	1.9090980
H	-4.9308500	0.5186100	2.2634400
H	-3.1147040	2.4087020	2.8077820
H	-1.4024420	1.9340960	2.8491680
H	-2.5203810	1.3317460	4.0810120
H	-4.4402980	-4.5475710	-2.5214330
H	-5.5051970	-3.3181090	-1.7993680
H	-4.9619130	-4.7082090	-0.8357220
H	4.3774760	-1.3729890	1.1503710
H	1.6378880	-3.7738110	3.4254060
H	-0.2629820	-2.8788590	2.1238480
H	4.4533360	-4.2619090	2.9324220
H	4.0867130	-3.1479460	4.2600940
H	5.2261850	-2.6627540	2.9831870
H	4.7146340	-0.0384890	-0.6610880
H	1.5059890	-1.7579820	-2.0413130
H	1.9026780	-1.1825450	-4.3420510
H	3.0930080	0.0619900	-3.9393460
H	1.3797230	0.3129710	-3.5388890
H	4.5470820	-1.4816460	-2.4728040
H	3.5173790	-2.7927730	-3.0668590
H	3.7597870	-2.5951540	-1.3202400
H	4.4256190	1.0945320	-2.7901970
H	5.2565620	2.2282130	-1.7160830
H	3.5239870	2.4353230	-2.0426980
H	5.3303600	1.7855650	0.7599420
H	3.9204720	1.0512740	1.5473020
H	3.7518110	2.5700450	0.6303480
H	-1.0552020	5.0933460	-0.1048730

H	-1.5239900	3.7505270	0.9600260
H	-1.2422700	3.4599710	-0.7753360
H	1.1953940	3.5566830	-1.6937330
H	1.3209970	5.2057320	-1.0386920
H	2.5213720	3.9789670	-0.5861390
H	0.8740790	5.6575150	1.4277740
H	2.0678820	4.4160760	1.8767050
H	0.4087620	4.3262810	2.5135310
H	0.1313280	1.9485210	1.5848420
H	-0.3417340	2.0663420	-2.4984860
H	-0.9489820	0.3297210	-2.7536140
H	1.8203150	2.0771500	1.0887130

V^V (L_1)

1

Symbol	X	Y	Z
C	0.6460070	3.3633290	-1.5198510
C	0.9488030	2.1935720	-0.7992210
C	2.2757270	1.7223830	-0.8178470
C	1.6341520	4.0453610	-2.2186760
C	2.9519470	3.5887610	-2.2145040
C	3.2635470	2.4328880	-1.5069010
N	-0.0259720	1.4758130	-0.0768250

P	2.4994730	0.2058530	0.1622710
C	3.9206950	-0.7996780	-0.5998870
C	3.8329800	-2.2186930	-0.0183120
C	3.7107400	-0.8550760	-2.1214450
C	3.0020360	0.8639470	1.8823560
C	4.0089360	2.0176830	1.7751910
C	3.5874080	-0.2530160	2.7527800
C	-3.3197100	2.4465100	1.3397250
C	-2.3225000	1.7119570	0.6872050
C	-1.0148250	2.2258530	0.5840190
C	-3.0392460	3.6789050	1.9166080
C	-1.7426260	4.1880220	1.8270090
C	-0.7469240	3.4789180	1.1700050
P	-2.5460950	0.1151990	-0.1481650
C	-3.7849960	-0.9200420	0.8384230
C	-3.2579610	0.6083490	-1.8498370
C	-3.6962260	-0.6388630	-2.6255360
C	-4.4373570	1.5826870	-1.7192770
C	-3.6007720	-2.3768460	0.3904020
C	-3.3899250	-0.7697660	2.3148140
V	-0.0000670	-0.5274510	-0.0571730
C	0.0145200	-1.6898930	1.2491170
C	0.0927430	-2.5716000	2.4728560
C	-1.0975920	-3.5503460	2.5056410
C	0.1139890	-1.7466970	3.7662510
C	1.3713070	-3.4264810	2.3691480
C	0.1374170	-1.4488340	-1.8189870
H	-0.3724980	3.7524650	-1.5189650
H	1.3695220	4.9482930	-2.7734030
H	4.2967570	2.0882420	-1.4891500
H	4.1513100	-2.2542480	1.0314520
H	4.5006210	-2.8883600	-0.5833630

H	2.8185980	-2.6265210	-0.0807000
H	2.7071940	-1.1929650	-2.3991900
H	4.4282790	-1.5718800	-2.5512870
H	3.8836670	0.1148510	-2.6054700
H	4.2450830	2.3680500	2.7930090
H	3.5965350	2.8723850	1.2212390
H	4.9552790	1.7258630	1.3040300
H	4.5713600	-0.5961450	2.4085310
H	2.9207780	-1.1210200	2.8160300
H	3.7203660	0.1299030	3.7769310
H	-4.3361690	2.0561920	1.3922790
H	-1.5017600	5.1536780	2.2770130
H	0.2539930	3.9061030	1.1000890
H	-3.9768850	-0.3461740	-3.6496850
H	-4.5715640	-1.1272330	-2.1759130
H	-2.8919270	-1.3839120	-2.7107710
H	-5.2958930	1.1591560	-1.1873300
H	-4.7810770	1.8480050	-2.7320880
H	-4.1431640	2.5169560	-1.2211230
H	-3.9443080	-2.5396970	-0.6400510
H	-4.1931030	-3.0398340	1.0412250
H	-2.5537600	-2.6906650	0.4526940
H	-3.8792720	-1.5591370	2.9070460
H	-3.7038590	0.1974020	2.7291410
H	-2.3052000	-0.8574850	2.4602800
H	-0.9493870	-4.2658000	3.3288750
H	-1.1711190	-4.1294630	1.5722450
H	-2.0520510	-3.0406240	2.6757700
H	-0.7515720	-1.0720490	3.8353090
H	0.0845700	-2.4192350	4.6368420
H	1.0235380	-1.1378400	3.8487290
H	1.4634990	-4.0631600	3.2622630

H	2.2765200	-2.8136580	2.2977880
H	1.3394040	-4.0873910	1.4883660
H	-0.8727700	-0.9975320	-1.9608980
H	0.8296360	-0.7864570	-2.3686770
H	0.1587300	-2.3095850	0.2903460
C	1.7379590	1.4079360	2.5612050
H	1.3584310	2.2991450	2.0499270
H	0.9255770	0.6706020	2.6037490
H	1.9867440	1.7022270	3.5933270
C	-5.2678880	-0.5590800	0.6854120
H	-5.6429710	-0.7404490	-0.3305610
H	-5.8481230	-1.2067910	1.3623600
H	-5.4958160	0.4785630	0.9614820
C	-2.1587160	1.3379180	-2.6365380
H	-1.8187830	2.2396910	-2.1125960
H	-1.2816670	0.7142320	-2.8542200
H	-2.5749270	1.6593610	-3.6043490
C	5.3355500	-0.2612660	-0.3389680
H	6.0492100	-0.9224650	-0.8565160
H	5.4973780	0.7508250	-0.7307250
H	5.6085620	-0.2676190	0.7230660
H	-3.8241340	4.2391060	2.4271150
H	3.7309150	4.1286810	-2.7551120
C	0.1039240	-2.8845930	-2.3772550
C	-1.1247530	-3.6405060	-1.8601770
H	-1.1738120	-4.6439000	-2.3092890
H	-1.0924040	-3.7757890	-0.7681030
H	-2.0617710	-3.1222980	-2.1079050
C	0.0093720	-2.7681160	-3.9096450
H	0.8974330	-2.2680060	-4.3268050
H	-0.0656570	-3.7635730	-4.3746680
H	-0.8766620	-2.1884300	-4.2138050

C	1.3454360	-3.7032910	-2.0137840
H	1.4177720	-3.8575780	-0.9259680
H	1.2845150	-4.6993920	-2.4784780
H	2.2758900	-3.2344980	-2.3593460

1-2-TS

Symbol	X	Y	Z
C	0.6592530	-3.4161850	1.5506190
C	0.9535500	-2.2478950	0.8191730
C	2.2699570	-1.7426450	0.8788080
C	1.6394830	-4.0606490	2.2938590
C	2.9447700	-3.5695500	2.3301640
C	3.2497350	-2.4162880	1.6151810
N	-0.0106430	-1.5646710	0.0629780
P	2.4973930	-0.2447130	-0.1301410
C	3.8795220	0.8072100	0.6444120
C	3.7814100	2.2067790	0.0204750
C	3.6273220	0.9137810	2.1570440
C	3.0582800	-0.9369640	-1.8181330
C	4.0965870	-2.0557950	-1.6556160
C	3.6276110	0.1769140	-2.7037860
C	-3.3657150	-2.5949120	-1.1337510
C	-2.3355890	-1.8333500	-0.5685470
C	-1.0186170	-2.3328330	-0.5350330
C	-3.1069850	-3.8381140	-1.6974290
C	-1.7993080	-4.3283960	-1.6882310
C	-0.7719250	-3.5937100	-1.1138890
P	-2.5098520	-0.1934410	0.1938140
C	-3.8022180	0.7814910	-0.7821760

C	-3.0927040	-0.5391680	1.9698560
C	-3.5408960	0.7582040	2.6539100
C	-4.2276240	-1.5706520	2.0102690
C	-3.6063400	2.2627880	-0.4310540
C	-3.4858570	0.5487020	-2.2681270
V	-0.0197490	0.4785210	0.0117160
C	-0.0855920	1.4922860	-1.3318700
C	-0.0004960	2.4118170	-2.5223210
C	-1.2251980	3.3338930	-2.6584820
C	0.1169770	1.5619700	-3.8000400
C	1.2428690	3.3058330	-2.3702240
C	0.0924340	1.9198990	1.4771710
H	-0.3504270	-3.8285460	1.5257840
H	1.3781260	-4.9612170	2.8542710
H	4.2739740	-2.0446830	1.6288740
H	4.1078350	2.2181200	-1.0272070
H	4.4332630	2.9021740	0.5729390
H	2.7603230	2.6010400	0.0619810
H	2.6347490	1.3124330	2.3977470
H	4.3632090	1.6106590	2.5887230
H	3.7387360	-0.0474740	2.6751570
H	4.3669750	-2.4277180	-2.6571830
H	3.6946170	-2.9065840	-1.0880940
H	5.0229120	-1.7250910	-1.1711720
H	4.5946600	0.5548530	-2.3474940
H	2.9384250	1.0253100	-2.7979980
H	3.7915010	-0.2235340	-3.7166970
H	-4.3883970	-2.2180900	-1.1315440
H	-1.5770240	-5.2997660	-2.1357170
H	0.2386560	-4.0042950	-1.0987840
H	-3.7241480	0.5548210	3.7209760
H	-4.4757690	1.1529870	2.2344170

H	-2.7793200	1.5520840	2.6050950
H	-5.1253470	-1.2450980	1.4710110
H	-4.5178930	-1.7358590	3.0605100
H	-3.9105320	-2.5404070	1.6013580
H	-3.9328050	2.4906830	0.5926280
H	-4.2085620	2.8871530	-1.1102960
H	-2.5600440	2.5675740	-0.5332590
H	-4.0126590	1.3020880	-2.8750120
H	-3.8164540	-0.4410130	-2.6090690
H	-2.4104100	0.6285930	-2.4798730
H	-1.0580200	4.0153360	-3.5064440
H	-1.3809560	3.9546790	-1.7655170
H	-2.1463650	2.7754810	-2.8586710
H	-0.7368200	0.8773670	-3.9096470
H	0.1357130	2.2221480	-4.6806830
H	1.0363140	0.9630390	-3.8109620
H	1.3614410	3.9320830	-3.2675650
H	2.1599550	2.7191220	-2.2433710
H	1.1447320	3.9796190	-1.5053770
H	-0.9105990	1.4712190	1.6825180
H	0.8170000	1.3268360	2.0642820
H	0.1760400	2.1364300	0.0172020
C	1.8284070	-1.5424860	-2.5092280
H	1.4627760	-2.4287050	-1.9779690
H	0.9940280	-0.8345120	-2.5932100
H	2.1146250	-1.8614960	-3.5243520
C	-5.2700450	0.4188930	-0.5212350
H	-5.5823800	0.6421190	0.5075590
H	-5.8978560	1.0321330	-1.1878490
H	-5.5058580	-0.6313560	-0.7357700
C	-1.8978850	-1.1211660	2.7401620
H	-1.5248410	-2.0472730	2.2857190

H	-1.0563410	-0.4173800	2.8240920
H	-2.2186770	-1.3624140	3.7660650
C	5.3102010	0.2873150	0.4391560
H	6.0004530	0.9764950	0.9518280
H	5.4784150	-0.7095320	0.8658640
H	5.6080120	0.2651360	-0.6160830
H	-3.9167810	-4.4204140	-2.1398930
H	3.7176510	-4.0800710	2.9067900
C	0.0617840	3.3784740	1.9776990
C	-1.0707650	4.1531020	1.3029030
H	-1.1018170	5.1886150	1.6729870
H	-0.9302430	4.1982890	0.2124980
H	-2.0510440	3.6967010	1.5035350
C	-0.1937740	3.3213080	3.4933460
H	0.6168560	2.7909650	4.0175240
H	-0.2544760	4.3375000	3.9120160
H	-1.1399850	2.8077600	3.7258880
C	1.3873160	4.0995410	1.7247990
H	1.5828880	4.2261960	0.6498560
H	1.3560320	5.1050180	2.1709280
H	2.2379730	3.5640510	2.1697300

A

Symbol	X	Y	Z
C	-0.7563410	-2.8225020	1.7549060
C	-0.9981190	-1.6156720	1.0681770
C	-2.2515390	-1.4631260	0.4148040
C	-1.7068330	-3.8341250	1.7823010

C	-2.9299570	-3.6883320	1.1271220
C	-3.1902070	-2.5021830	0.4513990
N	-0.0436310	-0.5942700	0.9764700
P	-2.5430190	0.1535430	-0.3803690
C	-3.1400050	-0.0946700	-2.1511540
C	-2.9703410	1.2491270	-2.8818810
C	-2.1607290	-1.1067830	-2.7662360
C	-3.7338970	1.0493270	0.7612370
C	-5.0263490	0.2702590	1.0298600
C	-4.0686360	2.4191830	0.1601350
C	3.5588530	-0.7452150	1.8114150
C	2.3739990	-0.6265330	1.0777160
C	1.1328620	-0.6440990	1.7541050
C	3.5256410	-0.8411820	3.1998920
C	2.3001210	-0.8080480	3.8669990
C	1.1107210	-0.7145820	3.1519160
P	2.2675350	-0.3303150	-0.7315330
C	3.6838630	0.8141350	-1.2563210
C	2.3976260	-2.0240970	-1.5667310
C	2.3948790	-1.8357680	-3.0897630
C	3.6185600	-2.8334820	-1.1144700
C	3.1695600	1.5787560	-2.4891530
C	3.9408410	1.8127590	-0.1208320
V	-0.0901690	0.5801530	-0.5434440
C	0.2701000	2.0827230	0.0341310
C	0.4737250	3.4180230	0.6895560
C	1.3640320	4.3158490	-0.1905670
C	1.0867670	3.2172210	2.0868840
C	-0.8885960	4.1223340	0.8318910
H	0.1971490	-2.9755870	2.2604680
H	-1.4823290	-4.7575130	2.3210720
H	-4.1524070	-2.3774990	-0.0475010

H	-3.6651340	2.0169090	-2.5179630
H	-3.1713030	1.1063900	-3.9555420
H	-1.9492930	1.6571120	-2.7895850
H	-1.1107410	-0.7679850	-2.6806870
H	-2.3647320	-1.2124710	-3.8434070
H	-2.2339530	-2.1022790	-2.3061380
H	-5.6546300	0.8635540	1.7137540
H	-4.8339870	-0.6929670	1.5223580
H	-5.6151810	0.0899030	0.1212820
H	-4.7597280	2.3290690	-0.6908650
H	-3.1725520	2.9609020	-0.1711810
H	-4.5704410	3.0380010	0.9206700
H	4.5206350	-0.7607010	1.2965770
H	2.2711200	-0.8596770	4.9574740
H	0.1497730	-0.7014660	3.6700420
H	2.3457000	-2.8230580	-3.5757690
H	3.2984500	-1.3347760	-3.4627290
H	1.5183470	-1.2617610	-3.4322820
H	4.5690550	-2.4077700	-1.4545260
H	3.5475660	-3.8495510	-1.5352030
H	3.6524750	-2.9369480	-0.0195960
H	2.9187600	0.9098990	-3.3259330
H	3.9521110	2.2675450	-2.8454220
H	2.2801170	2.1796190	-2.2454190
H	4.4626360	2.6942720	-0.5252710
H	4.5724430	1.3908460	0.6706410
H	3.0082450	2.1509190	0.3421010
H	1.4680900	5.3066280	0.2787650
H	0.9130300	4.4621320	-1.1840510
H	2.3714410	3.9084370	-0.3306060
H	2.0666790	2.7207200	2.0440170
H	1.2219630	4.1910390	2.5836280

H	0.4286610	2.6015150	2.7183130
H	-0.7425630	5.1177250	1.2799460
H	-1.5761930	3.5628930	1.4785890
H	-1.3666390	4.2638980	-0.1491600
C	-2.9714800	1.2027870	2.0886140
H	-2.8413840	0.2315490	2.5887710
H	-1.9755080	1.6457450	1.9527830
H	-3.5508920	1.8507610	2.7651940
C	5.0087280	0.1233070	-1.5989700
H	4.9398180	-0.5273100	-2.4804700
H	5.7535480	0.9017990	-1.8299310
H	5.4067350	-0.4661940	-0.7610950
C	1.1413780	-2.8103230	-1.1703160
H	1.1035630	-3.0083180	-0.0912590
H	0.2087240	-2.3033060	-1.4604330
H	1.1519140	-3.7851860	-1.6831570
C	-4.5796820	-0.5818040	-2.3261100
H	-4.7792530	-0.7393320	-3.3984820
H	-4.7691060	-1.5364660	-1.8178590
H	-5.3088650	0.1574560	-1.9669160
H	4.4564440	-0.9347960	3.7620480
H	-3.6740930	-4.4859360	1.1490500

5-6-TS

Symbol	X	Y	Z
C	-0.7624120	-3.2740990	0.9439160
C	-1.0215950	-1.9301970	0.6081830
C	-2.2955790	-1.6030380	0.0912140
C	-1.7362210	-4.2509470	0.7804570
C	-2.9965520	-3.9242610	0.2821840
C	-3.2638940	-2.6017590	-0.0548380

N	-0.0609070	-0.9182540	0.7302560
P	-2.5268830	0.1722630	-0.2702640
C	-3.6156380	0.3323410	-1.8166380
C	-3.5092880	1.7789820	-2.3323400
C	-3.0462180	-0.6414210	-2.8627370
C	-3.4158500	0.8155580	1.2770710
C	-4.5665800	-0.0947210	1.7251000
C	-3.9454130	2.2307290	1.0158240
C	3.4607560	-1.3938150	1.7864990
C	2.3484520	-1.0285420	1.0188100
C	1.0522690	-1.1676080	1.5598050
C	3.3045520	-1.8410300	3.0940530
C	2.0256120	-1.9119170	3.6506090
C	0.9094230	-1.5827680	2.8921090
P	2.3908750	-0.2474780	-0.6340020
C	3.9057470	0.8821320	-0.7208920
C	2.4694590	-1.6278170	-1.9266460
C	2.7933880	-1.0532090	-3.3122950
C	3.4704090	-2.7240580	-1.5490150
C	3.6215710	1.9368250	-1.8038690
C	4.0391820	1.5914930	0.6344900
V	0.0366320	0.6537930	-0.4403800
C	0.3082360	2.1021960	0.3498900
C	0.4997070	3.4094400	1.0612700
C	1.5197380	4.2807990	0.3061650
C	0.9745970	3.1394320	2.4991760
C	-0.8414530	4.1636470	1.1055030
C	-0.0352490	1.6276740	-2.4141600
H	0.2182980	-3.5595240	1.3253850
H	-1.5022080	-5.2852420	1.0425350
H	-4.2531440	-2.3482090	-0.4335280
H	-3.9953860	2.4932900	-1.6556730

H	-4.0254370	1.8508610	-3.3030320
H	-2.4754350	2.1085110	-2.4825130
H	-1.9564420	-0.5566010	-2.9861450
H	-3.5044170	-0.4316360	-3.8421150
H	-3.2624400	-1.6881550	-2.6097730
H	-5.0359910	0.3524350	2.6164730
H	-4.2115510	-1.0948400	2.0100800
H	-5.3524930	-0.2081780	0.9686460
H	-4.8369680	2.2240140	0.3731830
H	-3.1931610	2.8815870	0.5507860
H	-4.2388960	2.6915060	1.9724040
H	4.4638420	-1.3307170	1.3631640
H	1.8978380	-2.2362800	4.6855810
H	-0.0921700	-1.6680800	3.3175510
H	2.7240700	-1.8639370	-4.0548890
H	3.8072530	-0.6394720	-3.3824440
H	2.0776020	-0.2745230	-3.6178280
H	4.5080330	-2.3664880	-1.5387230
H	3.4111830	-3.5366090	-2.2912670
H	3.2436490	-3.1591040	-0.5645320
H	3.5078940	1.5000840	-2.8052630
H	4.4669150	2.6417330	-1.8504080
H	2.7174530	2.5171670	-1.5743960
H	4.6734850	2.4837370	0.5149920
H	4.5044790	0.9545650	1.3969330
H	3.0666630	1.9158420	1.0228030
H	1.6127250	5.2560370	0.8081290
H	1.1979570	4.4713570	-0.7298400
H	2.5163680	3.8243770	0.2806190
H	1.9441190	2.6231980	2.5235680
H	1.0841890	4.0920910	3.0399610
H	0.2519900	2.5175310	3.0474300

H	-0.6890960	5.1606140	1.5469580
H	-1.5787460	3.6354970	1.7233070
H	-1.2626300	4.3035710	0.0978400
H	0.9792720	1.3887990	-2.7651020
H	-0.7546900	0.9563480	-2.8973700
H	-0.2609190	2.6474570	-2.7601650
H	0.0628750	2.1935300	-1.1096020
C	-2.3740040	0.8435470	2.4046380
H	-1.9968490	-0.1639540	2.6332480
H	-1.5105590	1.4728210	2.1576790
H	-2.8448040	1.2389590	3.3190590
C	5.2283920	0.1709200	-1.0363440
H	5.2550140	-0.2585140	-2.0460850
H	6.0417560	0.9119160	-0.9790960
H	5.4657660	-0.6251170	-0.3170610
C	1.0623390	-2.2389290	-1.9850720
H	0.7934120	-2.7579040	-1.0573210
H	0.2870240	-1.4798290	-2.1917400
H	1.0194310	-2.9776320	-2.8009070
C	-5.1087180	0.0276800	-1.6270700
H	-5.6048980	0.1206960	-2.6066140
H	-5.3097330	-0.9871930	-1.2626670
H	-5.5977890	0.7413800	-0.9514240
H	4.1792470	-2.1248050	3.6818010
H	-3.7640000	-4.6897530	0.1580230

6

Symbol	X	Y	Z

C	-0.7602380	-3.1307240	1.2709920
C	-1.0207090	-1.8557250	0.7424690
C	-2.2817590	-1.6041190	0.1614570
C	-1.7216040	-4.1328980	1.2063860
C	-2.9630770	-3.8900100	0.6220160
C	-3.2361030	-2.6242150	0.1122090
N	-0.0634660	-0.8231460	0.7408810
P	-2.5226520	0.1325340	-0.3509120
C	-3.6857200	0.1965310	-1.8544760
C	-3.5784480	1.6016090	-2.4762710
C	-3.2121570	-0.8635630	-2.8628600
C	-3.3716500	0.8835760	1.1785820
C	-4.5042040	0.0069760	1.7309370
C	-3.9106890	2.2771860	0.8313820
C	3.4420840	-0.9503670	1.9655480
C	2.3446700	-0.7847310	1.1130550
C	1.0373300	-0.9099150	1.6292140
C	3.2561910	-1.1943060	3.3219720
C	1.9607860	-1.2645950	3.8391970
C	0.8598130	-1.1260720	3.0032740
P	2.4110840	-0.3094330	-0.6490150
C	3.8543780	0.8868430	-0.9130340
C	2.6459160	-1.9173090	-1.6252190
C	2.8656930	-1.6026340	-3.1110040
C	3.8033210	-2.7634110	-1.0785730
C	3.4885060	1.7395870	-2.1405140
C	3.9391810	1.8028000	0.3132780
V	0.0347090	0.5859490	-0.5492590
C	0.2225120	2.2430340	-0.0220590
C	0.4326200	3.5077570	0.7731730
C	1.4240420	4.4163920	0.0196600
C	0.9408150	3.1998900	2.1887510

C	-0.9042410	4.2668930	0.8775020
C	-0.0954400	0.5404660	-2.5886890
H	0.2070500	-3.3453160	1.7261110
H	-1.4919170	-5.1191050	1.6156350
H	-4.2170300	-2.4322820	-0.3217560
H	-4.0709550	2.3631210	-1.8579930
H	-4.0853480	1.6000830	-3.4542480
H	-2.5429870	1.9249120	-2.6388410
H	-2.1586960	-0.7501180	-3.1386000
H	-3.8054400	-0.7641590	-3.7855210
H	-3.3568990	-1.8853860	-2.4859400
H	-4.9463390	0.5218290	2.5993680
H	-4.1349940	-0.9664860	2.0824620
H	-5.3134720	-0.1684440	1.0128950
H	-4.8285030	2.2275830	0.2301800
H	-3.1773420	2.8872080	0.2878120
H	-4.1634080	2.8113300	1.7611120
H	4.4550100	-0.8877190	1.5649500
H	1.8077200	-1.4354500	4.9067960
H	-0.1511370	-1.2035530	3.4067460
H	2.9091030	-2.5500850	-3.6712240
H	3.8104340	-1.0748780	-3.2987310
H	2.0456110	-1.0075680	-3.5373570
H	4.7861560	-2.3033640	-1.2303030
H	3.8115570	-3.7290260	-1.6096040
H	3.6798540	-2.9827310	-0.0076190
H	3.3494640	1.1385630	-3.0505580
H	4.3026700	2.4539240	-2.3414690
H	2.5709400	2.3196650	-1.9647660
H	4.5223170	2.7007130	0.0553260
H	4.4349830	1.3222090	1.1658630
H	2.9460020	2.1263660	0.6417010

H	1.5873260	5.3422660	0.5923130
H	1.0273510	4.7013640	-0.9674500
H	2.4007870	3.9414500	-0.1308620
H	1.9089880	2.6811090	2.1841470
H	1.0661860	4.1365410	2.7530310
H	0.2302070	2.5690350	2.7420400
H	-0.7280110	5.2554890	1.3288890
H	-1.6228190	3.7351280	1.5131720
H	-1.3596900	4.4272170	-0.1120500
H	0.8561720	0.9705600	-2.9416550
H	-0.1348540	-0.5204410	-2.8792980
H	-0.9089460	1.0853610	-3.0815550
H	0.0407040	2.4315290	-1.1276280
C	-2.3043420	0.9971550	2.2744540
H	-1.9378570	0.0086940	2.5853080
H	-1.4410780	1.5896420	1.9523510
H	-2.7474350	1.4811870	3.1595750
C	5.2258260	0.2362080	-1.1286790
H	5.2857550	-0.3426010	-2.0595270
H	5.9791800	1.0374740	-1.1967500
H	5.5217180	-0.4140300	-0.2935400
C	1.3589950	-2.7389300	-1.4703290
H	1.1972910	-3.0486300	-0.4300690
H	0.4612460	-2.2067460	-1.8139120
H	1.4500690	-3.6573710	-2.0714640
C	-5.1708690	-0.0629980	-1.5586950
H	-5.7172190	-0.0334240	-2.5150810
H	-5.3627140	-1.0467400	-1.1118460
H	-5.6173910	0.7039280	-0.9137230
H	4.1193330	-1.3230870	3.9771630
H	-3.7178160	-4.6763400	0.5710380

6’

Symbol	X	Y	Z
C	-0.5723790	-3.4248840	0.4661360
C	-0.8960600	-2.0659040	0.3120230
C	-2.1816150	-1.7188200	-0.1537300
C	-1.5004780	-4.4123310	0.1610950
C	-2.7709640	-4.0724350	-0.3010470
C	-3.1022400	-2.7297970	-0.4491600
N	0.0166960	-1.0328400	0.5964610
P	-2.4944670	0.0819760	-0.2210580
C	-3.6669510	0.4298770	-1.6808790
C	-3.6418190	1.9397770	-1.9771910
C	-3.1335110	-0.3472740	-2.8961560
C	-3.3685570	0.4144440	1.4367360
C	-4.4656750	-0.6136250	1.7459730
C	-3.9608840	1.8297040	1.4258720
C	3.5007280	-1.3272410	1.8425300
C	2.4099740	-0.9910890	1.0325310
C	1.1038290	-1.3077500	1.4599370
C	3.3066410	-1.9394900	3.0762090
C	2.0094180	-2.2104370	3.5159280
C	0.9155550	-1.8976630	2.7184050
P	2.4779310	-0.0867710	-0.5543290
C	3.8418960	1.2182060	-0.4395240
C	2.8438610	-1.3782050	-1.8886560
C	3.0921470	-0.6829950	-3.2342810
C	4.0268420	-2.2837580	-1.5248090
C	3.5092000	2.2943890	-1.4859330
C	3.7776360	1.8365450	0.9648420

V	0.0466650	0.6322280	-0.3531950
C	0.2059610	2.0375330	0.7307920
C	-0.0055730	3.5340630	0.8569450
C	-0.7931440	4.0661970	-0.3461380
C	1.3376560	4.2796870	0.9478580
C	-0.7800900	3.8136240	2.1586930
C	-0.1000100	1.1469440	-2.2905220
H	0.4164550	-3.7120790	0.8245790
H	-1.2237620	-5.4617010	0.2833930
H	-4.1037170	-2.4710410	-0.7902370
H	-4.0897730	2.5297250	-1.1672100
H	-4.2353450	2.1325740	-2.8849230
H	-2.6332960	2.3281140	-2.1574110
H	-2.0639120	-0.1854740	-3.0713780
H	-3.6706510	-0.0109930	-3.7970630
H	-3.2981040	-1.4287490	-2.7978120
H	-4.9422550	-0.3358850	2.7000280
H	-4.0542470	-1.6252290	1.8693430
H	-5.2563910	-0.6516490	0.9886310
H	-4.8803750	1.8906030	0.8279530
H	-3.2516050	2.5742080	1.0379000
H	-4.2225750	2.1240220	2.4545450
H	4.5161130	-1.1114600	1.5077870
H	1.8489590	-2.6755860	4.4907920
H	-0.0962270	-2.1293230	3.0553990
H	3.1481320	-1.4489500	-4.0239770
H	4.0401120	-0.1283570	-3.2570010
H	2.2791040	0.0070830	-3.5042520
H	4.9818240	-1.7477840	-1.4734510
H	4.1287170	-3.0580910	-2.3022490
H	3.8648090	-2.8029630	-0.5686270
H	3.5283650	1.9047880	-2.5129970

H	4.2567210	3.1014510	-1.4275410
H	2.5211990	2.7394740	-1.3074460
H	4.3176100	2.7962320	0.9612500
H	4.2469200	1.1946570	1.7214580
H	2.7473300	2.0359000	1.2834830
H	-0.9615880	5.1496740	-0.2490520
H	-1.7763570	3.5795220	-0.4242250
H	-0.2504460	3.8944310	-1.2881270
H	1.9561660	4.1296860	0.0534920
H	1.1419740	5.3590990	1.0422020
H	1.9183420	3.9711710	1.8290390
H	-0.9444590	4.8966830	2.2707770
H	-0.2172150	3.4670960	3.0392940
H	-1.7623420	3.3251570	2.1701030
H	-0.9606540	1.7387950	-2.6214700
H	0.8030990	1.7819810	-2.3657730
H	0.0287120	0.2765440	-2.9523500
H	0.4916880	1.5245560	1.6752930
C	-2.3159760	0.3052650	2.5482270
H	-1.8517840	-0.6909050	2.5719360
H	-1.5212900	1.0537530	2.4499650
H	-2.8099190	0.4633320	3.5201270
C	-5.1370490	0.0371300	-1.4677890
H	-5.6237810	0.6279820	-0.6814240
H	-5.6809060	0.2412240	-2.4041400
H	-5.2817140	-1.0267170	-1.2423330
C	5.2669520	0.7087890	-0.6861300
H	5.4231220	0.3683730	-1.7179890
H	5.9664910	1.5419810	-0.5115640
H	5.5555300	-0.1041570	-0.0055160
C	1.5932210	-2.2600800	-2.0053830
H	1.4055320	-2.8224420	-1.0820330

H	0.6874460	-1.6871870	-2.2490500
H	1.7475080	-2.9959880	-2.8103580
H	-3.5020690	-4.8468110	-0.5383090
H	4.1653960	-2.2001900	3.6971410

6’-7’-TS

Symbol	X	Y	Z
C	-0.6015180	-3.5923930	0.2074110
C	-0.8776470	-2.2075750	0.1719680
C	-2.1719000	-1.8101310	-0.2315960
C	-1.5734010	-4.5251430	-0.1258370
C	-2.8567420	-4.1236090	-0.5024720
C	-3.1449930	-2.7651820	-0.5465100
N	0.0705460	-1.2286230	0.4648350
P	-2.4089040	-0.0125690	-0.1844020
C	-3.5557140	0.4944860	-1.6152100
C	-3.3818750	2.0014840	-1.8570900
C	-3.1253050	-0.2667160	-2.8796900
C	-3.2177800	0.2825970	1.5102400
C	-4.3420020	-0.7213570	1.7988520
C	-3.7530390	1.7134650	1.6078280
C	3.5278760	-1.4801920	1.7793150
C	2.4331120	-1.1181460	0.9874400
C	1.1365420	-1.5724290	1.3084640
C	3.3502090	-2.2524970	2.9222640
C	2.0616750	-2.6611610	3.2742280
C	0.9688410	-2.3282470	2.4840290
P	2.4255950	-0.0443810	-0.4768380
C	3.7290370	1.3092370	-0.2546770

C	2.8285430	-1.1753580	-1.9475910
C	3.0771000	-0.3512760	-3.2156530
C	4.0366490	-2.0727240	-1.6442240
C	3.3881330	2.4498570	-1.2259860
C	3.6260400	1.8190400	1.1919510
V	0.0304360	0.6621550	-0.3810470
C	-0.0619340	2.2464690	0.4533480
C	-0.2259180	3.6912240	0.8681830
C	-1.3323900	4.3517480	0.0344750
C	1.0784550	4.4773100	0.6369710
C	-0.5688300	3.7368480	2.3666410
C	0.1578910	1.3284140	-2.0446130
H	0.3911140	-3.9396700	0.4960460
H	-1.3216960	-5.5878810	-0.0951390
H	-4.1492390	-2.4465930	-0.8274470
H	-3.7114990	2.6013630	-0.9984960
H	-3.9960070	2.3017780	-2.7208360
H	-2.3395060	2.2669790	-2.0797270
H	-2.0666370	-0.1160670	-3.1344810
H	-3.7125730	0.1074360	-3.7330950
H	-3.3012100	-1.3475850	-2.8022340
H	-4.7696060	-0.4896600	2.7876530
H	-3.9687160	-1.7540010	1.8367720
H	-5.1616290	-0.6752160	1.0717460
H	-4.6395400	1.8807740	0.9814810
H	-2.9901720	2.4495380	1.3274910
H	-4.0469150	1.9204130	2.6491880
H	4.5330920	-1.1534850	1.5090270
H	1.9050500	-3.2474850	4.1825680
H	-0.0299970	-2.6644230	2.7679350
H	3.1730640	-1.0380040	-4.0714000
H	4.0046810	0.2348320	-3.1677000

H	2.2424350	0.3313860	-3.4351420
H	4.9683230	-1.5129280	-1.5011920
H	4.1924600	-2.7520900	-2.4975290
H	3.8648120	-2.6977280	-0.7561360
H	3.4848700	2.1466390	-2.2770020
H	4.0823540	3.2885960	-1.0578410
H	2.3692850	2.8231580	-1.0739730
H	4.1964010	2.7568650	1.2826790
H	4.0420480	1.1056310	1.9149250
H	2.5917760	2.0310450	1.4941760
H	-1.4267180	5.4114850	0.3142760
H	-2.3081370	3.8750920	0.1885840
H	-1.1028200	4.3149150	-1.0425940
H	1.3400230	4.5268220	-0.4301440
H	0.9452860	5.5103690	0.9923940
H	1.9273710	4.0437760	1.1817510
H	-0.6848440	4.7822320	2.6903500
H	0.2336160	3.2866790	2.9719590
H	-1.5036580	3.2075490	2.5938460
H	0.0196620	2.2068270	-0.9498840
H	0.3446540	2.0036530	-2.8832660
H	-0.4207520	0.4105960	-2.3216790
H	0.5828930	1.6181260	1.1493330
C	5.1772050	0.8687110	-0.5095410
H	5.3603740	0.6113740	-1.5608560
H	5.8428740	1.7113530	-0.2629290
H	5.4865980	0.0187320	0.1129010
C	1.6186480	-2.0902840	-2.1847350
H	1.4086670	-2.7325860	-1.3205780
H	0.7039650	-1.5323080	-2.4288290
H	1.8403930	-2.7492790	-3.0390570
C	-2.1212290	0.0823600	2.5674570

H	-1.6857300	-0.9257310	2.5248210
H	-1.3070260	0.8125860	2.4648070
H	-2.5646190	0.2127090	3.5674720
C	-5.0462660	0.2128230	-1.3734370
H	-5.4573320	0.7897530	-0.5355840
H	-5.6055520	0.5107360	-2.2747340
H	-5.2620000	-0.8492100	-1.1998760
H	4.2075420	-2.5255720	3.5396210
H	-3.6206110	-4.8591570	-0.7587950

6’-7”-TS

Symbol	X	Y	Z
C	-0.5501550	-3.5901250	0.1767910
C	-0.8458600	-2.2094580	0.1527950
C	-2.1389330	-1.8194990	-0.2621900
C	-1.5017920	-4.5319290	-0.1869840
C	-2.7823420	-4.1407850	-0.5821140
C	-3.0895910	-2.7859340	-0.6091390
N	0.0850340	-1.2217820	0.4656540
P	-2.4094040	-0.0218210	-0.1753210
C	-3.5920420	0.4798010	-1.5802780
C	-3.5296160	2.0029230	-1.7658670
C	-3.0984320	-0.1974200	-2.8687820
C	-3.2167350	0.2123930	1.5305690
C	-4.2975950	-0.8406530	1.8105130
C	-3.8105620	1.6191260	1.6484830
C	3.5461230	-1.4437450	1.7840990
C	2.4515300	-1.0874310	0.9885970
C	1.1570740	-1.5538020	1.3054290

C	3.3725930	-2.2233230	2.9225120
C	2.0871940	-2.6467320	3.2677800
C	0.9942410	-2.3183460	2.4762130
P	2.4579190	-0.0193690	-0.4798080
C	3.7432560	1.3512370	-0.2497680
C	2.8906730	-1.1489590	-1.9412180
C	3.0969360	-0.3118380	-3.2089410
C	4.1308850	-2.0058720	-1.6530810
C	3.4108100	2.4816740	-1.2370190
C	3.6001330	1.8693630	1.1900620
V	0.0046340	0.6581010	-0.3795610
C	-0.0725790	2.2360580	0.4587650
C	-0.2753430	3.6831080	0.8445680
C	-1.3749910	4.3101820	-0.0207640
C	1.0220680	4.4862830	0.6344190
C	-0.6566220	3.7448080	2.3327750
C	-0.1770720	1.3780430	-2.0028080
H	0.4423390	-3.9262400	0.4789530
H	-1.2362950	-5.5915180	-0.1664660
H	-4.0937900	-2.4815540	-0.9017840
H	-3.9457010	2.5457880	-0.9071410
H	-4.1304790	2.2794010	-2.6467450
H	-2.5059770	2.3566530	-1.9340710
H	-2.0498140	0.0420520	-3.0917570
H	-3.7031200	0.1662510	-3.7147050
H	-3.1978830	-1.2906120	-2.8347350
H	-4.7395740	-0.6336110	2.7985990
H	-3.8799770	-1.8564210	1.8432770
H	-5.1153080	-0.8278950	1.0799850
H	-4.7248000	1.7425590	1.0524820
H	-3.0930320	2.3910960	1.3416950
H	-4.0789600	1.8136350	2.6991270

H	4.5491930	-1.1062050	1.5194220
H	1.9329840	-3.2405770	4.1715990
H	-0.0024140	-2.6655640	2.7543990
H	3.1825350	-0.9867460	-4.0751380
H	4.0188810	0.2844790	-3.1724100
H	2.2532850	0.3687940	-3.4037270
H	5.0463890	-1.4163620	-1.5297320
H	4.2940740	-2.6874170	-2.5033940
H	3.9930730	-2.6289650	-0.7575400
H	3.5549320	2.1798960	-2.2827690
H	4.0792070	3.3362290	-1.0459370
H	2.3802670	2.8391660	-1.1287610
H	4.1582990	2.8136190	1.2897190
H	4.0046100	1.1642510	1.9277290
H	2.5554210	2.0713140	1.4612510
H	-1.4880330	5.3748500	0.2329760
H	-2.3455110	3.8223030	0.1316010
H	-1.1283520	4.2495830	-1.0919090
H	1.3070400	4.5267200	-0.4272140
H	0.8652060	5.5212760	0.9738410
H	1.8648010	4.0719990	1.2033450
H	-0.8234350	4.7908300	2.6312140
H	0.1468710	3.3405910	2.9683270
H	-1.5759740	3.1841200	2.5476240
H	0.3776730	2.1645420	-0.9265220
H	-0.3531690	2.0948250	-2.8077210
H	0.0889160	0.3505010	-2.3572880
H	0.4637190	1.6096540	1.2336090
C	-2.1108680	0.0433430	2.5829300
H	-1.6362830	-0.9459790	2.5276530
H	-1.3260590	0.8060110	2.4887830
H	-2.5562350	0.1443840	3.5854750

C	-5.0637250	0.0972320	-1.3589630
H	-5.5109200	0.6122860	-0.4992140
H	-5.6354300	0.4048850	-2.2491150
H	-5.2238570	-0.9809890	-1.2365240
C	5.2019450	0.9319270	-0.4763970
H	5.4067790	0.6823590	-1.5260310
H	5.8514930	1.7826230	-0.2146060
H	5.5116200	0.0833520	0.1477120
C	1.7115450	-2.1088410	-2.1595370
H	1.5279270	-2.7435520	-1.2835600
H	0.7695090	-1.5991840	-2.4088290
H	1.9515960	-2.7732870	-3.0044880
H	-3.5314580	-4.8817810	-0.8649750
H	4.2307350	-2.4912560	3.5409610

7’

Symbol	X	Y	Z
C	-0.5250250	-3.5596120	0.0119040
C	-0.8449670	-2.1876640	0.0774160
C	-2.1315720	-1.7947630	-0.3511770
C	-1.4456840	-4.4871690	-0.4580220
C	-2.7167790	-4.0908550	-0.8752150
C	-3.0507670	-2.7432400	-0.8094240
N	0.0668980	-1.2074180	0.4904790
P	-2.4498060	-0.0249150	-0.1226330
C	-3.5976980	0.6062890	-1.4922990
C	-3.4366260	2.1330800	-1.5500300
C	-3.1293250	-0.0004650	-2.8228040
C	-3.2890810	0.0238050	1.5893790
C	-4.3993220	-1.0294710	1.7135480

C	-3.8524710	1.4163360	1.8800220
C	3.5515900	-1.6005400	1.7063360
C	2.4486260	-1.1695430	0.9617210
C	1.1534160	-1.6150000	1.2871230
C	3.3811710	-2.4405490	2.8015960
C	2.0933960	-2.8507450	3.1527140
C	0.9917330	-2.4471570	2.4082930
P	2.4358510	-0.0167430	-0.4392040
C	3.7245840	1.3402270	-0.1123160
C	2.8706350	-1.0495970	-1.9693570
C	3.1147840	-0.1430260	-3.1817370
C	4.0915320	-1.9464100	-1.7213150
C	3.3623660	2.5532150	-0.9834910
C	3.6481810	1.7412290	1.3711610
V	-0.0079160	0.6545180	-0.2938240
C	-0.0529130	2.2705210	0.8063870
C	-0.2274320	3.7965660	0.7456970
C	-0.1442150	4.3581820	-0.6778660
C	0.8945350	4.4230540	1.5967110
C	-1.5787690	4.1952930	1.3465050
C	0.0007960	1.0437300	-1.9842290
H	0.4610680	-3.9036510	0.3242320
H	-1.1597180	-5.5404890	-0.5049710
H	-4.0478700	-2.4270070	-1.1176980
H	-3.7349920	2.6224690	-0.6120990
H	-4.0778390	2.5367300	-2.3494190
H	-2.4015160	2.4199430	-1.7761460
H	-2.0496870	0.1220900	-2.9805000
H	-3.6471060	0.5120110	-3.6490660
H	-3.3606850	-1.0713810	-2.8956320
H	-4.8335150	-0.9529090	2.7234280
H	-4.0117130	-2.0513810	1.6024130

H	-5.2180640	-0.8874120	0.9986470
H	-4.6879580	1.6873710	1.2201350
H	-3.0821480	2.1911590	1.8006910
H	-4.2355560	1.4380480	2.9126360
H	4.5573980	-1.2770230	1.4343690
H	1.9444620	-3.4962980	4.0210880
H	-0.0070250	-2.7904010	2.6836560
H	3.2177300	-0.7717620	-4.0803050
H	4.0388260	0.4440950	-3.0920520
H	2.2804130	0.5502080	-3.3610360
H	5.0170760	-1.3857020	-1.5476940
H	4.2523640	-2.5699980	-2.6152870
H	3.9307590	-2.6285280	-0.8742880
H	3.3716220	2.3237870	-2.0575630
H	4.0969550	3.3554860	-0.8101630
H	2.3735500	2.9578530	-0.7354960
H	4.2408450	2.6581030	1.5177860
H	4.0630880	0.9693470	2.0318870
H	2.6284990	1.9575970	1.7192900
H	-0.1873090	5.4573650	-0.6513830
H	-0.9869160	4.0193540	-1.2991640
H	0.7926120	4.0868940	-1.1865480
H	1.8887810	4.2200790	1.1703890
H	0.7726650	5.5160200	1.6490560
H	0.8807280	4.0351780	2.6272480
H	-1.6698350	5.2917240	1.3762040
H	-1.6904710	3.8259320	2.3774980
H	-2.4122490	3.8119120	0.7419090
H	1.0395190	2.0191010	0.5915540
H	-0.2029790	2.0960970	-1.6459520
H	0.0385250	0.9082880	-3.0685120
H	-0.2301400	1.8788520	1.8252770

C	-2.2190970	-0.3061560	2.6403560
H	-1.7817650	-1.3016110	2.4873180
H	-1.4024470	0.4273260	2.6614850
H	-2.6925670	-0.3013410	3.6349470
C	-5.0857200	0.2779570	-1.3067570
H	-5.5220000	0.7626770	-0.4240240
H	-5.6335420	0.6559500	-2.1847770
H	-5.2858330	-0.7999420	-1.2445600
C	5.1728910	0.9334340	-0.4210970
H	5.3434560	0.7535660	-1.4903080
H	5.8343080	1.7627600	-0.1235880
H	5.4981560	0.0452930	0.1364660
C	1.6730090	-1.9676040	-2.2550360
H	1.4876430	-2.6645000	-1.4276000
H	0.7438950	-1.4156380	-2.4503410
H	1.8992530	-2.5728190	-3.1470390
H	4.2450710	-2.7666280	3.3830210
H	-3.4374030	-4.8214700	-1.2454620

7”

Symbol	X	Y	Z
C	-0.7471430	-3.5477490	0.0267450
C	-1.0049860	-2.1658150	0.0490980
C	-2.3159980	-1.7257190	-0.2206010
C	-1.7663380	-4.4550960	-0.2328050
C	-3.0691970	-4.0163520	-0.4734730
C	-3.3362060	-2.6522200	-0.4619360
N	0.0058680	-1.2158410	0.3098750
P	-2.4941540	0.0773960	-0.1591770
C	-3.7099260	0.6582950	-1.4939350
C	-3.4378430	2.1503240	-1.7502190

C	-3.4362920	-0.1144550	-2.7917030
C	-3.1328120	0.4345240	1.5900200
C	-4.2902930	-0.4811810	2.0045480
C	-3.5464790	1.9043930	1.7176000
C	3.3350790	-1.4576260	1.9306290
C	2.3199060	-1.1128200	1.0348940
C	1.0024370	-1.5608470	1.2525050
C	3.0545110	-2.2182660	3.0624650
C	1.7455750	-2.6401530	3.2935500
C	0.7281170	-2.3184800	2.4017700
P	2.4582180	-0.1066890	-0.4730880
C	3.7542920	1.2393560	-0.1990350
C	3.0219910	-1.3547310	-1.7964160
C	3.1954930	-0.6274470	-3.1341670
C	4.3203360	-2.0809110	-1.4116220
C	3.5247730	2.3189250	-1.2698970
C	3.4869220	1.8044700	1.2035210
V	-0.0239300	0.5478820	-0.5234220
C	0.0867460	2.4633150	-0.1828550
C	0.1512830	3.7622820	0.6311880
C	-1.0334840	4.6445040	0.1846670
C	1.4346080	4.5534930	0.3474280
C	0.0378020	3.4819960	2.1346120
C	-0.1849090	0.3471940	-2.2891310
H	0.2607650	-3.9130150	0.2245790
H	-1.5380390	-5.5230240	-0.2472860
H	-4.3550610	-2.3057270	-0.6401420
H	-3.6072290	2.7738050	-0.8618950
H	-4.1198740	2.5115580	-2.5360830
H	-2.4097070	2.3185570	-2.1020940
H	-2.4031750	0.0124780	-3.1396460
H	-4.0968680	0.2807430	-3.5794150

H	-3.6383200	-1.1898460	-2.6964410
H	-4.5648920	-0.2502340	3.0465260
H	-4.0056830	-1.5424240	1.9695260
H	-5.1897990	-0.3421270	1.3925530
H	-4.4593490	2.1432700	1.1562800
H	-2.7510470	2.5852860	1.3862480
H	-3.7472110	2.1315300	2.7766630
H	4.3586430	-1.1247850	1.7521670
H	1.5098980	-3.2283250	4.1831040
H	-0.2869010	-2.6699940	2.5919320
H	3.3796910	-1.3666970	-3.9298200
H	4.0557750	0.0571600	-3.1243630
H	2.2968810	-0.0562300	-3.4144890
H	5.2071920	-1.4410410	-1.4450330
H	4.4857780	-2.8968360	-2.1333570
H	4.2548430	-2.5423850	-0.4153190
H	3.7943460	1.9625850	-2.2733800
H	4.1606560	3.1915870	-1.0516070
H	2.4856110	2.6626500	-1.3032900
H	4.0250680	2.7578990	1.3214760
H	3.8406560	1.1280150	1.9923710
H	2.4185690	1.9905710	1.3768470
H	-1.0224970	5.6012240	0.7293890
H	-2.0048600	4.1661200	0.3791240
H	-0.9791870	4.8707000	-0.8917910
H	1.5483020	4.7677520	-0.7266930
H	1.3923040	5.5190560	0.8740120
H	2.3335310	4.0316210	0.6907880
H	0.0765360	4.4240680	2.7027500
H	0.8583630	2.8428510	2.4933180
H	-0.9102870	2.9865250	2.3878010
H	0.3727000	2.6409910	-1.2429500

H	-0.0946580	1.1464030	-3.0392040
H	-0.3442670	-0.6516210	-2.7223080
H	-1.0094400	2.1515130	-0.2441110
C	-5.1867880	0.4686130	-1.1193290
H	-5.4877940	1.0648610	-0.2487310
H	-5.8064370	0.8015290	-1.9670510
H	-5.4444230	-0.5814940	-0.9262970
C	-1.9485420	0.1674690	2.5322690
H	-1.6588770	-0.8914470	2.5338100
H	-1.0594910	0.7628380	2.2761610
H	-2.2393000	0.4314320	3.5615120
C	5.2221760	0.8026730	-0.2806730
H	5.8508570	1.6546200	0.0249630
H	5.4664690	-0.0379090	0.3821590
H	5.5169780	0.5379970	-1.3046490
C	1.9424290	-2.4380840	-1.9275140
H	1.8583370	-3.0292120	-1.0055110
H	0.9518430	-2.0413660	-2.1710100
H	2.2362640	-3.1291440	-2.7330880
H	-3.8694300	-4.7317290	-0.6691650
H	3.8521660	-2.4774240	3.7605210

V^V (L_2)

1

Symbol	X	Y	Z
C	0.7102750	3.6855300	0.2520090
C	0.9797490	2.3272000	0.4432340
C	2.2780020	1.8499110	0.1787810
C	1.7111640	4.5463020	-0.1908160

C	3.0114880	4.0901680	-0.4382760
C	3.2774640	2.7298810	-0.2418630
P	-0.1714670	1.0981200	1.1587640
C	3.9829810	-0.4027160	-0.6195400
C	4.4381230	-1.8467780	-0.4144470
C	3.7747140	-0.1095890	-2.1061790
C	3.0425840	-0.0891110	2.2147920
C	4.4636300	0.4006560	2.4766550
C	2.7702170	-1.4811490	2.7868880
C	4.0800820	5.0376310	-0.9119970
C	-3.9472700	1.5056670	-0.2959780
C	-2.6065940	1.1564850	-0.1192250
C	-1.8594530	1.7402290	0.9134640
C	-4.5632580	2.4407620	0.5442800
C	-3.7992350	3.0271130	1.5625060
C	-2.4646660	2.6809830	1.7524720
C	-6.0099460	2.8125390	0.3677140
C	-2.9013750	-0.9390620	-2.1104580
C	-0.8754850	1.2542820	-2.3801110
C	0.3859730	0.7307880	-3.0590530
C	-1.8865070	1.8442810	-3.3599050
C	-2.4024860	-1.6955900	-3.3414710
C	-3.6904190	-1.8591260	-1.1810680
V	0.1294940	-1.0332350	0.1667890
C	-0.9469700	-1.6435570	1.3779770
C	-1.6710610	-1.9326570	2.6610080
C	-1.4486100	-3.4153620	3.0114450
C	-3.1741340	-1.6645320	2.4673070
C	-1.1363290	-1.0497790	3.7954270
C	0.5227870	-2.3091030	-1.4321890
C	0.6464350	-3.8294180	-1.1589100
C	-0.7218880	-4.5186530	-1.1884140

C	1.5232570	-4.4415340	-2.2655490
C	1.3110200	-4.1088690	0.1995000
H	-0.2917300	4.0775920	0.4435060
H	1.4771460	5.6030350	-0.3458690
H	4.2865800	2.3619740	-0.4443740
H	4.7800490	0.2605680	-0.2442260
H	4.6453500	-2.0887860	0.6366300
H	5.3688620	-2.0156170	-0.9770780
H	3.6949950	-2.5610260	-0.7979770
H	3.0480910	-0.8019900	-2.5568010
H	4.7288810	-0.2509290	-2.6366330
H	3.4366180	0.9174780	-2.2988330
H	2.3379850	0.6077300	2.6997160
H	4.6450340	0.4463450	3.5614210
H	4.6315110	1.4109500	2.0736940
H	5.2188520	-0.2761870	2.0484620
H	3.4458330	-2.2463760	2.3775830
H	1.7343140	-1.8005050	2.5926470
H	2.9136940	-1.4691790	3.8782430
H	4.2233950	5.8623920	-0.1971620
H	3.8079060	5.4904910	-1.8780690
H	5.0463040	4.5300720	-1.0389480
H	-4.5427110	1.0504810	-1.0908170
H	-4.2661200	3.7565590	2.2295490
H	-1.8967690	3.1300530	2.5712630
H	-6.4790880	2.2481630	-0.4497890
H	-6.1170310	3.8852560	0.1442210
H	-6.5816010	2.6152040	1.2875220
H	-3.5796540	-0.1487840	-2.4728820
H	-0.5611080	2.0608640	-1.6988620
H	0.8715410	1.5441850	-3.6198990
H	0.1854410	-0.0883280	-3.7654110

H	1.1087700	0.3644190	-2.3186820
H	-2.2256770	1.1113430	-4.1068630
H	-1.4185030	2.6758300	-3.9089640
H	-2.7700560	2.2507760	-2.8452400
H	-1.7781240	-1.0811270	-4.0036010
H	-3.2702530	-2.0320840	-3.9293950
H	-1.8323320	-2.5910870	-3.0652560
H	-4.5362430	-2.3023530	-1.7287580
H	-4.0977750	-1.3294190	-0.3091960
H	-3.0658480	-2.6898000	-0.8199270
H	-2.0071720	-3.6741690	3.9243250
H	-0.3847480	-3.6287100	3.1970250
H	-1.7970870	-4.0804920	2.2057800
H	-3.6110430	-2.3469670	1.7237980
H	-3.7058460	-1.8213860	3.4187390
H	-3.3561420	-0.6287500	2.1420610
H	-1.6376360	-1.3156900	4.7387380
H	-1.3221250	0.0158520	3.5996480
H	-0.0533360	-1.1820920	3.9361770
H	-1.2086140	-2.3189540	0.4916270
H	0.0212330	-2.1454560	-2.3946840
H	1.5466330	-1.9075010	-1.5849190
H	-0.6203960	-5.5961050	-0.9839290
H	-1.2079560	-4.4158510	-2.1708040
H	-1.4079760	-4.1105080	-0.4292410
H	1.6206740	-5.5325260	-2.1423440
H	2.5387070	-4.0133210	-2.2523260
H	1.0944870	-4.2529620	-3.2624590
H	0.6713950	-3.7957270	1.0391690
H	2.2768940	-3.5895610	0.2956950
H	1.5079990	-5.1848250	0.3296780
P	2.5125210	0.0482090	0.4319720

P	-1.6136150	0.0309360	-1.1769300

1-2-TS

Symbol	X	Y	Z
C	0.0492250	3.8370450	0.2423440
C	0.5355750	2.5443580	0.4424940
C	1.8826830	2.2680950	0.1177490
C	0.8800150	4.8360320	-0.2673770
C	2.2197370	4.5844260	-0.5715350
C	2.7055670	3.2846080	-0.3645860
P	-0.3653620	1.1573860	1.2264890
C	3.9323480	0.3261760	-0.6600280
C	4.6982300	-0.9648330	-0.3665350
C	3.6194280	0.4719500	-2.1513530
C	2.9823080	0.5352870	2.1725460
C	4.2570480	1.3411750	2.4058360
C	3.0377090	-0.8722680	2.7643950
C	3.1301880	5.6646020	-1.0912300
C	-4.2731180	0.9276190	-0.0444900
C	-2.8885090	0.7807050	0.0629020
C	-2.1608500	1.4867180	1.0349950
C	-4.9679730	1.7907450	0.8100310
C	-4.2355510	2.5004020	1.7713730
C	-2.8571610	2.3471150	1.8918780
C	-6.4585930	1.9623810	0.7048500
C	-2.9158750	-1.3635860	-1.9953410
C	-1.3133540	1.1539310	-2.2892360
C	0.0161060	0.8032130	-2.9556350
C	-2.4041450	1.5826010	-3.2655230

C	-2.2062140	-1.9759260	-3.2039530
C	-3.4479170	-2.4432630	-1.0563560
V	0.2228880	-0.8599010	0.0348540
C	-0.4105880	-1.8518380	1.2204380
C	-0.8875890	-2.5633210	2.4501180
C	0.1039320	-3.6647250	2.8599410
C	-2.2727930	-3.1797780	2.2019410
C	-0.9888860	-1.5185210	3.5763370
C	1.2427110	-2.3460270	-1.0504590
C	1.3853760	-3.8758970	-1.1808760
C	0.0466620	-4.5845280	-0.9440710
C	1.8712870	-4.1894740	-2.6044140
C	2.4275640	-4.3669590	-0.1682150
H	-0.9902620	4.0733260	0.4839650
H	0.4728270	5.8380850	-0.4259880
H	3.7504430	3.0793560	-0.6129720
H	4.5809480	1.1670700	-0.3648070
H	5.1070410	-0.9870270	0.6523350
H	5.5491140	-1.0496660	-1.0598230
H	4.0757210	-1.8614450	-0.4987820
H	3.0333340	-0.3761000	-2.5372060
H	4.5606980	0.4985120	-2.7216040
H	3.0702450	1.3961090	-2.3792620
H	2.1475750	1.0627700	2.6644820
H	4.4354840	1.4444300	3.4873220
H	4.1830890	2.3568760	1.9890810
H	5.1444480	0.8516300	1.9762960
H	3.8350330	-1.4879180	2.3226270
H	2.0824490	-1.3966930	2.6172440
H	3.2265230	-0.8156100	3.8476710
H	2.5657370	6.5590150	-1.3893030
H	3.7064040	5.3209590	-1.9635820

H	3.8562760	5.9716720	-0.3213600
H	-4.8325550	0.3751320	-0.8041080
H	-4.7606870	3.1761080	2.4517240
H	-2.3171250	2.8891870	2.6722800
H	-6.7198020	3.0111140	0.4950670
H	-6.9552550	1.6869210	1.6479150
H	-6.8843370	1.3411400	-0.0950190
H	-3.7618350	-0.7615690	-2.3675720
H	-1.1154780	2.0013880	-1.6145350
H	0.3764640	1.6546350	-3.5529670
H	-0.0504780	-0.0681170	-3.6246080
H	0.7934270	0.5955690	-2.2011170
H	-2.6388350	0.8044840	-4.0070100
H	-2.0723270	2.4741540	-3.8197480
H	-3.3330720	1.8521820	-2.7403620
H	-1.9433230	-1.2303160	-3.9660680
H	-2.8703670	-2.7118280	-3.6819800
H	-1.2867750	-2.5089380	-2.9176580
H	-4.1561800	-3.0909890	-1.5952710
H	-3.9728990	-2.0223000	-0.1870130
H	-2.6284000	-3.0749210	-0.6840800
H	-0.2304590	-4.1286000	3.8004290
H	1.1137530	-3.2617440	3.0260310
H	0.1666810	-4.4608000	2.1039490
H	-2.2424730	-3.9396200	1.4069700
H	-2.6311810	-3.6688290	3.1210760
H	-3.0033830	-2.4085520	1.9191650
H	-1.3581460	-2.0020700	4.4940050
H	-1.6850420	-0.7089630	3.3112650
H	-0.0109400	-1.0644030	3.7935550
H	0.6117820	-2.4297390	0.3391270
H	0.4072340	-1.9987690	-1.7289770

H	2.1575290	-1.8679310	-1.4191580
H	0.1695850	-5.6759300	-1.0109050
H	-0.7015460	-4.2898630	-1.6959870
H	-0.3736440	-4.3612630	0.0473540
H	1.9896230	-5.2744880	-2.7500690
H	2.8451170	-3.7161630	-2.8073520
H	1.1556040	-3.8274260	-3.3600910
H	2.1325000	-4.1289600	0.8651610
H	3.4105730	-3.9060860	-0.3503940
H	2.5566220	-5.4579500	-0.2375530
P	2.4106000	0.5239730	0.3965080
P	-1.8370390	-0.1674900	-1.0788090

A

Symbol	X	Y	Z
C	0.8217020	-2.7185270	-1.3470200
C	1.1004520	-1.3856510	-1.0207760
C	2.3588780	-1.0901110	-0.4468310
C	1.7579680	-3.7222480	-1.1183290
C	3.0149020	-3.4381810	-0.5696070
C	3.2944390	-2.1087660	-0.2395020
P	-0.0158800	0.0412170	-1.3992870
C	4.0078400	0.6729790	1.2813390
C	4.4757030	2.1003040	1.5736420
C	3.5489220	-0.0305510	2.5589730
C	3.3263330	1.4234830	-1.5612900
C	4.7311260	0.9405890	-1.9118150
C	3.1873940	2.9449710	-1.5759180
C	4.0328170	-4.5273250	-0.3671430

C	-3.8303600	-1.2353020	-0.4249290
C	-2.5226380	-0.7535770	-0.3392880
C	-1.7039730	-0.6897330	-1.4805710
C	-4.3591300	-1.6694500	-1.6457820
C	-3.5348940	-1.6141620	-2.7767870
C	-2.2330190	-1.1259910	-2.7002580
C	-5.7767650	-2.1604600	-1.7514370
C	-3.0188740	0.2367490	2.4275580
C	-1.0226750	-1.9134540	1.8189160
C	0.2185380	-1.6897160	2.6810750
C	-2.0451310	-2.8485390	2.4548050
C	-2.3896300	0.5414250	3.7882970
C	-3.8217490	1.4253530	1.9072850
V	0.2051550	1.1596490	0.6793630
C	-0.6901160	2.4085850	0.0732390
C	-1.4295600	3.4429230	-0.7178760
C	-1.9040270	4.5579580	0.2297140
C	-2.6310430	2.8024580	-1.4323840
C	-0.4748170	4.0355390	-1.7697260
H	-0.1448710	-2.9777640	-1.7870370
H	1.5094050	-4.7544840	-1.3797920
H	4.2679390	-1.8753190	0.1995470
H	4.8548580	0.1131970	0.8516080
H	4.9721600	2.5628740	0.7103850
H	5.2046700	2.0868310	2.3982190
H	3.6416780	2.7532650	1.8789400
H	2.7105180	0.5061410	3.0341220
H	4.3718220	-0.0495400	3.2897690
H	3.2381720	-1.0699980	2.3833970
H	2.6207680	1.0215450	-2.3083350
H	5.0059280	1.2995690	-2.9155770
H	4.7985760	-0.1578140	-1.9262830

H	5.4855170	1.3277430	-1.2094830
H	3.8982860	3.4392490	-0.8986290
H	2.1715670	3.2546680	-1.2942370
H	3.3831070	3.3241410	-2.5905830
H	3.5903440	-5.4030070	0.1310690
H	4.8818120	-4.1834840	0.2401690
H	4.4323560	-4.8726030	-1.3340870
H	-4.4605490	-1.2775240	0.4677000
H	-3.9266760	-1.9475480	-3.7413560
H	-1.6220650	-1.0703730	-3.6047560
H	-6.2252800	-2.3268400	-0.7621500
H	-5.8308960	-3.1034600	-2.3152720
H	-6.4029600	-1.4271260	-2.2839860
H	-3.6930840	-0.6287540	2.5389050
H	-0.6805750	-2.3671520	0.8750090
H	0.7329600	-2.6438720	2.8725990
H	-0.0139060	-1.2343880	3.6559450
H	0.9435640	-1.0405090	2.1629300
H	-2.4097060	-2.4693410	3.4217720
H	-1.5848690	-3.8313030	2.6403990
H	-2.9117930	-3.0128780	1.7968860
H	-1.8945570	-0.3334750	4.2324600
H	-3.1689630	0.8675010	4.4934470
H	-1.6541000	1.3602600	3.7187650
H	-4.5931120	1.7011930	2.6424320
H	-4.3301490	1.2046160	0.9580770
H	-3.1718530	2.2991260	1.7541960
H	-2.4010890	5.3538890	-0.3468500
H	-1.0588090	5.0103240	0.7706210
H	-2.6247840	4.1830950	0.9711740
H	-3.3228330	2.3290320	-0.7226580
H	-3.1882180	3.5747890	-1.9857330

H	-2.3051530	2.0387380	-2.1536660
H	-1.0142660	4.7618710	-2.3980400
H	-0.0705220	3.2496630	-2.4252490
H	0.3659480	4.5624710	-1.2941700
P	-1.7158180	-0.2857690	1.2219730
P	2.6632600	0.6666460	0.0024200

5-6-TS

Symbol	X	Y	Z
C	1.8312380	2.9560210	-1.3670780
C	1.5833260	1.6298780	-0.9770510
C	2.6486970	0.9172030	-0.3959730
C	3.0453070	3.5737220	-1.0919450
C	4.0850780	2.8866640	-0.4511570
C	3.8738630	1.5432670	-0.1363900
P	0.0150400	0.7632810	-1.3938630
C	3.0845290	-1.6773400	-1.6920560
C	2.7624870	-3.1730940	-1.6822660
C	2.6327080	-1.0122610	-2.9902000
C	3.4747300	-1.4338180	1.2464910
C	4.9161460	-1.7670790	0.8551840
C	2.8535700	-2.5858260	2.0402610
C	5.3862070	3.5733490	-0.1387380
C	-3.7152710	1.7199450	-0.0074150
C	-2.5543810	1.0538890	-0.4047700
C	-1.3601030	1.7590450	-0.6641720
C	-3.7230580	3.1019990	0.2012390
C	-2.5235700	3.7907110	-0.0091130
C	-1.3722560	3.1419340	-0.4480260

C	-4.9657000	3.8263680	0.6430270
C	-3.8470070	-1.4959660	0.3551430
C	-2.6817100	-1.0943140	-2.3728160
C	-2.0172980	-2.3995790	-2.8131280
C	-4.1345790	-0.9594300	-2.8203000
C	-4.0013720	-3.0007050	0.1249070
C	-3.7940070	-1.1868090	1.8486240
V	-0.0570560	-1.2401570	0.1143720
C	-0.0865850	-0.6203440	1.6555210
C	-0.0975610	0.0498970	2.9910620
C	-0.5131870	-0.9488430	4.0822010
C	-1.0766140	1.2353450	2.9317170
C	1.3147510	0.5844590	3.2897680
H	1.0712290	3.5123800	-1.9184360
H	3.1958830	4.6134880	-1.3945610
H	4.6890650	0.9840520	0.3275030
H	4.1752020	-1.5494430	-1.6112650
H	3.0846920	-3.6736020	-0.7570930
H	3.2735010	-3.6738540	-2.5186370
H	1.6824010	-3.3545290	-1.8118640
H	1.5436220	-1.0748770	-3.1371770
H	3.1130250	-1.5192470	-3.8413380
H	2.9152170	0.0491220	-3.0330560
H	3.4855410	-0.5434100	1.8947410
H	5.5127250	-1.9135750	1.7684300
H	5.4042800	-0.9777560	0.2672530
H	4.9750770	-2.7014670	0.2771970
H	2.8334520	-3.5206270	1.4582590
H	1.8287230	-2.3602210	2.3644170
H	3.4552610	-2.7794220	2.9415960
H	6.1229250	2.8750640	0.2824250
H	5.2373670	4.3858190	0.5896440

H	5.8240070	4.0250200	-1.0420360
H	-4.6357790	1.1594140	0.1758840
H	-2.4879460	4.8693020	0.1676390
H	-0.4708710	3.7343390	-0.6007320
H	-4.7851730	4.4037130	1.5627490
H	-5.7932050	3.1297730	0.8380820
H	-5.3020280	4.5390000	-0.1263530
H	-4.7301990	-0.9977300	-0.0786150
H	-2.1094970	-0.2728700	-2.8357270
H	-2.1129370	-2.5217340	-3.9029100
H	-2.4629670	-3.2887720	-2.3425430
H	-0.9377850	-2.3976070	-2.5881570
H	-4.7711860	-1.7755050	-2.4468760
H	-4.1870370	-0.9824020	-3.9197060
H	-4.5696720	-0.0027830	-2.4930480
H	-4.0808930	-3.2719600	-0.9360350
H	-4.9248440	-3.3440300	0.6155600
H	-3.1712100	-3.5685190	0.5686200
H	-4.6858230	-1.5998330	2.3442660
H	-3.7676120	-0.1093640	2.0582010
H	-2.9112560	-1.6494680	2.3144450
H	-0.4890380	-0.4565190	5.0664310
H	0.1730630	-1.8092380	4.1242220
H	-1.5343890	-1.3253750	3.9250520
H	-2.0997900	0.9032200	2.7149630
H	-1.0839430	1.7594990	3.8999190
H	-0.7825520	1.9520400	2.1516480
H	1.2893440	1.1986880	4.2029150
H	1.6862630	1.2130790	2.4665720
H	2.0257900	-0.2356890	3.4632120
C	-0.4545420	-3.2647570	0.6151260
H	-0.4958680	-2.0751260	1.4726870

H	0.6024780	-3.5033030	0.3663890
H	-1.0920020	-3.5905950	-0.2210840
H	-0.7159280	-3.8538500	1.5048790
P	-2.4086290	-0.7531820	-0.5598700
P	2.3842920	-0.8667840	-0.1632790

6

Symbol	X	Y	Z
C	1.7487350	2.8749820	-1.4234870
C	1.5348900	1.5605410	-0.9833270
C	2.6377620	0.8742760	-0.4385010
C	2.9721150	3.5086470	-1.2320640
C	4.0505230	2.8513010	-0.6284690
C	3.8677580	1.5152360	-0.2634250
P	-0.0178950	0.6175970	-1.2685680
C	3.0889610	-1.7403670	-1.6570710
C	2.8227820	-3.2458020	-1.6090130
C	2.6010260	-1.1234110	-2.9660220
C	3.5049130	-1.4207730	1.2752100
C	4.9441030	-1.7580420	0.8786140
C	2.8963750	-2.5539970	2.1051320
C	5.3571910	3.5576810	-0.3949240
C	-3.8009750	1.5699200	-0.0886740
C	-2.6076950	0.9213390	-0.4083860
C	-1.4634650	1.6510910	-0.7836650
C	-3.8943210	2.9651370	-0.0990690
C	-2.7505330	3.6841170	-0.4602690
C	-1.5643590	3.0455960	-0.8130500
C	-5.1730820	3.6717340	0.2603040

C	-3.7071990	-1.5759790	0.6778280
C	-2.7607520	-1.3229120	-2.1719520
C	-2.1716410	-2.6502560	-2.6436340
C	-4.2473460	-1.1830470	-2.4961510
C	-3.9055090	-3.0847840	0.5404630
C	-3.4940540	-1.1729170	2.1350010
V	-0.0298120	-1.2703680	0.2551960
C	-0.0404840	-0.4344120	1.7687660
C	0.0326620	0.5715160	2.8865120
C	-0.6243720	0.0060090	4.1556890
C	-0.6708810	1.8681670	2.4619540
C	1.5127370	0.8785010	3.1776940
H	0.9632710	3.4140470	-1.9525560
H	3.0954470	4.5395060	-1.5745210
H	4.7102380	0.9752070	0.1736050
H	4.1753340	-1.5701580	-1.5906340
H	3.1650470	-3.7112120	-0.6729540
H	3.3526560	-3.7445800	-2.4346870
H	1.7506840	-3.4697790	-1.7279460
H	1.5138730	-1.2296500	-3.0975010
H	3.0915990	-1.6350440	-3.8083740
H	2.8453830	-0.0541300	-3.0393180
H	3.5186290	-0.5138650	1.8989880
H	5.5453700	-1.8874730	1.7913210
H	5.4278950	-0.9767410	0.2770590
H	5.0032100	-2.7015820	0.3160530
H	2.8260720	-3.4924930	1.5329830
H	1.8945480	-2.3041870	2.4791770
H	3.5358170	-2.7536890	2.9785910
H	6.1620980	2.8517450	-0.1468070
H	5.2719790	4.2738800	0.4380400
H	5.6662700	4.1287680	-1.2828630

H	-4.6813790	0.9898650	0.1997300
H	-2.7852390	4.7769160	-0.4710410
H	-0.7105340	3.6635580	-1.0822370
H	-5.0100360	4.4103340	1.0599300
H	-5.9429260	2.9666280	0.6035750
H	-5.5794040	4.2166240	-0.6062750
H	-4.6213520	-1.0850940	0.3052660
H	-2.2224170	-0.5235840	-2.7089060
H	-2.3875850	-2.7855190	-3.7145670
H	-2.5902840	-3.5173690	-2.1116820
H	-1.0779250	-2.6704980	-2.5282150
H	-4.8529700	-1.9772120	-2.0348770
H	-4.3920490	-1.2540510	-3.5851020
H	-4.6489710	-0.2093260	-2.1784870
H	-4.1293640	-3.3932170	-0.4893110
H	-4.7590590	-3.3919740	1.1641160
H	-3.0281180	-3.6478740	0.8854040
H	-4.3878980	-1.4198160	2.7279400
H	-3.3017500	-0.0957710	2.2497500
H	-2.6449260	-1.7202860	2.5708000
H	-0.5122650	0.7208770	4.9850980
H	-0.1560900	-0.9413100	4.4671380
H	-1.6992800	-0.1703850	4.0112380
H	-1.7244740	1.6926250	2.1989900
H	-0.6391570	2.5942470	3.2890510
H	-0.1756350	2.3185270	1.5907500
H	1.5826270	1.6389970	3.9706670
H	2.0183290	1.2733930	2.2841550
H	2.0494690	-0.0171570	3.5257660
C	-0.4832410	-3.2248420	0.3744830
H	-0.2982580	-1.5124870	2.0637260
H	0.6421380	-3.3093070	0.4036560

H	-0.8309670	-3.7794030	-0.5078660
H	-0.8557120	-3.7028620	1.2906560
P	-2.3617410	-0.8857200	-0.3964640
P	2.3961690	-0.9027900	-0.1399680

6'

Symbol	X	Y	Z
C	0.2830610	-3.4436830	-0.3517520
C	0.7543510	-2.1330100	-0.4904340
C	2.0528020	-1.8390060	-0.0236010
C	1.0795000	-4.4263870	0.2263110
C	2.3798550	-4.1510190	0.6704670
C	2.8516310	-2.8427760	0.5319170
P	-0.1289250	-0.7836410	-1.3778860
C	4.0030600	0.1872480	0.8998030
C	4.5921520	1.5847650	0.7074530
C	3.7006080	-0.0917140	2.3734100
C	3.1809510	-0.0317060	-1.9782590
C	4.5327000	-0.7237260	-2.1381210
C	3.1459120	1.3592050	-2.6045000
C	3.2285430	-5.2303250	1.2849680
C	-4.1659310	-0.8995760	-0.5357220
C	-2.7941020	-0.6237860	-0.4616020
C	-1.8978800	-1.2603830	-1.3282270
C	-4.6695650	-1.8265530	-1.4503470
C	-3.7610300	-2.4709870	-2.3030130
C	-2.4028640	-2.1819210	-2.2579580
C	-6.1417770	-2.1211970	-1.5367750
C	-3.2559310	1.9162130	0.8950910

C	-2.2086450	-0.4334180	2.3867170
C	-3.6414300	-0.7207540	2.8303150
C	-1.3798450	-1.7162700	2.3228380
C	-2.9324030	2.8245170	2.0790460
C	-3.3193570	2.6645460	-0.4345040
V	0.3267450	1.0000290	0.3280870
C	0.3362320	2.5662100	-0.4172640
C	0.5421710	4.0463240	-0.5989770
C	2.0505090	4.3332420	-0.4676200
C	-0.2041960	4.8289250	0.4919940
C	0.0500420	4.4775760	-1.9890230
C	0.5272650	1.3488440	2.2883860
H	-0.7216580	-3.7012720	-0.6943280
H	0.6829310	-5.4399250	0.3317110
H	3.8609740	-2.6113170	0.8814470
H	4.7458070	-0.5503420	0.5531990
H	4.9624800	1.7484700	-0.3138820
H	5.4426930	1.7223060	1.3922040
H	3.8541650	2.3699290	0.9322740
H	3.1273840	0.7256360	2.8302330
H	4.6492100	-0.1715460	2.9257850
H	3.1459650	-1.0272060	2.5319720
H	2.4210490	-0.6395430	-2.4964240
H	4.7634840	-0.8282470	-3.2092470
H	4.5330300	-1.7353250	-1.7051640
H	5.3523330	-0.1463410	-1.6831800
H	3.8828680	2.0421170	-2.1568720
H	2.1502140	1.8154710	-2.5144860
H	3.3799760	1.2818180	-3.6772410
H	2.7560290	-5.6364130	2.1929830
H	4.2235030	-4.8550640	1.5621200
H	3.3668990	-6.0709690	0.5877230

H	-4.8661020	-0.3882950	0.1297370
H	-4.1323610	-3.1962110	-3.0318160
H	-1.7234400	-2.6663490	-2.9641610
H	-6.3368240	-3.1983500	-1.4220930
H	-6.5451670	-1.8212190	-2.5164590
H	-6.7082870	-1.5880980	-0.7609080
H	-4.2404860	1.4545600	1.0748220
H	-1.7470650	0.2475770	3.1192990
H	-3.6232110	-1.1878590	3.8271590
H	-4.1437920	-1.4252300	2.1511840
H	-4.2552610	0.1882250	2.9099290
H	-1.7928070	-2.4235790	1.5870630
H	-1.3868650	-2.2146130	3.3041320
H	-0.3299260	-1.5315410	2.0523160
H	-2.9727070	2.2880760	3.0388080
H	-3.6658770	3.6434680	2.1306360
H	-1.9369380	3.2775170	1.9777330
H	-4.0334660	3.4991250	-0.3627280
H	-3.6499150	2.0123930	-1.2567370
H	-2.3386460	3.0804390	-0.7074180
H	2.2409090	5.4053120	-0.6313040
H	2.6362050	3.7678540	-1.2053400
H	2.4146770	4.0753390	0.5380910
H	0.0847880	4.4868280	1.4974080
H	0.0411510	5.8994400	0.4168120
H	-1.2938300	4.7295460	0.3891620
H	0.1962630	5.5598430	-2.1268630
H	-1.0217210	4.2639450	-2.1206950
H	0.6025300	3.9600260	-2.7886400
H	1.4879500	1.9038030	2.2385450
H	0.6631590	0.4381480	2.8936630
H	-0.2085500	2.0126430	2.7657220

H	0.1254980	1.9414200	-1.3440570
P	2.5501500	-0.0898320	-0.2220540
P	-2.0700710	0.4999000	0.7768770

6’-7’-TS

Symbol	X	Y	Z
C	0.2563340	-3.6030370	-0.3927090
C	0.6849610	-2.2762270	-0.4806320
C	1.9804540	-1.9612250	-0.0156980
C	1.0943560	-4.5883460	0.1286330
C	2.3913360	-4.2946210	0.5580830
C	2.8196370	-2.9633600	0.4712790
P	-0.2287670	-0.8908190	-1.2618290
C	3.8533360	0.0468770	1.0141810
C	4.4521410	1.4470860	0.9092480
C	3.4894670	-0.2847120	2.4619440
C	3.1448390	-0.1214900	-1.8923580
C	4.5067110	-0.8026500	-2.0043160
C	3.1298790	1.2727660	-2.5085850
C	3.3053080	-5.3542490	1.1123380
C	-4.2722290	-0.7763010	-0.5010630
C	-2.8885260	-0.5680720	-0.4240930
C	-2.0065430	-1.3369450	-1.1944030
C	-4.8069610	-1.7708630	-1.3215820
C	-3.9163690	-2.5475390	-2.0781780
C	-2.5458490	-2.3251040	-2.0325070
C	-6.2900650	-2.0063500	-1.4059730
C	-3.2203490	2.0920910	0.7858460
C	-2.2435520	-0.2155500	2.3910240

C	-3.6781640	-0.4076570	2.8783920
C	-1.4838660	-1.5443520	2.3909010
C	-2.7953740	3.0540110	1.8931620
C	-3.2964030	2.7619300	-0.5852210
V	0.2999190	1.0106240	0.2620160
C	0.5325000	2.6413320	-0.4109980
C	0.9141060	4.0803820	-0.6572660
C	2.3279010	4.3265540	-0.1139450
C	-0.0728110	5.0171600	0.0618400
C	0.8720400	4.3634200	-2.1667290
C	0.7710240	1.5031720	1.9288730
H	-0.7445300	-3.8813600	-0.7293110
H	0.7282360	-5.6162760	0.1947250
H	3.8281980	-2.7192980	0.8159700
H	4.6100080	-0.6783890	0.6733070
H	4.8324210	1.6713120	-0.0970940
H	5.2971470	1.5419190	1.6083920
H	3.7093920	2.2124720	1.1764490
H	2.8788750	0.5113800	2.9097440
H	4.4105100	-0.3636320	3.0596440
H	2.9439700	-1.2335340	2.5659250
H	2.4091080	-0.7283230	-2.4465540
H	4.7786850	-0.9033320	-3.0661230
H	4.5023040	-1.8145100	-1.5720930
H	5.3024770	-0.2168400	-1.5185380
H	3.8243600	1.9647980	-2.0090510
H	2.1222720	1.7065770	-2.4716340
H	3.4308390	1.2120650	-3.5658200
H	2.8506660	-6.3523160	1.0483320
H	3.5437510	-5.1612390	2.1702860
H	4.2594340	-5.3835750	0.5636820
H	-4.9564890	-0.1610470	0.0888260

H	-4.3118310	-3.3253840	-2.7369210
H	-1.8837480	-2.9104810	-2.6756810
H	-6.6549990	-1.8629260	-2.4347320
H	-6.8478970	-1.3225980	-0.7512750
H	-6.5431600	-3.0372870	-1.1140880
H	-4.2212900	1.7040460	1.0340580
H	-1.7342640	0.4890560	3.0709580
H	-3.6612600	-0.8346900	3.8929930
H	-4.2315540	-1.1106840	2.2379390
H	-4.2411710	0.5352220	2.9328130
H	-1.9389760	-2.2627090	1.6919000
H	-1.5165370	-1.9898810	3.3965610
H	-0.4244170	-1.4460640	2.1078090
H	-2.8318530	2.5867490	2.8884430
H	-3.4716370	3.9220400	1.9137820
H	-1.7761570	3.4343530	1.7316560
H	-3.9862340	3.6187050	-0.5471770
H	-3.6631400	2.0722250	-1.3599010
H	-2.3137230	3.1397290	-0.9047450
H	2.6219130	5.3728660	-0.2875460
H	3.0620840	3.6762620	-0.6089620
H	2.3777950	4.1395310	0.9697650
H	-0.0620440	4.8542130	1.1510830
H	0.2098850	6.0654250	-0.1189340
H	-1.1033490	4.8811230	-0.2972540
H	1.1353440	5.4146060	-2.3582000
H	-0.1337850	4.1919090	-2.5813940
H	1.5840330	3.7344730	-2.7190030
H	0.2119460	2.4767630	1.0041420
H	0.3946680	0.5042000	2.2598020
H	1.1331300	2.1273560	2.7496850
H	-0.1735450	2.1705460	-1.1666370

P	2.4368210	-0.1895050	-0.1639820
P	-2.1093630	0.6130770	0.7195910

6'-7"-TS

Symbol	X	Y	Z
C	0.5406920	-3.5745760	-0.3298910
C	0.8854090	-2.2242910	-0.4565230
C	2.1596940	-1.8199380	-0.0057710
C	1.4349940	-4.4863210	0.2240350
C	2.7111900	-4.0991450	0.6524080
C	3.0594000	-2.7512070	0.5196180
P	-0.1260340	-0.9167560	-1.2574220
C	3.9019330	0.3793960	0.9299600
C	4.3843440	1.8152380	0.7267140
C	3.5788960	0.1144760	2.4008470
C	3.1580810	0.0581030	-1.9558380
C	4.5633940	-0.5285400	-2.0752640
C	3.0438700	1.4337690	-2.6086890
C	3.6593410	-5.0999000	1.2554730
C	-4.1667690	-1.0348530	-0.5507390
C	-2.7984200	-0.7509390	-0.4569800
C	-1.8686310	-1.4785060	-1.2101420
C	-4.6357030	-2.0641680	-1.3695780
C	-3.6950950	-2.7998210	-2.1061610
C	-2.3390400	-2.5028920	-2.0453420
C	-6.1029560	-2.3795150	-1.4715200

C	-3.3116260	1.8369210	0.8324000
C	-2.1799410	-0.4420730	2.3507730
C	-3.6024470	-0.7433780	2.8184630
C	-1.3389250	-1.7191300	2.3257130
C	-2.9922830	2.7587000	2.0074740
C	-3.4057830	2.5760600	-0.4996520
V	0.2570560	1.0120430	0.2715310
C	0.2658420	2.6854660	-0.3406370
C	0.3734000	4.1707170	-0.5817140
C	1.8570920	4.5755390	-0.4897370
C	-0.4284890	4.9497170	0.4680920
C	-0.1510340	4.4823950	-1.9925510
C	0.4548900	1.4966260	1.9852970
H	-0.4413260	-3.9228030	-0.6578730
H	1.1338330	-5.5329780	0.3227550
H	4.0487600	-2.4304080	0.8571150
H	4.7112700	-0.3037920	0.6240380
H	4.7831310	1.9885390	-0.2821700
H	5.1923510	2.0376710	1.4402230
H	3.5753150	2.5398380	0.9073350
H	2.9113650	0.8877720	2.8083170
H	4.5090170	0.1479410	2.9884000
H	3.1160660	-0.8685110	2.5717670
H	2.4593010	-0.6117580	-2.4839290
H	4.8247660	-0.6372320	-3.1388970
H	4.6365130	-1.5269740	-1.6192510
H	5.3236470	0.1227920	-1.6170920
H	3.6609800	2.1946630	-2.1090450
H	2.0053480	1.7916790	-2.6352820
H	3.3875810	1.3689560	-3.6523150
H	3.7695810	-5.9836320	0.6090880
H	3.2916320	-5.4556470	2.2312080

H	4.6583670	-4.6692440	1.4116870
H	-4.8914440	-0.4553970	0.0272090
H	-4.0400130	-3.6051400	-2.7603680
H	-1.6369490	-3.0578070	-2.6730700
H	-6.7059250	-1.7200930	-0.8320560
H	-6.3053530	-3.4195000	-1.1723170
H	-6.4605920	-2.2657090	-2.5064960
H	-4.2823880	1.3521670	1.0248550
H	-1.7195090	0.2732080	3.0528320
H	-3.5648920	-1.1985130	3.8203640
H	-4.1022520	-1.4633410	2.1533630
H	-4.2307680	0.1555570	2.8944970
H	-1.7486850	-2.4514160	1.6130320
H	-1.3430330	-2.1859790	3.3223310
H	-0.2897140	-1.5456870	2.0452600
H	-3.0660910	2.2401460	2.9749250
H	-3.7050580	3.5972480	2.0298220
H	-1.9805330	3.1782540	1.9221090
H	-4.1615900	3.3740300	-0.4381010
H	-3.6935150	1.9046330	-1.3227170
H	-2.4432180	3.0371360	-0.7608180
H	1.9726690	5.6411460	-0.7402550
H	2.4783060	3.9932950	-1.1858190
H	2.2491900	4.4295700	0.5288820
H	-0.1225710	4.6814030	1.4916240
H	-0.2567720	6.0294120	0.3424190
H	-1.5072780	4.7660860	0.3710130
H	-0.1059900	5.5657570	-2.1803440
H	-1.1949470	4.1617240	-2.1213030
H	0.4560380	3.9843280	-2.7646150
H	0.9434760	2.3770830	0.8944390
H	0.7144200	0.4209030	2.1711170

H	0.4941960	2.1191630	2.8827100
H	0.4535190	2.0548680	-1.2674360
P	2.4949850	-0.0319150	-0.2103740
P	-2.0865810	0.4562720	0.7103550

7’

Symbol	X	Y	Z
C	0.9026630	-3.3851590	-1.0269880
C	1.0872790	-2.0137320	-0.8348550
C	2.2659330	-1.5833810	-0.1967250
C	1.8615340	-4.2994920	-0.5944910
C	3.0441950	-3.8823130	0.0228590
C	3.2305060	-2.5070230	0.2080850
P	-0.0679930	-0.7124000	-1.4112810
C	3.5496940	0.4805440	1.4607060
C	3.8520190	1.9616100	1.6848640
C	3.0220320	-0.1861620	2.7312830
C	3.3793020	0.6963880	-1.5310310
C	4.8177490	0.1836760	-1.5216410
C	3.3052420	2.1782800	-1.8845230
C	4.0908210	-4.8627210	0.4782930
C	-3.9117330	-1.5792610	-0.2488510
C	-2.6084830	-1.0702290	-0.3069000
C	-1.7262390	-1.4912560	-1.3088820
C	-4.3576960	-2.5247860	-1.1749890
C	-3.4673390	-2.9362190	-2.1777270
C	-2.1790470	-2.4199140	-2.2558900
C	-5.7552160	-3.0781440	-1.1194200
C	-3.3477710	1.2460110	1.3093440
C	-1.5729750	-0.8432060	2.4309450
C	-2.8006490	-1.5499050	3.0011430

C	-0.4215760	-1.8204520	2.2074360
C	-3.0175650	2.1953310	2.4594540
C	-3.8932620	1.9764930	0.0820140
V	0.1132780	1.1974670	0.0675800
C	-0.2963580	2.7316670	-1.0844590
C	-0.3606940	4.2606410	-0.9913390
C	1.0506490	4.8499500	-1.1071970
C	-0.9998180	4.7444740	0.3151400
C	-1.2148940	4.7629880	-2.1704790
C	0.3031500	1.6763030	1.7345590
H	-0.0050580	-3.7577650	-1.5055580
H	1.6826000	-5.3673710	-0.7446550
H	4.1440970	-2.1686010	0.7043710
H	4.4887540	-0.0205390	1.1740320
H	4.3638190	2.4229080	0.8291040
H	4.5108120	2.0761170	2.5589060
H	2.9338910	2.5351240	1.8864370
H	2.0975650	0.2916070	3.0836150
H	3.7716100	-0.0873860	3.5314510
H	2.8195620	-1.2575830	2.5960110
H	2.8150710	0.1420880	-2.3014290
H	5.2655950	0.3399190	-2.5149760
H	4.8806910	-0.8917480	-1.3023130
H	5.4418710	0.7278910	-0.7956680
H	3.8563620	2.8104730	-1.1736750
H	2.2693550	2.5323570	-1.9224260
H	3.7481220	2.3417270	-2.8788330
H	3.7512570	-5.9004180	0.3563460
H	4.3449200	-4.7127480	1.5387730
H	5.0210890	-4.7443030	-0.0993680
H	-4.6020630	-1.2407890	0.5277930
H	-3.8015700	-3.6623550	-2.9234910

H	-1.5228410	-2.7284780	-3.0736480
H	-6.3337400	-2.7750380	-2.0062030
H	-6.2955360	-2.7287310	-0.2289030
H	-5.7442770	-4.1785120	-1.1005370
H	-4.1317310	0.5525250	1.6551180
H	-1.2428860	-0.0739340	3.1469070
H	-2.5228750	-2.0464290	3.9436290
H	-3.1720550	-2.3294470	2.3193180
H	-3.6271480	-0.8618060	3.2305720
H	-0.6813240	-2.5890180	1.4635510
H	-0.1822540	-2.3343290	3.1509230
H	0.4880210	-1.3143230	1.8612390
H	-2.7583390	1.6581750	3.3837790
H	-3.8930970	2.8238780	2.6826970
H	-2.1813800	2.8636610	2.2079890
H	-4.8282610	2.4932990	0.3461590
H	-4.1151910	1.2913010	-0.7492930
H	-3.1950130	2.7422280	-0.2852940
H	1.0133340	5.9478240	-1.0340730
H	1.5174950	4.6010860	-2.0721260
H	1.7096170	4.4838150	-0.3038120
H	-0.3986980	4.4837600	1.2000220
H	-1.0945460	5.8407890	0.3047440
H	-2.0090420	4.3308310	0.4557780
H	-1.2653280	5.8631810	-2.1742730
H	-2.2461170	4.3800610	-2.1099260
H	-0.7927930	4.4415330	-3.1351610
H	-1.3242460	2.2654520	-1.0054330
H	0.3834310	1.6749770	2.8258870
H	0.4560250	2.6928570	1.2628320
H	0.0964400	2.3953210	-2.0629860
P	2.4226810	0.2264440	0.0077210

P	-1.9212290	0.1337650	0.8824470

7”

Symbol	X	Y	Z
C	1.3000290	-2.9728310	-1.3291160
C	1.3062030	-1.6080390	-1.0108880
C	2.4433280	-1.0770310	-0.3802970
C	2.3924110	-3.7765300	-1.0258050
C	3.5422620	-3.2525100	-0.4182840
C	3.5483780	-1.8920200	-0.1066140
P	-0.0233370	-0.4290930	-1.4576930
C	3.4756930	0.9054470	1.5001250
C	3.6448840	2.3626000	1.9291730
C	3.0094380	0.0320110	2.6673810
C	3.2008440	1.5190020	-1.4462400
C	4.5820900	0.9491410	-1.7636300
C	3.2363310	3.0493580	-1.3711080
C	4.7207020	-4.1355700	-0.1103990
C	-3.5087220	-1.9946710	0.0421920
C	-2.3469350	-1.2386650	-0.1439180
C	-1.5627810	-1.4143860	-1.2884070
C	-3.9284810	-2.9160290	-0.9207910
C	-3.1544110	-3.0576640	-2.0816370
C	-1.9918810	-2.3176800	-2.2685300
C	-5.1763830	-3.7339630	-0.7295730
C	-3.0875800	0.5238850	2.0876380
C	-0.5389880	-1.1934240	2.1342300
C	-0.5132840	-0.8440430	3.6201070
C	-0.8233010	-2.6828470	1.9390740
C	-2.7271610	1.6117760	3.1013550
C	-4.2440640	1.0156350	1.2076530

V	0.0040970	1.4229610	0.0912550
C	-0.3253660	2.9957540	-1.1277700
C	-1.7528780	3.3919620	-1.5256040
C	-1.6823680	4.4092810	-2.6753770
C	-2.4427730	4.0406420	-0.3194720
C	-2.5382080	2.1627790	-1.9989160
C	0.2562060	1.9725790	1.7451520
H	0.4305230	-3.4196450	-1.8154760
H	2.3564710	-4.8407250	-1.2740620
H	4.4347020	-1.4716060	0.3748280
H	4.4582890	0.5357060	1.1631020
H	4.1857860	2.9609350	1.1860680
H	4.2240890	2.4021930	2.8642410
H	2.6760540	2.8492840	2.1198790
H	2.0656820	0.3990210	3.0935580
H	3.7639170	0.0619140	3.4681390
H	2.8699740	-1.0209720	2.3848700
H	2.5123630	1.2268780	-2.2585610
H	4.9757210	1.4392980	-2.6676140
H	4.5561570	-0.1317140	-1.9583770
H	5.2994660	1.1436550	-0.9506310
H	4.1284270	3.4017050	-0.8344010
H	2.3568000	3.4837610	-0.8784280
H	3.2879120	3.4670090	-2.3876280
H	5.1122910	-4.6052500	-1.0258790
H	4.4391350	-4.9497070	0.5753330
H	5.5403070	-3.5708110	0.3550500
H	-4.1036600	-1.8826430	0.9524890
H	-3.4754990	-3.7583450	-2.8570150
H	-1.4155210	-2.4378390	-3.1894610
H	-4.9462860	-4.8106030	-0.7263520
H	-5.8919250	-3.5579440	-1.5474230

H	-5.6791590	-3.4925380	0.2170920
H	-3.4212140	-0.3747760	2.6362950
H	0.4626670	-0.9917510	1.7208880
H	0.2166570	-1.4955100	4.1239310
H	-1.4885720	-1.0161180	4.1004750
H	-0.2108990	0.1920540	3.8152670
H	-1.8327860	-2.9580270	2.2818770
H	-0.0992900	-3.2611560	2.5336110
H	-0.7197180	-2.9992050	0.8935690
H	-1.9569960	1.3087090	3.8185090
H	-3.6270000	1.8743950	3.6782660
H	-2.3822010	2.5270460	2.5975580
H	-5.1154650	1.2216340	1.8476580
H	-4.5578380	0.2948210	0.4430470
H	-3.9881360	1.9539090	0.6970900
H	-2.6927000	4.7384310	-2.9677310
H	-1.2014740	3.9712230	-3.5639050
H	-1.1076570	5.3028680	-2.3854960
H	-1.9199920	4.9599890	-0.0106130
H	-3.4845870	4.3114770	-0.5519750
H	-2.4552850	3.3626540	0.5470930
H	-3.5532250	2.4440550	-2.3215050
H	-2.6516850	1.4096500	-1.2042850
H	-2.0382230	1.6710250	-2.8478690
H	0.2219830	3.8635180	-0.7229920
H	0.0536640	3.0158320	1.3904520
H	0.4848760	1.9142270	2.8128220
H	0.2456120	2.6027510	-1.9931430
P	2.3722540	0.7064770	0.0174690
P	-1.6366620	-0.0771520	1.0774680

Cr^{IV} original ligand (L_0)

1

Symbol	X	Y	Z
C	0.6647450	-3.5671510	0.6303410
C	0.8649320	-2.2220280	0.2221960
C	2.2103920	-1.7663430	0.2034230
C	1.7236460	-4.3952280	0.9615840
C	3.0550230	-3.9533980	0.9244480
C	3.2601770	-2.6279370	0.5504190
N	-0.1654550	-1.3506970	-0.0656820
P	2.4605290	-0.0025200	-0.1942130
C	3.7444150	0.5155850	1.0576720
C	4.2570420	1.9386900	0.8420930
C	3.2228900	0.3203960	2.4828330
C	3.3060260	0.0007100	-1.8587590
C	4.7470530	-0.5038560	-1.8645890
C	3.1662900	1.3457720	-2.5710440
C	4.1939690	-4.8690360	1.2876000
C	-3.8565680	-1.5392790	-0.1820010
C	-2.5588050	-1.1407150	0.1281260
C	-1.4355320	-1.8394720	-0.3741770
C	-4.1041540	-2.6367730	-1.0148380
C	-2.9929630	-3.3132670	-1.5293840
C	-1.6898800	-2.9321610	-1.2215420
C	-5.5131440	-3.0631650	-1.3337710
P	-2.0692980	0.2968480	1.1248720
C	-3.5722840	1.3659680	1.2893350
C	-1.7512180	-0.4975380	2.7884320
C	-0.8135240	0.3259430	3.6715020
C	-3.0187150	-0.9446050	3.5118350

C	-3.3680970	2.4677640	2.3305290
C	-3.9548200	1.9556050	-0.0685510
Cr	0.0656670	0.7585540	0.0355130
C	-0.6182470	1.2863390	-1.6080290
C	-0.8590080	0.7646990	-3.0132040
C	-0.5446590	1.8935640	-4.0143210
C	-2.3505340	0.3993670	-3.1516450
C	-0.0068320	-0.4585390	-3.3678450
C	0.4511380	2.5949620	1.0071690
C	0.5620330	4.0235090	0.4200560
C	-0.8275000	4.6014380	0.1210820
C	1.2388990	4.9468700	1.4506540
C	1.3966090	4.0460870	-0.8664130
H	-0.3507670	-3.9570280	0.7063280
H	1.5087920	-5.4208100	1.2781260
H	4.2851120	-2.2468400	0.5515290
H	4.5924210	-0.1732580	0.9101210
H	4.6930810	2.0834020	-0.1563000
H	5.0440190	2.1643040	1.5793300
H	3.4578860	2.6816660	0.9748740
H	2.4042960	1.0200600	2.7105260
H	4.0302440	0.5170100	3.2059460
H	2.8608190	-0.7035890	2.6565390
H	2.6893500	-0.7337950	-2.4010950
H	5.1147950	-0.5539290	-2.9019960
H	4.8378790	-1.5132200	-1.4399100
H	5.4230700	0.1683100	-1.3134420
H	3.8267070	2.1127320	-2.1390130
H	2.1361920	1.7220370	-2.5140980
H	3.4380100	1.2403300	-3.6335920
H	4.3003300	-5.6918710	0.5617130
H	4.0436700	-5.3303710	2.2768320

H	5.1513480	-4.3281900	1.3152130
H	-4.7076070	-0.9808590	0.2196350
H	-3.1484910	-4.1602320	-2.2040030
H	-0.8532650	-3.4785460	-1.6612860
H	-5.5341530	-3.8033270	-2.1465790
H	-6.1344510	-2.2082440	-1.6426550
H	-6.0025860	-3.5208780	-0.4581520
H	-4.3880340	0.7079880	1.6350900
H	-1.1978910	-1.4006930	2.4817470
H	-0.5748760	-0.2337350	4.5899540
H	-1.2506280	1.2892590	3.9739240
H	0.1373020	0.5393150	3.1607730
H	-3.6088400	-0.0914060	3.8810710
H	-2.7564430	-1.5617920	4.3860590
H	-3.6638390	-1.5544960	2.8608980
H	-3.2334720	2.0686970	3.3456110
H	-4.2500890	3.1272420	2.3518180
H	-2.4936010	3.0889770	2.0882980
H	-4.8923290	2.5268000	0.0217170
H	-4.1028130	1.1811650	-0.8336690
H	-3.1755910	2.6422620	-0.4305810
H	-0.7215110	1.5582160	-5.0501910
H	0.5065820	2.2136880	-3.9424980
H	-1.1777190	2.7770390	-3.8330960
H	-2.9932060	1.2584200	-2.9032040
H	-2.5777520	0.1011830	-4.1890850
H	-2.6222420	-0.4369980	-2.4924560
H	-0.3316710	-0.8944180	-4.3267250
H	-0.0757170	-1.2336000	-2.5943180
H	1.0493910	-0.1775110	-3.4795030
H	-1.0554850	2.2933240	-1.4877500
H	-0.2592640	2.6541780	1.8562770

H	1.4219500	2.3736470	1.4930590
H	-0.7538710	5.6093570	-0.3207430
H	-1.4275750	4.6895270	1.0411890
H	-1.3890270	3.9727650	-0.5852300
H	1.2848490	5.9921910	1.0985650
H	2.2714400	4.6205600	1.6584360
H	0.6908730	4.9382530	2.4072340
H	0.9072760	3.4732220	-1.6667620
H	2.3903400	3.6027650	-0.7025850
H	1.5477300	5.0777640	-1.2276630

1-2-TS

Symbol	X	Y	Z
C	0.9096550	-3.5542300	-0.4530860
C	1.0401140	-2.1387860	-0.4627530
C	2.3429400	-1.6260360	-0.1987600
C	1.9970800	-4.3795280	-0.2151490
C	3.2830780	-3.8754740	0.0344630
C	3.4188370	-2.4878800	0.0390430
N	0.0006900	-1.2682410	-0.6439330
P	2.4485970	0.1871590	-0.1982680
C	3.8268870	0.6008940	0.9813590
C	4.1975940	2.0833180	0.9253160
C	3.4279830	0.1877920	2.3989000
C	3.0655700	0.5613930	-1.9250630
C	4.4986930	0.1094860	-2.1915720
C	2.8161020	2.0073800	-2.3509650
C	4.4498290	-4.7963910	0.2822340
C	-3.6268550	-1.8173190	-0.2408440

C	-2.3134480	-1.4205840	-0.0025990
C	-1.2860990	-1.7476440	-0.9168650
C	-3.9705640	-2.5522670	-1.3845420
C	-2.9469720	-2.8781570	-2.2795370
C	-1.6279870	-2.4842860	-2.0572370
C	-5.3969900	-2.9753860	-1.6232140
P	-1.6751650	-0.4658170	1.4243100
C	-3.1771060	0.2953370	2.2102930
C	-1.0806290	-1.8411070	2.5398970
C	-0.0582580	-1.3441330	3.5613350
C	-2.1956940	-2.6772130	3.1607510
C	-2.8552700	0.8974080	3.5797510
C	-3.7525250	1.3537530	1.2673810
Cr	-0.0008490	0.7511040	-0.0536370
C	-0.7723570	1.5027930	-1.3753550
C	-1.4947430	1.9436150	-2.6092770
C	-1.0353460	3.3288240	-3.0908250
C	-3.0099620	1.9543730	-2.3447740
C	-1.1708810	0.8990440	-3.6936420
C	0.1375250	2.5921770	1.1787260
C	0.0857620	4.1214660	0.9719180
C	-1.3512280	4.5640120	0.6677620
C	0.5488330	4.8067450	2.2665980
C	1.0180070	4.5387240	-0.1707930
H	-0.0687050	-4.0047090	-0.6250210
H	1.8402970	-5.4631130	-0.2138560
H	4.4063070	-2.0632760	0.2454940
H	4.7108780	0.0150830	0.6792400
H	4.5968900	2.3795140	-0.0545310
H	4.9723810	2.3059470	1.6763380
H	3.3307300	2.7260700	1.1452080
H	2.5617500	0.7709160	2.7521670

H	4.2589320	0.3737570	3.0980840
H	3.1684900	-0.8790960	2.4617990
H	2.3833020	-0.0744390	-2.5153490
H	4.7387130	0.2217050	-3.2612830
H	4.6522090	-0.9480140	-1.9305530
H	5.2294420	0.7138860	-1.6301100
H	3.4837610	2.7179590	-1.8405790
H	1.7781230	2.2979700	-2.1372820
H	2.9875890	2.1174600	-3.4341200
H	4.6596280	-5.4325820	-0.5934040
H	4.2631780	-5.4719890	1.1329570
H	5.3668770	-4.2307700	0.5048310
H	-4.4149170	-1.5584700	0.4732460
H	-3.1872890	-3.4469020	-3.1824490
H	-0.8483940	-2.7371910	-2.7792720
H	-5.5009160	-3.5206740	-2.5721780
H	-6.0750920	-2.1079630	-1.6607530
H	-5.7599240	-3.6352950	-0.8187650
H	-3.9264590	-0.5019280	2.3532130
H	-0.5353270	-2.4769630	1.8224800
H	0.3992010	-2.1970820	4.0878110
H	-0.5060850	-0.6871690	4.3228010
H	0.7503030	-0.7861230	3.0669790
H	-2.7693110	-2.1100140	3.9109580
H	-1.7724360	-3.5574160	3.6712480
H	-2.8989590	-3.0458130	2.3982530
H	-2.5505300	0.1368140	4.3123400
H	-3.7459410	1.4035820	3.9851410
H	-2.0509820	1.6481140	3.5168750
H	-4.6598180	1.8020410	1.7035590
H	-4.0196490	0.9345070	0.2877290
H	-3.0254900	2.1611290	1.0908810

H	-1.5488290	3.5941290	-4.0294910
H	0.0479780	3.3441460	-3.2852620
H	-1.2628700	4.1147540	-2.3554220
H	-3.2713960	2.6815070	-1.5599860
H	-3.5653900	2.2259330	-3.2580470
H	-3.3515560	0.9604600	-2.0170950
H	-1.6949320	1.1460670	-4.6318400
H	-1.4853520	-0.1055700	-3.3761620
H	-0.0899240	0.8674790	-3.8992700
H	-0.4341430	2.3235660	-0.2052700
H	-0.5646140	2.3209340	1.9821870
H	1.1469810	2.3279670	1.5313360
H	-1.4031050	5.6460050	0.4661960
H	-2.0167920	4.3498090	1.5196670
H	-1.7614730	4.0415380	-0.2105740
H	0.5021680	5.9049680	2.1782450
H	1.5888200	4.5343720	2.5093610
H	-0.0817400	4.5114370	3.1208540
H	0.7182690	4.0791170	-1.1224500
H	2.0548840	4.2282710	0.0329310
H	1.0148840	5.6320390	-0.3090940

A

Symbol	X	Y	Z
C	1.0225180	-3.4112350	0.2972820
C	1.0789580	-2.0277880	-0.0052890
C	2.3543180	-1.4115650	0.1044690
C	2.1576630	-4.1240370	0.6519790
C	3.4212300	-3.5210750	0.7399040

C	3.4827430	-2.1550670	0.4664710
N	-0.0236240	-1.2532720	-0.3127710
P	2.3639570	0.3821220	-0.2263900
C	3.5567130	1.0736770	1.0160720
C	3.9283490	2.5253550	0.7246320
C	2.9285940	0.9349670	2.4047030
C	3.1042420	0.5265380	-1.9314280
C	4.5806210	0.1475930	-2.0183000
C	2.8046620	1.8792330	-2.5796160
C	4.6397440	-4.3143320	1.1333310
C	-3.6409910	-1.7724640	-0.8865560
C	-2.4318120	-1.2627860	-0.4098100
C	-1.1932810	-1.8287760	-0.8001810
C	-3.6840990	-2.8486960	-1.7760820
C	-2.4566720	-3.3925010	-2.1802520
C	-1.2433630	-2.9036000	-1.7126110
C	-4.9869110	-3.3888660	-2.3056490
P	-2.2241430	0.1514830	0.7212570
C	-3.8042770	1.1114680	0.6577170
C	-2.1003920	-0.6829440	2.3855280
C	-1.4100890	0.2004350	3.4256830
C	-3.4127740	-1.2841350	2.8797340
C	-3.8382840	2.1722170	1.7596040
C	-3.9488130	1.7380740	-0.7304950
Cr	-0.0187890	0.8644550	-0.0416190
C	-0.1178650	2.6165880	-0.2381520
C	-0.2670690	4.0614910	-0.6015950
C	1.0707760	4.7949210	-0.3977690
C	-1.3340240	4.7217050	0.2913880
C	-0.6918570	4.1878800	-2.0769510
H	0.0615590	-3.9271140	0.2720620
H	2.0567470	-5.1888710	0.8852430

H	4.4496170	-1.6503250	0.5546950
H	4.4719030	0.4600880	0.9731530
H	4.4972450	2.6346570	-0.2096550
H	4.5563640	2.9213030	1.5386690
H	3.0300940	3.1549680	0.6551280
H	2.0223770	1.5590730	2.4787330
H	3.6343760	1.2727070	3.1800900
H	2.6514380	-0.1055520	2.6328410
H	2.5197910	-0.2370190	-2.4740090
H	4.9123370	0.1622820	-3.0690040
H	4.7687640	-0.8637310	-1.6301240
H	5.2186890	0.8544980	-1.4647940
H	3.3723450	2.6974520	-2.1117850
H	1.7358980	2.1275160	-2.5042820
H	3.0799510	1.8550040	-3.6462080
H	4.5206450	-4.7746970	2.1276540
H	5.5384460	-3.6809650	1.1671590
H	4.8380100	-5.1323370	0.4218040
H	-4.5829560	-1.3194140	-0.5623710
H	-2.4501420	-4.2214430	-2.8948440
H	-0.3125230	-3.3477240	-2.0702750
H	-5.8483750	-2.9169250	-1.8109180
H	-5.0667540	-4.4762760	-2.1495750
H	-5.0873790	-3.2090830	-3.3885660
H	-4.6346220	0.4042940	0.8236510
H	-1.4087190	-1.5086990	2.1473960
H	-1.2069300	-0.3795970	4.3401280
H	-2.0219940	1.0685750	3.7126080
H	-0.4472820	0.5808240	3.0496870
H	-4.1436170	-0.5074170	3.1547030
H	-3.2353990	-1.8976820	3.7776140
H	-3.8716970	-1.9356680	2.1203440

H	-3.9332990	1.7282770	2.7608330
H	-4.7019540	2.8401380	1.6136310
H	-2.9284460	2.7913740	1.7449580
H	-4.8791180	2.3250630	-0.7903980
H	-3.9754120	0.9809300	-1.5278940
H	-3.1025680	2.4104880	-0.9396110
H	0.9833320	5.8552570	-0.6891840
H	1.8670640	4.3409810	-1.0063670
H	1.3819120	4.7603330	0.6578810
H	-1.0878160	4.5963440	1.3574220
H	-1.4046060	5.8026250	0.0822910
H	-2.3271440	4.2817900	0.1170750
H	-0.8326460	5.2454870	-2.3586060
H	-1.6388800	3.6582850	-2.2637090
H	0.0691070	3.7581840	-2.7465950

5-6-TS

Symbol	X	Y	Z
C	1.0194540	-2.6515130	-1.6460870
C	1.0967940	-1.3716770	-1.0322800
C	2.3868130	-0.9635260	-0.5840720
C	2.1433520	-3.4451530	-1.8066450
C	3.4163410	-3.0423250	-1.3716210
C	3.5002370	-1.7933440	-0.7582780
N	0.0205390	-0.5639330	-0.7980690
P	2.4292150	0.6562000	0.2388200
C	3.7835170	0.5262220	1.5084870
C	4.0817600	1.8783600	2.1580740
C	3.4034300	-0.5149680	2.5617780

C	3.0375830	1.8151460	-1.0986900
C	4.4812850	1.5763790	-1.5317370
C	2.7577370	3.2861660	-0.7868950
C	4.6193730	-3.9319780	-1.5479940
C	-3.6063240	-1.2311650	-0.6784370
C	-2.2931090	-0.9643630	-0.2915860
C	-1.2600210	-0.8971960	-1.2517000
C	-3.9432390	-1.4319770	-2.0215390
C	-2.9138000	-1.3535150	-2.9688730
C	-1.5975980	-1.0929080	-2.5991190
C	-5.3627890	-1.7074600	-2.4450420
P	-1.6826360	-0.6262310	1.4026460
C	-3.1871140	-0.1918630	2.3997210
C	-1.1216960	-2.3301600	1.9268520
C	-0.0988530	-2.2755890	3.0604150
C	-2.2593680	-3.3119310	2.1918760
C	-2.8364430	-0.0960640	3.8860480
C	-3.7756910	1.1249640	1.8893180
Cr	-0.0219940	1.0488790	0.5138770
C	-0.8257840	2.2064510	-0.4396060
C	-1.5009280	3.1413520	-1.3877330
C	-1.4922980	4.5652630	-0.8089270
C	-2.9473980	2.6703790	-1.6195970
C	-0.7271650	3.1030690	-2.7175480
H	0.0525580	-3.0266750	-1.9841330
H	2.0283630	-4.4258890	-2.2797470
H	4.4757480	-1.4589600	-0.3913680
H	4.6941080	0.1850110	0.9884720
H	4.5129920	2.5974170	1.4478500
H	4.8107460	1.7506710	2.9742970
H	3.1742870	2.3288330	2.5907950
H	2.5291320	-0.1828840	3.1445100

H	4.2352980	-0.6625740	3.2690160
H	3.1650530	-1.4912780	2.1150160
H	2.3690550	1.5243100	-1.9276230
H	4.7215310	2.1949790	-2.4115640
H	4.6589360	0.5271830	-1.8100790
H	5.1960610	1.8491470	-0.7385070
H	3.4069430	3.6772110	0.0107920
H	1.7114830	3.4313580	-0.4804520
H	2.9383930	3.9011700	-1.6834000
H	5.5322100	-3.4537130	-1.1629360
H	4.7964310	-4.1737480	-2.6087530
H	4.4988820	-4.8899980	-1.0158700
H	-4.3974340	-1.2810620	0.0757870
H	-3.1511900	-1.4919100	-4.0279970
H	-0.8161310	-1.0226450	-3.3590880
H	-5.7574870	-0.8957110	-3.0772860
H	-6.0304990	-1.8063050	-1.5769770
H	-5.4351490	-2.6379850	-3.0300330
H	-3.9344760	-0.9925550	2.2668800
H	-0.5843320	-2.6621310	1.0223790
H	0.3301100	-3.2753590	3.2353620
H	-0.5387620	-1.9337220	4.0100020
H	0.7296670	-1.5987230	2.8069580
H	-2.8181450	-3.0576320	3.1068010
H	-1.8602730	-4.3303030	2.3265790
H	-2.9711780	-3.3458700	1.3527960
H	-2.5301200	-1.0643380	4.3069810
H	-3.7112290	0.2487960	4.4600700
H	-2.0196020	0.6235640	4.0591130
H	-4.6752930	1.3889940	2.4682660
H	-4.0592720	1.0674990	0.8291820
H	-3.0503000	1.9472330	1.9929110

H	-1.9785710	5.2703670	-1.5029310
H	-0.4637000	4.9181340	-0.6336340
H	-2.0350380	4.6083000	0.1489450
H	-3.5330830	2.7307520	-0.6901780
H	-3.4455870	3.2969350	-2.3783450
H	-2.9681110	1.6249720	-1.9652440
H	-1.2128000	3.7454360	-3.4710520
H	-0.6885250	2.0763640	-3.1124480
H	0.3078730	3.4526290	-2.5833110
C	0.1431010	2.4198750	2.2620950
H	-0.4487460	2.5741380	0.9440880
H	0.9429320	3.1740250	2.2105870
H	0.4826290	1.5998610	2.9125190
H	-0.7279330	2.8818840	2.7535010

6

Symbol	X	Y	Z
C	1.0446670	-3.1190780	0.4799850
C	1.0944270	-1.7339560	0.1748760
C	2.3890240	-1.1456220	0.1482570
C	2.1935030	-3.8637180	0.6863890
C	3.4739890	-3.2921010	0.6252770
C	3.5327200	-1.9243580	0.3729410
N	-0.0290500	-0.9499770	-0.0053510
P	2.4477460	0.6683620	-0.0272890
C	3.5193010	1.1850420	1.4070940
C	3.8682610	2.6729950	1.3892040
C	2.8367010	0.7989820	2.7197530
C	3.3939340	1.0527830	-1.5848400

C	4.8958020	0.7775500	-1.5364620
C	3.0789980	2.4644770	-2.0849610
C	4.7120890	-4.1219830	0.8400450
C	-3.6998480	-1.3734370	-0.2352410
C	-2.4385540	-0.8968170	0.1242430
C	-1.2563830	-1.5149010	-0.3499270
C	-3.8536630	-2.4636910	-1.0943930
C	-2.6837750	-3.0562290	-1.5885180
C	-1.4200690	-2.6039040	-1.2303810
C	-5.2127740	-2.9632180	-1.5086870
P	-2.1047350	0.5781510	1.1323820
C	-3.6527090	1.5940300	1.1564980
C	-1.8812790	-0.1626320	2.8329180
C	-1.0833310	0.7368340	3.7763840
C	-3.1794170	-0.6730280	3.4524180
C	-3.5458890	2.7333940	2.1729120
C	-3.9530130	2.1285340	-0.2436260
Cr	0.0393350	1.1504220	0.1273310
C	-0.5030870	1.7087330	-1.5662570
C	-0.6801880	1.1339600	-2.9651720
C	-0.4083410	2.2393040	-4.0030740
C	-2.1413280	0.6717910	-3.1336600
C	0.2585650	-0.0439950	-3.2535140
H	0.0756270	-3.6102640	0.5759450
H	2.0934940	-4.9280690	0.9217440
H	4.5133700	-1.4403390	0.3722870
H	4.4523680	0.6037940	1.3136620
H	4.4579320	2.9587980	0.5071290
H	4.4703880	2.9222010	2.2776140
H	2.9639130	3.2982050	1.4128790
H	1.9383370	1.4137620	2.8830170
H	3.5154430	0.9788530	3.5683160

H	2.5445430	-0.2616020	2.7445000
H	2.9435880	0.3369900	-2.2899090
H	5.3384860	0.9786180	-2.5251930
H	5.1202600	-0.2693900	-1.2901520
H	5.4156680	1.4225710	-0.8116110
H	3.5570170	3.2380210	-1.4652950
H	1.9960490	2.6557760	-2.0811290
H	3.4500520	2.5908960	-3.1145880
H	5.6190320	-3.4995440	0.8394890
H	4.8314810	-4.8803260	0.0489950
H	4.6781260	-4.6610410	1.8003800
H	-4.5947680	-0.8737570	0.1473120
H	-2.7645540	-3.8921980	-2.2899110
H	-0.5403320	-3.0824290	-1.6638680
H	-5.3674780	-2.8499990	-2.5938560
H	-6.0166870	-2.4115060	-1.0001560
H	-5.3410650	-4.0317530	-1.2737210
H	-4.4741380	0.9254850	1.4664770
H	-1.2480860	-1.0343530	2.5952050
H	-0.8670840	0.2010630	4.7144560
H	-1.6203840	1.6601110	4.0383130
H	-0.1213360	1.0286040	3.3324960
H	-3.8479120	0.1522620	3.7440630
H	-2.9617370	-1.2536470	4.3631480
H	-3.7274680	-1.3340790	2.7637450
H	-3.5053820	2.3679650	3.2085020
H	-4.4282190	3.3874830	2.0920580
H	-2.6529930	3.3520470	1.9941940
H	-4.8982750	2.6941930	-0.2345240
H	-4.0487600	1.3214270	-0.9827420
H	-3.1546080	2.8040000	-0.5862490
H	-0.5203760	1.8495940	-5.0288920

H	0.6136130	2.6382130	-3.9069780
H	-1.1104400	3.0801870	-3.8843860
H	-2.8408880	1.5027330	-2.9548970
H	-2.3108990	0.3079230	-4.1611780
H	-2.3890080	-0.1436700	-2.4404810
H	-0.0108350	-0.5360900	-4.2024010
H	0.2249600	-0.7923240	-2.4524340
H	1.2969880	0.3045680	-3.3516830
C	0.2158720	3.0609170	0.9381330
H	-0.8332800	2.7645460	-1.5236260
H	1.0021530	3.6569600	0.4399330
H	0.4395950	3.0643330	2.0241400
H	-0.7265970	3.6262590	0.8132820

6’

Symbol	X	Y	Z
C	1.2123370	-3.2473570	-0.3746520
C	1.1811540	-1.8295060	-0.3011820
C	2.4415600	-1.1810500	-0.1975090
C	2.4026410	-3.9526750	-0.3946360
C	3.6499560	-3.3107710	-0.3268400
C	3.6313090	-1.9246040	-0.2073610
N	0.0140430	-1.0968680	-0.2349440
P	2.4065420	0.6114970	0.1378330
C	3.4723670	0.7308810	1.6654560
C	3.8340500	2.1599130	2.0683070
C	2.7612700	0.0064260	2.8104840
C	3.2651170	1.4869380	-1.2587930
C	4.7078500	1.0666400	-1.5268870

C	3.1317800	3.0065440	-1.1210390
C	4.9352180	-4.0952270	-0.3571410
C	-3.6233980	-1.5271730	-0.7511530
C	-2.3959570	-1.1172970	-0.2326130
C	-1.1710730	-1.5742500	-0.7847230
C	-3.7054610	-2.3863340	-1.8512240
C	-2.4975180	-2.8001440	-2.4255710
C	-1.2638120	-2.4070080	-1.9184030
C	-5.0309140	-2.8570890	-2.3903320
P	-2.1448420	0.0507540	1.1375560
C	-3.7488500	0.9188820	1.4537870
C	-1.8562460	-1.1137140	2.5731340
C	-1.0951480	-0.4632010	3.7278520
C	-3.1125050	-1.8521300	3.0275740
C	-3.6783100	1.7349830	2.7471130
C	-4.1165240	1.8092840	0.2690350
Cr	0.0004880	0.9521570	0.3607840
C	-0.3962960	1.5436910	-1.3610180
C	-0.7901700	2.7741550	-2.1527440
C	-0.8933130	4.0422450	-1.3015530
C	-2.1501670	2.4952360	-2.8233370
C	0.2415050	2.9933080	-3.2773080
C	0.0749290	2.6261640	1.5737470
H	0.2718950	-3.7996560	-0.3901590
H	2.3656020	-5.0455150	-0.4457680
H	4.5864950	-1.4032190	-0.0996190
H	4.4031400	0.1861760	1.4359720
H	4.5361110	2.6269650	1.3648910
H	4.3235680	2.1509120	3.0552340
H	2.9422250	2.7999010	2.1457760
H	1.8823130	0.5804230	3.1425030
H	3.4357860	-0.0910760	3.6756580

H	2.4328980	-1.0055500	2.5287270
H	2.6393430	1.1738040	-2.1124640
H	5.1145630	1.6524700	-2.3669290
H	4.7860380	0.0057640	-1.8008650
H	5.3581470	1.2521930	-0.6567050
H	3.8386240	3.4178710	-0.3877130
H	2.1169320	3.2989560	-0.8164440
H	3.3531420	3.4896320	-2.0857350
H	4.9522830	-4.8743680	0.4217510
H	5.8070650	-3.4443480	-0.1963360
H	5.0763820	-4.6041740	-1.3247010
H	-4.5499680	-1.1637000	-0.2962220
H	-2.5201500	-3.4428470	-3.3110180
H	-0.3528960	-2.7360770	-2.4208850
H	-5.0489740	-2.8388490	-3.4909780
H	-5.8589020	-2.2283460	-2.0312720
H	-5.2458650	-3.8934020	-2.0802650
H	-4.5216940	0.1400760	1.5703650
H	-1.1766090	-1.8461150	2.1072650
H	-0.8295250	-1.2234260	4.4798550
H	-1.6793480	0.3168130	4.2373560
H	-0.1596010	-0.0027920	3.3807590
H	-3.8225190	-1.1838850	3.5399000
H	-2.8451890	-2.6497560	3.7391670
H	-3.6338530	-2.3269930	2.1823420
H	-3.5907290	1.1015250	3.6404540
H	-4.5972720	2.3315340	2.8597470
H	-2.8256560	2.4321660	2.7372590
H	-5.0904230	2.2921980	0.4471780
H	-4.1881870	1.2430320	-0.6700570
H	-3.3657330	2.6018980	0.1315640
H	-1.1816830	4.9076900	-1.9209110

H	0.0632830	4.2776830	-0.8125690
H	-1.6458350	3.9281550	-0.5074180
H	-2.9405120	2.3423260	-2.0778320
H	-2.4474560	3.3416620	-3.4651530
H	-2.1058180	1.5917150	-3.4519500
H	-0.0649300	3.8313360	-3.9256770
H	0.3346010	2.0965370	-3.9106110
H	1.2350310	3.2304910	-2.8754890
H	-0.8824220	3.1778880	1.5829050
H	0.2654410	2.3177960	2.6234750
H	0.8597060	3.3578630	1.3096410
H	-0.3486670	0.6666360	-2.0358630

6’-7’-TS

Symbol	X	Y	Z
C	-1.0134710	-2.9169370	1.4201660
C	-1.0857110	-1.5962910	0.8983690
C	-2.3679250	-1.1705160	0.4454330
C	-2.1302210	-3.7331680	1.4832960
C	-3.3939360	-3.3150430	1.0341990
C	-3.4751520	-2.0251910	0.5138720
N	-0.0108020	-0.7669310	0.7375200
P	-2.4198300	0.5102950	-0.2442690
C	-3.5206230	0.3953450	-1.7367650
C	-3.8321170	1.7691330	-2.3294080
C	-2.8509970	-0.5060290	-2.7745340
C	-3.2848200	1.5391780	1.0461060
C	-4.7371540	1.1498320	1.3080280
C	-3.1242640	3.0377760	0.7933380

C	-4.5912630	-4.2260940	1.1118570
C	3.6486760	-1.1770310	0.9236200
C	2.3637750	-0.9591710	0.4307140
C	1.2360220	-1.0675820	1.2805020
C	3.8729770	-1.5068120	2.2665530
C	2.7559830	-1.6010610	3.1034330
C	1.4643330	-1.3831270	2.6295200
C	5.2676450	-1.7653930	2.7744420
P	1.9103020	-0.5133710	-1.2874530
C	3.4809420	0.1169930	-2.0508420
C	1.5425460	-2.1938440	-2.0124620
C	0.7046500	-2.1056630	-3.2875380
C	2.7705360	-3.0867200	-2.1666980
C	3.3394710	0.2769860	-3.5658110
C	3.8587680	1.4469300	-1.3918550
Cr	-0.0612000	0.9553430	-0.5388700
C	0.6122320	2.2248500	0.7858450
C	0.8655520	3.6154150	1.3428070
C	0.3979940	4.7452060	0.4179630
C	2.3805730	3.7731510	1.5796060
C	0.1368240	3.7191720	2.6951860
C	0.0713870	2.2984840	-1.9193360
H	-0.0528410	-3.3066650	1.7596150
H	-2.0167530	-4.7452060	1.8854770
H	-4.4420300	-1.6773800	0.1376380
H	-4.4653480	-0.0722420	-1.4125060
H	-4.4809610	2.3698310	-1.6772530
H	-4.3564830	1.6508360	-3.2910370
H	-2.9077160	2.3382170	-2.5190370
H	-1.9293510	-0.0324380	-3.1500730
H	-3.5236620	-0.6647450	-3.6323890
H	-2.5922030	-1.4927000	-2.3620280

H	-2.6848660	1.2876950	1.9379890
H	-5.1463630	1.7602100	2.1291980
H	-4.8365460	0.0955470	1.6022750
H	-5.3720160	1.3261240	0.4246890
H	-3.7879120	3.3998670	-0.0049560
H	-2.0900830	3.2764480	0.5124060
H	-3.3721880	3.6048740	1.7046770
H	-4.4247130	-5.1665070	0.5614860
H	-5.4867640	-3.7488500	0.6869670
H	-4.8263710	-4.4998340	2.1535510
H	4.5104780	-1.0889570	0.2550030
H	2.8992190	-1.8389120	4.1616070
H	0.6151240	-1.4421520	3.3137620
H	5.3073120	-1.7411800	3.8731210
H	5.9817400	-1.0181330	2.3956720
H	5.6335920	-2.7552220	2.4541610
H	4.2778070	-0.6200690	-1.8536870
H	0.8981840	-2.6228270	-1.2259400
H	0.3670730	-3.1102920	-3.5889090
H	1.2672520	-1.6803620	-4.1322260
H	-0.1891280	-1.4855130	-3.1319960
H	3.4476370	-2.7212710	-2.9551560
H	2.4656390	-4.1078970	-2.4466940
H	3.3425550	-3.1591810	-1.2290740
H	3.2255260	-0.6880650	-4.0794320
H	4.2380470	0.7627580	-3.9784480
H	2.4729660	0.9050850	-3.8276920
H	4.8380650	1.7906340	-1.7619130
H	3.9208200	1.3691310	-0.2963800
H	3.1166750	2.2248870	-1.6296620
H	0.5819920	5.7267530	0.8834980
H	-0.6774050	4.6782350	0.1976400

H	0.9385860	4.7261160	-0.5415460
H	2.9367990	3.7014470	0.6316880
H	2.6084050	4.7529710	2.0316800
H	2.7626320	2.9902460	2.2537340
H	0.3398760	4.6903470	3.1766100
H	0.4656470	2.9245540	3.3834180
H	-0.9517790	3.6242600	2.5685980
H	0.8017270	2.4212210	-0.6192050
H	0.7585290	2.1948030	-2.7761330
H	-0.4612570	3.2625460	-1.9710060
H	1.0627790	1.4807110	1.4666170

6’-7”-TS

Symbol	X	Y	Z
C	1.2489680	-2.7659170	-1.3558330
C	1.2353300	-1.4170630	-0.9107790
C	2.4850740	-0.8852980	-0.4780880
C	2.4159210	-3.5139490	-1.3683960
C	3.6465910	-2.9913420	-0.9432670
C	3.6440150	-1.6677840	-0.5031720
N	0.1081490	-0.6459790	-0.8076690
P	2.4248550	0.8428560	0.0904290
C	3.7993660	1.0117240	1.3289900
C	4.0767570	2.4718130	1.6890350
C	3.4469230	0.1955900	2.5731390
C	2.9128690	1.7972180	-1.4420580
C	4.3536910	1.5617070	-1.8876410
C	2.5594040	3.2830140	-1.3647390
C	4.9014170	-3.8252540	-0.9642290

C	-3.4537630	-1.6137910	-0.6887750
C	-2.1830740	-1.1877300	-0.3136180
C	-1.1275600	-1.1255930	-1.2544090
C	-3.7330520	-1.9852170	-2.0114380
C	-2.6890570	-1.9151230	-2.9391360
C	-1.4100170	-1.4940580	-2.5759980
C	-5.1196750	-2.4197140	-2.4093810
P	-1.6331040	-0.6314500	1.3362280
C	-3.1560380	-0.3435840	2.3495290
C	-0.8085490	-2.1685890	2.0023950
C	0.1881010	-1.8583740	3.1180830
C	-1.7832300	-3.2874840	2.3589480
C	-2.7900500	-0.1042740	3.8164370
C	-3.9442320	0.8389690	1.7906530
Cr	-0.0478000	1.0356650	0.5300730
C	-1.0225550	2.1288680	-0.7608010
C	-2.0341600	3.1848710	-1.1613720
C	-1.3910240	4.1239280	-2.1993940
C	-2.5314750	4.0252540	0.0188150
C	-3.2226730	2.4541530	-1.8137410
C	0.0011150	2.5082840	1.7860750
H	0.3167170	-3.2328750	-1.6768960
H	2.3671310	-4.5530090	-1.7099460
H	4.5873070	-1.2356980	-0.1552720
H	4.7123040	0.5927180	0.8742370
H	4.4756560	3.0436730	0.8398880
H	4.8256100	2.5197930	2.4956580
H	3.1699880	2.9816230	2.0494840
H	2.5429740	0.6000590	3.0569580
H	4.2700880	0.2375380	3.3041840
H	3.2641110	-0.8627100	2.3348460
H	2.2398790	1.3355730	-2.1859440

H	4.5310370	2.0361430	-2.8662700
H	4.5786350	0.4903710	-1.9955140
H	5.0763940	1.9970960	-1.1790150
H	3.2214140	3.8356010	-0.6818520
H	1.5236950	3.4333050	-1.0275980
H	2.6574080	3.7453460	-2.3601090
H	4.7719260	-4.7683640	-0.4089730
H	5.7476240	-3.2880740	-0.5106620
H	5.1951960	-4.0949140	-1.9921940
H	-4.2563190	-1.6611640	0.0540960
H	-2.8808650	-2.1911120	-3.9801760
H	-0.6167880	-1.4410900	-3.3252120
H	-5.1311880	-2.8559870	-3.4184630
H	-5.8225040	-1.5704260	-2.4085440
H	-5.5217880	-3.1725480	-1.7136330
H	-3.7733330	-1.2555300	2.2806410
H	-0.2226710	-2.4938640	1.1275730
H	0.8000620	-2.7480970	3.3361960
H	-0.3059090	-1.5575790	4.0540300
H	0.8689790	-1.0468860	2.8245560
H	-2.3787460	-3.0482710	3.2541280
H	-1.2294350	-4.2152250	2.5744150
H	-2.4764770	-3.5030830	1.5311710
H	-2.3830910	-1.0033030	4.2998880
H	-3.6861700	0.1948270	4.3828750
H	-2.0451150	0.7021880	3.9138490
H	-4.8855880	0.9668910	2.3482320
H	-4.1950770	0.7146750	0.7280090
H	-3.3615030	1.7660340	1.8912800
H	-2.1166330	4.8749520	-2.5538380
H	-1.0306110	3.5624690	-3.0759090
H	-0.5328300	4.6636260	-1.7681400

H	-1.7064230	4.5841670	0.4883810
H	-3.2838740	4.7578500	-0.3147750
H	-2.9942600	3.3969790	0.7922450
H	-3.9773120	3.1741140	-2.1722850
H	-3.7070740	1.7750420	-1.0972020
H	-2.8949810	1.8468740	-2.6721270
H	-0.3242710	2.7142990	0.3472930
H	0.9050030	3.0833360	2.0467630
H	-0.8786280	2.9325550	2.2997650
H	-0.6079340	1.6433400	-1.6630100

7’

Symbol	X	Y	Z
C	-0.6990680	-3.3271580	-1.0620580
C	-0.8744370	-2.0722670	-0.4395780
C	-2.1982080	-1.5901690	-0.3226950
C	-1.7863520	-4.0704550	-1.4991870
C	-3.1031000	-3.6133110	-1.3506400
C	-3.2786180	-2.3594730	-0.7620590
N	0.1622660	-1.2620580	-0.0021200
P	-2.3022510	0.0625040	0.4546370
C	-4.0042180	0.6535230	-0.0109840
C	-4.4657640	1.8967760	0.7495700
C	-4.1216330	0.8532410	-1.5239240
C	-2.3459570	-0.4141360	2.2659900
C	-3.6469120	-1.0811060	2.7048340
C	-1.9184050	0.7126110	3.2054820
C	-4.2720930	-4.4426060	-1.8147030
C	3.7482150	-1.6597740	0.8354230

C	2.5556310	-1.1284800	0.3285740
C	1.3341790	-1.8292120	0.4690380
C	3.7949930	-2.8871820	1.4935420
C	2.5789190	-3.5720460	1.6457580
C	1.3855500	-3.0657030	1.1570380
C	5.0802890	-3.4503680	2.0411310
P	2.4064260	0.4897840	-0.4983650
C	3.7206410	1.5193380	0.3299010
C	2.9622810	0.1600950	-2.2483550
C	2.6239860	1.2973810	-3.2126870
C	4.4201470	-0.2771140	-2.3658620
C	3.9227980	2.8861870	-0.3258860
C	3.4258550	1.6554090	1.8260610
Cr	-0.0482510	0.8269360	-0.2634880
C	0.2521350	2.8900470	-0.0196390
C	-0.7594710	4.0367590	0.1587640
C	-1.7614690	4.0438360	-0.9989650
C	-0.0222520	5.3880290	0.1751230
C	-1.4996870	3.8792560	1.4895380
C	-0.5631570	0.8083870	-2.0412680
H	0.3104550	-3.7145250	-1.2109460
H	-1.6068480	-5.0357240	-1.9826170
H	-4.2974610	-1.9779910	-0.6537800
H	-4.6703560	-0.1746540	0.2826550
H	-4.3644820	1.7912460	1.8390880
H	-5.5302800	2.0813950	0.5346640
H	-3.9095630	2.7919260	0.4422700
H	-3.4862900	1.6810750	-1.8697260
H	-5.1631690	1.0984570	-1.7863800
H	-3.8323430	-0.0423770	-2.0912130
H	-1.5456440	-1.1734760	2.2859840
H	-3.5342090	-1.4982480	3.7184410

H	-3.9270800	-1.9107060	2.0381760
H	-4.4841170	-0.3662800	2.7383830
H	-2.6627360	1.5213630	3.2589950
H	-0.9612070	1.1598820	2.8966580
H	-1.7877870	0.3184260	4.2259760
H	-4.1882380	-4.6990070	-2.8829630
H	-5.2239750	-3.9095950	-1.6752180
H	-4.3390270	-5.3922710	-1.2592670
H	4.6782120	-1.0947160	0.7235780
H	2.5669990	-4.5279900	2.1786350
H	0.4619680	-3.6242820	1.3191690
H	5.0267820	-3.5929640	3.1327330
H	5.9310370	-2.7845950	1.8350850
H	5.3132050	-4.4326720	1.5995920
H	4.6597880	0.9519110	0.2176020
H	2.3132780	-0.6955560	-2.5046800
H	2.7866050	0.9632730	-4.2498100
H	3.2557270	2.1830680	-3.0519090
H	1.5728030	1.6043190	-3.1194220
H	5.1129020	0.5406310	-2.1097600
H	4.6398790	-0.5741270	-3.4037370
H	4.6484080	-1.1392330	-1.7224700
H	4.2876240	2.8023810	-1.3588810
H	4.6771150	3.4566760	0.2392500
H	2.9952490	3.4764800	-0.3357660
H	4.2584020	2.1766230	2.3245810
H	3.3005770	0.6799480	2.3182170
H	2.5145710	2.2461560	2.0036560
H	-2.5083210	4.8496720	-0.8942810
H	-2.2921900	3.0840190	-1.0544730
H	-1.2472530	4.1892520	-1.9633610
H	0.5157270	5.5561250	-0.7723570

H	-0.7163730	6.2341810	0.3211100
H	0.7212580	5.4213000	0.9887770
H	-2.2471670	4.6759460	1.6440460
H	-0.7958130	3.9140170	2.3377950
H	-2.0192710	2.9140190	1.5329320
H	0.8774070	3.1308990	-0.9029720
H	-0.7769220	1.7159480	-2.6271750
H	-0.7140810	-0.1303150	-2.6020440
H	0.9568570	2.9424360	0.8379580

7”

Symbol	X	Y	Z
C	-1.6375590	-3.0578480	-0.7202560
C	-1.4956720	-1.7204200	-0.2976810
C	-2.6624890	-0.9214970	-0.2805020
C	-2.8760170	-3.5554330	-1.1055810
C	-4.0332640	-2.7630990	-1.0965280
C	-3.8977040	-1.4383310	-0.6734880
N	-0.2883560	-1.1271220	0.0444600
P	-2.3252280	0.7510920	0.3502340
C	-3.7375700	1.8138780	-0.2065140
C	-3.7322350	3.1942700	0.4534000
C	-3.7541500	1.9388880	-1.7306530
C	-2.4872840	0.4955700	2.1943140
C	-3.9245750	0.3037150	2.6709230
C	-1.7202070	1.5397330	3.0064540
C	-5.3579580	-3.3188750	-1.5501080
C	3.0394120	-2.1445110	1.2760700
C	2.0396920	-1.4908540	0.5493310

C	0.6790380	-1.8412270	0.7301720
C	2.7511420	-3.1557570	2.1951030
C	1.4035530	-3.5036870	2.3617440
C	0.3918440	-2.8694860	1.6551330
C	3.8353840	-3.8417600	2.9847670
P	2.2990940	-0.1850260	-0.7109990
C	4.1170020	0.2149950	-0.5485340
C	2.1389350	-1.1736690	-2.2947850
C	2.1183430	-0.3120700	-3.5593020
C	3.1265860	-2.3321860	-2.4064420
C	4.7025100	0.9462770	-1.7590560
C	4.4184490	0.9706490	0.7476640
Cr	-0.0282800	0.9397930	-0.3550270
C	-0.0100540	3.0235390	-0.0297070
C	1.2477920	3.8017460	0.3970450
C	0.9089520	5.2828370	0.6376390
C	2.3082320	3.7206080	-0.7041440
C	1.7825030	3.2114800	1.7092340
C	-0.5594270	0.9655680	-2.1316380
H	-0.7589160	-3.7054370	-0.7559300
H	-2.9459280	-4.5957500	-1.4379340
H	-4.7846990	-0.7976470	-0.6545070
H	-4.6497330	1.2824210	0.1153860
H	-3.7844130	3.1406520	1.5499670
H	-4.6084370	3.7681950	0.1123480
H	-2.8322550	3.7655620	0.1820950
H	-2.8858190	2.5138580	-2.0846170
H	-4.6656250	2.4687250	-2.0506100
H	-3.7359070	0.9617790	-2.2346250
H	-1.9560830	-0.4639700	2.3148910
H	-3.9327700	-0.0135020	3.7260030
H	-4.4459020	-0.4728020	2.0904460

H	-4.5090030	1.2346380	2.6055420
H	-2.1389160	2.5518200	2.8998030
H	-0.6626620	1.5849200	2.7067810
H	-1.7509080	1.2797030	4.0766480
H	-5.3657360	-3.5037230	-2.6370060
H	-6.1833030	-2.6268860	-1.3274100
H	-5.5838660	-4.2780000	-1.0584420
H	4.0869710	-1.8696090	1.1286830
H	1.1371450	-4.2877550	3.0773330
H	-0.6469490	-3.1597790	1.8250130
H	3.8090370	-4.9343290	2.8454850
H	3.7301510	-3.6512860	4.0653360
H	4.8339290	-3.4926320	2.6836190
H	4.6116850	-0.7689430	-0.4973050
H	1.1259440	-1.5878030	-2.1600200
H	1.6660100	-0.8797740	-4.3879410
H	3.1278970	-0.0194840	-3.8809500
H	1.5246980	0.6031400	-3.4213230
H	4.1605470	-1.9786290	-2.5489710
H	2.8753360	-2.9556730	-3.2797090
H	3.1041700	-2.9813530	-1.5185730
H	4.7487880	0.3041000	-2.6482070
H	5.7344520	1.2576750	-1.5312380
H	4.1368930	1.8524780	-2.0214300
H	5.5039420	0.9669440	0.9365060
H	3.9282330	0.5247790	1.6257070
H	4.0988970	2.0189120	0.6778020
H	1.7957580	5.8622430	0.9494690
H	0.1450500	5.3912160	1.4252750
H	0.5100520	5.7491600	-0.2779950
H	1.9407270	4.1881590	-1.6325030
H	3.2461230	4.2282660	-0.4201760

H	2.5352780	2.6725720	-0.9387830
H	2.7178390	3.6973280	2.0359930
H	1.9834420	2.1322180	1.6118500
H	1.0448090	3.3386860	2.5190060
H	-0.4258780	3.4899410	-0.9443370
H	-0.5719200	1.8721900	-2.7567060
H	-0.9144520	0.0610840	-2.6551390
H	-0.7737190	3.2069910	0.7518570

Cr^{VI} original ligand (L_0)

1

Symbol	X	Y	Z
C	-0.5692410	-3.4278080	-0.7011000
C	-0.8758910	-2.1319530	-0.2657370
C	-2.2072900	-1.7255530	-0.1594740
C	-1.6020400	-4.3074540	-0.9948260
C	-2.9481840	-3.9338260	-0.8636680
C	-3.2315200	-2.6260850	-0.4524130
N	0.1085590	-1.1676150	0.0558830
P	-2.3786680	0.0323940	0.2823280
C	-3.8235400	0.5253400	-0.7913680
C	-4.4286920	1.9000690	-0.5166200
C	-3.5747200	0.2856740	-2.2835550
C	-2.9666050	0.0319740	2.0467700

C	-4.3544240	-0.5839290	2.2192780
C	-2.8680690	1.4010950	2.7150520
C	-4.0447890	-4.9169600	-1.1548610
C	3.7907360	-1.5377000	0.3035260
C	2.5255750	-1.0960810	-0.0724590
C	1.3751920	-1.6692040	0.4889080
C	3.9359850	-2.5520140	1.2575290
C	2.7730070	-3.1040740	1.8207110
C	1.5045150	-2.6832690	1.4431530
C	5.2915540	-3.0357920	1.6843120
P	2.0998570	0.2418270	-1.2177140
C	3.5920850	1.2850750	-1.5208430
C	1.6476090	-0.6751130	-2.7794060
C	0.6581650	0.0780690	-3.6707750
C	2.8779770	-1.1911570	-3.5247920
C	3.3842220	2.2580990	-2.6828020
C	4.0478550	2.0036010	-0.2538140
Cr	0.0187350	0.6599180	-0.0315570
C	0.5662250	1.1760790	1.4707220
C	1.0499000	1.1493910	2.8871010
C	0.7837280	2.5121780	3.5488380
C	2.5658320	0.8731950	2.8858820
C	0.3327020	0.0262490	3.6526200
C	-0.8501540	2.2120790	-1.0287470
C	-0.7618740	3.7059550	-0.6462230
C	0.6805130	4.2075630	-0.5207910
C	-1.4261050	4.4557080	-1.8205400
C	-1.5235460	4.0197590	0.6460580
H	0.4676260	-3.7451830	-0.8189140
H	-1.3585790	-5.3149950	-1.3401560
H	-4.2770410	-2.3179310	-0.3833770
H	-4.5689550	-0.2157980	-0.4586850

H	-4.7024150	2.0352840	0.5380550
H	-5.3521900	2.0007180	-1.1063270
H	-3.7679450	2.7261290	-0.8125900
H	-2.9313010	1.0544180	-2.7365750
H	-4.5368010	0.3305640	-2.8150480
H	-3.1337500	-0.6999120	-2.4907480
H	-2.2251330	-0.6373330	2.5156540
H	-4.5766730	-0.6617920	3.2942300
H	-4.4257150	-1.5976450	1.8018340
H	-5.1402660	0.0426230	1.7714940
H	-3.6381350	2.0963950	2.3536940
H	-1.8901510	1.8695560	2.5534140
H	-3.0155480	1.2901940	3.7998710
H	-4.0666150	-5.7060970	-0.3867270
H	-3.8864920	-5.4135390	-2.1234770
H	-5.0314650	-4.4355340	-1.1711450
H	4.6823440	-1.0884320	-0.1393370
H	2.8677370	-3.8861480	2.5778850
H	0.6177740	-3.1304630	1.8957440
H	6.0968030	-2.5229250	1.1419350
H	5.3956740	-4.1171410	1.5086320
H	5.4436830	-2.8667200	2.7614240
H	4.3654730	0.5531680	-1.8142910
H	1.1161060	-1.5502150	-2.3712990
H	0.4420980	-0.5259290	-4.5645280
H	1.0415640	1.0487260	-4.0178590
H	-0.3016070	0.2476180	-3.1602510
H	3.4462930	-0.3850340	-4.0103360
H	2.5560180	-1.8847160	-4.3158430
H	3.5575400	-1.7460140	-2.8601970
H	3.1515760	1.7523360	-3.6287130
H	4.3097390	2.8314680	-2.8375870

H	2.5845020	2.9810260	-2.4706950
H	4.9548560	2.5857320	-0.4736870
H	4.2915380	1.3061380	0.5581640
H	3.2823210	2.7089990	0.1041840
H	1.1607650	2.4913140	4.5816950
H	-0.2888160	2.7489270	3.5929510
H	1.3023340	3.3272160	3.0217910
H	3.1168000	1.6584610	2.3526140
H	2.9172620	0.8672740	3.9280190
H	2.8013360	-0.1018180	2.4396700
H	0.7773430	-0.0648850	4.6543740
H	0.4451030	-0.9408430	3.1442980
H	-0.7341390	0.2432680	3.7823870
H	0.3695820	2.1823390	0.9680980
H	-0.1009770	1.9642140	-1.8155250
H	-1.8352060	2.0000730	-1.4414660
H	0.6852680	5.2813830	-0.2847110
H	1.2317720	4.0790550	-1.4630390
H	1.2512760	3.7105100	0.2804060
H	-1.3525340	5.5426260	-1.6665800
H	-2.4930540	4.2028250	-1.9119160
H	-0.9363070	4.2192920	-2.7778780
H	-1.0067950	3.6228640	1.5333600
H	-2.5428800	3.6126750	0.6347550
H	-1.6001770	5.1085260	0.7819640

1-2-TS

Symbol	X	Y	Z
C	-0.8657310	-3.4938280	-0.5207750

C	-1.0561110	-2.1533440	-0.1598360
C	-2.3543030	-1.6320830	-0.0867770
C	-1.9684020	-4.2975160	-0.7756050
C	-3.2787930	-3.8052690	-0.6778950
C	-3.4495600	-2.4574790	-0.3401600
N	0.0084900	-1.2683170	0.1206560
P	-2.4019610	0.1511450	0.2768740
C	-3.7145700	0.7470300	-0.9044130
C	-4.2432660	2.1601130	-0.6594280
C	-3.2812710	0.5279100	-2.3560160
C	-3.0270670	0.3015780	2.0160590
C	-4.4591060	-0.1992270	2.1950420
C	-2.8258540	1.7071680	2.5811740
C	-4.4544010	-4.7060090	-0.9253520
C	3.6604490	-1.8149380	0.3266240
C	2.4089300	-1.3349870	-0.0481540
C	1.2411620	-1.8314710	0.5570020
C	3.7766990	-2.7895780	1.3254080
C	2.6001410	-3.2699420	1.9254320
C	1.3437780	-2.8127140	1.5486690
C	5.1173080	-3.3057270	1.7614650
P	2.0220730	-0.0271920	-1.2457700
C	3.5402420	0.9387370	-1.6428070
C	1.4662680	-0.9635800	-2.7553320
C	0.4814570	-0.1649440	-3.6091790
C	2.6324830	-1.5536690	-3.5457620
C	3.2934190	1.9029810	-2.8062900
C	4.0994430	1.6478840	-0.4130830
Cr	0.0234680	0.5755180	-0.0505140
C	0.6934190	1.1020900	1.3406090
C	1.2004140	1.2639100	2.7321340
C	0.9944030	2.6889010	3.2669210

C	2.6968030	0.9046800	2.7690200
C	0.4143290	0.2547210	3.5967480
C	-0.5680850	2.4545280	-0.8606960
C	-0.2802450	3.9421330	-0.5837580
C	1.2021260	4.2170180	-0.3294510
C	-0.7211760	4.6916210	-1.8545990
C	-1.1166470	4.4244480	0.6052410
H	0.1401660	-3.9040660	-0.6156280
H	-1.8096750	-5.3398310	-1.0621830
H	-4.4645320	-2.0567640	-0.2863280
H	-4.5456970	0.0532240	-0.6956160
H	-4.6851870	2.2742460	0.3393010
H	-5.0381800	2.3692170	-1.3907760
H	-3.4779650	2.9389410	-0.7856470
H	-2.4935600	1.2306430	-2.6691300
H	-4.1408830	0.6979160	-3.0207700
H	-2.9267570	-0.4975320	-2.5380990
H	-2.3477750	-0.3864890	2.5480860
H	-4.7258500	-0.1556040	3.2617380
H	-4.5809160	-1.2421950	1.8724330
H	-5.1835450	0.4256660	1.6515710
H	-3.5418050	2.4292060	2.1650520
H	-1.8141440	2.0855520	2.3860420
H	-2.9769370	1.6932700	3.6709750
H	-4.3582610	-5.2268060	-1.8895490
H	-5.4009720	-4.1494240	-0.9273130
H	-4.5176940	-5.4805530	-0.1450420
H	4.5626190	-1.4281230	-0.1536300
H	2.6752830	-4.0227800	2.7139230
H	0.4469820	-3.2004770	2.0353220
H	5.9294650	-2.9104150	1.1372210
H	5.1511050	-4.4043190	1.7153690

H	5.3206370	-3.0201960	2.8054490
H	4.2667190	0.1753350	-1.9709840
H	0.9105660	-1.8029500	-2.3061050
H	0.1367870	-0.7852720	-4.4500490
H	0.9263470	0.7453400	-4.0363180
H	-0.4172960	0.1265810	-3.0426540
H	3.2145220	-0.7838950	-4.0727220
H	2.2433420	-2.2476710	-4.3056950
H	3.3157470	-2.1255990	-2.8996020
H	3.0597130	1.3822350	-3.7442680
H	4.2025500	2.4966830	-2.9808060
H	2.4773720	2.6087960	-2.5918330
H	5.0113080	2.1959930	-0.6924050
H	4.3673170	0.9450490	0.3862250
H	3.3832940	2.3790300	-0.0105080
H	1.4081970	2.7394090	4.2844630
H	-0.0677730	2.9595360	3.3330640
H	1.5177680	3.4397860	2.6592980
H	3.3010230	1.6475170	2.2335570
H	3.0248690	0.8984780	3.8185020
H	2.8868220	-0.0915870	2.3467450
H	0.8208450	0.2770970	4.6183160
H	0.5139080	-0.7697360	3.2119080
H	-0.6496930	0.5160310	3.6511210
H	0.1222840	2.0786740	0.5928410
H	0.0525200	2.0926860	-1.7096620
H	-1.6207770	2.3495620	-1.1213930
H	1.3671150	5.2897150	-0.1526590
H	1.8224710	3.9265350	-1.1894600
H	1.5789580	3.6840980	0.5568270
H	-0.5550570	5.7725950	-1.7336870
H	-1.7916310	4.5397290	-2.0626900

H	-0.1517550	4.3634450	-2.7381420
H	-0.8315780	3.9053690	1.5320630
H	-2.1915340	4.2575650	0.4400610
H	-0.9676500	5.5011360	0.7719920

A

Symbol	X	Y	Z
C	0.7757660	-2.6056370	-1.4467610
C	0.9890610	-1.3140110	-0.9365830
C	2.2871950	-0.9281830	-0.5184550
C	1.8333150	-3.4932900	-1.5409020
C	3.1274020	-3.1434840	-1.1162880
C	3.3243280	-1.8631070	-0.5916450
N	-0.0667590	-0.4004580	-0.7406520
P	2.5144160	0.7619340	0.1769330
C	3.7291270	0.5635790	1.5580410
C	4.1447460	1.9153160	2.1427470
C	3.1283880	-0.3513360	2.6266200
C	3.1895610	1.8200440	-1.1800020
C	4.5735410	1.3657310	-1.6401680
C	3.1513290	3.3091990	-0.8256230
C	4.2601740	-4.1174280	-1.2473750
C	-3.7217030	-0.7474720	-0.8831220
C	-2.4173040	-0.6104290	-0.4198980
C	-1.3453020	-0.5773360	-1.3336640
C	-3.9799220	-0.8427840	-2.2578910
C	-2.8928900	-0.8142160	-3.1469740
C	-1.5798920	-0.6945830	-2.7020640
C	-5.3835550	-0.9586760	-2.7786920

P	-1.8428850	-0.4720150	1.3106270
C	-3.1871490	0.1393530	2.4119210
C	-1.4343150	-2.2311830	1.7672080
C	-0.3400640	-2.3299810	2.8296870
C	-2.6779340	-3.0495380	2.1058760
C	-2.7057030	0.1624010	3.8650650
C	-3.7246230	1.4972550	1.9747290
Cr	0.1690360	0.6501130	0.6802410
C	-0.6063300	1.9248720	0.0667530
C	-1.1373510	3.0152760	-0.7783170
C	-0.9106440	4.3383680	-0.0188110
C	-2.6296810	2.8380950	-1.1061150
C	-0.3313790	2.9916590	-2.0949700
H	-0.2251380	-2.9122690	-1.7540730
H	1.6563690	-4.4927530	-1.9450440
H	4.3273060	-1.5855220	-0.2603610
H	4.6199650	0.0780730	1.1275270
H	4.7659000	2.4968880	1.4498660
H	4.7435490	1.7445500	3.0494080
H	3.2757250	2.5284620	2.4306170
H	2.2470690	0.1137140	3.1018520
H	3.8637920	-0.5217850	3.4265300
H	2.8424750	-1.3372320	2.2315710
H	2.4634330	1.6327900	-1.9897960
H	4.8912030	1.9838210	-2.4932100
H	4.5822490	0.3184880	-1.9741740
H	5.3281010	1.4910000	-0.8486210
H	3.9833340	3.5945580	-0.1683020
H	2.2143610	3.6040760	-0.3312720
H	3.2482650	3.9034670	-1.7459750
H	4.0025600	-5.0822720	-0.7853860
H	5.1784730	-3.7432330	-0.7763770

H	4.4778480	-4.3169450	-2.3082710
H	-4.5546820	-0.7757270	-0.1760680
H	-3.0848500	-0.8850670	-4.2201780
H	-0.7501500	-0.6706250	-3.4115710
H	-6.1073820	-1.1120240	-1.9676850
H	-5.4760380	-1.7945360	-3.4873840
H	-5.6702490	-0.0432720	-3.3197920
H	-3.9912880	-0.6097790	2.3165970
H	-1.0169350	-2.6204110	0.8237460
H	-0.0646930	-3.3850340	2.9764210
H	-0.6548560	-1.9363030	3.8062840
H	0.5787930	-1.8001700	2.5312040
H	-3.1284470	-2.7379910	3.0602590
H	-2.4022900	-4.1101060	2.2046990
H	-3.4429000	-2.9820250	1.3172930
H	-2.4261310	-0.8337040	4.2330750
H	-3.5175880	0.5315790	4.5084000
H	-1.8476710	0.8407820	3.9979730
H	-4.5121200	1.8165420	2.6731390
H	-4.1677130	1.4632940	0.9711160
H	-2.9363320	2.2665650	1.9888690
H	-1.2594050	5.1707260	-0.6479470
H	0.1522280	4.5058790	0.2070900
H	-1.4781440	4.3641340	0.9228640
H	-3.2567250	2.9297670	-0.2131390
H	-2.9213230	3.6389070	-1.8011050
H	-2.8316710	1.8747270	-1.5950900
H	-0.7194400	3.7795000	-2.7570040
H	-0.4441780	2.0274040	-2.6109740
H	0.7336550	3.1899300	-1.9281820

5-6-TS

Symbol	X	Y	Z
C	1.0364560	-2.9913040	-0.7661160
C	1.1398240	-1.6112750	-0.5441740
C	2.4007500	-1.0298510	-0.3151150
C	2.1843610	-3.7706390	-0.7736430
C	3.4542280	-3.2131780	-0.5596020
C	3.5383400	-1.8347340	-0.3213590
N	0.0279340	-0.7523530	-0.4895800
P	2.3495450	0.7723620	-0.0066200
C	3.7214030	1.0547240	1.2146850
C	4.1125790	2.5201200	1.4091370
C	3.4538460	0.3416010	2.5410590
C	2.8007700	1.5674480	-1.6221210
C	4.2159490	1.2324900	-2.0872620
C	2.5314850	3.0737830	-1.6135770
C	4.6846680	-4.0711590	-0.6062640
C	-3.6077340	-1.3590300	-0.6924370
C	-2.3482870	-0.9962470	-0.2279180
C	-1.2107850	-1.1523470	-1.0515000
C	-3.7649170	-1.8800490	-1.9837470
C	-2.6250480	-2.0064430	-2.7979000
C	-1.3577430	-1.6622100	-2.3453830
C	-5.1100880	-2.3251480	-2.4772470
P	-1.9019270	-0.2720190	1.3828240
C	-3.3332860	0.4216400	2.3007000
C	-1.1424800	-1.6805740	2.3349090
C	0.1234460	-1.2024350	3.0443420
C	-2.1187710	-2.4376360	3.2297680
C	-2.8349770	1.0372540	3.6134070

C	-4.1215140	1.4402130	1.4843690
Cr	-0.0634040	0.8468890	0.4234810
C	-0.8599760	1.8543550	-0.5778740
C	-1.4941520	2.6320720	-1.6718740
C	-1.3975620	4.1346330	-1.3587200
C	-2.9639430	2.2054160	-1.8343200
C	-0.7268070	2.2758220	-2.9618030
H	0.0612430	-3.4580510	-0.9102050
H	2.0936940	-4.8463560	-0.9410420
H	4.5237350	-1.3987240	-0.1434420
H	4.5804580	0.5596250	0.7319320
H	4.5069810	2.9689440	0.4889410
H	4.9140460	2.5714700	2.1612370
H	3.2857520	3.1446160	1.7756890
H	2.6568310	0.8330260	3.1178380
H	4.3643620	0.3745120	3.1574560
H	3.1904730	-0.7173090	2.4054530
H	2.0767650	1.0937490	-2.3079700
H	4.3787420	1.6524210	-3.0911440
H	4.3906650	0.1490450	-2.1525460
H	4.9748830	1.6714190	-1.4222980
H	3.2888310	3.6286930	-1.0445890
H	1.5450050	3.3143820	-1.1926170
H	2.5587180	3.4571290	-2.6445720
H	4.9209210	-4.3449610	-1.6469930
H	4.5370750	-5.0082570	-0.0503920
H	5.5583720	-3.5533530	-0.1892480
H	-4.4830500	-1.2457430	-0.0484250
H	-2.7388730	-2.3896800	-3.8145220
H	-0.4866100	-1.7705890	-2.9943810
H	-5.2564810	-2.0618420	-3.5342570
H	-5.9257460	-1.8817230	-1.8905860

H	-5.2004390	-3.4206600	-2.4001670
H	-3.9758850	-0.4457080	2.5278890
H	-0.8301660	-2.3635970	1.5285770
H	0.6337080	-2.0510920	3.5237460
H	-0.0861580	-0.4562940	3.8256570
H	0.8422880	-0.7651750	2.3272770
H	-2.4648560	-1.8359450	4.0824930
H	-1.6163310	-3.3268700	3.6389340
H	-2.9990310	-2.7886180	2.6710640
H	-2.2557160	0.3374760	4.2313220
H	-3.6999610	1.3620890	4.2097270
H	-2.2148830	1.9258720	3.4219590
H	-4.9807610	1.7894720	2.0754730
H	-4.5092190	1.0286930	0.5431690
H	-3.5034860	2.3214690	1.2528780
H	-1.8582880	4.7030770	-2.1797500
H	-0.3543760	4.4693640	-1.2639680
H	-1.9358580	4.3899180	-0.4336010
H	-3.5721470	2.5034750	-0.9722470
H	-3.3745520	2.7058480	-2.7232880
H	-3.0547290	1.1197360	-1.9822980
H	-1.1947150	2.8003440	-3.8076030
H	-0.7747940	1.1950630	-3.1626790
H	0.3242920	2.5842790	-2.9119120
C	0.4016670	2.2901790	1.9266180
H	-0.4481530	2.4235420	0.5743060
H	1.2422100	2.9660090	1.7439890
H	0.6964610	1.5125340	2.6455470
H	-0.4295990	2.8643130	2.3604130

6

Symbol	X	Y	Z
C	-1.1734080	2.5601460	-1.7293550
C	-1.2342420	1.3583730	-1.0190020
C	-2.4578810	0.8465350	-0.5733930
C	-2.3553350	3.2376230	-1.9985710
C	-3.6001460	2.7495900	-1.5692030
C	-3.6308170	1.5495390	-0.8465010
N	-0.1068090	0.6017240	-0.6626560
P	-2.2857360	-0.7094970	0.3749400
C	-3.5720570	-0.4887650	1.7017200
C	-3.9212390	-1.7435360	2.5016720
C	-3.3092410	0.7255460	2.5965760
C	-2.8591460	-2.0558100	-0.7686920
C	-4.3030290	-1.8900750	-1.2388000
C	-2.6063350	-3.4551810	-0.2038380
C	-4.8616660	3.4993540	-1.8877850
C	3.5575090	0.7358660	-1.1400430
C	2.2962630	0.7387400	-0.5338600
C	1.1304100	0.6784470	-1.3344170
C	3.6806580	0.7247470	-2.5281370
C	2.5031060	0.6771970	-3.3100260
C	1.2474960	0.6458650	-2.7367410
C	5.0222390	0.7698150	-3.1951360
P	1.9630670	0.7878240	1.2535120
C	3.4700280	0.3346720	2.2054830
C	1.4676060	2.5560500	1.5599570
C	0.4919150	2.7321160	2.7224900
C	2.6937940	3.4646510	1.6459270
C	3.2154150	0.5066710	3.7069520

C	3.9291430	-1.0813410	1.8654700
Cr	0.1560650	-0.6063620	0.6242410
C	0.6604460	-2.1369760	0.0737370
C	1.1647730	-3.1208100	-0.9378320
C	1.0664720	-4.5452850	-0.3672990
C	2.6463760	-2.7969130	-1.2153330
C	0.3646050	-2.9967420	-2.2425200
H	-0.2154540	2.9689330	-2.0555990
H	-2.3135160	4.1803560	-2.5491770
H	-4.5963040	1.1710960	-0.5019420
H	-4.4522980	-0.2508990	1.0802050
H	-4.3489080	-2.5326570	1.8706650
H	-4.6837190	-1.4800260	3.2496880
H	-3.0649550	-2.1631570	3.0488840
H	-2.5894420	0.5017080	3.3945790
H	-4.2499620	1.0152860	3.0877290
H	-2.9569040	1.6021880	2.0325980
H	-2.1872740	-1.8986870	-1.6288500
H	-4.5372220	-2.6883090	-1.9592210
H	-4.4733550	-0.9324440	-1.7494010
H	-5.0194780	-1.9837530	-0.4082880
H	-3.3860680	-3.7539750	0.5091390
H	-1.6365850	-3.5412010	0.3050990
H	-2.6219130	-4.1909320	-1.0219540
H	-5.0434530	3.5095460	-2.9736930
H	-4.7901050	4.5477370	-1.5619010
H	-5.7367000	3.0481190	-1.4021280
H	4.4563940	0.7730660	-0.5204830
H	2.5931490	0.6576490	-4.3984500
H	0.3498050	0.5982430	-3.3557650
H	5.1442260	-0.0852470	-3.8772200
H	5.8423010	0.7510720	-2.4655900

H	5.1239460	1.6822570	-3.8025890
H	4.2462870	1.0525810	1.8913130
H	0.9272660	2.8009380	0.6304570
H	0.2708280	3.8026180	2.8463110
H	0.8928770	2.3669350	3.6789550
H	-0.4641740	2.2246980	2.5292590
H	3.2704300	3.2976900	2.5670520
H	2.3652170	4.5144280	1.6502320
H	3.3659940	3.3335290	0.7844560
H	2.9868370	1.5455060	3.9813140
H	4.1203160	0.2150390	4.2594030
H	2.3922160	-0.1335980	4.0588070
H	4.8473030	-1.3098460	2.4262110
H	4.1531690	-1.2078690	0.7980610
H	3.1736890	-1.8290090	2.1540000
H	1.4594810	-5.2601920	-1.1050190
H	0.0291700	-4.8329330	-0.1464620
H	1.6632930	-4.6511650	0.5515560
H	3.2538620	-2.8663710	-0.3026320
H	3.0364290	-3.5337580	-1.9329730
H	2.7669630	-1.7977040	-1.6525290
H	0.8350630	-3.6154630	-3.0205260
H	0.3435280	-1.9579960	-2.6026430
H	-0.6658520	-3.3532300	-2.1175370
C	-0.3177240	-0.8142360	2.6090950
H	0.6135880	-2.4288290	1.1629200
H	-1.1073910	-1.5397760	2.8266930
H	-0.5901100	0.1722990	3.0002250
H	0.6137340	-1.1440300	3.0912340

6’

Symbol	X	Y	Z
C	-1.6684120	-2.9308570	-0.9794060
C	-1.5147470	-1.6502270	-0.4280650
C	-2.6534330	-0.8634250	-0.2438420
C	-2.9382900	-3.4116190	-1.2659560
C	-4.0969300	-2.6593460	-1.0076910
C	-3.9316710	-1.3705170	-0.4997470
N	-0.2585850	-1.0987940	-0.0773400
P	-2.2536390	0.7836980	0.3591780
C	-3.5467860	1.9640400	-0.2407240
C	-3.1502020	3.4203310	-0.0081270
C	-3.9803240	1.7595090	-1.6939290
C	-2.3466210	0.6762840	2.2202590
C	-3.6858380	0.0960570	2.6799350
C	-2.0173900	1.9659910	2.9709130
C	-5.4597000	-3.2284770	-1.2851680
C	2.9747250	-2.5293040	1.0106350
C	2.0116970	-1.7219640	0.4044560
C	0.6488400	-1.9931710	0.5508120
C	2.5982890	-3.6231980	1.7955080
C	1.2270140	-3.8674410	1.9581100
C	0.2596440	-3.0754910	1.3506500
C	3.6204490	-4.5097700	2.4488030
P	2.2825220	-0.2449250	-0.5969290
C	4.0014200	0.2955770	-0.2014510
C	2.2175080	-0.8494630	-2.3610880
C	2.2301200	0.2555390	-3.4181960
C	3.2917730	-1.9069520	-2.6224550
C	4.6432230	1.2065860	-1.2496700
C	4.0764830	0.8360210	1.2247660
Cr	0.1086880	0.7980050	-0.2367510

C	0.7913000	2.3279010	0.2400520
C	1.3492160	3.6880580	0.4816260
C	0.2255540	4.6744360	0.0835170
C	2.5870390	3.9748190	-0.3731860
C	1.6614650	3.8598370	1.9786420
C	-0.7192970	1.3573640	-1.7537600
H	-0.7931030	-3.5523430	-1.1750360
H	-3.0390560	-4.4112870	-1.6960330
H	-4.8139670	-0.7561740	-0.3035270
H	-4.4005640	1.7183810	0.4144260
H	-2.8757300	3.6393240	1.0312090
H	-3.9976340	4.0716540	-0.2665140
H	-2.3107280	3.7031710	-0.6599050
H	-3.2305390	2.1384710	-2.4029650
H	-4.8966710	2.3432090	-1.8669620
H	-4.1993070	0.7168210	-1.9520800
H	-1.5559750	-0.0652580	2.4280330
H	-3.6543880	-0.0429360	3.7708700
H	-3.8974290	-0.8830660	2.2302050
H	-4.5250580	0.7745200	2.4634870
H	-2.8436490	2.6897840	2.9245650
H	-1.1009930	2.4668920	2.6241000
H	-1.8612620	1.7272660	4.0331110
H	-6.2577630	-2.5303990	-0.9982940
H	-5.6129340	-4.1664840	-0.7304110
H	-5.5800860	-3.4611830	-2.3543500
H	4.0386120	-2.3134690	0.8858480
H	0.9056080	-4.7018740	2.5863190
H	-0.7955860	-3.2980420	1.5124470
H	3.5981530	-5.5204460	2.0123080
H	3.4210530	-4.6181940	3.5253750
H	4.6377000	-4.1134530	2.3288240

H	4.5495950	-0.6610870	-0.2300990
H	1.2327280	-1.3447150	-2.3814580
H	1.9300100	-0.1706070	-4.3869840
H	3.2303600	0.6870880	-3.5516140
H	1.5364420	1.0790530	-3.1939200
H	4.3061130	-1.4822800	-2.5965560
H	3.1431880	-2.3320980	-3.6264770
H	3.2419790	-2.7355340	-1.9019240
H	4.8995400	0.6550290	-2.1639760
H	5.5803390	1.6166600	-0.8452510
H	4.0089470	2.0539400	-1.5369750
H	5.1259730	1.0208280	1.4970490
H	3.6612760	0.1321280	1.9612830
H	3.5361320	1.7865670	1.3207790
H	0.5836420	5.6989820	0.2608130
H	-0.6799160	4.5279270	0.6865220
H	-0.0344510	4.5944600	-0.9827940
H	2.4124790	3.7523580	-1.4372450
H	2.8336770	5.0432710	-0.2961240
H	3.4608180	3.4117880	-0.0258610
H	2.0357680	4.8787120	2.1529960
H	2.4289620	3.1550460	2.3254680
H	0.7626260	3.7241560	2.5990080
H	0.3746020	2.0352920	-1.2424290
H	-1.1346030	2.2804230	-2.1763950
H	-0.8418910	0.4868300	-2.4215750
H	-0.0114070	2.0059730	1.0209950

6’-7’-TS

Symbol	X	Y	Z
C	-1.0546840	-3.2979450	-0.2001960
C	-1.1368480	-1.9092450	-0.0224640
C	-2.3828750	-1.2898820	-0.1886910
C	-2.1951060	-4.0219770	-0.5300960
C	-3.4459490	-3.4112190	-0.7087610
C	-3.5191230	-2.0282890	-0.5290870
N	-0.0307570	-1.0820050	0.2392300
P	-2.3057930	0.4683640	0.2235040
C	-3.6730030	1.2728720	-0.7241940
C	-4.0272670	2.6821720	-0.2623520
C	-3.4135830	1.2052920	-2.2290340
C	-2.6494820	0.4820910	2.0500910
C	-4.0914650	0.0577590	2.3374680
C	-2.2633070	1.7592060	2.7857400
C	-4.6545690	-4.2209790	-1.0889100
C	3.4816100	-1.5102520	1.2728860
C	2.3253250	-1.1254270	0.5896550
C	1.0573030	-1.5740190	0.9788800
C	3.3955050	-2.3613010	2.3761430
C	2.1189580	-2.8087840	2.7581800
C	0.9640170	-2.4319110	2.0833050
C	4.6182450	-2.7821680	3.1433820
P	2.1825280	-0.1420520	-0.9116550
C	3.6385030	0.9784610	-1.0328350
C	2.2122600	-1.4198320	-2.2643280
C	1.8045100	-0.9761760	-3.6658280
C	3.5780870	-2.1137980	-2.2820090
C	3.5682010	1.9093950	-2.2416550
C	3.8806590	1.7425250	0.2677520
Cr	0.0023470	0.7813900	-0.4283030
C	0.2168010	2.3451590	0.2969420

C	0.3719790	3.7859180	0.6782890
C	-0.9944120	4.4868770	0.6239940
C	1.3006720	4.4647630	-0.3552150
C	0.9804790	3.8860760	2.0864170
C	0.1555470	1.2125690	-2.1267980
H	-0.1009880	-3.8152510	-0.0826740
H	-2.1083100	-5.1033150	-0.6655600
H	-4.4823730	-1.5281080	-0.6600360
H	-4.5288810	0.6166540	-0.4896570
H	-4.2559110	2.7289390	0.8106730
H	-4.9230810	3.0222120	-0.8026030
H	-3.2237830	3.3964370	-0.4812650
H	-2.5967290	1.8829210	-2.5263980
H	-4.3140590	1.5294070	-2.7713020
H	-3.1750830	0.1871020	-2.5716860
H	-1.9740590	-0.3219300	2.3892220
H	-4.2127030	-0.0905590	3.4206960
H	-4.3548340	-0.8906090	1.8481840
H	-4.8143220	0.8274800	2.0289950
H	-2.9116010	2.6084080	2.5271710
H	-1.2192760	2.0413060	2.5956700
H	-2.3627330	1.5936730	3.8687090
H	-4.8033300	-5.0614410	-0.3946150
H	-4.5441820	-4.6493070	-2.0974040
H	-5.5684190	-3.6111070	-1.0805280
H	4.4628220	-1.1582100	0.9432880
H	2.0307580	-3.4733420	3.6215870
H	-0.0084100	-2.7994670	2.4154040
H	4.6058310	-2.3656660	4.1627860
H	5.5406270	-2.4434040	2.6524590
H	4.6685300	-3.8766500	3.2417780
H	4.4718620	0.2681300	-1.1769920

H	1.4595380	-2.1350480	-1.8908660
H	1.8521310	-1.8504800	-4.3318450
H	2.4782180	-0.2179910	-4.0880550
H	0.7728830	-0.6059060	-3.7246470
H	4.3553170	-1.4673200	-2.7171730
H	3.5110460	-3.0123200	-2.9121380
H	3.9041400	-2.4411120	-1.2849790
H	3.4990890	1.3672100	-3.1935850
H	4.4811500	2.5209270	-2.2846690
H	2.7160920	2.6017760	-2.1662570
H	4.8032620	2.3335320	0.1720570
H	3.9960530	1.0857280	1.1386530
H	3.0668900	2.4483460	0.4771440
H	-0.8652440	5.5353190	0.9280020
H	-1.7205450	4.0245320	1.3030010
H	-1.4022890	4.4881840	-0.3972050
H	0.8997640	4.3857090	-1.3781020
H	1.3654350	5.5349350	-0.1120120
H	2.3187580	4.0565570	-0.3402960
H	1.1341450	4.9451300	2.3374330
H	1.9574050	3.3833520	2.1481460
H	0.3170520	3.4546180	2.8488000
H	-0.0532520	2.2428050	-1.0939970
H	0.5088650	1.8278160	-2.9593020
H	-0.5546250	0.3867130	-2.3906250
H	0.9073790	1.5911190	0.8534970

6’-7”-TS

Symbol X Y Z

C	-1.6684120	-2.9308570	-0.9794060
C	-1.5147470	-1.6502270	-0.4280650
C	-2.6534330	-0.8634250	-0.2438420
C	-2.9382900	-3.4116190	-1.2659560
C	-4.0969300	-2.6593460	-1.0076910
C	-3.9316710	-1.3705170	-0.4997470
N	-0.2585850	-1.0987940	-0.0773400
P	-2.2536390	0.7836980	0.3591780
C	-3.5467860	1.9640400	-0.2407240
C	-3.1502020	3.4203310	-0.0081270
C	-3.9803240	1.7595090	-1.6939290
C	-2.3466210	0.6762840	2.2202590
C	-3.6858380	0.0960570	2.6799350
C	-2.0173900	1.9659910	2.9709130
C	-5.4597000	-3.2284770	-1.2851680
C	2.9747250	-2.5293040	1.0106350
C	2.0116970	-1.7219640	0.4044560
C	0.6488400	-1.9931710	0.5508120
C	2.5982890	-3.6231980	1.7955080
C	1.2270140	-3.8674410	1.9581100
C	0.2596440	-3.0754910	1.3506500
C	3.6204490	-4.5097700	2.4488030
P	2.2825220	-0.2449250	-0.5969290
C	4.0014200	0.2955770	-0.2014510
C	2.2175080	-0.8494630	-2.3610880
C	2.2301200	0.2555390	-3.4181960
C	3.2917730	-1.9069520	-2.6224550
C	4.6432230	1.2065860	-1.2496700
C	4.0764830	0.8360210	1.2247660
Cr	0.1086880	0.7980050	-0.2367510
C	0.7913000	2.3279010	0.2400520
C	1.3492160	3.6880580	0.4816260

C	0.2255540	4.6744360	0.0835170
C	2.5870390	3.9748190	-0.3731860
C	1.6614650	3.8598370	1.9786420
C	-0.7192970	1.3573640	-1.7537600
H	-0.7931030	-3.5523430	-1.1750360
H	-3.0390560	-4.4112870	-1.6960330
H	-4.8139670	-0.7561740	-0.3035270
H	-4.4005640	1.7183810	0.4144260
H	-2.8757300	3.6393240	1.0312090
H	-3.9976340	4.0716540	-0.2665140
H	-2.3107280	3.7031710	-0.6599050
H	-3.2305390	2.1384710	-2.4029650
H	-4.8966710	2.3432090	-1.8669620
H	-4.1993070	0.7168210	-1.9520800
H	-1.5559750	-0.0652580	2.4280330
H	-3.6543880	-0.0429360	3.7708700
H	-3.8974290	-0.8830660	2.2302050
H	-4.5250580	0.7745200	2.4634870
H	-2.8436490	2.6897840	2.9245650
H	-1.1009930	2.4668920	2.6241000
H	-1.8612620	1.7272660	4.0331110
H	-6.2577630	-2.5303990	-0.9982940
H	-5.6129340	-4.1664840	-0.7304110
H	-5.5800860	-3.4611830	-2.3543500
H	4.0386120	-2.3134690	0.8858480
H	0.9056080	-4.7018740	2.5863190
H	-0.7955860	-3.2980420	1.5124470
H	3.5981530	-5.5204460	2.0123080
H	3.4210530	-4.6181940	3.5253750
H	4.6377000	-4.1134530	2.3288240
H	4.5495950	-0.6610870	-0.2300990
H	1.2327280	-1.3447150	-2.3814580

H	1.9300100	-0.1706070	-4.3869840
H	3.2303600	0.6870880	-3.5516140
H	1.5364420	1.0790530	-3.1939200
H	4.3061130	-1.4822800	-2.5965560
H	3.1431880	-2.3320980	-3.6264770
H	3.2419790	-2.7355340	-1.9019240
H	4.8995400	0.6550290	-2.1639760
H	5.5803390	1.6166600	-0.8452510
H	4.0089470	2.0539400	-1.5369750
H	5.1259730	1.0208280	1.4970490
H	3.6612760	0.1321280	1.9612830
H	3.5361320	1.7865670	1.3207790
H	0.5836420	5.6989820	0.2608130
H	-0.6799160	4.5279270	0.6865220
H	-0.0344510	4.5944600	-0.9827940
H	2.4124790	3.7523580	-1.4372450
H	2.8336770	5.0432710	-0.2961240
H	3.4608180	3.4117880	-0.0258610
H	2.0357680	4.8787120	2.1529960
H	2.4289620	3.1550460	2.3254680
H	0.7626260	3.7241560	2.5990080
H	0.3746020	2.0352920	-1.2424290
H	-1.1346030	2.2804230	-2.1763950
H	-0.8418910	0.4868300	-2.4215750
H	-0.0114070	2.0059730	1.0209950

7’

Symbol	X	Y	Z
C	-0.6973520	-3.1771350	-0.7320040

C	-0.9319730	-1.8748530	-0.2682870
C	-2.2292820	-1.3277730	-0.2933710
C	-1.7592340	-3.9193180	-1.2206900
C	-3.0640520	-3.3935010	-1.2718870
C	-3.2774350	-2.0893130	-0.8111370
N	0.1136440	-1.0330740	0.1631040
P	-2.3516210	0.3577080	0.4294640
C	-3.9006700	1.0788460	-0.2829680
C	-4.3251460	2.3828100	0.3948750
C	-3.8153610	1.2211920	-1.8010950
C	-2.6221940	-0.0797200	2.2292920
C	-4.0312620	-0.6239620	2.4638870
C	-2.2408980	0.9901360	3.2467750
C	-4.2004160	-4.2334940	-1.7751500
C	3.5762070	-1.3498260	1.3111770
C	2.4413680	-0.9546320	0.6084320
C	1.1979780	-1.5173840	0.9241640
C	3.4821660	-2.2985060	2.3393860
C	2.2226710	-2.8517990	2.6281610
C	1.0812160	-2.4811340	1.9291050
C	4.6915070	-2.7135150	3.1256770
P	2.3088440	0.2128650	-0.7832990
C	3.7794840	1.3359410	-0.6448950
C	2.5248970	-0.8734510	-2.2818730
C	2.2681810	-0.1577180	-3.6089200
C	3.8569840	-1.6214520	-2.2952920
C	3.8601150	2.3479700	-1.7898590
C	3.9592750	2.0118430	0.7225010
Cr	-0.0739240	0.6415080	-0.4126970
C	0.6778340	2.3647530	0.3276330
C	-0.1741870	3.6551020	0.3699230
C	-0.8721510	3.9334410	-0.9654940

C	0.8421580	4.7847310	0.6393290
C	-1.1920180	3.6521450	1.5037140
C	-0.5309760	0.6891000	-2.0764120
H	0.3128060	-3.5902520	-0.7164410
H	-1.5793770	-4.9339720	-1.5828720
H	-4.2895940	-1.6806270	-0.8447440
H	-4.6624270	0.3182080	-0.0457400
H	-4.3284080	2.3069500	1.4915860
H	-5.3482660	2.6322310	0.0777340
H	-3.6770340	3.2220870	0.1073640
H	-3.0997150	2.0039120	-2.0950840
H	-4.7989730	1.5207720	-2.1925300
H	-3.5335400	0.2847530	-2.3042560
H	-1.9073120	-0.9114800	2.3466370
H	-4.0933460	-1.0296460	3.4846750
H	-4.2897910	-1.4399520	1.7732340
H	-4.7938530	0.1645040	2.3795330
H	-2.8932230	1.8732920	3.1972210
H	-1.1990290	1.3190710	3.1350320
H	-2.3452900	0.5639100	4.2558310
H	-3.9170170	-4.7785700	-2.6871090
H	-5.0903480	-3.6281950	-1.9926130
H	-4.4807160	-4.9871730	-1.0218790
H	4.5528260	-0.9263970	1.0633500
H	2.1390820	-3.5934660	3.4259340
H	0.1125490	-2.9230430	2.1705380
H	4.6335820	-2.3219300	4.1536610
H	5.6197010	-2.3399340	2.6739290
H	4.7590550	-3.8086040	3.2007690
H	4.6058650	0.6142040	-0.7675540
H	1.7115580	-1.6024840	-2.1217720
H	2.0315930	-0.9011050	-4.3841160

H	3.1552690	0.3916060	-3.9519380
H	1.4306280	0.5544590	-3.5683840
H	4.7062240	-0.9397820	-2.4556410
H	3.8591900	-2.3391100	-3.1295620
H	4.0315840	-2.1915190	-1.3716330
H	3.9768290	1.8718250	-2.7708700
H	4.7383310	2.9917300	-1.6354060
H	2.9779350	3.0069030	-1.8296400
H	5.0203400	2.2743550	0.8453280
H	3.6798300	1.3718170	1.5708800
H	3.3895300	2.9477550	0.7969440
H	-1.3385820	4.9295990	-0.9520990
H	-1.6699230	3.2013150	-1.1617360
H	-0.1622250	3.9179510	-1.8087730
H	1.5597260	4.8952780	-0.1881360
H	0.3142350	5.7440450	0.7532640
H	1.4076640	4.6048960	1.5665230
H	-1.7554690	4.5969720	1.5203660
H	-0.7030490	3.5397550	2.4826780
H	-1.9204560	2.8425400	1.3914170
H	1.6097610	2.6181160	-0.1768280
H	-0.4937880	1.7134250	-2.4882850
H	-0.8490470	-0.1180070	-2.7506380
H	0.9198220	1.9666050	1.3342000

7”

Symbol	X	Y	Z
C	-1.6379580	-2.9659050	-0.3321440
C	-1.5465170	-1.5926180	-0.0801610

C	-2.7007940	-0.8028550	-0.1382480
C	-2.8779890	-3.5234900	-0.6188420
C	-4.0520770	-2.7540380	-0.6479740
C	-3.9419320	-1.3816550	-0.3996610
N	-0.3108860	-0.9397350	0.1837610
P	-2.3261410	0.9217490	0.2382740
C	-3.4294580	2.0880870	-0.6781750
C	-2.8281830	3.4979710	-0.6780850
C	-3.7382420	1.6390100	-2.1064620
C	-2.6403630	1.0801010	2.0644650
C	-4.0514120	0.6372400	2.4548430
C	-2.3106410	2.4645190	2.6197360
C	-5.3793290	-3.3939370	-0.9413760
C	3.0158150	-2.1379090	1.2320280
C	1.9980470	-1.4765260	0.5467490
C	0.6633640	-1.6536540	0.9321750
C	2.7210910	-2.9687130	2.3203630
C	1.3772770	-3.1161240	2.7006840
C	0.3503320	-2.4758260	2.0195380
C	3.8064770	-3.6866250	3.0696600
P	2.1293690	-0.3501490	-0.8752880
C	3.9288430	0.0614890	-1.0439140
C	1.7183240	-1.4793160	-2.3153410
C	1.5406600	-0.7757680	-3.6617970
C	2.6802470	-2.6617190	-2.4233200
C	4.2530180	0.7927100	-2.3484130
C	4.5594450	0.7445820	0.1681320
Cr	0.0193570	0.7893520	-0.2891790
C	0.8293230	2.2726930	0.4902220
C	1.7076690	3.4785260	0.7111380
C	0.7607110	4.6509580	1.0608220
C	2.4540620	3.8171860	-0.5840310

C	2.6518440	3.2381250	1.8937830
C	-0.4857080	0.9338470	-1.9654480
H	-0.7459470	-3.5941810	-0.3140080
H	-2.9389150	-4.5945720	-0.8264360
H	-4.8425600	-0.7629730	-0.4135240
H	-4.3659110	2.0980630	-0.0958730
H	-2.5946860	3.8748600	0.3262940
H	-3.5423640	4.1996950	-1.1329380
H	-1.9099660	3.5278460	-1.2872780
H	-2.8431300	1.6834440	-2.7462940
H	-4.4740730	2.3301110	-2.5437080
H	-4.1565950	0.6263230	-2.1680510
H	-1.9180600	0.3537560	2.4776290
H	-4.1573200	0.7028210	3.5480040
H	-4.2620620	-0.4013800	2.1659980
H	-4.8228500	1.2889770	2.0162840
H	-3.0664000	3.2082910	2.3291000
H	-1.3236060	2.8272180	2.2979360
H	-2.3049680	2.4225480	3.7188700
H	-6.1946880	-2.6583990	-0.9417120
H	-5.6163230	-4.1651620	-0.1927990
H	-5.3669190	-3.8908080	-1.9232280
H	4.0578680	-2.0272750	0.9238020
H	1.1320170	-3.7516570	3.5548580
H	-0.6865180	-2.6108910	2.3326930
H	3.6112570	-4.7690100	3.1010170
H	3.8567560	-3.3373860	4.1125210
H	4.7920070	-3.5309100	2.6112520
H	4.3568850	-0.9533850	-1.1142360
H	0.7339980	-1.8616160	-1.9997950
H	0.9263540	-1.4056380	-4.3220860
H	2.4985830	-0.6128960	-4.1729700

H	1.0408180	0.2003940	-3.5841780
H	3.6815300	-2.3499870	-2.7574510
H	2.2971750	-3.3690030	-3.1742750
H	2.7827160	-3.2102540	-1.4758960
H	4.1152560	0.1532310	-3.2282770
H	5.3087460	1.1007310	-2.3342880
H	3.6476920	1.7011130	-2.4868280
H	5.6463490	0.5766080	0.1429910
H	4.1917350	0.3589720	1.1294780
H	4.4056350	1.8296120	0.1432890
H	1.3643440	5.5499800	1.2544750
H	0.1711510	4.4461420	1.9668220
H	0.0709110	4.8838430	0.2346360
H	1.7513790	3.9931830	-1.4139660
H	3.0460600	4.7336570	-0.4482350
H	3.1354610	3.0109460	-0.8800440
H	3.3591480	4.0741210	1.9906950
H	3.2333640	2.3165310	1.7795750
H	2.0886490	3.1677690	2.8371730
H	-0.0995160	2.5853390	-0.1125800
H	-0.2130110	1.8368810	-2.5370980
H	-1.0926300	0.2007200	-2.5215830
H	0.5227340	1.7511520	1.4377680

Cr^{VI} (L_1)

1

Symbol	X	Y	Z
C	-0.5323340	-3.5733980	-0.6446430
C	-0.9139580	-2.2632290	-0.3134210
C	-2.2419290	-1.8557200	-0.4762890
C	-1.4794330	-4.4778540	-1.1061100
C	-2.8118470	-4.0927170	-1.2320160
C	-3.1851810	-2.7893850	-0.9186110
N	0.0238590	-1.3165860	0.1792940
P	-2.5356750	-0.1115930	-0.0379900
C	-3.7896270	0.5092640	-1.3426060
C	-3.8976220	2.0364640	-1.3275880
C	-3.2693460	0.0498670	-2.7171350
C	-3.2698440	-0.2031690	1.7064260
C	-4.5838210	-0.9897380	1.8086490
C	-3.4846720	1.2334020	2.1949500
C	3.5352780	-2.3559780	0.6712530
C	2.4216030	-1.6164020	0.2642900
C	1.1629090	-1.9253280	0.7946880
C	3.3995590	-3.3286510	1.6590460
C	2.1565210	-3.5573490	2.2529810
C	1.0298140	-2.8718210	1.8121810
P	2.3911980	-0.1013660	-0.7399100
C	3.9960850	0.8417940	-0.3710780
C	2.2387560	-0.5461900	-2.5812610
C	2.4706730	0.6944000	-3.4553520
C	3.2142390	-1.6692060	-2.9572160
C	3.7455310	2.3247380	-0.6627290
C	4.3432050	0.6883090	1.1171520

Cr	0.0819750	0.5054580	0.0179850
C	0.4832200	1.1409300	1.5236250
C	0.8651240	1.4174200	2.9411230
C	1.8510570	2.6081630	2.9474640
C	1.4596020	0.1608090	3.5913650
C	-0.3819650	1.8640480	3.7363260
C	-0.4336210	2.0714680	-1.1499830
H	0.5070210	-3.8875280	-0.5590220
H	-1.1713100	-5.4915790	-1.3681630
H	-4.2280190	-2.5080180	-1.0401110
H	-4.1846550	2.4281360	-0.3424750
H	-4.6880420	2.3332980	-2.0338530
H	-2.9819600	2.5319860	-1.6535070
H	-2.2024480	0.2716080	-2.8789920
H	-3.8328760	0.5770800	-3.5022840
H	-3.4080820	-1.0269320	-2.8788470
H	-4.8035620	-1.1415450	2.8773930
H	-4.5144420	-1.9878760	1.3534440
H	-5.4391610	-0.4597500	1.3796810
H	-4.3242990	1.7136650	1.6718240
H	-2.5930520	1.8595360	2.0576240
H	-3.7320010	1.2289120	3.2679450
H	4.5147670	-2.1707330	0.2291640
H	2.0616680	-4.2957940	3.0512500
H	0.0456520	-3.0837810	2.2332170
H	2.3373680	0.4030320	-4.5087020
H	3.4799300	1.1131060	-3.3633520
H	1.7419640	1.4951360	-3.2537960
H	4.2633330	-1.3551470	-2.9316850
H	2.9959980	-1.9885620	-3.9885250
H	3.0939400	-2.5504280	-2.3096040
H	3.3874970	2.5136700	-1.6834830

H	4.6895250	2.8774720	-0.5412950
H	3.0252600	2.7479760	0.0471790
H	5.0212450	1.5057780	1.4056300
H	4.8563920	-0.2549320	1.3373490
H	3.4620120	0.7449670	1.7636890
H	2.2553410	2.7261030	3.9633360
H	1.3356330	3.5438470	2.6848400
H	2.6952870	2.4778060	2.2616020
H	2.3442930	-0.2152890	3.0607290
H	1.7549650	0.3936720	4.6245280
H	0.7189270	-0.6504710	3.6279490
H	-0.0473390	2.1845050	4.7338260
H	-1.1055530	1.0532500	3.8675800
H	-0.8858260	2.7194500	3.2635360
H	0.5920700	1.8223060	-1.5279060
H	-1.1578130	1.7356580	-1.8967180
H	0.1479200	2.0590320	0.9062160
C	-2.2413620	-0.9497610	2.5709260
H	-2.2038060	-2.0170890	2.3058620
H	-1.2281710	-0.5423490	2.4887880
H	-2.5523530	-0.8880470	3.6251410
C	5.1912440	0.3433600	-1.1957910
H	5.0953490	0.5503800	-2.2688380
H	6.0850450	0.8811780	-0.8426890
H	5.3865560	-0.7288820	-1.0582860
C	0.8112940	-1.0523960	-2.8411910
H	0.6231380	-2.0234670	-2.3695240
H	0.0277400	-0.3486480	-2.5149580
H	0.6826380	-1.1901750	-3.9256610
C	-5.2261970	-0.0066230	-1.1431520
H	-5.7828240	0.1957430	-2.0714980
H	-5.3211530	-1.0768890	-0.9393240

H	-5.7400000	0.5375840	-0.3405040
H	4.2717120	-3.9020440	1.9774950
H	-3.5617940	-4.8021480	-1.5849650
C	-0.5209060	3.5949030	-0.8670160
C	-1.4780410	3.9561820	0.2771990
H	-1.0811420	3.6309580	1.2525090
H	-1.5870290	5.0490500	0.3322140
H	-2.4782530	3.5299310	0.1559990
C	-0.9819690	4.2590360	-2.1802730
H	-2.0080950	3.9813280	-2.4570600
H	-0.9541170	5.3534770	-2.0756650
H	-0.3183220	3.9891130	-3.0162950
C	0.8536900	4.1758250	-0.5209880
H	1.2748310	3.7518650	0.4023330
H	1.5751840	4.0240960	-1.3363720
H	0.7656590	5.2591070	-0.3552620

1-2-TS

Symbol	X	Y	Z
C	-0.5381030	-3.6521850	-0.5176170
C	-0.9096860	-2.3270380	-0.2377650
C	-2.2243970	-1.8969460	-0.4750600
C	-1.4774180	-4.5481630	-1.0112350
C	-2.7906910	-4.1374910	-1.2251060
C	-3.1563210	-2.8210080	-0.9568240
N	0.0189270	-1.3838770	0.2674420
P	-2.5204580	-0.1445130	-0.0462170
C	-3.7484150	0.5132530	-1.3539450
C	-3.8733250	2.0408800	-1.2838590

C	-3.1817100	0.1187870	-2.7289860
C	-3.2766630	-0.2288220	1.6897290
C	-4.5755050	-1.0417390	1.7750330
C	-3.5311770	1.2106140	2.1559810
C	3.5374000	-2.3243530	0.8104970
C	2.4094410	-1.6284050	0.3658590
C	1.1545590	-1.9539060	0.9068660
C	3.4190680	-3.2665210	1.8298910
C	2.1783920	-3.5144850	2.4215740
C	1.0374930	-2.8759530	1.9496300
P	2.3552470	-0.1669940	-0.7336920
C	3.9599120	0.8034970	-0.4433620
C	2.1960800	-0.7089000	-2.5489700
C	2.4866410	0.4729290	-3.4859650
C	3.1226030	-1.8926690	-2.8526250
C	3.7101750	2.2615910	-0.8419610
C	4.3069030	0.7715430	1.0508000
Cr	0.0632170	0.4347840	0.0158990
C	0.4880190	1.1515370	1.4303840
C	0.8391490	1.6163350	2.8030020
C	1.7889500	2.8302550	2.7606070
C	1.4699040	0.4114310	3.5323360
C	-0.4274000	2.0418330	3.5728590
C	-0.3667230	2.1978780	-1.0906560
H	0.4905030	-3.9812750	-0.3739650
H	-1.1775010	-5.5736300	-1.2341170
H	-4.1855720	-2.5248700	-1.1419890
H	-4.1702120	2.3957620	-0.2887330
H	-4.6634120	2.3510360	-1.9847970
H	-2.9654100	2.5653200	-1.5884520
H	-2.1398200	0.4462210	-2.8724600
H	-3.7811510	0.6089290	-3.5115410

H	-3.2215710	-0.9634980	-2.9107320
H	-4.8284610	-1.1681970	2.8396470
H	-4.4696800	-2.0501470	1.3498120
H	-5.4269980	-0.5436510	1.3020470
H	-4.4121990	1.6432780	1.6614960
H	-2.6750210	1.8735260	1.9682940
H	-3.7333330	1.2201000	3.2383850
H	4.5173060	-2.1330910	0.3730770
H	2.0984810	-4.2315210	3.2406740
H	0.0549680	-3.0984020	2.3692320
H	2.2837070	0.1497920	-4.5184370
H	3.5292360	0.8098120	-3.4554750
H	1.8354460	1.3389830	-3.2873810
H	4.1864020	-1.6356140	-2.7860200
H	2.9330140	-2.2310360	-3.8836530
H	2.9253440	-2.7451640	-2.1856240
H	3.3672780	2.3832450	-1.8771410
H	4.6509850	2.8241700	-0.7419370
H	2.9782250	2.7277000	-0.1736930
H	5.0352260	1.5703970	1.2573260
H	4.7603400	-0.1754610	1.3652470
H	3.4322150	0.9522730	1.6809510
H	2.1738390	3.0038220	3.7760070
H	1.2529060	3.7386750	2.4534120
H	2.6493120	2.6914720	2.0961760
H	2.3837760	0.0466060	3.0473180
H	1.7242270	0.7157910	4.5579230
H	0.7593000	-0.4246290	3.5924520
H	-0.1116400	2.4068110	4.5610310
H	-1.1187990	1.2073710	3.7281560
H	-0.9597910	2.8611720	3.0721240
H	0.5482170	1.9384310	-1.6622350

H	-1.2402970	1.8473820	-1.6419840
H	0.0383970	2.0043920	0.4945510
C	-2.2499040	-0.9386990	2.5881970
H	-2.1766500	-2.0072390	2.3352570
H	-1.2446090	-0.5058470	2.5314790
H	-2.5913580	-0.8760630	3.6329420
C	5.1529520	0.2429190	-1.2303950
H	5.0561290	0.3736100	-2.3146450
H	6.0502890	0.7991880	-0.9177660
H	5.3454860	-0.8187870	-1.0240460
C	0.7463770	-1.1513090	-2.7983440
H	0.5015450	-2.0894820	-2.2890850
H	0.0028090	-0.3873330	-2.5088870
H	0.6114680	-1.3250550	-3.8768640
C	-5.1844160	-0.0226240	-1.2095510
H	-5.7229240	0.2064040	-2.1425140
H	-5.2736620	-1.0997350	-1.0433480
H	-5.7220230	0.4885310	-0.4007280
H	4.3042800	-3.8052660	2.1721850
H	-3.5335970	-4.8388280	-1.6080040
C	-0.4385490	3.7287180	-0.8628370
C	-1.3973580	4.0927110	0.2769590
H	-1.0123210	3.7359700	1.2461670
H	-1.4959460	5.1851350	0.3539240
H	-2.4020190	3.6769980	0.1388020
C	-0.9199330	4.3475460	-2.1875420
H	-1.9506110	4.0592470	-2.4372760
H	-0.8922740	5.4451980	-2.1200340
H	-0.2699930	4.0506240	-3.0253610
C	0.9381870	4.3100150	-0.5426110
H	1.3764420	3.8495350	0.3530840
H	1.6367350	4.1791120	-1.3814320

H	0.8564210	5.3883630	-0.3437700

A

Symbol	X	Y	Z
C	-0.6148680	-2.7683260	1.8230580
C	-0.8931090	-1.5766890	1.1353460
C	-2.0922390	-1.4657380	0.3815710
C	-1.4888470	-3.8423100	1.7333750
C	-2.6345730	-3.7634290	0.9388710
C	-2.9270760	-2.5828620	0.2646470
N	0.0251730	-0.5015260	1.0857580
P	-2.4973050	0.1704700	-0.3381560
C	-3.0352110	0.0056180	-2.1283030
C	-2.7864510	1.3837710	-2.7708670
C	-2.0742840	-1.0130060	-2.7615900
C	-3.7310190	0.9156560	0.8643890
C	-4.9482820	0.0021120	1.0626960
C	-4.1893100	2.2779680	0.3339380
C	3.6413730	-0.6599830	1.7210390
C	2.4132250	-0.5422150	1.0685310
C	1.2297330	-0.5250980	1.8380330
C	3.6866930	-0.7226950	3.1135680
C	2.5100980	-0.6655680	3.8624410
C	1.2720680	-0.5794440	3.2295960
P	2.1557070	-0.3059560	-0.7376190
C	3.4580110	0.8974030	-1.4018850

C	2.2591650	-1.9979920	-1.5759470
C	2.1132800	-1.8115030	-3.0915630
C	3.5521960	-2.7464120	-1.2285230
C	2.7906820	1.6464180	-2.5683420
C	3.8307200	1.8809740	-0.2863030
Cr	-0.1352110	0.4591000	-0.4049210
C	0.2609930	1.9362690	0.1223300
C	0.4235580	3.2946090	0.6951690
C	1.2191780	4.2054680	-0.2624100
C	1.0951140	3.1644050	2.0784410
C	-0.9756320	3.9233060	0.8623090
H	0.3016390	-2.8661050	2.4043350
H	-1.2631960	-4.7618800	2.2765210
H	-3.8407720	-2.5139670	-0.3272330
H	-3.4083550	2.1754550	-2.3318910
H	-3.0304020	1.3296580	-3.8429800
H	-1.7311840	1.6982820	-2.6899120
H	-1.0078160	-0.7716130	-2.5768460
H	-2.2051590	-1.0077250	-3.8544020
H	-2.2456660	-2.0381460	-2.4056620
H	-5.6143200	0.4843950	1.7954250
H	-4.6751370	-0.9788620	1.4747600
H	-5.5299610	-0.1450710	0.1441980
H	-4.8019150	2.1730500	-0.5728390
H	-3.3521010	2.9505970	0.1125350
H	-4.8158360	2.7649770	1.0965530
H	4.5688130	-0.7045730	1.1464540
H	2.5571510	-0.6992210	4.9523400
H	0.3453330	-0.5545140	3.8058880
H	2.0703550	-2.8033830	-3.5668860
H	2.9577110	-1.2738980	-3.5410690
H	1.1837180	-1.2845100	-3.3609190

H	4.4513070	-2.2816300	-1.6450340
H	3.4872840	-3.7612780	-1.6512710
H	3.6815210	-2.8565720	-0.1414980
H	2.4877200	0.9704010	-3.3814290
H	3.5079900	2.3642780	-2.9949620
H	1.9081460	2.2151760	-2.2400390
H	4.2793290	2.7792100	-0.7367060
H	4.5719860	1.4599380	0.4042910
H	2.9677890	2.1950350	0.3077850
H	1.2341470	5.2192830	0.1630930
H	0.7308950	4.2661610	-1.2462410
H	2.2557110	3.8885910	-0.4046220
H	2.0984750	2.7212550	2.0243200
H	1.1923160	4.1682570	2.5176340
H	0.4841210	2.5531050	2.7584290
H	-0.8519760	4.9412740	1.2594970
H	-1.6032000	3.3646250	1.5660880
H	-1.4930580	4.0024290	-0.1043530
C	-2.9895750	1.0455490	2.2043620
H	-2.8179550	0.0610160	2.6648120
H	-2.0187860	1.5492640	2.1072350
H	-3.6104180	1.6299540	2.9004470
C	4.7546390	0.2397670	-1.8887510
H	4.6149600	-0.3975160	-2.7709070
H	5.4495840	1.0432740	-2.1782120
H	5.2471580	-0.3442550	-1.0990140
C	1.0854130	-2.8396260	-1.0677470
H	1.1562020	-3.0264570	0.0112660
H	0.1033960	-2.3955020	-1.2898210
H	1.1111820	-3.8194970	-1.5686870
C	-4.4889100	-0.4121810	-2.3515810
H	-4.6647910	-0.5252380	-3.4328480

H	-4.7350820	-1.3732300	-1.8801140
H	-5.1935260	0.3476300	-1.9864260
H	4.6492230	-0.8172860	3.6189220
H	-3.3122360	-4.6153730	0.8650490

5-6-TS

Symbol	X	Y	Z
C	-0.7322490	-3.2416140	1.0012470
C	-0.9900870	-1.9201450	0.6035690
C	-2.2054370	-1.5970030	-0.0383490
C	-1.6624840	-4.2365440	0.7328250
C	-2.8473980	-3.9316540	0.0627580
C	-3.1164430	-2.6184220	-0.3142370
N	-0.0565600	-0.8790830	0.7706830
P	-2.4666330	0.1981530	-0.2867220
C	-3.6318120	0.4826120	-1.7582160
C	-3.6142360	1.9854780	-2.1039380
C	-3.1171150	-0.3459800	-2.9480940
C	-3.3105900	0.6976040	1.3553120
C	-4.3586370	-0.3449760	1.7737650
C	-3.9776970	2.0726550	1.2068330
C	3.4596530	-1.2000920	1.8219910
C	2.3294830	-0.9015090	1.0562000
C	1.0496420	-1.0638050	1.6325840
C	3.3201610	-1.6151510	3.1435470
C	2.0513980	-1.7300630	3.7179530
C	0.9134210	-1.4687230	2.9655010
P	2.3272940	-0.2329580	-0.6532220
C	3.7769400	0.9720950	-0.8298040

C	2.4596430	-1.6883190	-1.8583150
C	2.6322300	-1.1643920	-3.2908840
C	3.6074380	-2.6341640	-1.4808680
C	3.3983300	1.9726410	-1.9320330
C	3.9588520	1.7113110	0.5023230
Cr	0.0339880	0.5514890	-0.3799140
C	0.3557190	2.0030520	0.3106160
C	0.4993150	3.3374440	0.9633950
C	1.4537070	4.2476470	0.1674730
C	0.9806420	3.1499560	2.4135030
C	-0.8948340	3.9999040	0.9692650
C	-0.1026900	1.1933570	-2.3695480
H	0.2087720	-3.4977790	1.4885080
H	-1.4533000	-5.2644170	1.0340650
H	-4.0604180	-2.3979640	-0.8092230
H	-2.6108140	2.4306660	-2.1097810
H	-4.2288560	2.5716870	-1.4101110
H	-4.0406340	2.1214870	-3.1096700
H	-2.1125040	-0.0589670	-3.2761300
H	-3.7913570	-0.1794640	-3.8021030
H	-3.1166980	-1.4247320	-2.7402210
H	-4.8809470	0.0371960	2.6649720
H	-3.8982280	-1.3033590	2.0495440
H	-5.1202890	-0.5344610	1.0083470
H	-4.9138900	2.0208450	0.6365180
H	-3.3291710	2.8189840	0.7320800
H	-4.2338490	2.4479750	2.2094510
H	4.4575200	-1.1234160	1.3892450
H	1.9509270	-2.0403380	4.7594640
H	-0.0805620	-1.5888630	3.3997340
H	2.6143220	-2.0231410	-3.9798310
H	3.5882550	-0.6492320	-3.4474110

H	1.8174870	-0.4902130	-3.5928670
H	4.5980900	-2.1825450	-1.6007350
H	3.5681080	-3.5080280	-2.1498260
H	3.5119250	-3.0086090	-0.4510650
H	3.2436400	1.4915570	-2.9077460
H	4.2190900	2.6956900	-2.0539530
H	2.4963010	2.5419810	-1.6734420
H	4.5183770	2.6419010	0.3225770
H	4.5332380	1.1211220	1.2264820
H	3.0061020	1.9751060	0.9747630
H	1.4556330	5.2410300	0.6384690
H	1.1169840	4.3765840	-0.8725330
H	2.4845870	3.8795440	0.1647120
H	1.9932890	2.7298310	2.4692500
H	0.9982010	4.1311350	2.9094120
H	0.3074690	2.4949760	2.9844710
H	-0.7901800	5.0225570	1.3596090
H	-1.5939560	3.4647850	1.6211220
H	-1.3207330	4.0700170	-0.0426320
H	0.7900650	1.6463840	-2.8171640
H	-0.1459970	0.1288290	-2.6516570
H	-0.9913290	1.7125980	-2.7355430
H	0.1873860	2.0195180	-1.0703760
C	-2.2486770	0.7526430	2.4581490
H	-1.7926890	-0.2297890	2.6361100
H	-1.4475440	1.4671800	2.2394290
H	-2.7350060	1.0597750	3.3969100
C	5.1144380	0.3113560	-1.1898740
H	5.1220680	-0.1318930	-2.1931310
H	5.8857230	1.0972070	-1.1804040
H	5.4254840	-0.4522200	-0.4651240
C	1.1506660	-2.4801840	-1.7736590

H	1.0315860	-2.9631600	-0.7961700
H	0.2573810	-1.8675840	-1.9703360
H	1.1658680	-3.2773680	-2.5323150
C	-5.0961510	0.0868760	-1.5052590
H	-5.6615370	0.3065960	-2.4244190
H	-5.2350550	-0.9808380	-1.2953060
H	-5.5649690	0.6632740	-0.6998450
H	4.2083120	-1.8559970	3.7302280
H	-3.5722370	-4.7170690	-0.1564850

6

Symbol	X	Y	Z
C	-0.8108590	-2.9802900	1.5547060
C	-1.0476260	-1.7781720	0.8786560
C	-2.2634010	-1.5606550	0.2042200
C	-1.7764190	-3.9802700	1.5256240
C	-2.9729120	-3.7873030	0.8360800
C	-3.2184720	-2.5784060	0.1878150
N	-0.0927360	-0.7397420	0.8085580
P	-2.4614350	0.1540060	-0.3916700
C	-3.6056080	0.1942930	-1.9151990
C	-3.4684910	1.5750090	-2.5858070
C	-3.2084090	-0.9302300	-2.8899240
C	-3.3039930	0.9831940	1.1100070
C	-4.4683630	0.1444010	1.6601550
C	-3.8085810	2.3744130	0.7028520
C	3.4140580	-0.7028790	1.9961830
C	2.3056980	-0.6409710	1.1455770
C	1.0084220	-0.6966610	1.7053220

C	3.2361490	-0.7818590	3.3735160
C	1.9487910	-0.7890180	3.9202770
C	0.8341970	-0.7561740	3.0941930
P	2.3541270	-0.3837620	-0.6657170
C	3.7849520	0.7779510	-1.1037260
C	2.5217530	-2.0864920	-1.4847230
C	2.6347570	-1.9331350	-3.0081730
C	3.7329420	-2.8495630	-0.9278510
C	3.3362830	1.5454340	-2.3591970
C	4.0052900	1.7687550	0.0446280
Cr	0.0689010	0.4748710	-0.4935190
C	0.2768060	2.1229460	-0.1057840
C	0.5343920	3.3893710	0.6514430
C	1.4951380	4.2642490	-0.1847380
C	1.0833230	3.0986210	2.0551940
C	-0.7892890	4.1756350	0.7740460
C	-0.1518320	0.2729020	-2.4753300
H	0.1294290	-3.1459700	2.0813240
H	-1.5882560	-4.9226530	2.0431630
H	-4.1740830	-2.4306900	-0.3137100
H	-3.9529460	2.3651830	-1.9986580
H	-3.9750300	1.5418270	-3.5625580
H	-2.4318470	1.8872120	-2.7683150
H	-2.2012250	-0.8296270	-3.3026870
H	-3.9068190	-0.9023010	-3.7401870
H	-3.2968680	-1.9263970	-2.4354430
H	-4.9129290	0.7008500	2.5001540
H	-4.1297470	-0.8232220	2.0551250
H	-5.2679400	-0.0344020	0.9344050
H	-4.7303280	2.3198960	0.1095770
H	-3.0687020	2.9470920	0.1301840
H	-4.0459110	2.9502510	1.6104420

H	4.4227180	-0.7055060	1.5810840
H	1.8164200	-0.8357510	5.0024430
H	-0.1713520	-0.7912510	3.5145410
H	2.7126430	-2.9381670	-3.4507780
H	3.5272510	-1.3756170	-3.3208150
H	1.7513860	-1.4516980	-3.4497270
H	4.6919010	-2.4311020	-1.2491850
H	3.6884880	-3.8831810	-1.3050940
H	3.7205780	-2.9071350	0.1705390
H	3.0861610	0.8812250	-3.1997030
H	4.1580750	2.1969200	-2.6934900
H	2.4694420	2.1892340	-2.1483050
H	4.5666570	2.6342190	-0.3392080
H	4.5956580	1.3361220	0.8611590
H	3.0689680	2.1385160	0.4705670
H	1.7227810	5.1798770	0.3801320
H	1.0251230	4.5671960	-1.1324810
H	2.4437940	3.7696410	-0.4148920
H	2.0234240	2.5312350	2.0354540
H	1.2754490	4.0479320	2.5758200
H	0.3582260	2.5317150	2.6561740
H	-0.5556890	5.1732240	1.1737550
H	-1.4910240	3.6967320	1.4652230
H	-1.2780000	4.3156030	-0.2017940
H	0.7934120	0.6359360	-2.9017320
H	-0.2357120	-0.8158290	-2.5947240
H	-0.9820430	0.7873730	-2.9617330
H	0.1190500	2.2423360	-1.2255220
C	-2.2703640	1.0940190	2.2364650
H	-1.9984790	0.1027570	2.6243380
H	-1.3537580	1.6085690	1.9309920
H	-2.7171730	1.6559990	3.0710130

C	5.1206780	0.0723880	-1.3697060
H	5.1024260	-0.5820560	-2.2496460
H	5.8765300	0.8496980	-1.5612130
H	5.4648720	-0.5070460	-0.5017060
C	1.2666300	-2.9011640	-1.1520870
H	1.1992710	-3.1227990	-0.0792950
H	0.3323970	-2.4173250	-1.4700020
H	1.3289880	-3.8664810	-1.6769060
C	-5.0943130	-0.0075340	-1.5824740
H	-5.6491790	0.0199240	-2.5329970
H	-5.3092320	-0.9770160	-1.1150890
H	-5.5092190	0.7871730	-0.9526890
H	4.1063440	-0.8417260	4.0293670
H	-3.7271690	-4.5757310	0.8152280

6’

Symbol	X	Y	Z
C	-0.5222270	-3.3852170	0.5020220
C	-0.8694270	-2.0399480	0.3145440
C	-2.1410620	-1.6894990	-0.1752100
C	-1.4364080	-4.3814360	0.1888950
C	-2.6977410	-4.0466410	-0.3031200
C	-3.0471650	-2.7102950	-0.4761120
N	0.0235620	-0.9833310	0.6055770
P	-2.4562640	0.1126220	-0.2261220
C	-3.6404250	0.4604410	-1.6882220
C	-3.6602910	1.9702760	-1.9913350
C	-3.1561660	-0.3324140	-2.9163540
C	-3.3169280	0.4310330	1.4484400

C	-4.3993380	-0.6189370	1.7466830
C	-3.9397710	1.8350080	1.4472750
C	3.5079520	-1.2741770	1.8337880
C	2.4137670	-0.9421860	1.0305670
C	1.1156340	-1.2346790	1.4900220
C	3.3102690	-1.8537950	3.0844560
C	2.0166060	-2.1020070	3.5466910
C	0.9156140	-1.8022350	2.7524550
P	2.4615920	-0.0671340	-0.5711840
C	3.8039010	1.2560920	-0.4975590
C	2.7813290	-1.3588950	-1.9151420
C	2.9523120	-0.6628990	-3.2723240
C	4.0030220	-2.2310040	-1.5964710
C	3.4358710	2.3175590	-1.5462460
C	3.7876250	1.8688870	0.9110190
Cr	0.0683090	0.5488130	-0.3150700
C	0.2496090	1.8913500	0.8223680
C	-0.0079330	3.3807600	0.8436440
C	-0.8170180	3.8487780	-0.3687710
C	1.3171620	4.1687030	0.9146230
C	-0.7746170	3.6911030	2.1497450
C	-0.2538980	1.0757860	-2.1349090
H	0.4641660	-3.6545240	0.8786120
H	-1.1611340	-5.4282370	0.3282100
H	-4.0479640	-2.4726370	-0.8322720
H	-4.0250060	2.5608630	-1.1416850
H	-4.3599250	2.1410220	-2.8237270
H	-2.6960670	2.3882700	-2.3018650
H	-2.0899330	-0.2144550	-3.1373930
H	-3.7111120	0.0220510	-3.7985420
H	-3.3574020	-1.4072120	-2.8170070
H	-4.8741420	-0.3498260	2.7032820

H	-3.9758780	-1.6256310	1.8673990
H	-5.1946490	-0.6607970	0.9960970
H	-4.8729600	1.8724460	0.8705630
H	-3.2668710	2.6049310	1.0465600
H	-4.1886940	2.1182170	2.4813760
H	4.5236430	-1.0811420	1.4862580
H	1.8643430	-2.5453340	4.5323350
H	-0.0951100	-2.0251200	3.0972410
H	2.9931070	-1.4323900	-4.0584560
H	3.8843510	-0.0862510	-3.3394460
H	2.1132810	0.0064750	-3.5134760
H	4.9493490	-1.6805350	-1.6318380
H	4.0629610	-3.0287790	-2.3534220
H	3.9146550	-2.7225280	-0.6163220
H	3.4370560	1.9186830	-2.5696060
H	4.1783380	3.1297620	-1.5130710
H	2.4509650	2.7629860	-1.3540280
H	4.2863030	2.8492360	0.8811890
H	4.3303310	1.2467580	1.6339110
H	2.7751830	2.0257520	1.3019660
H	-1.0701710	4.9131810	-0.2610020
H	-1.7560720	3.2857970	-0.4609040
H	-0.2530050	3.7406940	-1.3065970
H	1.9452670	4.0199180	0.0291460
H	1.0669970	5.2382260	0.9698080
H	1.9004530	3.9173130	1.8105880
H	-0.9464910	4.7761960	2.2059290
H	-0.1939890	3.3970090	3.0368490
H	-1.7514040	3.1986240	2.1977870
H	-1.1228680	1.6032300	-2.5243020
H	0.6033320	1.7796970	-2.0331630
H	0.0318490	0.2180630	-2.7620600

H	0.5853670	1.4693400	1.7897130
C	-2.2687130	0.3119840	2.5618080
H	-1.8214420	-0.6912330	2.5908700
H	-1.4658190	1.0530610	2.4846730
H	-2.7688440	0.4714800	3.5292960
C	-5.1007780	0.0516800	-1.4266080
H	-5.5780460	0.6460200	-0.6387270
H	-5.6642650	0.2402340	-2.3536470
H	-5.2294640	-1.0115310	-1.1930380
C	5.2209020	0.7408660	-0.7796680
H	5.3613650	0.4206420	-1.8196190
H	5.9237680	1.5691810	-0.5994610
H	5.5138150	-0.0845230	-0.1160390
C	1.5524860	-2.2750350	-1.9651380
H	1.4685080	-2.8848370	-1.0567770
H	0.6081960	-1.7296280	-2.1035630
H	1.6600950	-2.9707700	-2.8111520
H	-3.4185630	-4.8295490	-0.5436500
H	4.1710330	-2.1124560	3.7032860

6’-7’-TS

Symbol	X	Y	Z
C	-0.5575230	-3.5738840	0.3358990
C	-0.8527910	-2.2034440	0.2260450
C	-2.1213690	-1.8286990	-0.2455840
C	-1.5042750	-4.5272080	-0.0181240
C	-2.7649230	-4.1501280	-0.4829070
C	-3.0707200	-2.7989660	-0.5869800
N	0.0881250	-1.1921620	0.4903760

P	-2.3754440	-0.0403210	-0.2058060
C	-3.3813060	0.5003820	-1.7221380
C	-3.2056420	2.0081850	-1.9350630
C	-2.8474370	-0.2459380	-2.9534860
C	-3.2517590	0.2539150	1.4525400
C	-4.3831590	-0.7636490	1.6620850
C	-3.8068810	1.6760940	1.5319060
C	3.5291190	-1.2905250	1.8374990
C	2.4212260	-1.0090980	1.0299930
C	1.1452270	-1.4797100	1.3737210
C	3.3593390	-2.0105850	3.0145650
C	2.0818920	-2.4379750	3.3857680
C	0.9804780	-2.1780640	2.5787910
P	2.3939850	-0.0386620	-0.4974890
C	3.6234920	1.3910690	-0.3439790
C	2.7862930	-1.2362430	-1.9166120
C	2.9966880	-0.4980440	-3.2434790
C	4.0483100	-2.0406940	-1.5564730
C	3.2477820	2.4925300	-1.3420920
C	3.5432270	1.9310310	1.0924130
Cr	-0.0119560	0.6085200	-0.2606080
C	-0.1767570	2.1915780	0.4339910
C	-0.3601890	3.6196580	0.8632420
C	-1.4849130	4.2988740	0.0708420
C	0.9384010	4.4136560	0.5978690
C	-0.6460550	3.6331070	2.3770110
C	0.4197300	1.0886990	-1.9135320
H	0.4209970	-3.8994850	0.6894080
H	-1.2489400	-5.5855970	0.0659860
H	-4.0601580	-2.5050220	-0.9368260
H	-3.6076260	2.5971410	-1.1013250
H	-3.7591740	2.3049540	-2.8390760

H	-2.1548180	2.2896700	-2.0917030
H	-1.8008770	0.0013050	-3.1829230
H	-3.4368190	0.0648970	-3.8296940
H	-2.9353710	-1.3368200	-2.8657020
H	-4.8735230	-0.5328370	2.6206010
H	-4.0066300	-1.7930600	1.7271630
H	-5.1565630	-0.7197710	0.8856340
H	-4.6392450	1.8477830	0.8364710
H	-3.0326050	2.4258100	1.3388080
H	-4.1894820	1.8540740	2.5486650
H	4.5271890	-0.9552550	1.5534420
H	1.9422950	-2.9834810	4.3212330
H	-0.0103860	-2.5285940	2.8719740
H	3.2265490	-1.2463530	-4.0171640
H	3.8390600	0.2057980	-3.2187090
H	2.0998420	0.0375870	-3.5868280
H	4.9595160	-1.4339780	-1.5311840
H	4.1898820	-2.8051710	-2.3353680
H	3.9438640	-2.5701670	-0.5993710
H	3.2768520	2.1459470	-2.3846620
H	3.9764300	3.3129220	-1.2540930
H	2.2588430	2.9165310	-1.1344870
H	4.0674580	2.8976880	1.1342070
H	4.0239230	1.2619470	1.8159290
H	2.5134430	2.1006000	1.4326360
H	-1.5312060	5.3575150	0.3626920
H	-2.4681620	3.8600760	0.2685570
H	-1.2989380	4.2649670	-1.0130740
H	1.1554190	4.4973650	-0.4761540
H	0.7922410	5.4335220	0.9815070
H	1.8095910	3.9890670	1.1093530
H	-0.7451330	4.6766500	2.7088410

H	0.1787020	3.1780850	2.9470450
H	-1.5752010	3.1080500	2.6328720
H	-0.0934140	2.0344110	-0.9831830
H	0.8404870	1.8051520	-2.6245880
H	-0.0287580	0.1794260	-2.3727240
H	0.5816320	1.5949170	1.0875130
C	5.0762820	0.9661100	-0.6135920
H	5.2545380	0.6998430	-1.6623750
H	5.7200020	1.8296950	-0.3861990
H	5.4133130	0.1378260	0.0222560
C	1.6321120	-2.2379490	-2.0595440
H	1.4937070	-2.8410490	-1.1539710
H	0.6681200	-1.7789190	-2.3186570
H	1.8855970	-2.9299490	-2.8767810
C	-2.2046070	0.0446240	2.5561030
H	-1.7814380	-0.9696880	2.5377160
H	-1.3788580	0.7673320	2.4945880
H	-2.6932750	0.1799670	3.5331910
C	-4.8829900	0.2024360	-1.5789350
H	-5.3537220	0.7710840	-0.7674370
H	-5.3810280	0.5038120	-2.5134760
H	-5.1020470	-0.8618810	-1.4288060
H	4.2211010	-2.2347040	3.6447860
H	-3.5030270	-4.9031200	-0.7617100

6’-7”-TS

Symbol	X	Y	Z
C	-0.4341580	-3.4990580	0.5113380
C	-0.7699450	-2.1435550	0.3436520

C	-2.0271640	-1.8247440	-0.2012510
C	-1.3279290	-4.4952900	0.1414350
C	-2.5728650	-4.1758610	-0.4023040
C	-2.9189770	-2.8403580	-0.5650050
N	0.0962080	-1.0834490	0.6464800
P	-2.3623320	-0.0444320	-0.2070140
C	-3.4345530	0.3844380	-1.7204430
C	-3.4189100	1.8994390	-1.9616260
C	-2.8503780	-0.3424020	-2.9429230
C	-3.2672440	0.2482210	1.4372710
C	-4.3337100	-0.8364260	1.6636930
C	-3.9213920	1.6342920	1.4482500
C	3.6364660	-1.3145580	1.7429510
C	2.4967520	-0.9872500	1.0006350
C	1.2223290	-1.3604260	1.4553980
C	3.5076850	-1.9696430	2.9631360
C	2.2361560	-2.2884810	3.4438520
C	1.0984260	-1.9886020	2.7017140
P	2.4034880	-0.0253080	-0.5247960
C	3.6541030	1.3866460	-0.4594160
C	2.6904200	-1.1980070	-1.9826430
C	2.7770260	-0.4133040	-3.2966880
C	3.9673980	-2.0238930	-1.7632530
C	3.2492590	2.4766510	-1.4621990
C	3.6260250	1.9472860	0.9699160
Cr	0.0227500	0.6084510	-0.3257080
C	-0.0893080	2.1114810	0.5788150
C	-0.3567510	3.5422290	0.9531010
C	-1.3225800	4.1814730	-0.0500460
C	0.9706110	4.3315430	0.9324500
C	-0.9110420	3.6016960	2.3877340
C	-0.1610530	1.3388810	-1.8889610

H	0.5356190	-3.7790660	0.9215540
H	-1.0428590	-5.5410530	0.2731950
H	-3.8973020	-2.5964260	-0.9760080
H	-3.9256560	2.4521260	-1.1601320
H	-3.9617980	2.1123930	-2.8952650
H	-2.4068280	2.3073690	-2.0719810
H	-1.8151860	-0.0525910	-3.1723370
H	-3.4507980	-0.0752390	-3.8257540
H	-2.8829590	-1.4350540	-2.8410980
H	-4.8698560	-0.5899790	2.5934190
H	-3.8872090	-1.8309800	1.7963330
H	-5.0824130	-0.8909300	0.8653370
H	-4.8209220	1.6762940	0.8204090
H	-3.2387140	2.4258050	1.1151650
H	-4.2335530	1.8754160	2.4758220
H	4.6297040	-1.0513100	1.3773430
H	2.1284780	-2.7809340	4.4123720
H	0.1086250	-2.2567870	3.0751810
H	2.8347930	-1.1297140	-4.1303860
H	3.6720650	0.2202860	-3.3527890
H	1.8930000	0.2196350	-3.4761350
H	4.8833130	-1.4254570	-1.8119600
H	4.0283410	-2.7786950	-2.5628160
H	3.9475150	-2.5664330	-0.8071280
H	3.2068200	2.1095350	-2.4956910
H	4.0003100	3.2809130	-1.4332200
H	2.2818310	2.9352870	-1.2216600
H	4.1288480	2.9261340	0.9823310
H	4.1496800	1.2928810	1.6779810
H	2.6054040	2.0920020	1.3476010
H	-1.4884360	5.2328120	0.2241320
H	-2.2970370	3.6797980	-0.0630410

H	-0.9113100	4.1697270	-1.0716450
H	1.4403280	4.3417460	-0.0602240
H	0.7418730	5.3737160	1.1975870
H	1.6921880	3.9530350	1.6669190
H	-1.0421970	4.6566210	2.6683590
H	-0.2133840	3.1499590	3.1087110
H	-1.8837130	3.1071140	2.4861580
H	0.4374520	2.0917910	-0.7598720
H	-0.3291540	2.0686450	-2.6851550
H	0.0376920	0.2900010	-2.2391040
H	0.3899430	1.4540520	1.3614020
C	-2.2443700	0.1286080	2.5751340
H	-1.7449500	-0.8498500	2.5794750
H	-1.4764030	0.9105800	2.5460970
H	-2.7772870	0.2331960	3.5326110
C	-4.9061470	-0.0404520	-1.5658700
H	-5.4215330	0.4996270	-0.7628180
H	-5.4266200	0.2083660	-2.5033880
H	-5.0414830	-1.1164690	-1.4027090
C	5.0880310	0.9411000	-0.7879790
H	5.2151580	0.6820120	-1.8469570
H	5.7600970	1.7876740	-0.5785000
H	5.4318890	0.0961780	-0.1772200
C	1.5111220	-2.1791700	-2.0443260
H	1.4581850	-2.8137900	-1.1511460
H	0.5308090	-1.6946960	-2.1737100
H	1.6560920	-2.8436490	-2.9094270
H	-3.2695740	-4.9621330	-0.6954750
H	4.3962920	-2.2190820	3.5447640

7’

Symbol	X	Y	Z
C	-0.6571050	-3.1555040	1.5902160
C	-0.8874990	-1.8954930	1.0138100
C	-2.0949310	-1.6951980	0.3198680
C	-1.6066930	-4.1664640	1.4815450
C	-2.8038740	-3.9587920	0.8005570
C	-3.0421760	-2.7173090	0.2228810
N	0.0684820	-0.8499000	1.0104370
P	-2.3012870	-0.0212510	-0.3084660
C	-3.1378580	-0.0187310	-2.0136470
C	-2.9989900	1.3659640	-2.6670480
C	-2.4450600	-1.0842710	-2.8757230
C	-3.3839190	0.8203980	1.0203240
C	-4.6160990	-0.0418840	1.3411630
C	-3.8255220	2.2066540	0.5402640
C	3.7122000	-1.3152000	1.5073220
C	2.4755710	-1.0083110	0.9304750
C	1.2975280	-1.1022730	1.6819920
C	3.7729860	-1.6689480	2.8512570
C	2.6061210	-1.6848740	3.6193850
C	1.3692700	-1.4046620	3.0454170
P	2.1680930	-0.2962910	-0.7047970
C	3.5191670	0.9898820	-1.0526970
C	2.0833680	-1.6974300	-1.9715960
C	1.9970480	-1.1704580	-3.4078490
C	3.3086020	-2.6129490	-1.8154940
C	2.9461240	2.0583090	-1.9931140

C	3.9673620	1.6574500	0.2587610
Cr	0.0434230	0.6717820	-0.1098360
C	0.3035590	2.1754820	1.0281130
C	0.1516850	3.7004020	0.9939740
C	-0.2322050	4.2410520	-0.3863030
C	1.5130960	4.2942180	1.4076200
C	-0.8993640	4.1306640	2.0272000
C	-0.0214670	1.0256180	-1.7860170
H	0.2736140	-3.3706190	2.1113760
H	-1.3979390	-5.1377840	1.9344620
H	-3.9780860	-2.5456670	-0.3077090
H	-3.4623280	2.1646980	-2.0755010
H	-3.5174510	1.3402470	-3.6377450
H	-1.9626220	1.6553480	-2.8777570
H	-1.3612520	-0.9314160	-2.9643110
H	-2.8646370	-1.0389810	-3.8922950
H	-2.6123630	-2.1003740	-2.4923950
H	-5.2316800	0.5241460	2.0578570
H	-4.3401220	-0.9874580	1.8269440
H	-5.2508470	-0.2593250	0.4770480
H	-4.6033930	2.1474740	-0.2334470
H	-2.9909280	2.8114830	0.1556400
H	-4.2619960	2.7531780	1.3905780
H	4.6273920	-1.2725410	0.9148360
H	2.6603240	-1.9264260	4.6827110
H	0.4591250	-1.4362770	3.6466260
H	2.0376250	-2.0327210	-4.0908850
H	2.8258960	-0.5045960	-3.6804420
H	1.0493140	-0.6558910	-3.6148310
H	4.2398090	-2.1610910	-2.1736980
H	3.1285330	-3.5122900	-2.4241650
H	3.4475890	-2.9501230	-0.7784890

H	2.4853630	1.6380720	-2.8994990
H	3.7628920	2.7194220	-2.3197400
H	2.2039980	2.6879860	-1.4831190
H	4.5388310	2.5622590	0.0006970
H	4.6295990	1.0142700	0.8491260
H	3.1484260	1.9751160	0.9164870
H	-0.3403380	5.3341560	-0.3436870
H	-1.1936130	3.8309210	-0.7329770
H	0.5424130	4.0346610	-1.1424280
H	2.2914460	4.0897680	0.6573140
H	1.4237180	5.3861010	1.5060760
H	1.8526550	3.8986890	2.3769710
H	-0.9364880	5.2286670	2.0767330
H	-0.6508260	3.7608300	3.0337170
H	-1.9063280	3.7789730	1.7702200
H	1.3254860	1.8389110	0.6155110
H	-0.0424260	2.1019550	-1.4567280
H	-0.0822880	0.8602900	-2.8633030
H	0.2381350	1.7616320	2.0465040
C	-2.5741650	0.9342210	2.3161060
H	-2.1497270	-0.0293000	2.6322740
H	-1.7719590	1.6709130	2.2441690
H	-3.2524060	1.2705920	3.1150860
C	-4.6416560	-0.3356620	-1.9603100
H	-5.2181330	0.4624970	-1.4765870
H	-5.0019310	-0.4061460	-2.9979350
H	-4.8790380	-1.2912560	-1.4773980
C	4.7626450	0.3450860	-1.6861210
H	4.5865050	-0.0289400	-2.7026760
H	5.5431600	1.1181630	-1.7548850
H	5.1712490	-0.4710780	-1.0750940
C	0.8325090	-2.5217430	-1.6476010

H	0.9114380	-3.0210800	-0.6730180
H	-0.0839550	-1.9201170	-1.6560270
H	0.7257440	-3.3071560	-2.4111360
H	4.7335190	-1.9128070	3.3076180
H	-3.5438650	-4.7557540	0.7168880

7”

Symbol	X	Y	Z
C	-0.8487450	-3.5459890	0.0344260
C	-1.0822560	-2.1647340	0.0462070
C	-2.3721190	-1.6844980	-0.2173260
C	-1.8990810	-4.4223230	-0.2175210
C	-3.1898780	-3.9448790	-0.4458530
C	-3.4265240	-2.5740800	-0.4405460
N	-0.0452340	-1.2176000	0.2896190
P	-2.4556330	0.1157910	-0.1606520
C	-3.6025480	0.7897880	-1.4995980
C	-3.2525460	2.2679750	-1.7294690
C	-3.3734740	0.0122240	-2.8028010
C	-2.9946710	0.5503580	1.6033820
C	-4.1553840	-0.3379810	2.0686640
C	-3.3796260	2.0273070	1.7194630
C	3.2796230	-1.5280500	1.8983780
C	2.2642110	-1.1597750	1.0139320
C	0.9487320	-1.5920090	1.2416780
C	2.9848170	-2.2957970	3.0225030
C	1.6719040	-2.6986160	3.2617430
C	0.6539390	-2.3543130	2.3782400
P	2.3947300	-0.1385440	-0.4789560

C	3.7256550	1.1724830	-0.2223290
C	2.8498950	-1.3755550	-1.8570000
C	2.8985850	-0.6524850	-3.2064260
C	4.2014330	-2.0533200	-1.5719990
C	3.5298120	2.2531100	-1.2970540
C	3.4971270	1.7224960	1.1929100
Cr	-0.0492580	0.4932810	-0.4003010
C	0.1873470	2.3681620	-0.1357270
C	0.3099670	3.6796860	0.6295580
C	-0.8730540	4.5471090	0.1257380
C	1.5888510	4.4593760	0.2949270
C	0.1903080	3.4728220	2.1436430
C	-0.2584460	0.2086330	-2.1153000
H	0.1504400	-3.9390030	0.2209060
H	-1.7039370	-5.4961390	-0.2321160
H	-4.4355490	-2.1984610	-0.6125660
H	-3.4429590	2.8925410	-0.8476790
H	-3.8805300	2.6589990	-2.5444000
H	-2.2036700	2.4001960	-2.0334950
H	-2.3632940	0.1493930	-3.2124640
H	-4.0704120	0.4013840	-3.5605270
H	-3.5654040	-1.0643590	-2.7011410
H	-4.4012360	-0.0692820	3.1079750
H	-3.8913540	-1.4045600	2.0602700
H	-5.0660840	-0.1977570	1.4721210
H	-4.2871730	2.2859370	1.1602980
H	-2.5682510	2.6911820	1.3974020
H	-3.5750100	2.2548590	2.7786030
H	4.3073390	-1.2080660	1.7238880
H	1.4341620	-3.2901750	4.1477450
H	-0.3648590	-2.6918350	2.5709320
H	3.0650560	-1.3917490	-4.0048220

H	3.7214830	0.0729040	-3.2618360
H	1.9595150	-0.1272640	-3.4415990
H	5.0594250	-1.4007960	-1.7529840
H	4.3005430	-2.9096570	-2.2563320
H	4.2659690	-2.4523850	-0.5493270
H	3.8273530	1.8920100	-2.2903020
H	4.1680910	3.1185080	-1.0627260
H	2.4984490	2.6137240	-1.3654510
H	4.0577860	2.6614750	1.3135460
H	3.8609300	1.0264080	1.9596910
H	2.4386490	1.9233320	1.4024680
H	-0.8314280	5.5226830	0.6318700
H	-1.8546420	4.1074740	0.3445230
H	-0.8087580	4.7317370	-0.9571560
H	1.6895910	4.6356400	-0.7860720
H	1.5333990	5.4425180	0.7839080
H	2.4936900	3.9641540	0.6563380
H	0.2534740	4.4447480	2.6537520
H	0.9994550	2.8422890	2.5393680
H	-0.7674600	3.0173080	2.4277990
H	0.3847370	2.5020990	-1.2192750
H	-0.1491050	1.0251380	-2.8457940
H	-0.4605070	-0.7775490	-2.5579400
H	-0.9092250	1.9930130	-0.0303430
C	-5.0843590	0.6625670	-1.1113210
H	-5.3469100	1.2375040	-0.2148320
H	-5.6891350	1.0622180	-1.9396470
H	-5.3951790	-0.3804910	-0.9607800
C	-1.7706070	0.2814450	2.4930880
H	-1.5431190	-0.7899620	2.5579700
H	-0.8631210	0.8062630	2.1438440
H	-1.9813400	0.6335370	3.5144280

C	5.1795510	0.6847350	-0.3179760
H	5.8265020	1.5051540	0.0304540
H	5.3979750	-0.1900290	0.3071280
H	5.4772820	0.4621260	-1.3496430
C	1.8053650	-2.4982380	-1.8928440
H	1.8299150	-3.0950710	-0.9708910
H	0.7790500	-2.1531690	-2.0541700
H	2.0578910	-3.1742710	-2.7236040
H	-4.0102340	-4.6397030	-0.6307760
H	3.7812930	-2.5767650	3.7131120
